# Effects of gE/gI deletions on the miRNA expression of PRV-infected PK-15 cells

**DOI:** 10.1007/s11262-020-01760-6

**Published:** 2020-05-08

**Authors:** Xiao Liu, Yuancheng Zhou, Yuan Luo, Yanxi Chen

**Affiliations:** 1 College of Animal Science and Technology, Southwest University, 2#Tiansheng Road, Beibei District, Chongqing, 400715 China; 2 Livestock and Poultry Biological Products Key Laboratory of Sichuan Province, Huashen Veterinary Biological Products Co., LTD, Chengdu, 610200 China

**Keywords:** Pseudorabies virus mutant strain, microRNA, Differential expression

## Abstract

Pseudorabies virus (PRV) belongs to the Alphaherpesvirinae subfamily of Herpesviridae. PRV-induced pseudorabies is a highly contagious disease that has caused huge economic losses to the global swine industry. The PRV gE/gI gene deletion vaccine strain (Fa ΔgE/gI strain) constructed from the PRV Fa wild-type strain was shown to have a protective effect against infection. However, the interaction between PRV gE/gI genes and host miRNA needs further exploration, and little is known about the regulatory mechanisms of non-coding RNAs during PRV infection. miRNAs play a key regulatory role in viral infection and immune responses, so we analyzed the differential expression of miRNAs induced by the PRV Fa ΔgE/gI strain and Fa wild-type strain in the PK15 cell line. High-throughput sequencing reads were aligned to known *Sus scrofa* pre-miRNAs in the miRBase database. Target genes of differentially expressed miRNAs were predicted using the miRGen 3.0 database, then filtered miRNA target genes were subjected to Gene Ontology (GO) analysis and Search Tool for the Retrieval of Interacting Genes/ Proteins (STRING) analysis. Stem-loop quantitative real-time PCR was performed to confirm the accuracy of high-throughput sequencing data. In total, 387, 472, and 490 annotated and novel mature miRNAs were identified from PRV Fa ΔgE/gI strain-infected, Fa wild-type strain-infected, and non-infected PK-15 cells, respectively. Five PRV-encoded miRNAs were also identified. GO analysis showed that target genes of differentially expressed miRNAs in PRV Fa ΔgE/gI strain-infected and Fa wild-type strain-infected PK-15 cells were mainly involved in biological regulation and metabolic processes. STRING analysis showed that immune-related target genes of differentially expressed miRNAs in the Toll-like receptor signaling pathway, B cell receptor signaling pathway, T cell receptor signaling pathway, nuclear factor-κB signaling pathway, and transforming growth factor-β signaling pathway were interrelated. This is the first report of the small RNA transcriptome in PRV mutant wild-type strain-infected and Fa ΔgE/gI strain-infected porcine cell lines. Our findings will contribute to the prevention and treatment of PRV mutant strains.

**Electronic supplementary material:**

The online version of this article (10.1007/s11262-020-01760-6) contains supplementary material, which is available to authorized users.

## Background

Pseudorabies virus (PRV) is a member of the Alphaherpesvirinae subfamily of the Herpesviridae family*.* It is a double-stranded linear DNA virus with a 150 kb genome that encodes approximately 100 proteins. The PRV viral envelope contains 11 glycosylation-modified membrane proteins, including essential glycoproteins gB, gD, gH, gL, and non-essential glycoproteins gC, gE, gI, gG, gM, gN, and gK. The gE protein is a key virulence protein of PRV, and the functional complexes of gE and gI are required for efficient anterograde axonal transport of PRV particles in neurons [1]. PRV demonstrates neurotropism and transsynaptic transmission, so has been developed into an effective vector system for the integration and expression of foreign proteins, as well as neural tracing [2–5].

PRV is widely distributed around the world. All strains and ages of pigs are susceptible to PRV, as well as a variety of domestic and wild animals. PRV spreads throughout the respiratory and reproductive systems, and PRV-infected pigs and mice are the main sources of infection [6]. Sows infected with PRV show clinical symptoms of abortion, and infected newborn piglets have severe neurological symptoms, with morbidity and mortality near 100% for those younger than 2 weeks old. PRV mutant strains also cause severe respiratory symptoms in adult pigs, and cause reproductive failure in boar [7–13].

Pseudorabies was effectively controlled using the PRV Bartha-K61 vaccine strain prior to 2011, but the prevalence of PRV mutants has reduced its immunoprotective effects and greatly increased the mortality of neonatal piglets, which has caused huge economic losses to China’s farming industry [6]. Additionally, the co-infection of PRV mutants with immunosuppressive classical swine fever virus (CSFV), porcine reproductive and respiratory syndrome virus (PRRSV), porcine circovirus, and other opportunistic pathogens has increased the difficulty of preventing PRV mutants. The porcine kidney cell line PK-15 and pig testicular cell line ST are used for the isolation and culture of PRV. Recently, the gE/gI gene deletion vaccine strain (FaΔgE/gI strain) based on the PRV Fa wild-type strain was used to prevent the spread of PRV mutants in China, and was confirmed to have a protective effect against infection [6, 14–16].

microRNAs (miRNAs) are endogenous non-coding RNAs 20–25 bp in length that were originally found in eukaryotes. They play an important regulatory role in a variety of physiological processes including apoptosis, cell differentiation, fat metabolism, development, and cancer. Recent studies showed that mammalian-encoded miRNAs regulate host–virus interactions by targeting viral or host genomes. miRNAs were also shown to be involved in the regulation of host immune responses and to function in the antiviral process. As well as eukaryotes, a series of viruses have been confirmed to encode miRNAs. Since the Epstein Barr virus was first reported to encode miRNAs, 569 viral-encoded miRNAs have been annotated by the miRBase 22.0 database, of which most are encoded by viruses belonging to the Herpesviridae family [17–20].

Latent infection is the main reason it is difficult to eradicate PRV, and glycoproteins gE and gI play a key role in PRV latent infection and invasion of the host nervous system. Viral-encoded miRNAs mediate silencing of host-encoded genes by targeting mRNAs, thus evading the host immune system recognition and clearance and leading to long-term latent infection in the host. Therefore, an investigation of the regulatory mechanisms of host- and viral-encoded miRNAs during PRV latent infection and immune evasion will contribute to a better understanding of PRV pathogenic mechanisms.

In this study, we comprehensively analyzed the miRNA expression profiles of PK-15 cells infected with the PRV gE/gI gene deletion vaccine strain (Fa ΔgE/gI strain) and PRV Fa wild-type strain. A series of annotated and novel miRNAs from infected and non-infected samples were identified, and the potential regulatory effect of gE/gI genes on miRNA expression was explored. Our findings provide a theoretical basis for the development of novel PRV recombinant vaccines and neural tracing vectors.

## Methods

### Data sources

The full-genomic sequence of the PRV Fa strain is available in GenBank (https://www.ncbi.nlm.nih.gov/genbank/). *Sus scrofa* genomic sequence information is available from the University of California Santa Cruz genome browser (https://genome.ucsc.edu/index.html). Annotated pig-encoded miRNAs are available from miRBase 22.1 (https://www.mirbase.org/) and Ensembl databases (https://www.ensembl.org/index.html).

### PRV Fa ΔgE/gI strain- and Fa wild-type strain-infected cells and RNA isolation

The PRV Fa ΔgE/gI strain and Fa wild-type strain were used in this research. A total of 10^6^ PK-15 cells were maintained in 175 cm^2^ dishes in modified RPMI-1640 nutrient solution (Thermo Fisher Scientific, Waltham, MA) supplemented with 50 mg/ml penicillin/streptomycin antibiotic solution and 10% fetal bovine serum (Thermo Fisher Scientific) at 37 °C with 5% CO_2_.

PK-15 cells at 80% confluency were infected with PRV Fa ΔgE/gI strain or Fa wild-type strain (multiplicity of infection = 1). PRV Fa ΔgE/gI strain-infected, Fa wild-type strain-infected, and non-infected PK-15 cells were harvested at 72 h post infection.

Total RNA from each sample was extracted using Trizol reagent (Thermo Fisher Scientific, Shanghai, China), and the concentration and purity of RNA samples were determined by the NanoDrop ND-1000 spectrophotometer (Nano Drop Inc., Wilmington, DE, USA). The integrity of total RNA samples was determined by the Agilent 2100 Bioanalyzer system (Agilent Technologies, Santa Clara, CA, USA).

### miRNA library construction and sequencing

Total RNA from PRV Fa ΔgE/gI strain-infected, Fa wild-type strain-infected, and non-infected PK-15 cells was ligated to 3′ and 5′ adapters with T4 RNA ligase. cDNA was synthesized and amplified using RT primers and amplification primers (Illumina, San Diego, CA, USA). PCR-amplified products of 120–140 bp were purified, and the complete libraries were tested by the Agilent 2100 Bioanalyzer (Agilent Technologies). cDNA samples were adjusted to 8 pM, then cluster generation was sequentially performed on the Illumina cBot system (Illumina). High-throughput sequencing was performed on an Illumina HiSeq 2000 using TruSeq Rapid SBS Kits (Illumina), according to the manufacturer’s instructions.

### miRNA data analysis

Total raw sequencing reads were filtered by the Solexa CHASTITY quality control filter, and aligned to miRBase porcine pre-miRNA sequences (https://www.mirbase.org/) using Novoalign software. Reads shorter than 15 nt and ribosomal RNA (rRNA), small nuclear RNA (snRNA), transfer RNA (tRNA), and small nucleolar RNA (snoRNA) data were discarded. Novel pig-encoded miRNAs and PRV-encoded miRNAs were predicted by the miRDeep2 web server, and the palindrome structures of miRNAs were analyzed by Mfold software (https://mfold.rna.albany.edu/). Differentially expressed (DE) miRNAs were determined by fold-change filtering, with fold-change values (log_2_) ≥ 2 identified as significantly up-regulated, and fold-change values (log_2_) ≥ 0.5 as significantly downregulated. DE miRNA target genes were predicted by the miRGen 3.0 database (https://www.diana.pcbi.upenn.edu/miRGen.html). Gene Ontology (GO) analysis of DE miRNA target genes was performed using the Database for Annotation, Visualization and Integrated Discovery (https://david.abcc.ncifcrf.gov/) and WEGO software (https://wego.genomics.org.cn/), and GO terms with a *p* value ≤ 0.05 were determined to be significant. The potential interaction between target genes of DE miRNAs was predicted by the online database Search Tool for the Retrieval of Interacting Genes/ Proteins (STRING).

### Stem-loop quantitative real-time PCR (qRT-PCR) validation of DE miRNAs

Stem-loop qRT-PCR was used to confirm the differential expression of ssc-miR-10b, ssc-miR-30a-5p, ssc-miR-21, and ssc-let-7f in Fa wild-type strain0infected PK-15 cells, and ssc-miR-182, ssc-miR-192, ssc-miR-19b, and ssc-miR-24-3p in PRV Fa ΔgE/gI strain-infected PK-15 cells. The expression levels of PRV-encoded ssc-miR-novel-chrPRV_425, ssc-miR-novel-chrPRV_428, ssc-miR-novel-chrPRV_434, ssc-miR-novel-chrPRV_435, and ssc-miR-novel-chrPRV_441 were also confirmed by stem-loop qRT-PCR. Experiments were performed in triplicate on the ABI Prism 7900HT sequencing detection system (Applied Biosystems, Foster City, CA, USA). Data analyses were performed using a two-tailed Student’s *t* test.

## Results

### Overview of high-throughput sequencing data

To obtain miRNA transcriptome of a PRV-infected porcine cell line, PK-15 cells were infected with PRV Fa wild-type and Fa ΔgE/gI strains. PRV infection was confirmed by PCR, and miRNA expression profiles were generated using the Illumina HiSeq 2000 platform. Clean reads, adapter-trimmed reads, and reads aligned to known *S. scrofa* pre-miRNAs of PRV Fa wild-type strain-infected, PRV Fa ΔgE/gI strain-infected, and non-infected PK-15 cells were generated (Table 1).

**Table 1:** Overview of miRNA transcriptome data

Samples	Clean reads	Adapter-trimmed reads (length ≥ 15nt)	Reads aligned to known *Sus scrofa* pre-miRNA in miRBase datebase
Non-infected PK-15 cells	3,987,148	3,575,737	2,215,640
Fa wild strain-infected PK-15 cells	4,185,319	3,660,767	867,380
PRV FaΔgE/gI strain-infected PK-15 cells	5,866,080	4,747,382	1,068,777

**Fig. 1:**
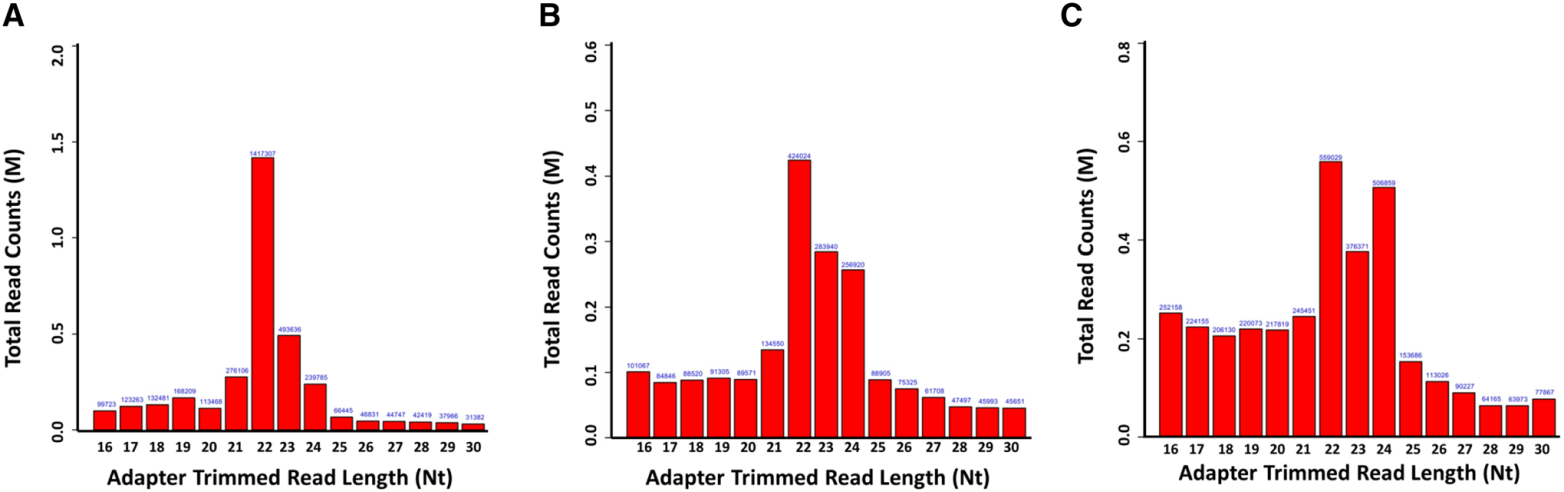
Length distribution of total small RNAs. **a** Read counts against read lengths for the complete adapter-trimmed read set in non-infected PK-15 cells. **b** Read counts against read lengths for the complete adapter-trimmed read set in Fa wild-type strain-infected PK-15 cells. **c** Read counts against read lengths for the complete adapter-trimmed read set in PRV Fa ΔgE/gI strain-infected PK-15 cells

The analysis of adapter-trimmed reads in small (s) RNA-sequencing libraries showed that the lengths of sRNAs (miRNAs, tRNAs, snoRNAs, snRNAs, and rRNAs) were concentrated around 21–24 nt (Fig. 1, Fig. S1), miRNA expression was significantly suppressed under PRV infection (Table 2).

**Table 2:** Small RNA percentages

RNA category	Non-infected PK-15 cells (%)	Fa wild strain-infected PK-15 cells (%)	PRV Fa ΔgE/gI strain-infected PK-15 cells (%)
rRNA	22.00	29.60	34.80
tRNA	4.00	32.50	21.10
miRNA	71.30	32.90	37.90
snRNA	1.80	2.70	3.50
Other ncRNA	0.90	2.20	2.70

**Fig. 2:**
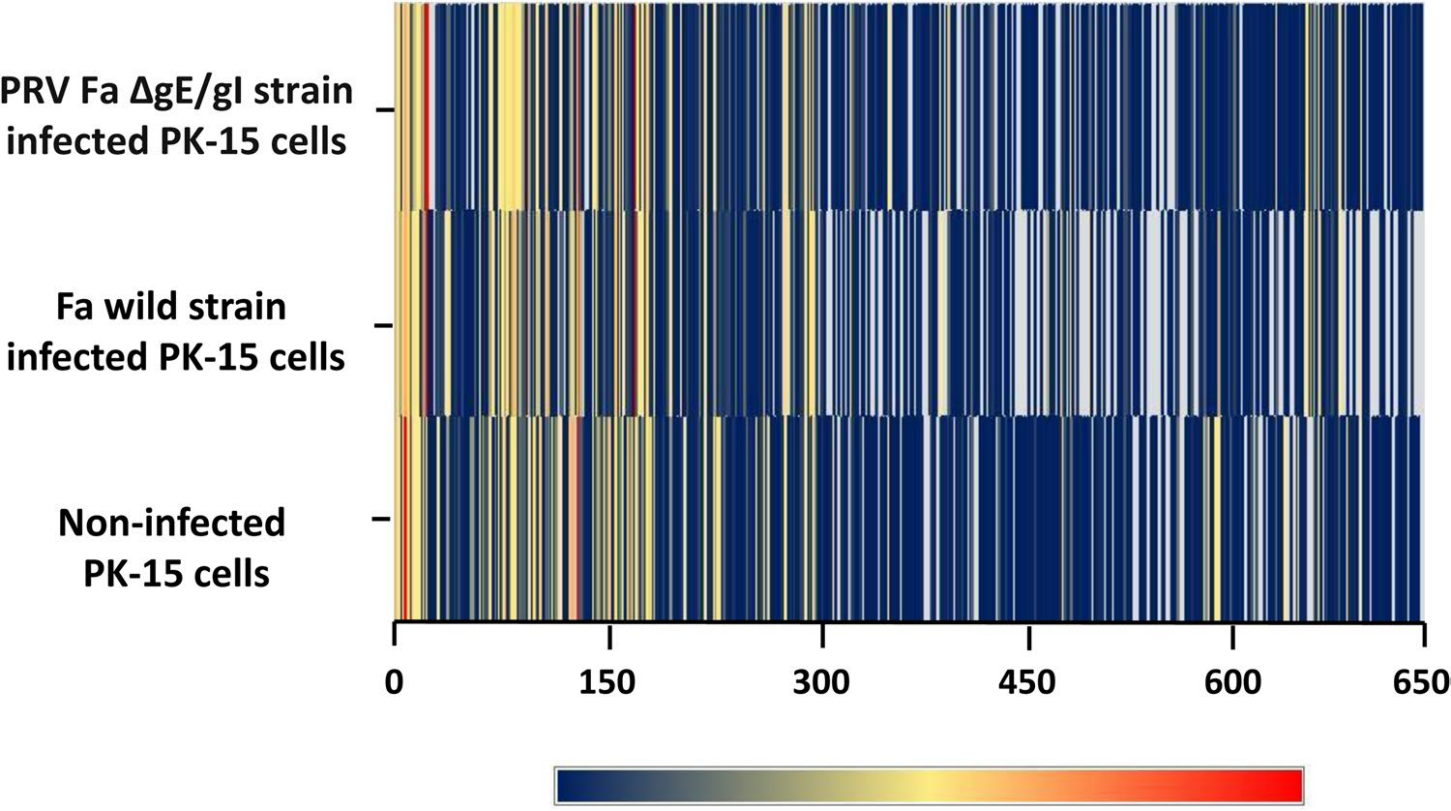
Heat map and hierarchical clustering of miRNA high-throughput sequencing data. Heat map and hierarchical clustering was used to analysis the miRNA high-throughput sequencing data of non-infected PK-15 cells, Fa wild-type strain-infected PK-15 cells, and PRV Fa ΔgE/gI strain-infected PK-15 cells base on their expression level. The red line indicates high relative expression and the green line indicates low relative expression

**Fig. 3:**
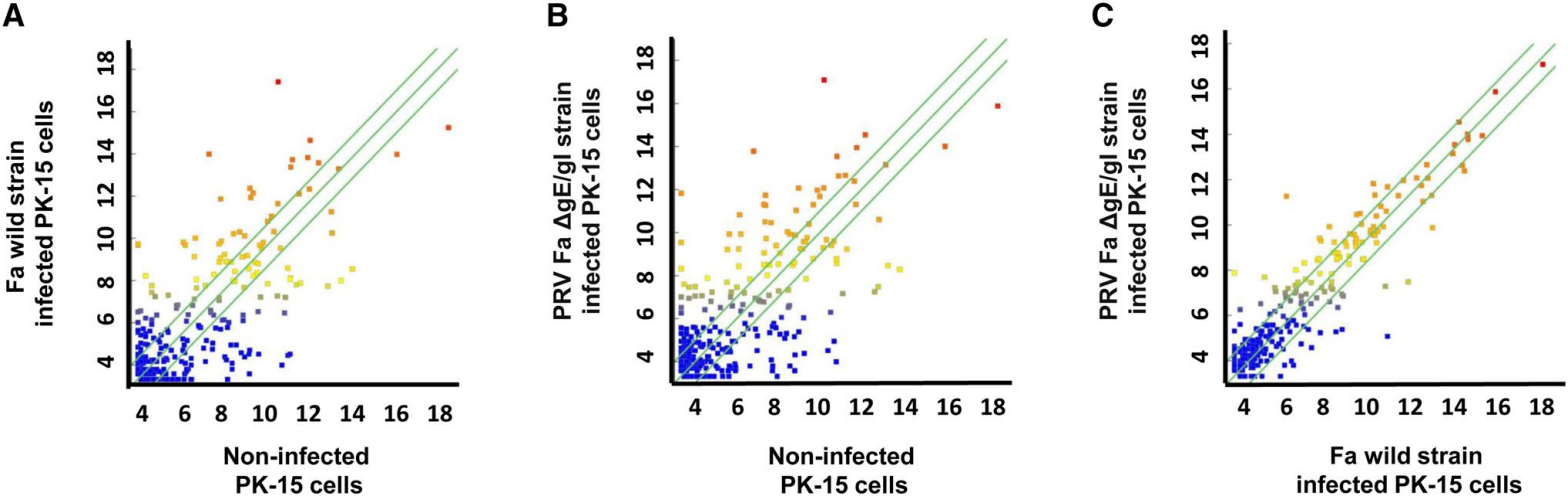
Scatter plot of miRNA high-throughput sequencing data. **a** Scatter plot showing variations in miRNA expression profiles between non-infected PK-15 cells and Fa wild-type strain-infected PK-15 cells. **b** Scatter plot showing variations in miRNA expression profiles between non-infected PK-15 cells and PRV Fa ΔgE/gI strain-infected PK-15 cells. **c** Scatter plot showing variations in miRNA expression profiles between Fa wild-type strain-infected PK-15 cells and PRV Fa ΔgE/gI strain-infected PK-15 cells. The high-throughput sequencing data are graphed on a scatter plot to visualize variations in miRNAs expression. The values on the X and Y axes of the scatter plot are the normalized signal values for the samples (log2 scaled). The green lines are fold-change lines (the default fold-change value is 2.0). The expression of the miRNAs above the top green line or below the bottom green line differed more than two-fold

High-throughput sequencing data were aligned to the reference *S. scrofa* genomic sequence and gene annotation data after processing using Illumina’s Genome Analyzer. A total of 387 (217 annotated and 170 novel), 472 (226 annotated and 246 novel), and 490 (241 annotated and 249 novel) mature miRNAs were identified in transcripts from PRV Fa wild-type strain-infected, PRV Fa ΔgE/gI strain-infected, and non-infected PK-15 cells, respectively. We also identified five PRV-encoded miRNAs (ssc-miR-novel-chrPRV_425, ssc-miR-novel-chrPRV_428, ssc-miR-novel-chrPRV_434, ssc-miR-novel-chrPRV_435, and ssc-miR-novel-chrPRV_441) in PRV Fa wild-type strain-infected and PRV Fa ΔgE/gI strain-infected PK-15 cells (Table S1, Figs. 2, 3).

### Analysis of significantly DE miRNAs

According to the miRNA expression analysis of high-throughput sequencing data, most mature miRNAs are expressed by a small number of miRNA genes. We found that 2.6% (10/387) of miRNAs accounted for 72.5% of total miRNA expression in PRV Fa wild-type strain-infected PK-15 cells, while 2.1% (10/472) of miRNAs accounted for 75.5% of total miRNA expression in PRV Fa ΔgE/gI strain-infected PK-15 cells. In all sequencing profiles, ssc-miR-21 and ssc-let-7f had extremely high expression levels (Table S1, Fig. S2).

**Fig. 4:**
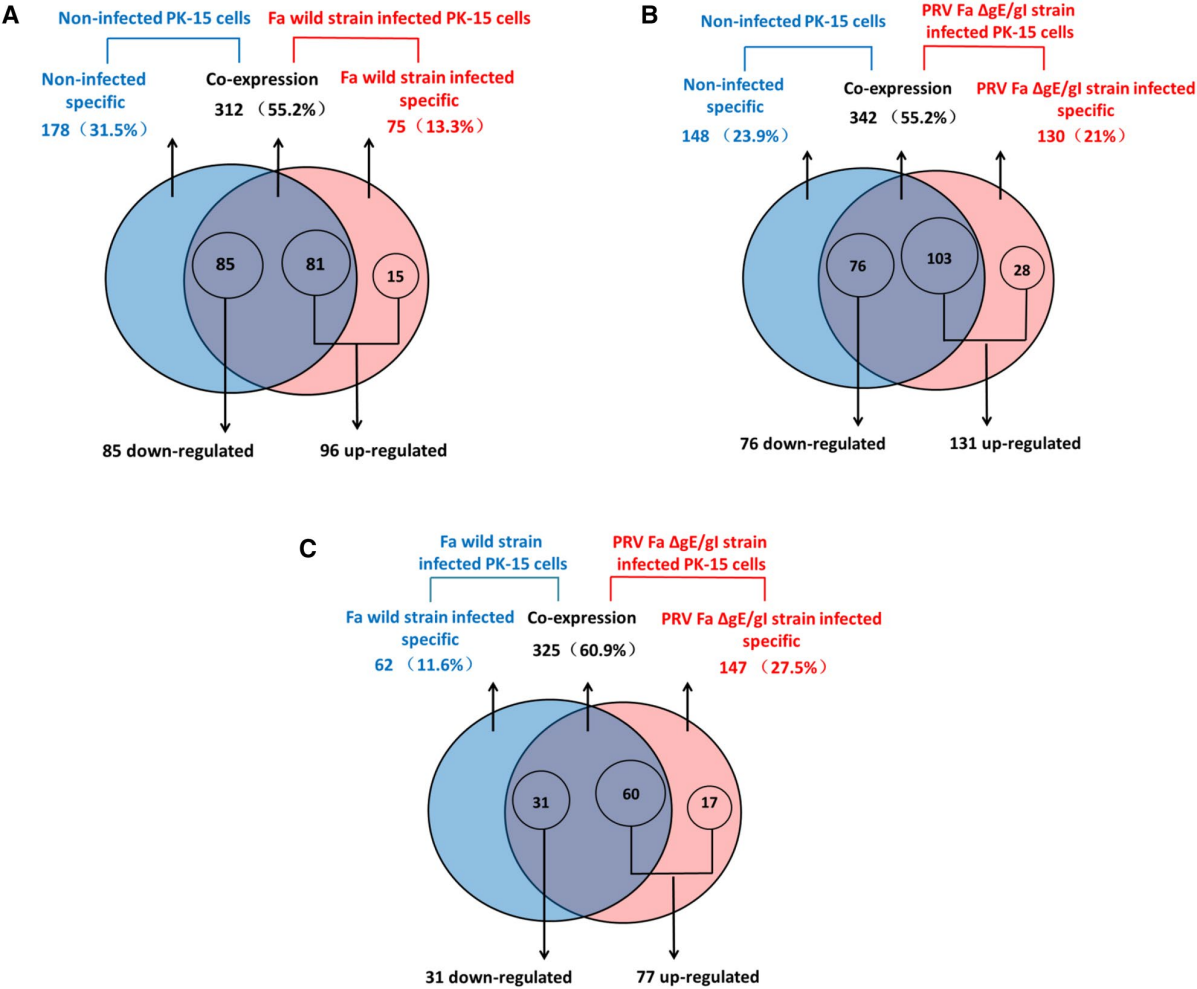
miRNA differential expression analysis. **a** Venn diagram showing the distribution of 565 unique miRNAs between non-infected PK-15 cells and Fa wild-type strain-infected PK-15 cells. A total of 181 DE miRNAs were identified in Fa wild-type strain-infected PK-15 cells. **b** Venn diagram showing the distribution of 620 unique miRNAs between non-infected PK-15 cells and PRV Fa ΔgE/gI strain-infected PK-15 cells. A total of 207 DE miRNAs were identified in PRV Fa ΔgE/gI strain-infected PK-15 cells. **c** Venn diagram showing the distribution of 534 unique miRNAs between Fa wild-type strain-infected PK-15 cells and PRV Fa ΔgE/gI strain-infected PK-15 cells. A total of 108 DE miRNAs were identified in PRV Fa ΔgE/gI strain-infected PK-15 cells

A total of 312 (55.2%) miRNAs were co-expressed in PRV Fa wild-type strain-infected PK-15 cells and non-infected PK-15 cells. However, 75 (13.3%) and 178 (31.5%) miRNAs were specifically expressed in PRV Fa wild-type strain-infected PK-15 cells and non-infected PK-15 cells, respectively. A total of 181 pig-encoded miRNAs were significantly DE between PRV Fa wild-type strain-infected PK-15 cells and non-infected PK-15 cells. Of these, 85 (47%) were significantly upregulated in PRV Fa wild-type strain-infected PK-15 cells, while 96 (53%) were significantly downregulated (Fig. 4, Table S2).

A total of 342 (55.2%) miRNAs were co-expressed in PRV Fa ΔgE/gI strain-infected PK-15 cells and non-infected PK-15 cells, and 130 (21%) and 148 (23.9%) miRNAs were specifically expressed in PRV Fa ΔgE/gI strain-infected PK-15 cells and non-infected PK-15 cells, respectively. A total of 207 pig-encoded miRNAs were significantly DE between PRV Fa ΔgE/gI strain-infected PK-15 cells and non-infected PK-15 cells. Of these, 76 (36.7%) were significantly upregulated in PRV Fa ΔgE/gI strain-infected PK-15 cells, while 131 (63.3%) were significantly downregulated (Fig. 4, Table S2).

To further investigate the effect of the deletion of gE/gI genes on miRNA expression levels, we compared miRNA expression profiles in PRV Fa wild-type strain-infected and Fa ΔgE/gI strain-infected PK-15 cells. We found that 325 (60.9%) miRNAs were co-expressed in Fa wild-type strain-infected and Fa ΔgE/gI strain-infected PK-15 cells, and that 147 (27.5%) and 62 (11.6%) miRNAs were specifically expressed in PRV Fa ΔgE/gI strain-infected PK-15 cells and Fa wild-type strain-infected PK-15 cells, respectively. A total of 77 miRNAs expressed in PRV Fa ΔgE/gI strain-infected PK-15 cells were significantly upregulated compared with PRV Fa wild-type strain-infected PK-15 cells, while 31 miRNAs expressed in PRV Fa ΔgE/gI strain-infected PK-15 cells were significantly downregulated. This indicates that the expression of these DE miRNAs is associated with the deletion of gE or gI genes (Fig. 4, Table S2).

### Stem-loop qRT-PCR confirmation of DE miRNAs

Stem-loop qRT-PCR was used to validate the expression levels of DE miRNAs (ssc-miR-10b, ssc-miR-30a-5p, ssc-miR-21, ssc-let-7f, ssc-miR-182, ssc-miR-192, ssc-miR-19b, and ssc-miR-24-3p) in PRV Fa ΔgE/gI strain-infected or Fa wild-type strain-infected PK-15 cells. The expression levels of five novel PRV-encoded miRNAs (ssc-miR-novel-chrPRV_425, ssc-miR-novel-chrPRV_428, ssc-miR-novel-chrPRV_434, ssc-miR-novel-chrPRV_435, and ssc-miR-novel-chrPRV_441) were also confirmed. Stem-loop qRT-PCR results were generally consistent with those of high-throughput sequencing (Table 3, Table S3).

**Table 3:** Comparison of qRT-PCR and high-throughput sequencing results

Group	miRNA	Pre-miRNA	qRT-PCR fold-change	High-throughput sequencing fold-change
PRV Fa wild strain-infected/non-infected	ssc-miR-10b	ssc-mir-10b	+ 45.81	+ 149.16
PRV Fa wild strain-infected/non-infected	ssc-miR-30a-5p	ssc-mir-30a	+ 5.21	+ 16.31
PRV Fa wild strain-infected/non-infected	ssc-miR-21	ssc-mir-21	− 0.18	− 0.1
PRV Fa wild strain-infected/non-infected	ssc-let-7f	ssc-let-7f	− 0.36	− 0.24
PRV FaΔgE/gI strain-infected/non-infected	ssc-miR-182	ssc-miR-182	+ 3.37	+ 5.18
PRV FaΔgE/gI strain-infected/non-infected	ssc-miR-192	ssc-mir-192	+ 20.96	+ 125.52
PRV FaΔgE/gI strain-infected/non-infected	ssc-miR-19b	ssc-miR-19b	− 0.11	− 0.02
PRV FaΔgE/gI strain-infected/non-infected	ssc-miR-24-3p	ssc-mir-24	− 0.24	− 0.02
PRV FaΔgE/gI strain-infected/PRV Fa wild strain-infected	ssc-miR-novel-chrPRV_425	ssc-mir-novel-chrPRV_425	+ 7.8	+ 4.48
PRV FaΔgE/gI strain-infected/PRV Fa wild strain-infected	ssc-miR-novel-chrPRV_428	ssc-mir-novel-chrPRV_428	− 0.84	− 0.87
PRV FaΔgE/gI strain-infected/PRV Fa wild strain-infected	ssc-miR-novel-chrPRV_434	ssc-mir-novel-chrPRV_434	− 0.7	− 0.35
PRV FaΔgE/gI strain-infected/PRV Fa wild strain-infected	ssc-miR-novel-chrPRV_435	ssc-mir-novel-chrPRV_435	− 0.39	− 0.73
PRV FaΔgE/gI strain-infected/PRV Fa wild strain-infected	ssc-miR-novel-chrPRV_441	ssc-mir-novel-chrPRV_441	+ 2.79	+ 1.5

Fold-change cutoffs of upregulated and downregulated miRNAs were + 2 and − 0.5, respectively

### DE miRNA target prediction and functional annotation

To further investigate the potential regulatory roles of DE miRNAs and PRV-encoded miRNAs on host-encoded genes, the microT-CDS database (https://diana.imis.athena-innovation.gr/DianaTools/index.php?r=microT_CDS/index) was used to predict target genes and target sites of miRNAs. We showed that PRV-encoded ssc-miR-novel-chrPRV_425, ssc-miR-novel-chrPRV_428, ssc-miR-novel-chrPRV_434, ssc-miR-novel-chrPRV_435, and ssc-miR-novel-chrPRV_441 targeted 1943, 1309, 195, 2131, and 680 pig-encoded genes (Fig. S3), respectively. A series of pig-encoded DE miRNAs were also shown to target pre-miRNAs encoded by the host, such as the significantly downregulated miRNA ssc-miR-19a in PRV Fa ΔgE/gI strain-infected and Fa wild-type strain-infected PK-15 cells, which was predicted to target pre-miRNA ssc-mir-21 (Table S4).

**Fig. 5:**
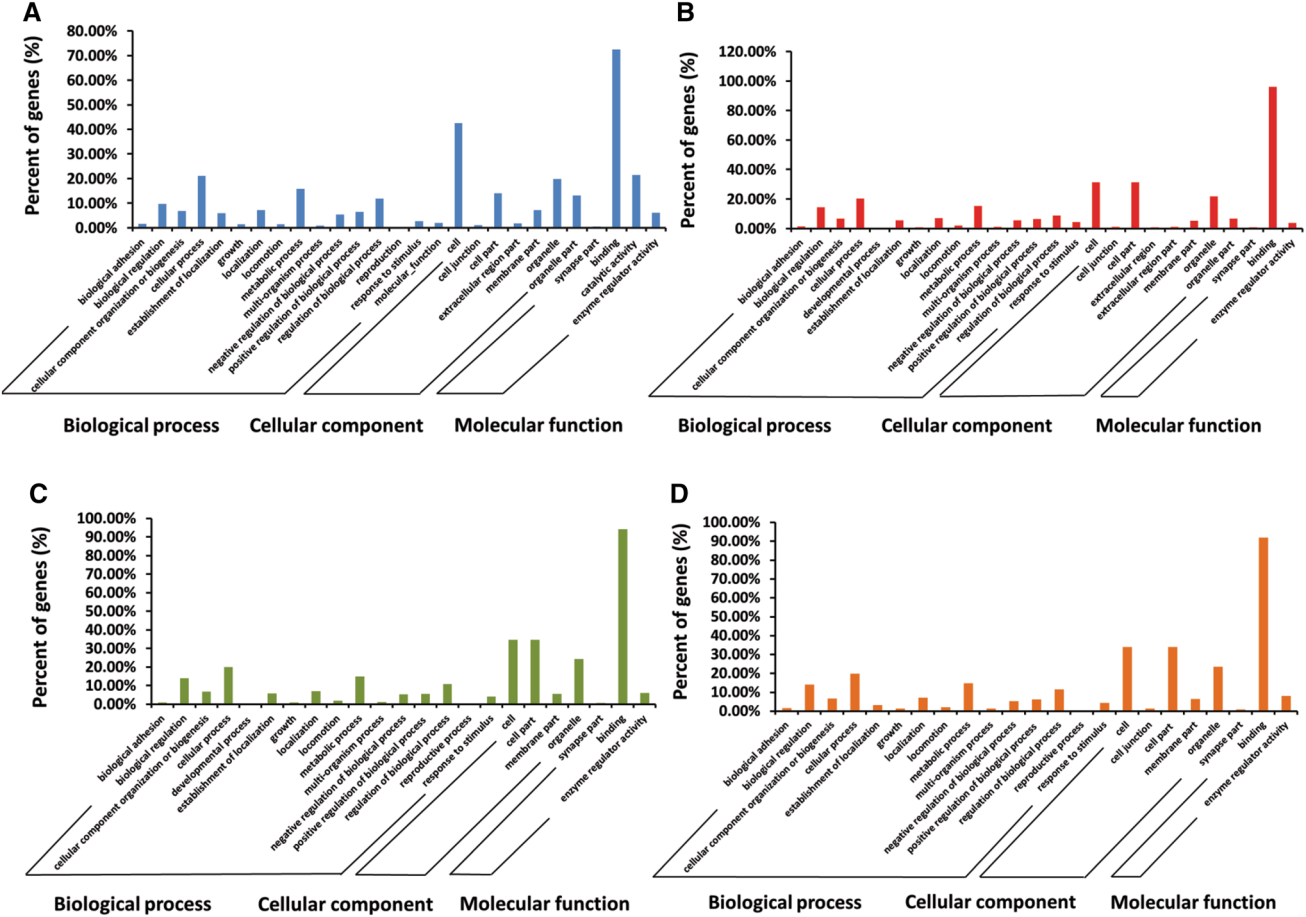
GO annotation of miRNA target genes. **a** GO annotation of DE miRNA target genes in Fa wild-type strain-infected PK-15 cells (vs. non-infected). **b** GO annotation of DE miRNA target genes in PRV Fa ΔgE/gI strain-infected PK-15 cells (vs. non-infected). **c** GO annotation of DE miRNA target genes in PRV Fa ΔgE/gI strain-infected PK-15 cells (vs. Fa wild-type strain-infected). **d** GO annotation of target genes of PRV-encoded miRNAs

GO functional analysis showed that the target genes of DE miRNAs in Fa wild-type strain-infected or PRV Fa ΔgE/gI strain-infected PK-15 cells were significantly associated with cellular process, metabolic process, regulation of biological process, and enzyme regulator activity (Fig. 5). Moreover, the target genes of DE (Fa ΔgE/gI strain-infected vs Fa wild-type strain-infected) miRNAs in PRV Fa ΔgE/gI strain-infected PK-15 cells were mainly related to biological regulation, cellular process, and metabolic process (Fig. 5).

### STRING analysis

**Fig. 6:**
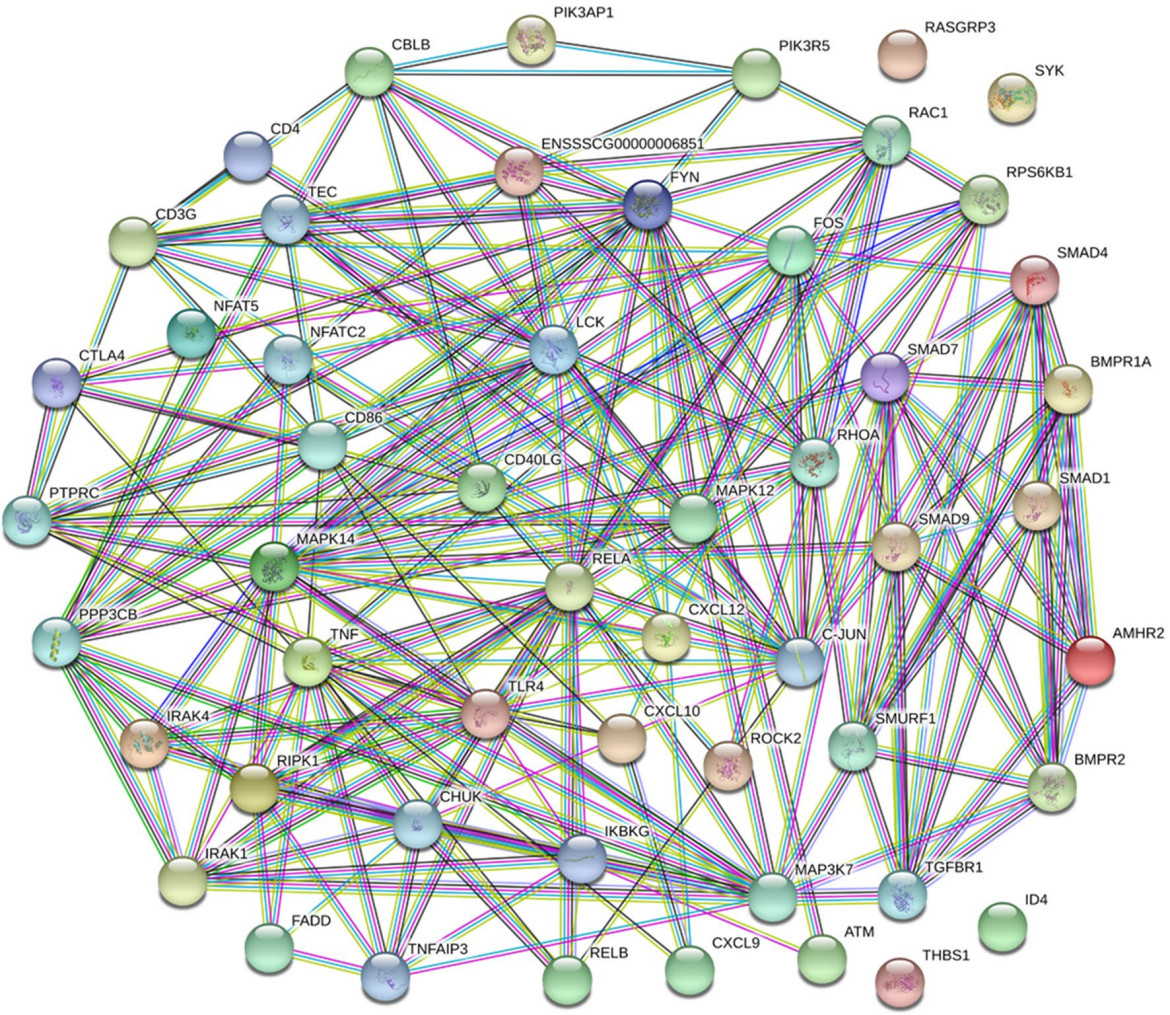
STRING analysis of immune-related target genes of DE miRNAs. The STRING database was used to analysis the relationships between the immune-related pathway target genes of DE miRNAs in PRV Fa ΔgE/gI strain-infected PK-15 cells and Fa wild-type strain-infected PK-15 cells. Different lines represent the types of evidence upon which the associations are based. Green: neighborhood evidence; red: gene fusion evidence; black: co-expression evidence; purple: database evidence; cyan: textmining evidence; yellow: homology evidence; blue: co-occurrence evidence

The STRING database was used to analyze the physical and functional interaction between immune-related pathway target genes of DE miRNAs in PRV Fa ΔgE/gI strain-infected and Fa wild-type strain-infected PK-15 cells. The functional protein-associated network showed that immune-related target genes of DE miRNAs in the Toll-like receptor signaling pathway, B cell receptor signaling pathway, T cell receptor signaling pathway, nuclear factor-κB signaling pathway and transforming growth factor-β signaling pathway are interrelated (Fig. 6). For example, target genes of DE miRNAs encoding the T-cell surface glycoprotein CD4, T-cell surface glycoprotein CD3 gamma chain, mitogen-activated protein kinase 12, C-X-C motif chemokine ligand 12 (CXCL12), CXCL9, CXCL10, tumor necrosis factor, and Toll-like receptor 4 were interrelated. However, a series of target genes of DE miRNAs encoding proteins were located outside the functional protein-associated network, such as spleen-associated tyrosine kinase, thrombospondin 1, inhibitor of DNA binding 4, and RAS guanyl releasing protein 3.

## Discussion

Porcine pseudorabies is one of the major infectious diseases caused by PRV, and is extremely harmful to the global pig breeding industry. Co-infection of PRV with PRRSV, CSFV, and other opportunistic pathogens increases the difficulty of prevention and treatment of pseudorabies in pigs. Additionally, the prevalence of the PRV Fa wild-type strain has caused great obstacles to the prevention of PRV in recent years [21].

Latent infection is a common feature of herpes virus, and is the main reason why PRV is difficult to cure. PRV mainly locates in the host’s trigeminal nerve, tonsils, and lungs, where it induces the programmed cell death of host immune cells and inhibits that of PRV-infected cells, thus promoting viral proliferation. Latent PRV infection in neurons is activated when the host’s immunity is weakened or in response to stress [22]. Currently, attenuated live vaccine immunity is still the major method for the prevention and treatment of pseudorabies. The PRV gE/gI gene deletion vaccine not only effectively induces the host’s immune response, but also inhibits the latent infection of PRV wild-type strains. The PRV recombinant virus can also be used as a tracer marker of neural circuits for the study of neural networks because of the neurotropic and trans-synaptic transmission of PRV [3].

gE/gI functional complexes present in the viral envelope and infected host cell membrane affect invasion of the nervous system by PRV [1]. Therefore, the study of gE and gI genes helps elucidate PRV latent infection mechanisms in the nervous system. To investigate the pathogenesis of PRV and explore the potential regulatory roles of non-coding RNAs in PRV infection, we constructed PK-15 cell models infected with a PRV Fa mutant wild-type strain and a gE/gI deletion vaccine strain, and analyzed host miRNA expression profiles in response to infection.

High-throughput sequencing showed that miRNA expression was significantly decreased under the infection pressure of the PRV Fa mutant wild-type strain and PRV FaΔgE/gI strain (Table 1). This is in-keeping with our previous result that transmissible gastroenteritis virus infection in PK-15 cells decreased miRNA expression, and the findings that miRNA expression in cancer tissues is significantly inhibited compared with healthy tissues [23, 24]. This indicates that miRNA expression is closely associated with physiological processes, PRV may affect the immune response by reducing the level of miRNA transcription in the host. The present study also showed that 46.8% and 43.9% miRNAs were significantly DE in PRV Fa wild-type strain-infected and PRV Fa ΔgE/gI strain-infected PK-15 cells compared with non-infected PK-15 cells, respectively, suggesting that PRV invasion significantly affected host miRNA expression.

Sequencing results showed that ssc-miR-21, ssc-miR-10b, ssc-miR-10a-5p, ssc-let-7a, ssc-let-7f, and ssc-miR-30a-5p miRNAs had the highest expression in each cell sample (Table S1), which agrees with previous findings in various mammals, suggesting that these miRNAs are conserved under PRV infection [25, 26]. Among these miRNAs, host-encoded ssc-miR-10a-5p can reduce PRRSV replication by inhibiting the expression of the host-encoded signal recognition particle 14 gene, while miR-10a was reported to inhibit the activation of T helper (Th)1/Th7 cells by targeting interleukin (IL)-12/ IL-23p40 [27, 28].

ssc-miR-10b was one of the most highly expressed miRNAs of all sequencing profiles in the present study, and was significantly up-regulated in PRV Fa wild-type strain-infected and PRV FaΔgE/gI strain-infected PK-15 cells. Conversely, ssc-miR-21 was significantly downregulated during PRV Fa wild-type strain and PRV FaΔgE/gI strain infection. miR-21 has been reported to participate in cancer processes, and to significantly promote cell proliferation and inhibit apoptosis, indicating that the miR-10 family and miR-21 play critical roles during PRV infection. Recent studies also showed that miR-146a promotes viral replication in cells by inhibiting the production of interferon-β [29]. We found that ssc-miR-146a-5p expression was significantly downregulated following infection with PRV Fa wild-type strain and PRV Fa ΔgE/gI strain, suggesting that PRV regulates viral replication levels by affecting the expression of host-encoded ssc-miR-146a-5p.

To explore the association between PRV gE and gI genes with miRNA expression profiles, we compared miRNA expression profiles of PRV Fa ΔgE/gI strain-infected and PRV Fa wild-type strain-infected PK-15 cells. This revealed that 108 miRNAs were significantly DE under gE/gI deletion (Fig. 4), indicating that gE and gI genes affect PRV-to-host infection by interfering with miRNA expression. This is in agreement with previous work showing that a series of miRNAs are significantly DE in different viral-infected cell lines or tissues [26, 30–32], indicating that these miRNAs play similar regulatory roles during viral infection (Table S5). The host-encoded miR-182 was significantly upregulated in PRV Fa ΔgE/gI strain-infected PK-15 cells, while miR-182 inhibits virus replication through activation of type I IFN response by targeting FOXO3 in neural cells [33], this suggested that upregulation of miR-182 after gE/gI deletion may inhibit PRV replication in the host. Recent research showed that miR-24-3p promotes virus replication through suppression of heme oxygenase-1 expression [34], this suggested that the significant down-regulation of miR-24-3p after gE/gI deletion may inhibit PRV infection. Further research will contribute to understand the roles of miRNA in PRV infection.

We also observed that five PRV-encoded miRNAs identified in PRV Fa ΔgE/gI strain-infected and PRV Fa wild-type-strain-infected PK-15 cells were located in the PRV large latency transcript region, and GO analysis showed that the target genes of these miRNAs participate in multiple biological processes (Fig. 5). PRV-encoded ssc-miR-novel-chrPRV_425 was significantly up-regulated after gE/gI deletion, therefore, ssc-miR-novel-chrPRV_425 may be regulated by gE/gI functional complexes and affect the virulence and replication of PRV.

## Conclusion

This study is the first to analyze miRNA expression profiles in hosts infected with PRV gE/gI gene deletion and PRV Fa wild-type strains. Our results provide a theoretical basis for research into PRV prevalence strains in southwest China, which is conducive to the prevention and treatment of pseudorabies.

## Electronic supplementary material

Below is the link to the electronic supplementary material.

Supplementary file1 (DOCX 2213 kb)

## Data Availability

All data generated or analyzed during this study are included in this published article [and its supplementary information files].

## Abbreviations

PRV: Pseudorabies virus
PRRSV: Porcine reproductive and respiratory syndrome virus
miRNAs: MicroRNAs
Nt: Nucleotide
DE: Differentially expressed
rRNA: Ribosomal RNA
tRNA: Transfer RNA
sRNA: Small RNA
snRNA: Small nuclear RNA
GO: Gene Ontology
STRING: Search Tool for the Retrieval of Interacting Genes/Proteins
CD3G: T-cell surface glycoprotein CD3 gamma chain
CXCL: C-X-C Motif Chemokine Ligand
PK-15: Porcine kidney epithelial cells
Th: T helper
IL: Interleukin
