# Supplementary material for: Effects of gE/gI deletions on the miRNA expression of PRV-infected PK-15 cells
Source: Virus Genes. 2020 May 8;56(4):461–71. doi: 10.1007/s11262-020-01760-6 (PMC7329775; doi:10.1007/s11262-020-01760-6)
Supplement: Supplementary file 1 — Supplementary file1 (DOCX 2213 kb) [file 11262_2020_1760_MOESM1_ESM.docx]

**Effects of gE/gI deletions on the miRNA expression of**

**PRV-infected PK-15 cells**

Xiao Liu ^1＃*^, Email: [Drliuxiao@126.com](mailto:Drliuxiao@126.com)

Yuancheng Zhou ^1＃^, Email: [abtczyc@163.com](mailto:abtczyc@163.com)

Yuan Luo ^1^, Email: 1844901494@qq.com

Yanxi Chen ^1^, Email: 791995512@qq.com

^1^Southwest University, College of Animal Science and technology, 400715, Chongqing, China

^2^ Livestock and Poultry Biological Products Key Laboratory of Sichuan Province, Huashen Veterinary Biological Products Co., LTD, Chengdu 610200, China

^*^Corresponding author at:

^1^Southwest University, College of Animal Science and technology, 2#Tiansheng Road, Beibei District, Chongqing 400715, China. Tel.: +86 023 68251196; fax: +86 023 68251196; E-mail address: Drliuxiao@126.com

^#^ These authors contributed equally to this work.

**Supplementary Materials**

**Supplementary Figure S1. Length distribution of total sRNAs and mapped sRNAs in Fa wild strain infected PK-15 cells and PRV FaΔgE/gI strain infected PK-15 cells.**

**Supplementary Figure S2. Top 10 miRNA reads in high-throughput sequencing profiles.**

**Supplementary Figure S3. Target prediction of PRV encoded-miRNAs.**

**Supplementary Figure S4. GO functional enrichment annotations for the target genes of DE miRNAs.**

**Table S1. Expression profiles of miRNAs.**

**Table S2. Fold-change levels of DE miRNAs (top 10)**

**Table S3. Primers used for stem-loop qRT-PCR.**

**Table S4. Target prediction for DE miRNAs.**

**Table S5. DE miRNAs in vivo and vitro under viral infection**

**Supplementary Materials**

**Supplementary Figure S1. Length distribution of total sRNAs and mapped sRNAs in Fa wild strain infected PK-15 cells and PRV FaΔgE/gI strain infected PK-15 cells.**

(A) Adapter-trimmed read counts against the read lengths in non-infected PK-15 cells.

(B) Adapter-trimmed read counts against the read lengths in Fa wild strain infected PK-15 cells.

(C) Adapter-trimmed read counts against the read lengths in PRV Fa ΔgE/gI strain infected PK-15 cells.

**A**


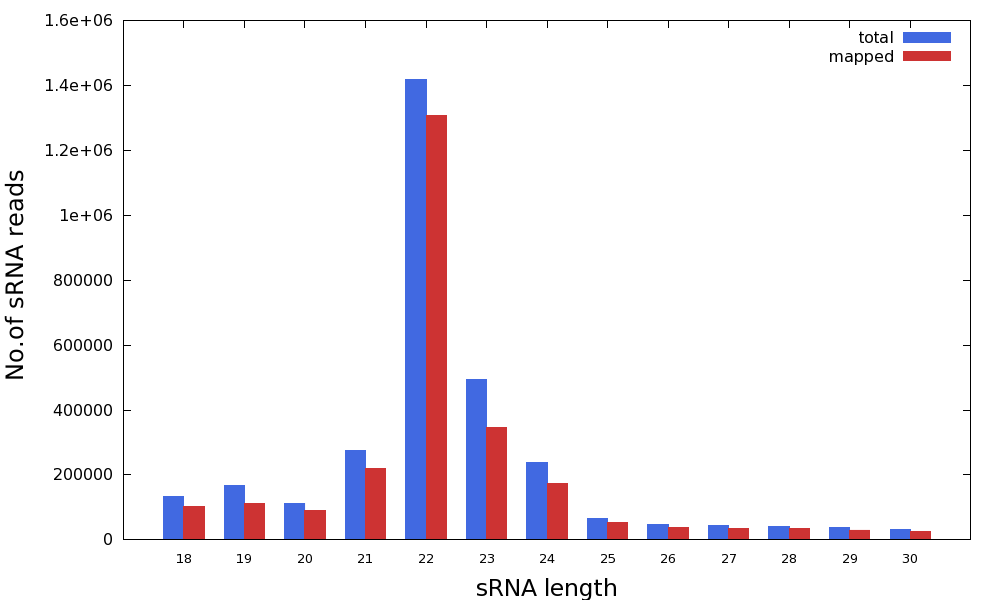


**B**


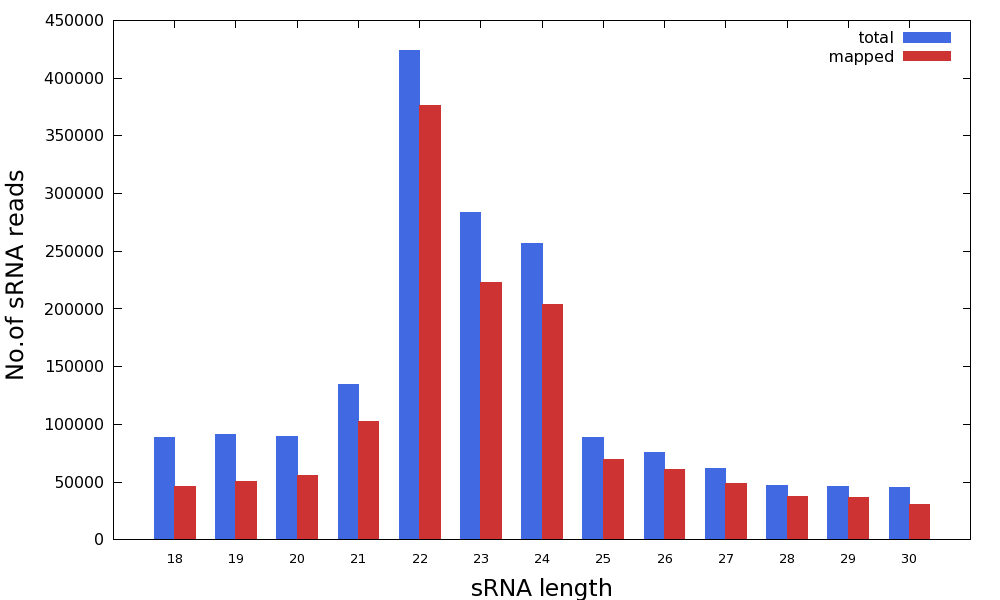


**C**


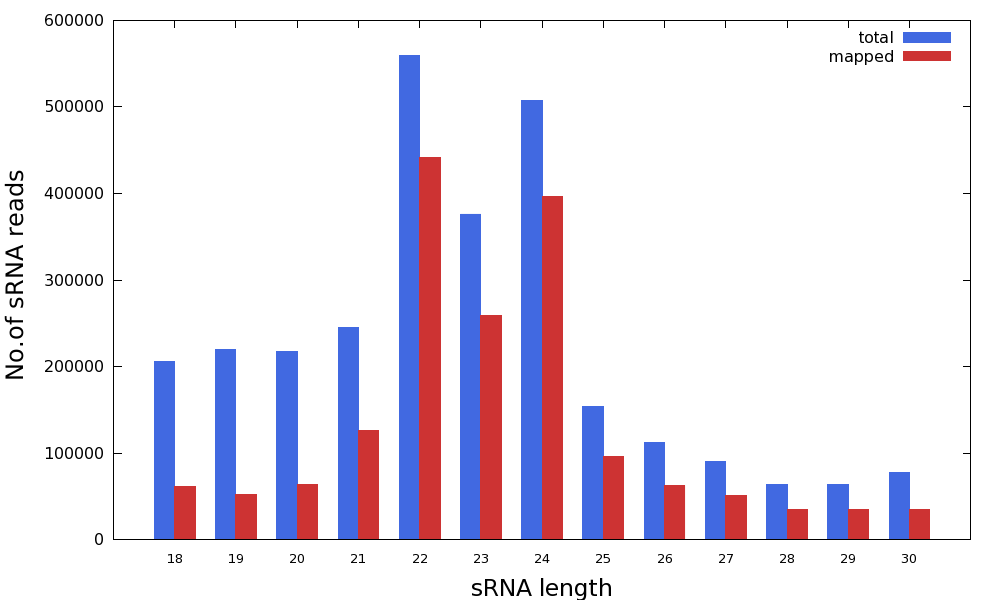


**Supplementary Figure S2. Top 10 miRNA reads in high-throughput sequencing profiles.**

(A) Top 10 miRNA reads of non-infected PK-15 cells

(B) Top 10 miRNA reads of Fa wild strain infected PK-15 cells

(C) Top 10 miRNA reads of PRV FaΔgE/gI strain infected PK-15 cells

**A**


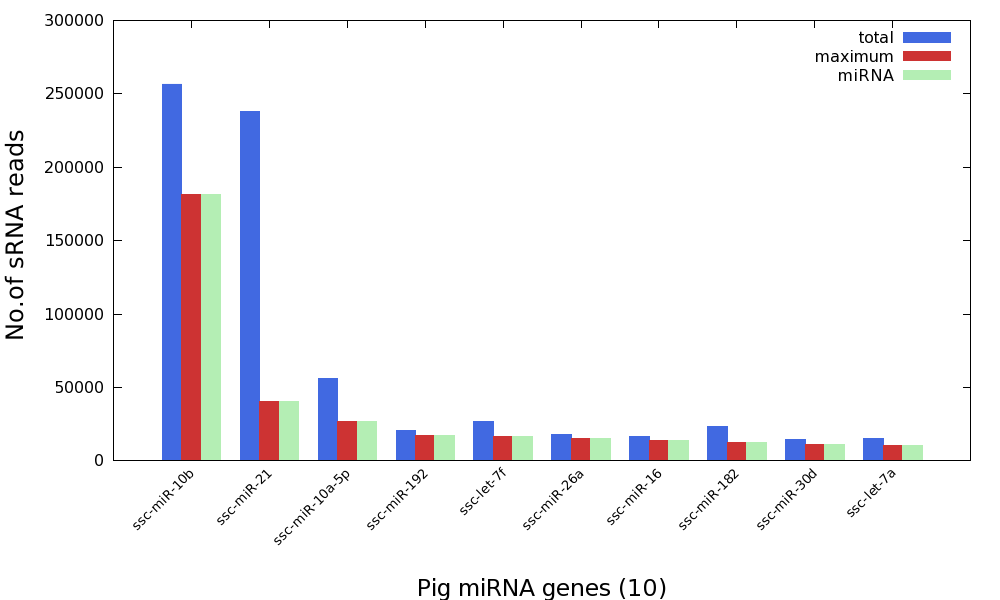


**B**

**
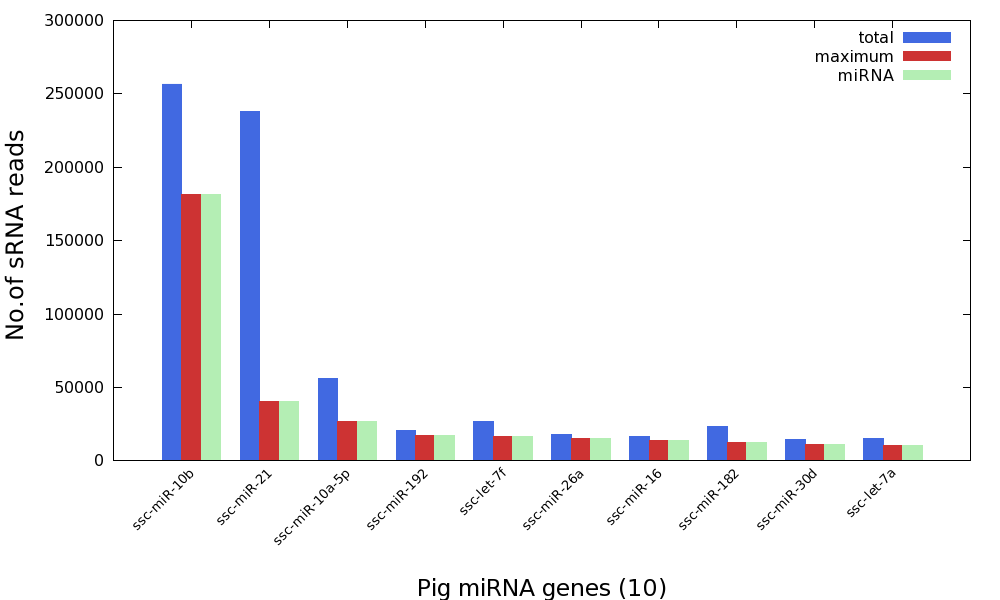
**

**C**


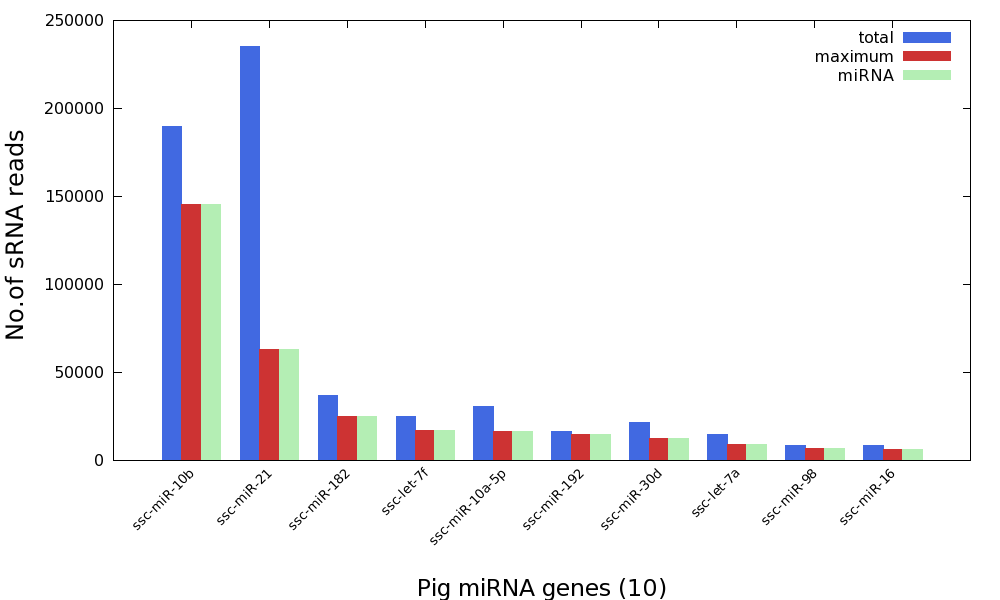


**Supplementary Figure S3. Target prediction of PRV encoded-miRNAs.**


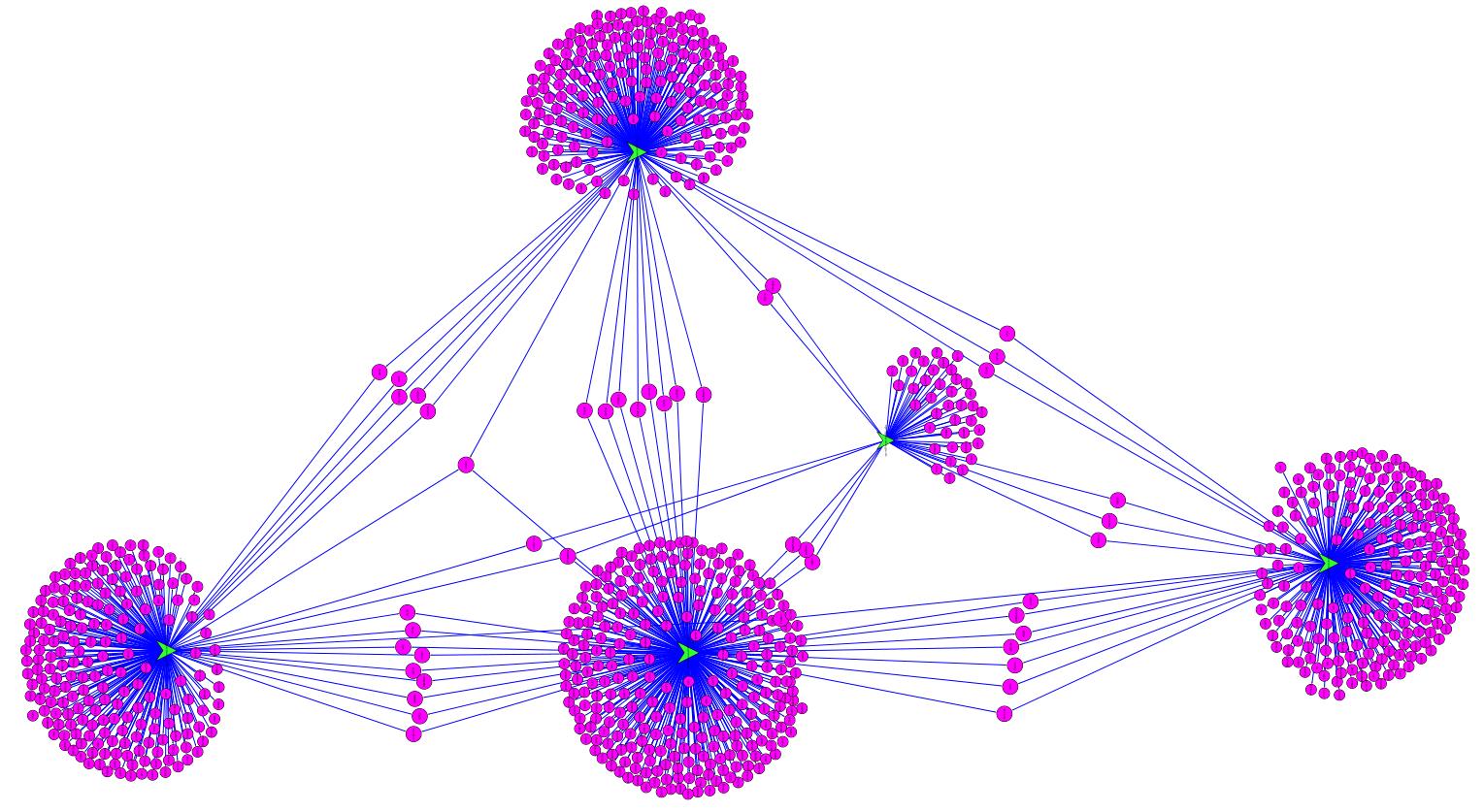


**Supplementary Figure S4. GO functional enrichment annotations for the target genes of DE miRNAs.**

GO terms of up-regulated (PRV Fa wild strain infected / non-infected) miRNAs target genes in PRV Fa wild strain infected PK-15 cells (Biological Process)


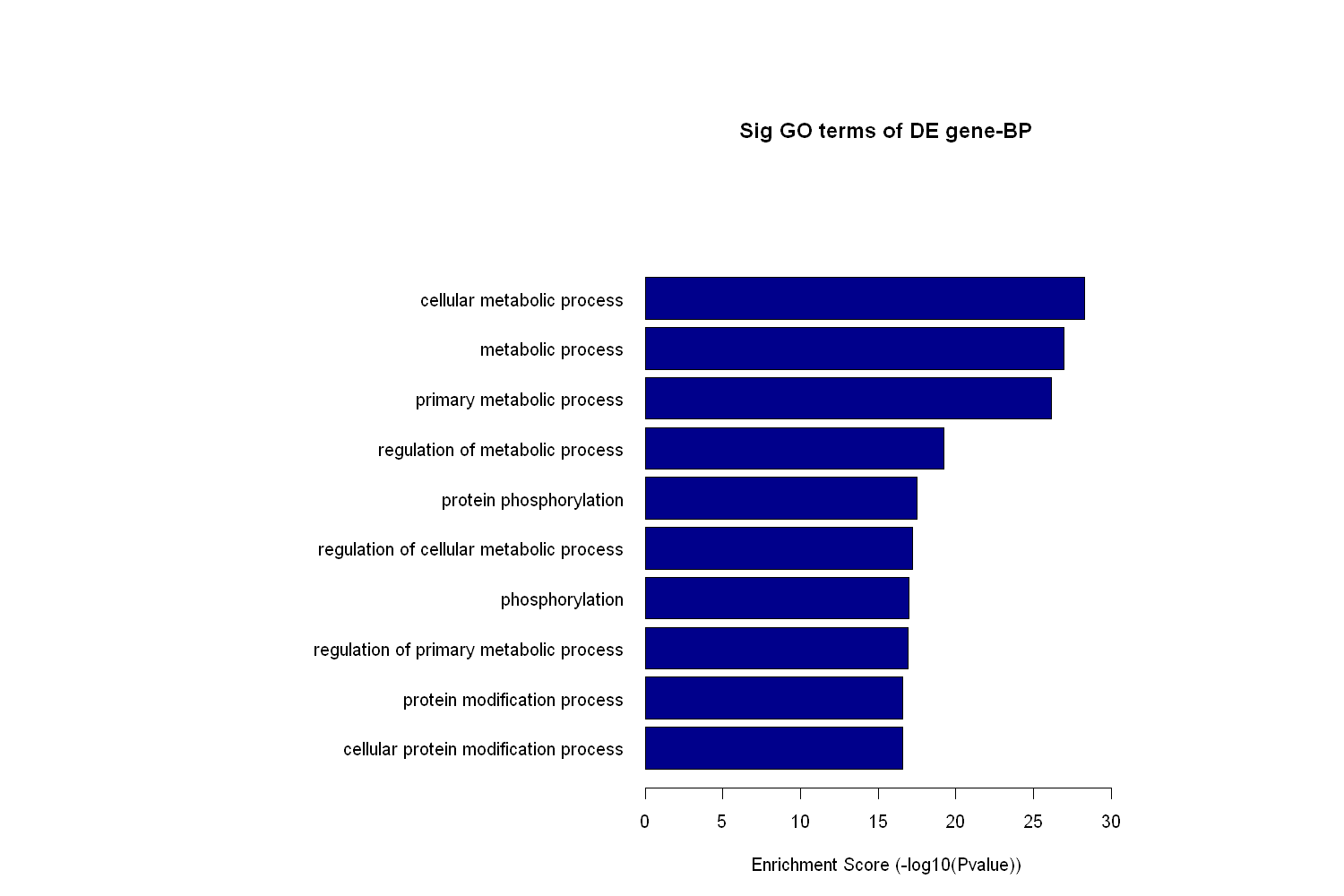


GO terms of up-regulated (PRV Fa wild strain infected / non-infected) miRNAs target genes in PRV Fa wild strain infected PK-15 cells (Cellular Component)


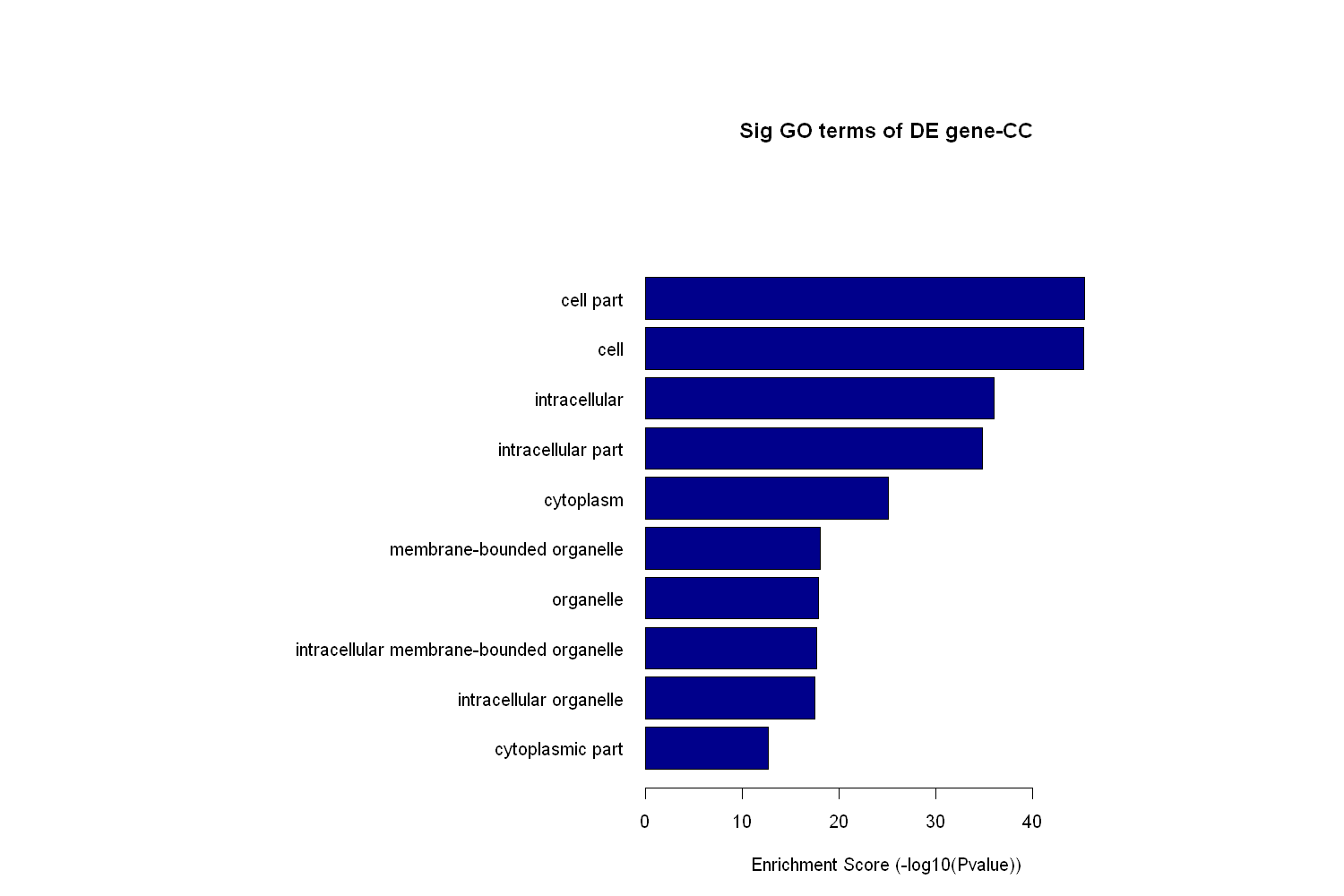


GO terms of up-regulated (PRV Fa wild strain infected / non-infected) miRNAs target genes in PRV Fa wild strain infected PK-15 cells (Molecular Function)


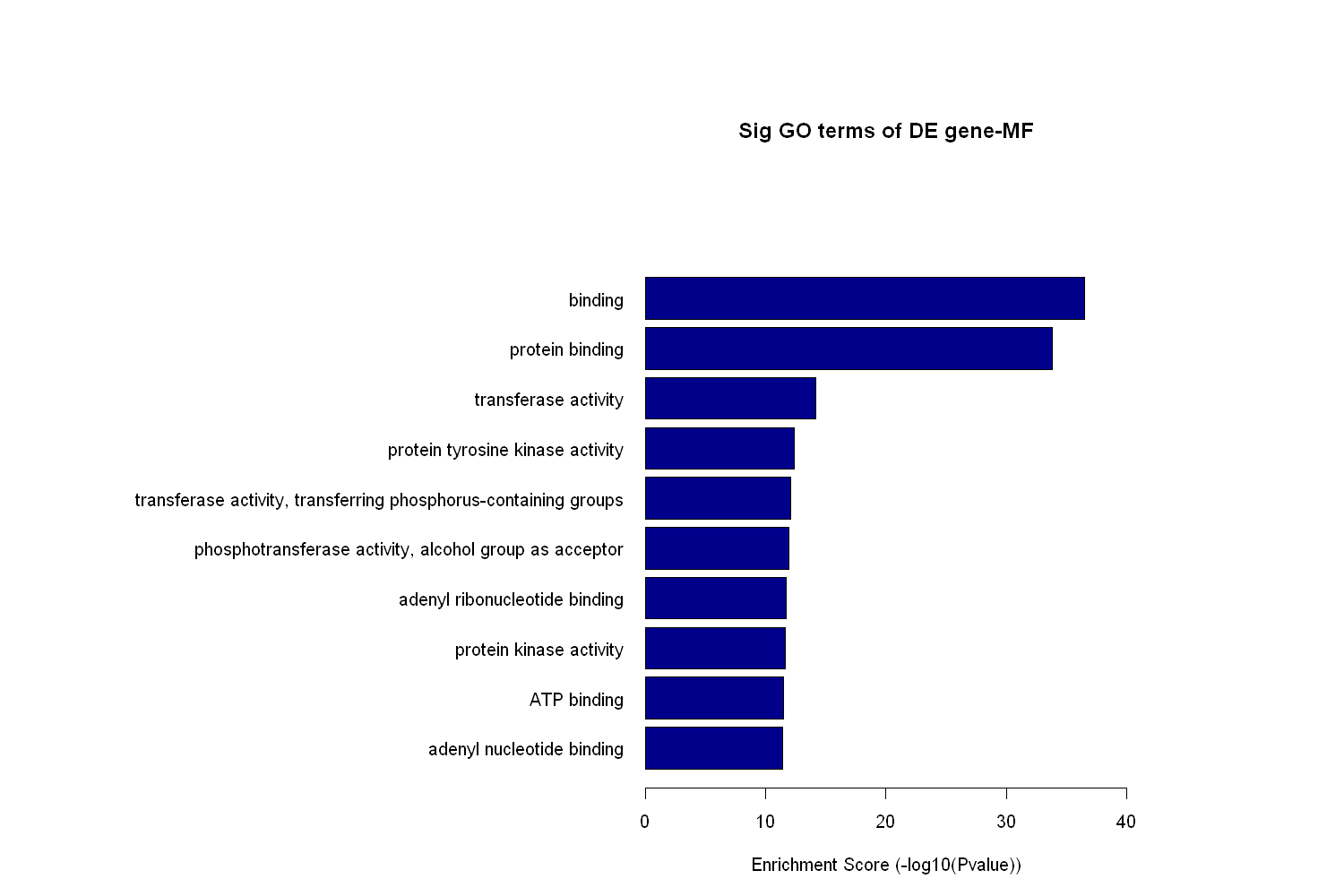


GO terms of down-regulated (PRV Fa wild strain infected / non-infected) miRNAs target genes in PRV Fa wild strain infected PK-15 cells (Biological Process)


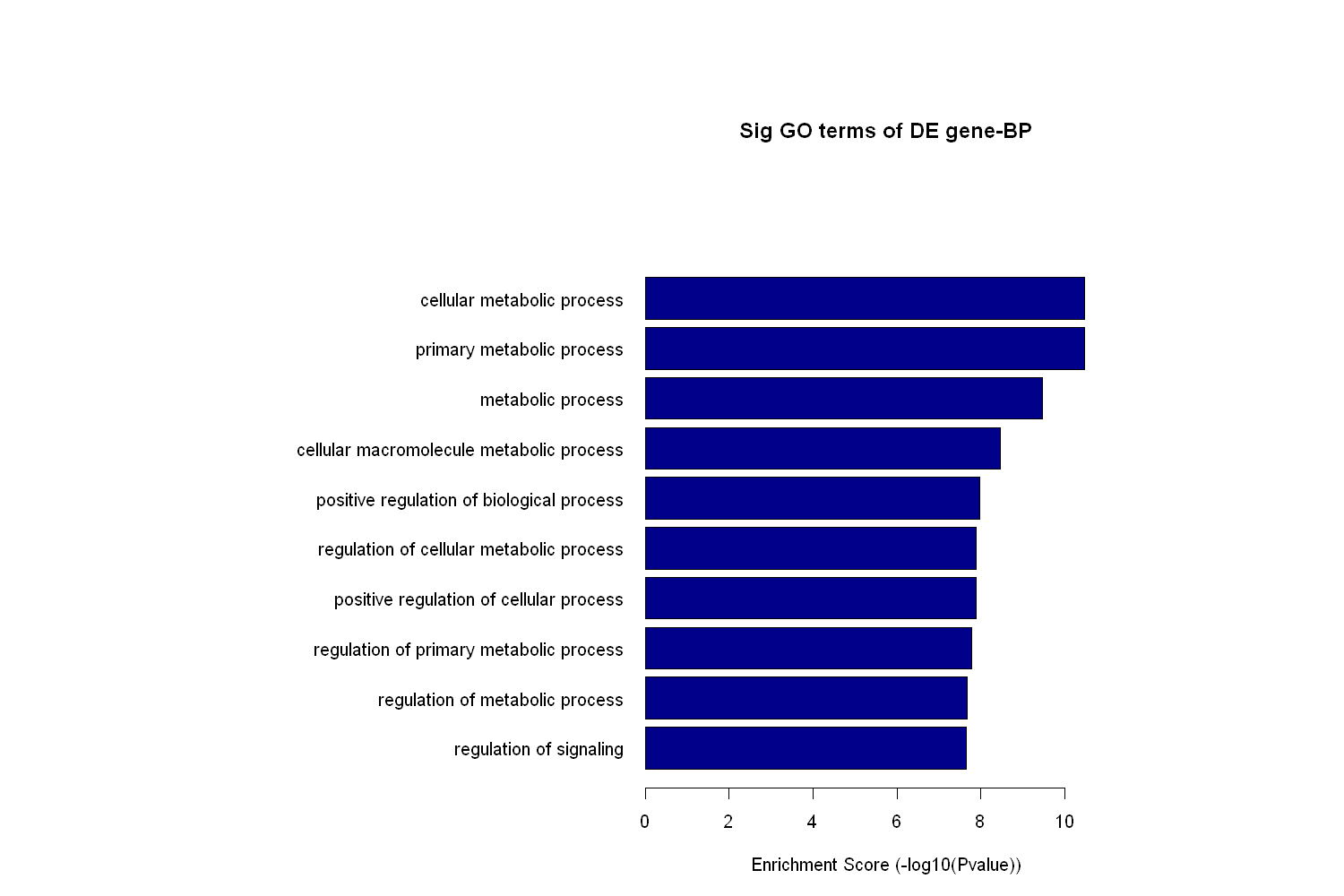


GO terms of down-regulated (PRV Fa wild strain infected / non-infected) miRNAs target genes in PRV Fa wild strain infected PK-15 cells (Cellular Component)


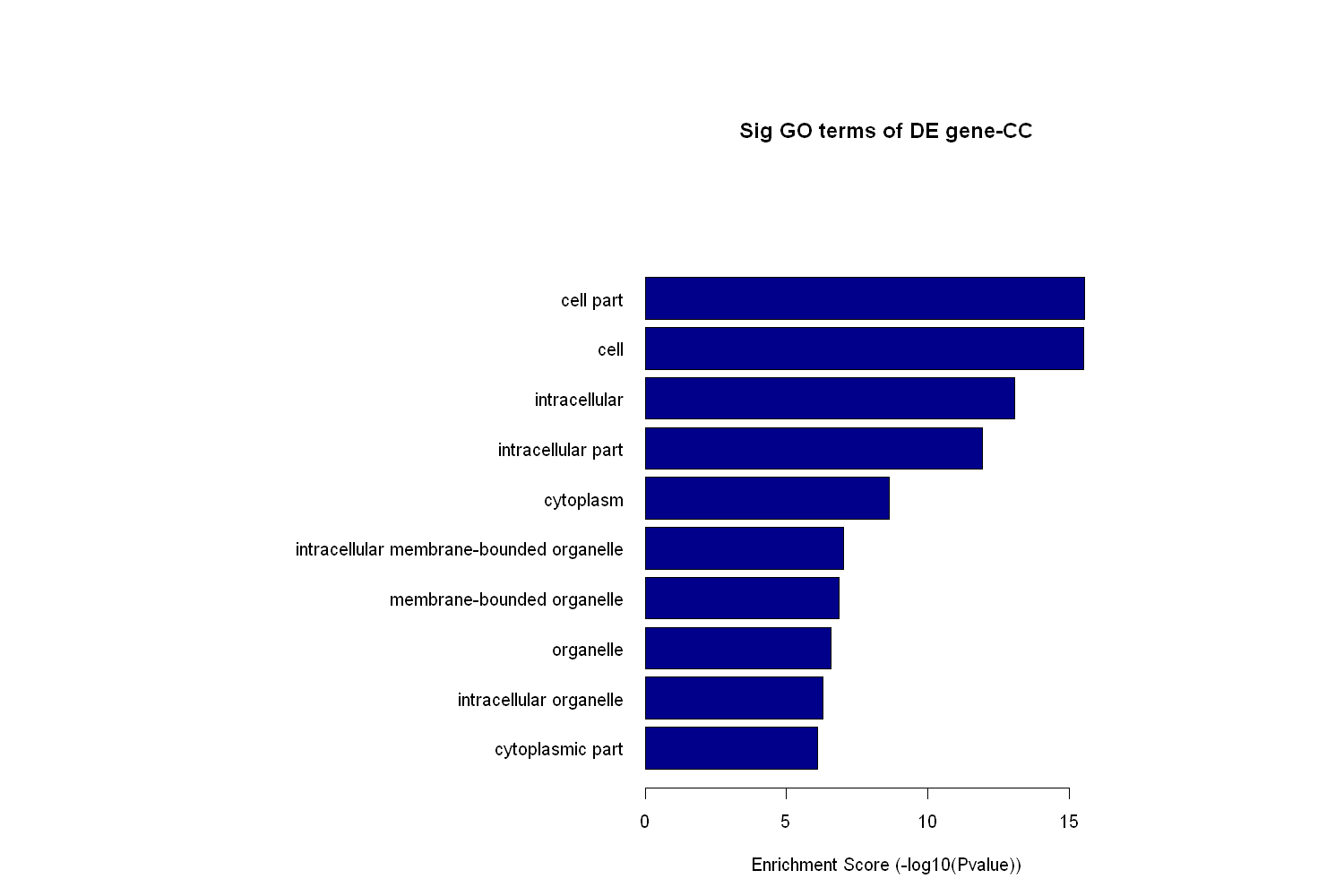


GO terms of down-regulated (PRV Fa wild strain infected / non-infected) miRNAs target genes in PRV Fa wild strain infected PK-15 cells (Molecular Function)


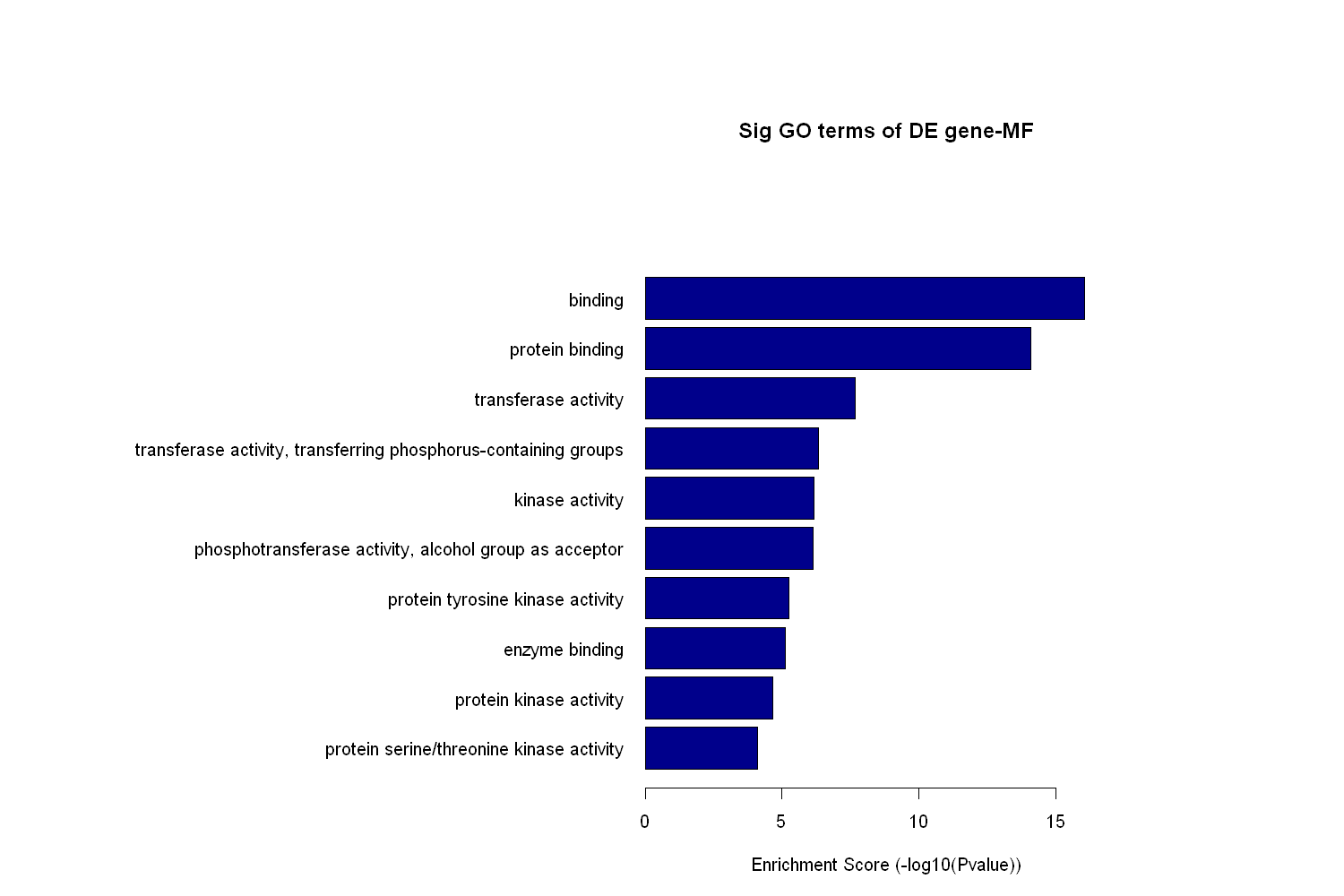


GO terms of up-regulated (PRV FaΔgE/gI strain infected / non-infected) miRNAs target genes in PRV FaΔgE/gI strain infected PK-15 cells (Biological Process)


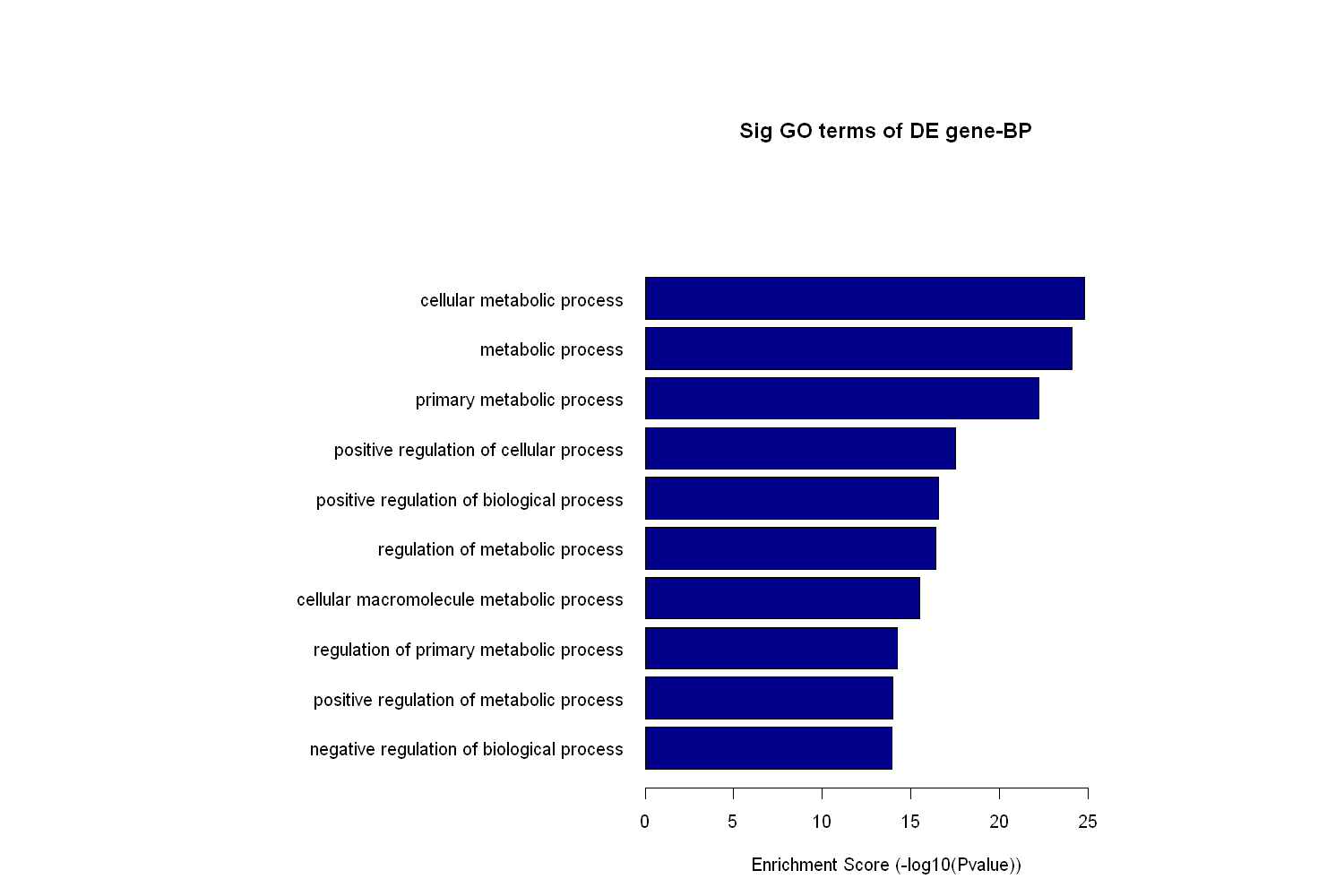


GO terms of up-regulated (PRV FaΔgE/gI strain infected / non-infected) miRNAs target genes in PRV FaΔgE/gI strain infected PK-15 cells (Cellular Component)


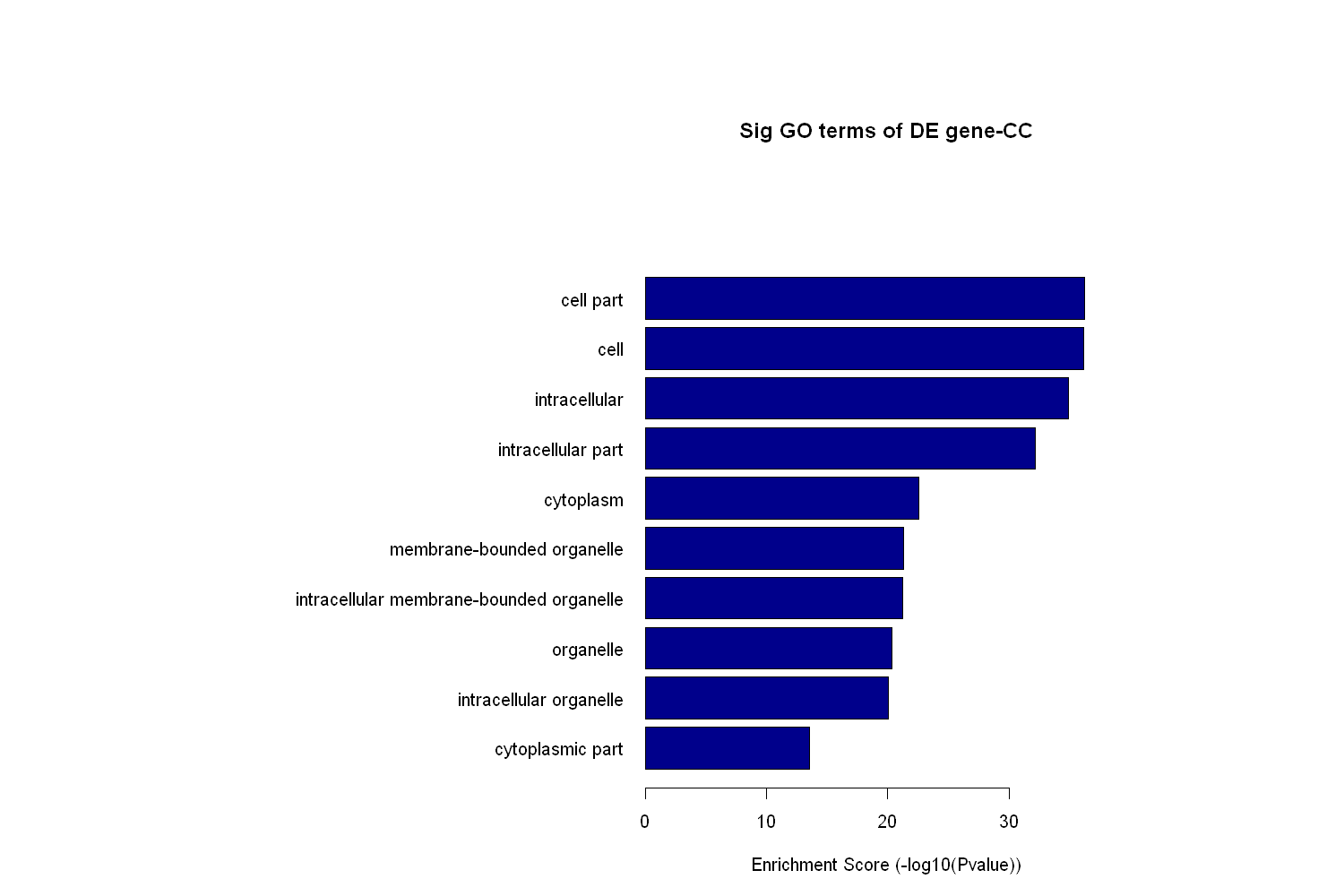


GO terms of up-regulated (PRV FaΔgE/gI strain infected / non-infected) miRNAs target genes in PRV FaΔgE/gI strain infected PK-15 cells (Molecular Function)


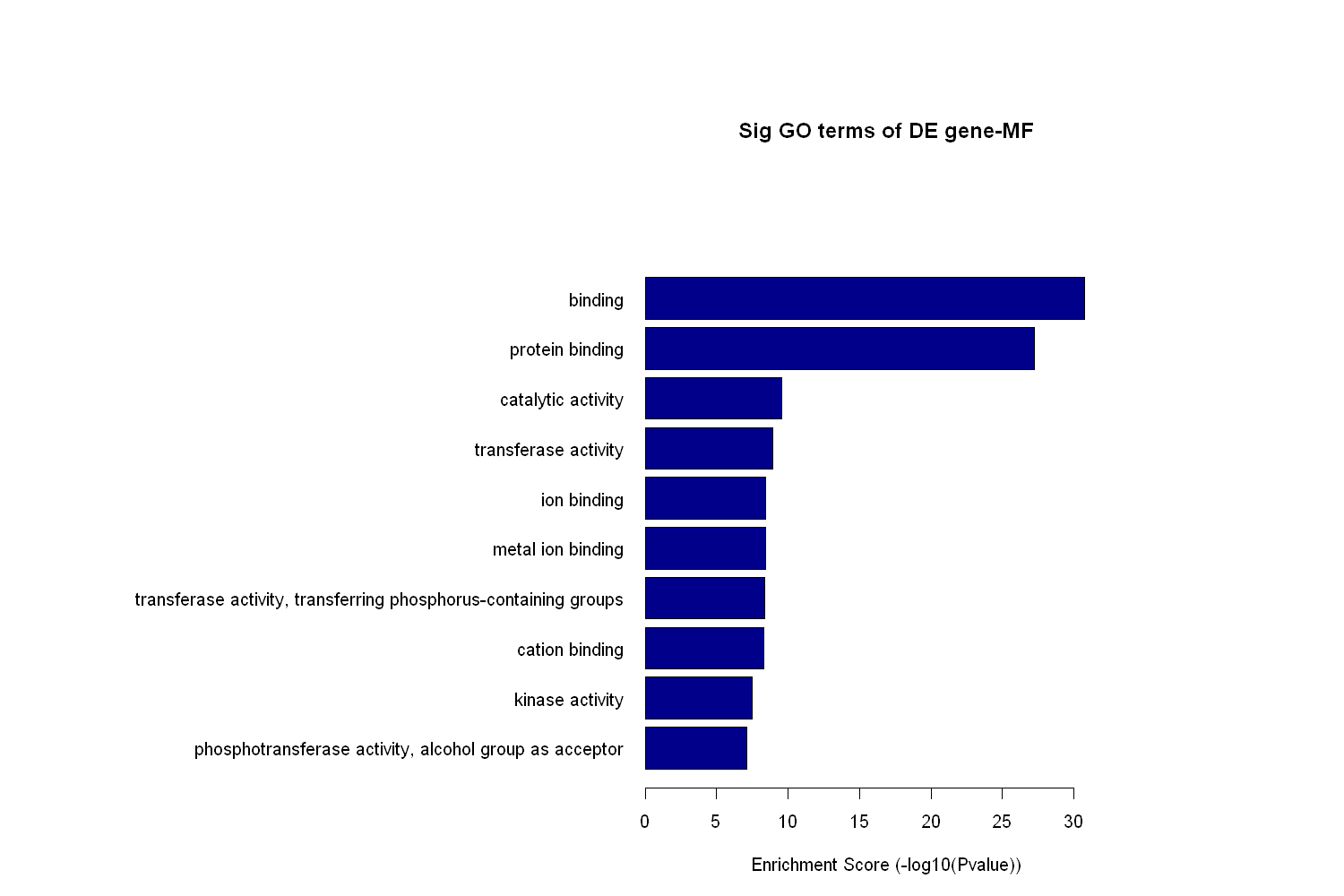


GO terms of down-regulated (PRV FaΔgE/gI strain infected / non-infected) miRNAs target genes in PRV FaΔgE/gI strain infected PK-15 cells (Biological Process)


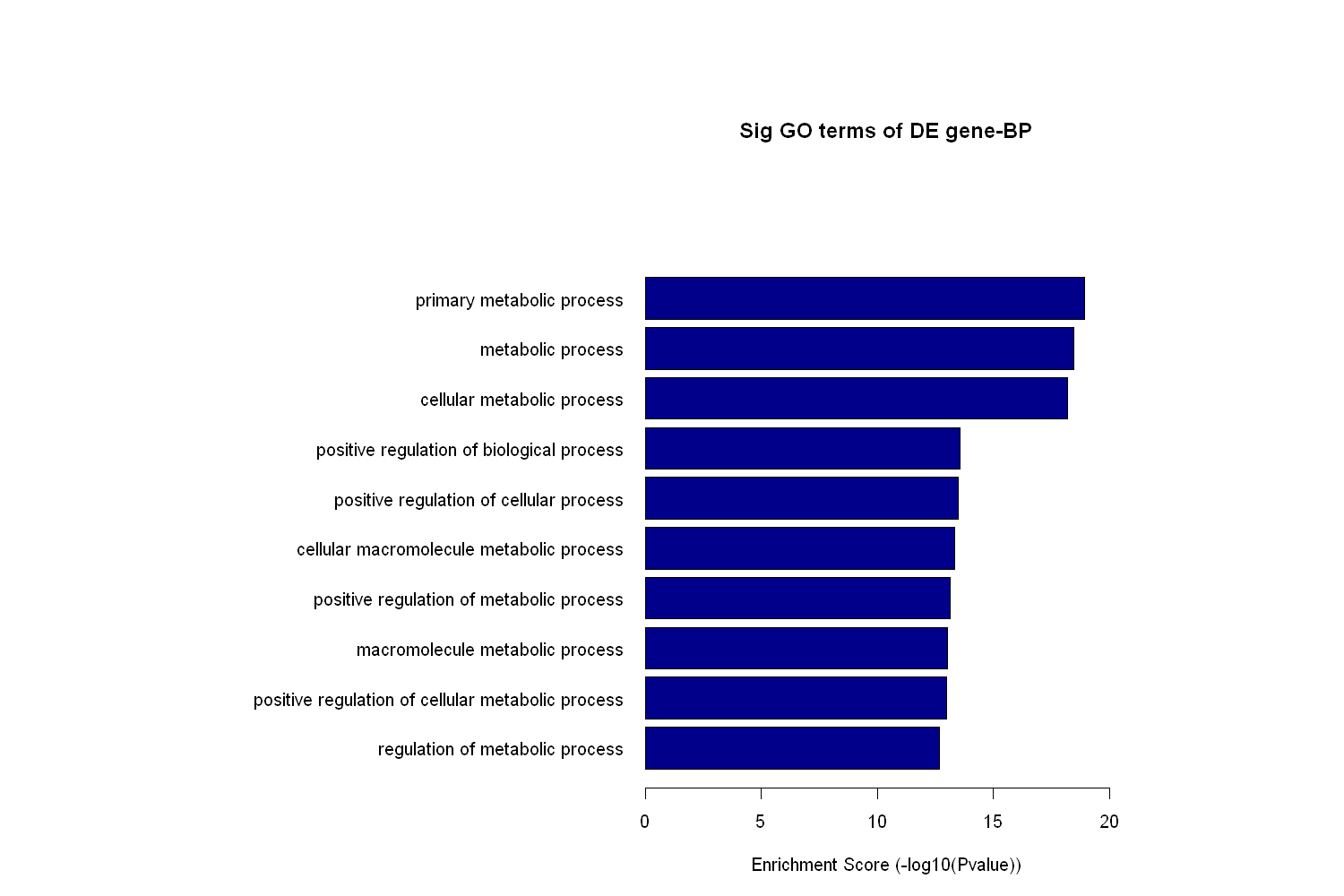


GO terms of down-regulated (PRV FaΔgE/gI strain infected / non-infected) miRNAs target genes in PRV FaΔgE/gI strain infected PK-15 cells (Cellular Component)


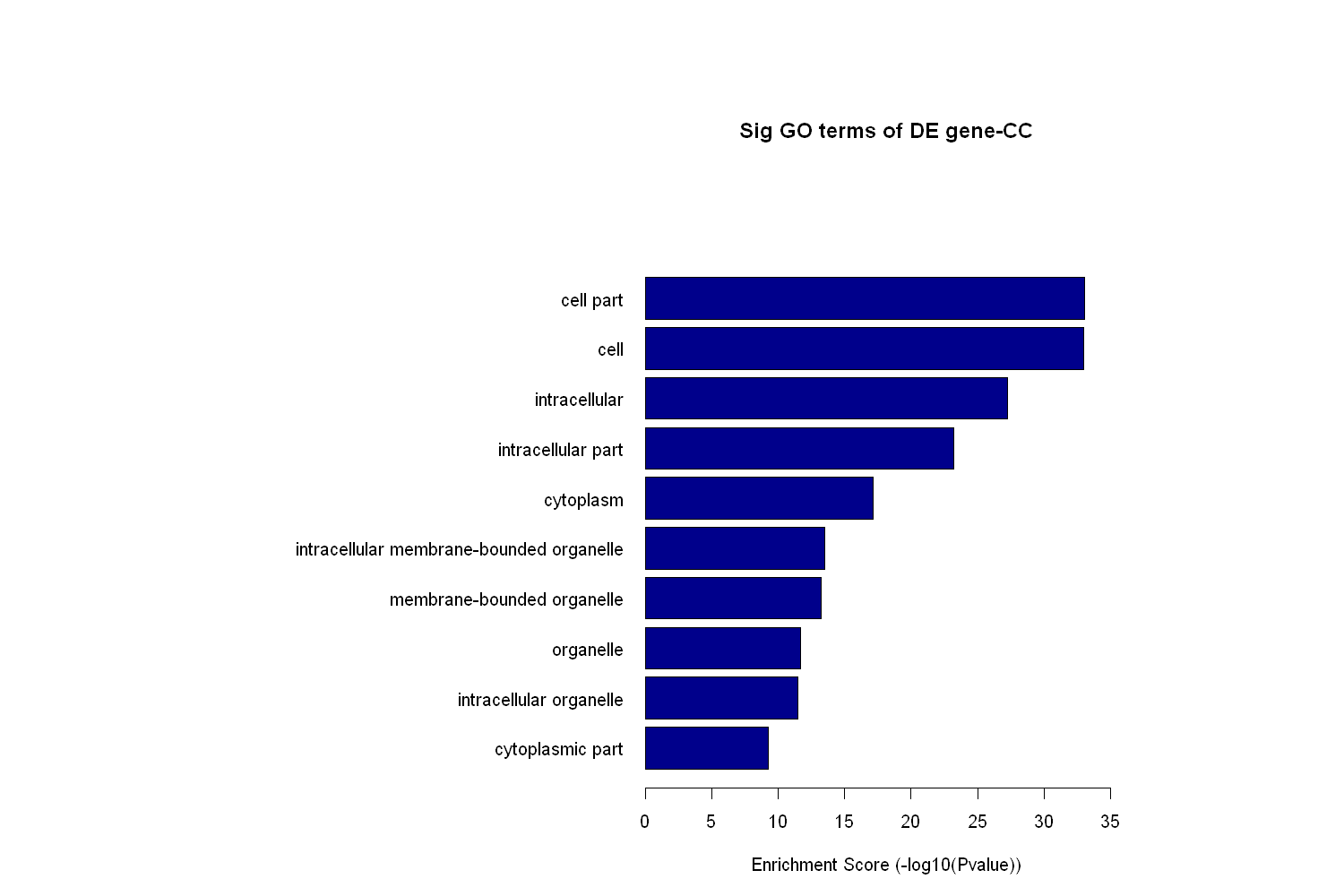


GO terms of down-regulated (PRV FaΔgE/gI strain infected / non-infected) miRNAs target genes in PRV FaΔgE/gI strain infected PK-15 cells (Molecular Function)


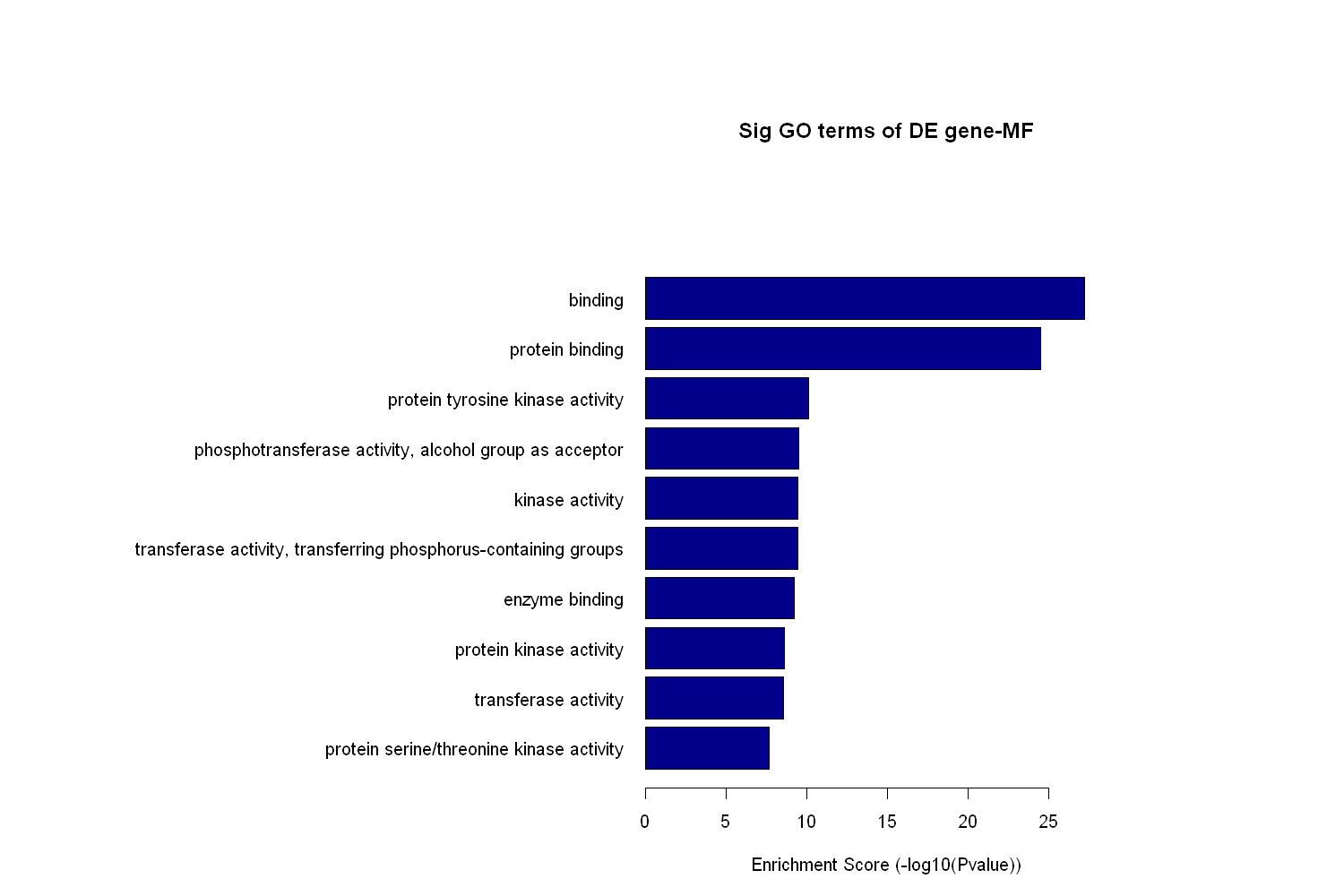


GO terms of up-regulated (PRV FaΔgE/gI strain infected / Fa wild strain infected)

miRNAs target genes (Biological Process)


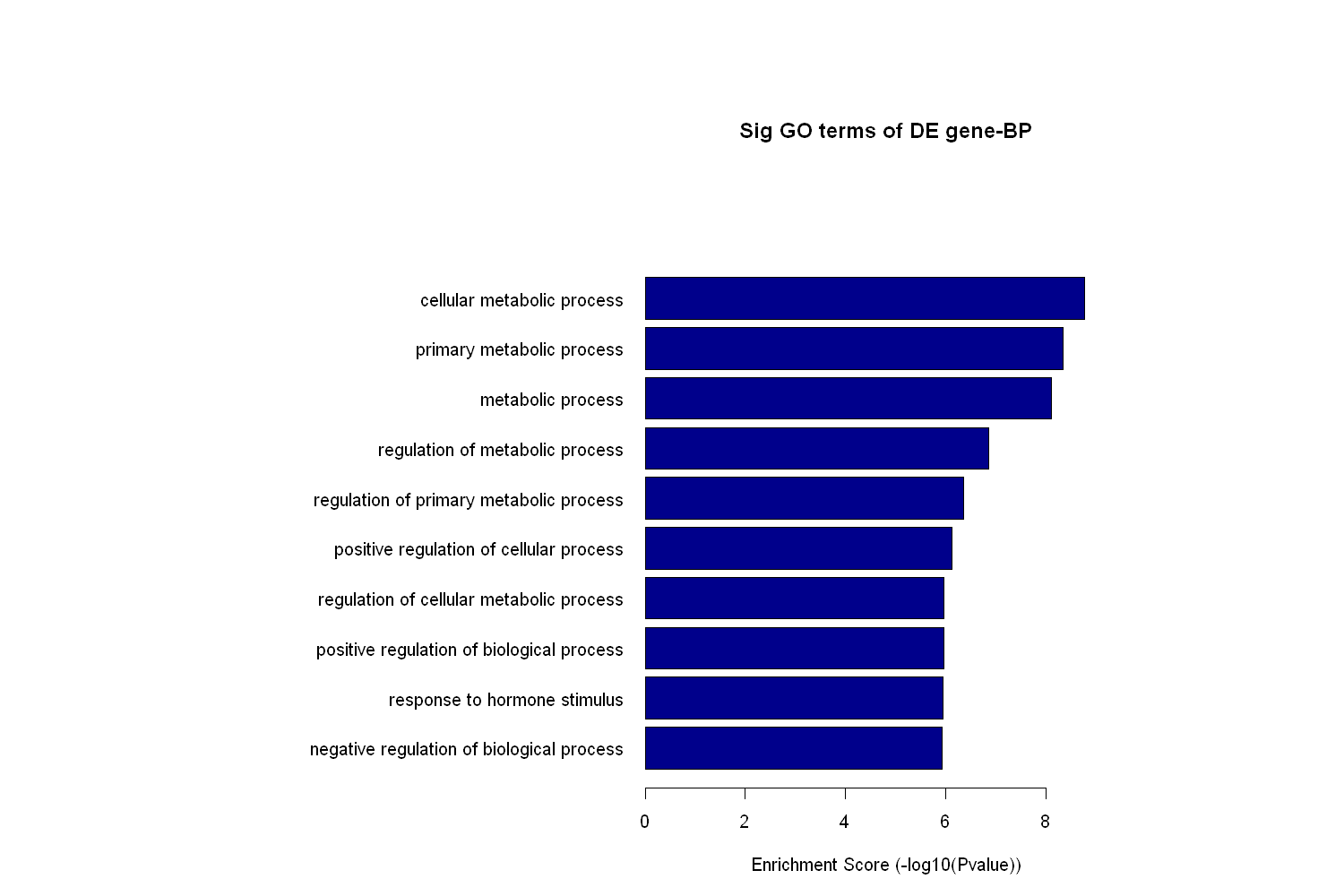


GO terms of up-regulated (PRV FaΔgE/gI strain infected / Fa wild strain infected)

miRNAs target genes (Cellular Component)


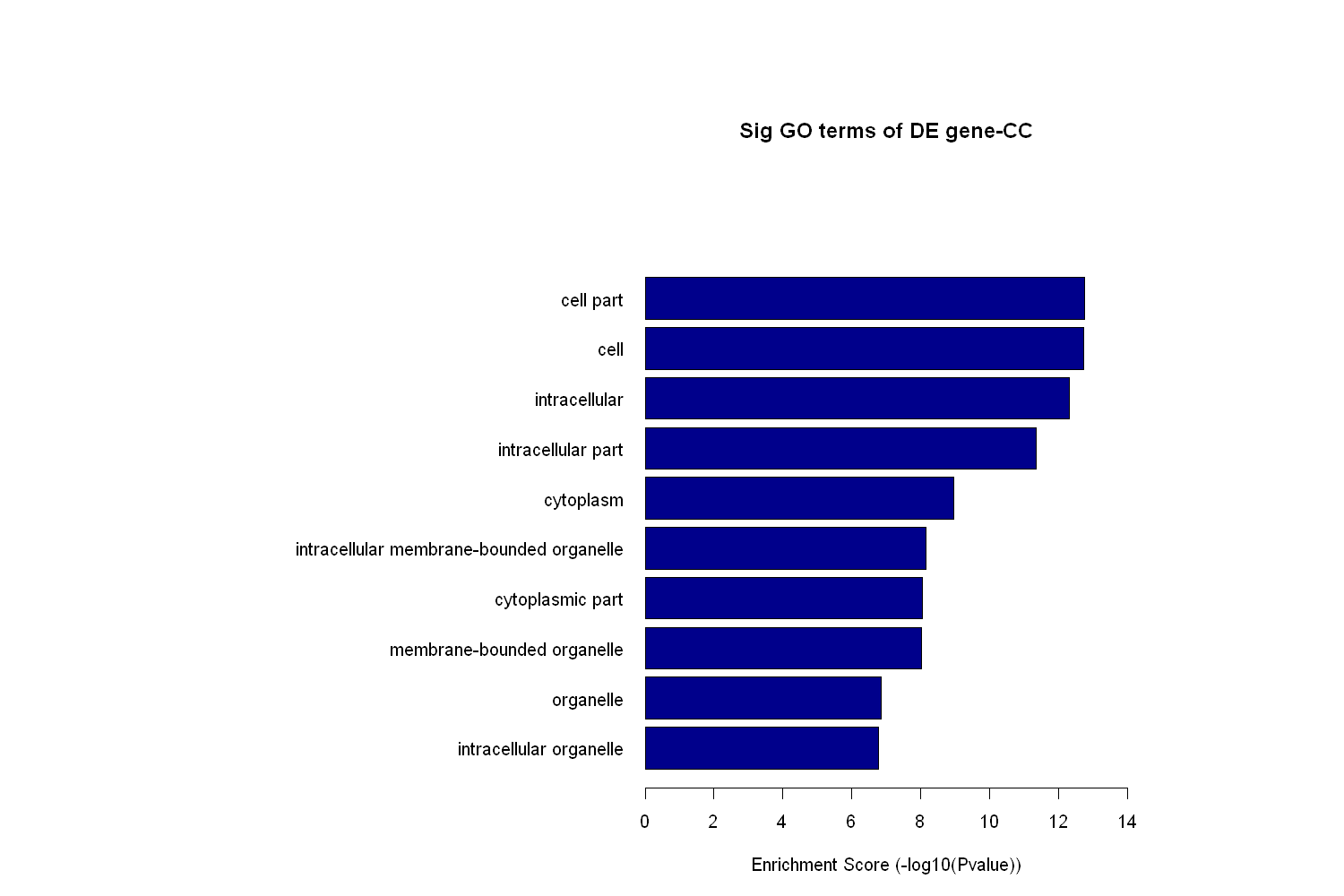


GO terms of up-regulated (PRV FaΔgE/gI strain infected / Fa wild strain infected)

miRNAs target genes (Molecular Function)


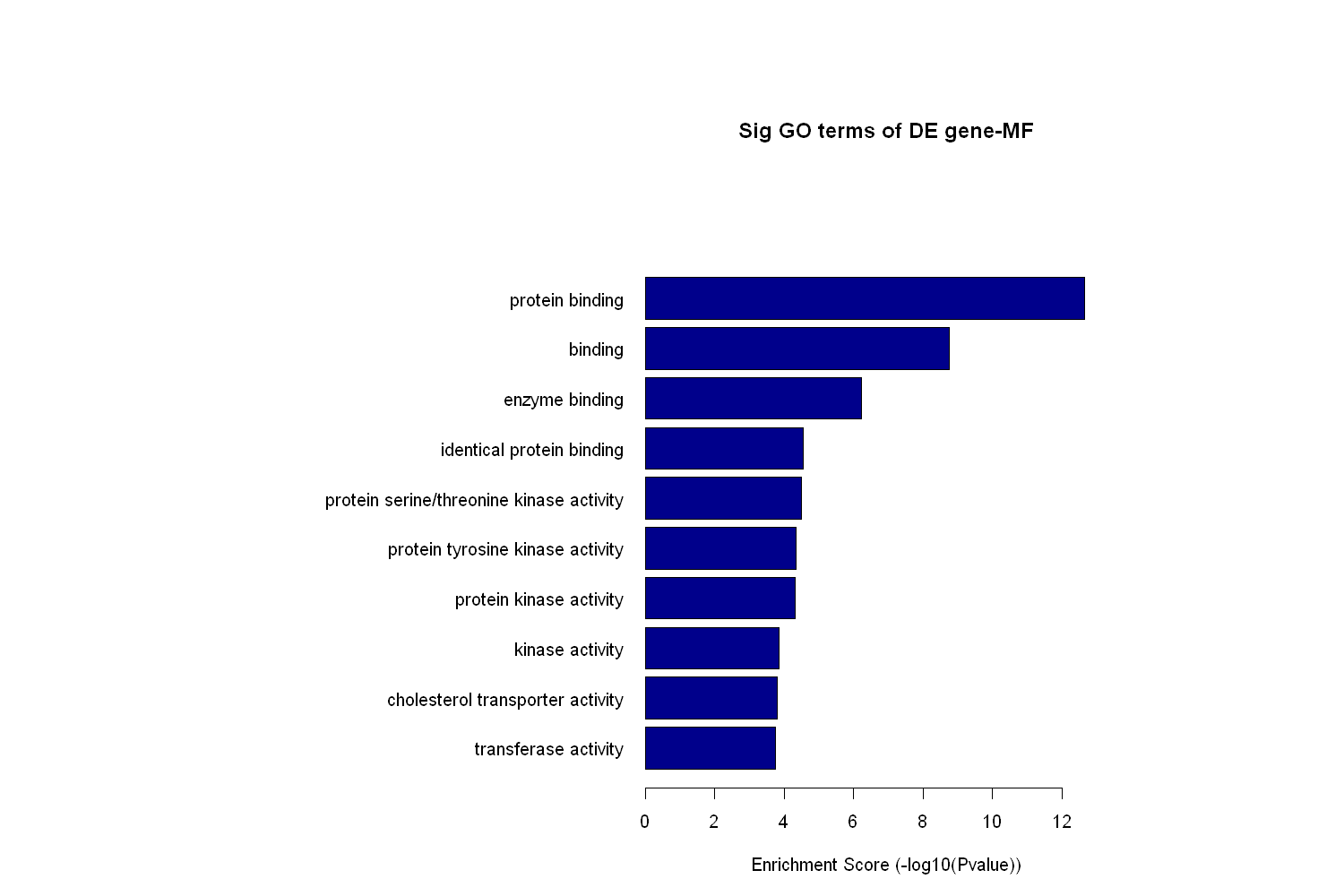


GO terms of down-regulated (PRV FaΔgE/gI strain infected / Fa wild strain infected)

miRNAs target genes (Biological Process)


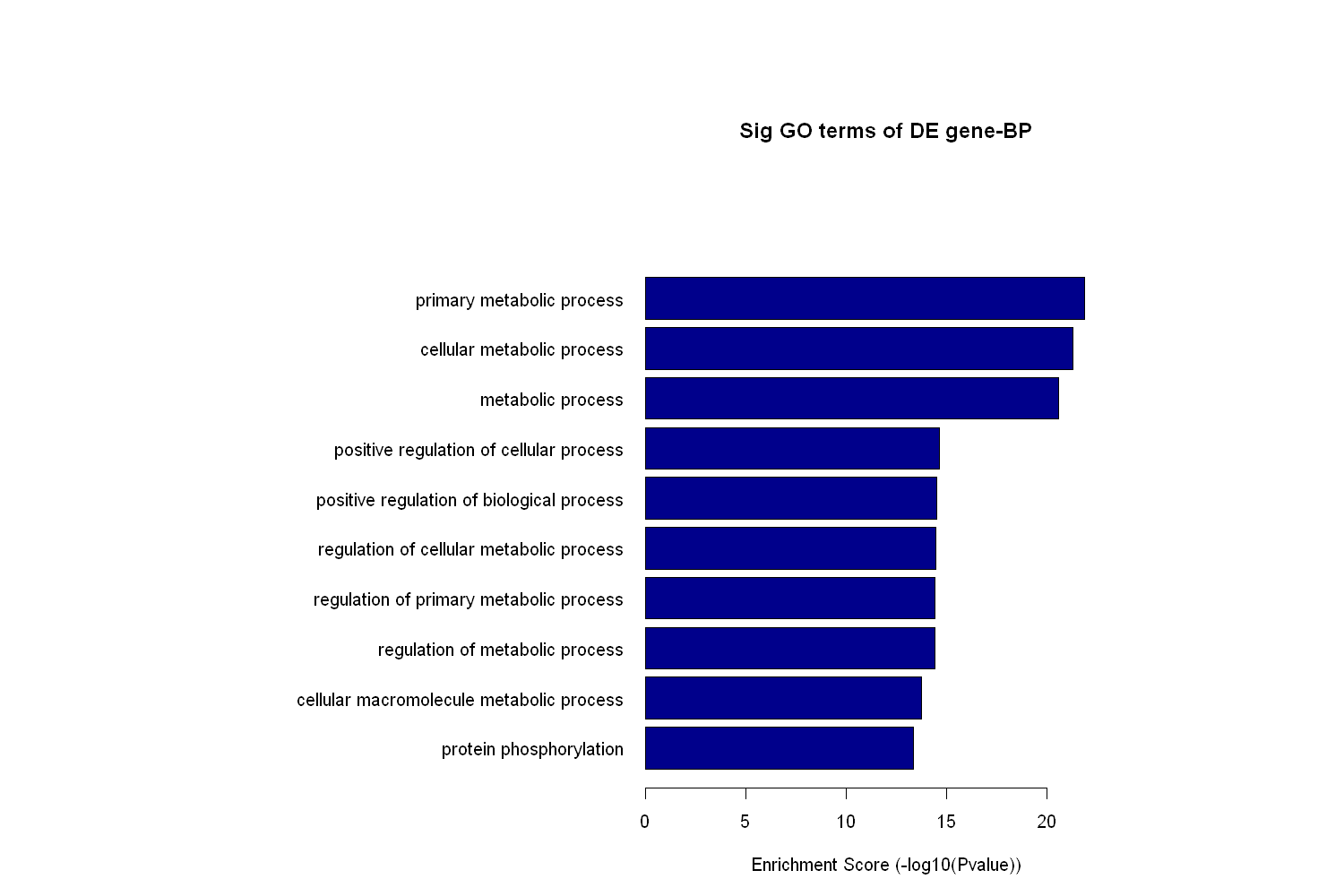


GO terms of down-regulated (PRV FaΔgE/gI strain infected / Fa wild strain infected)

miRNAs target genes (Cellular Component)
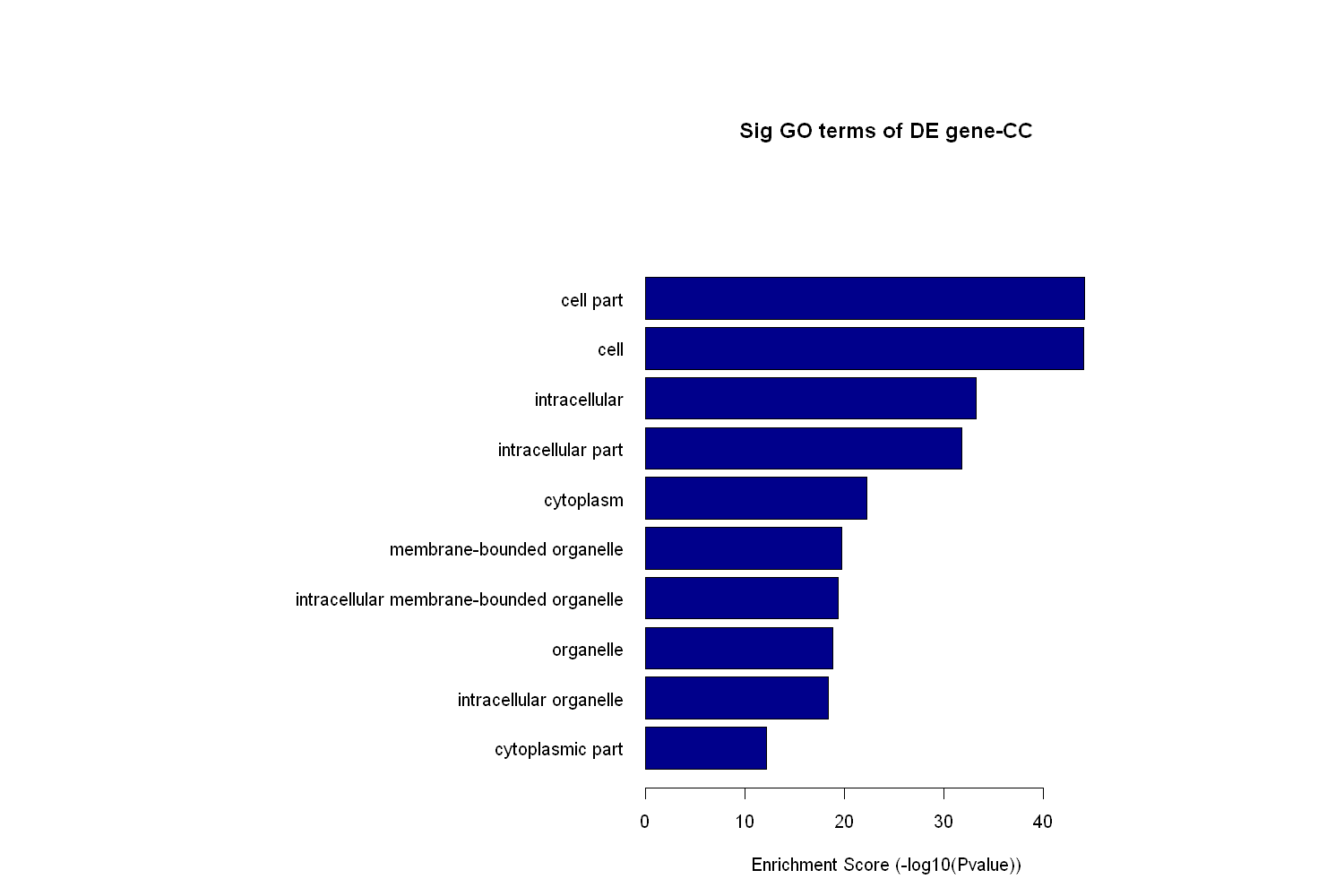


GO terms of down-regulated (PRV FaΔgE/gI strain infected / Fa wild strain infected)

miRNAs target genes (Molecular Function)


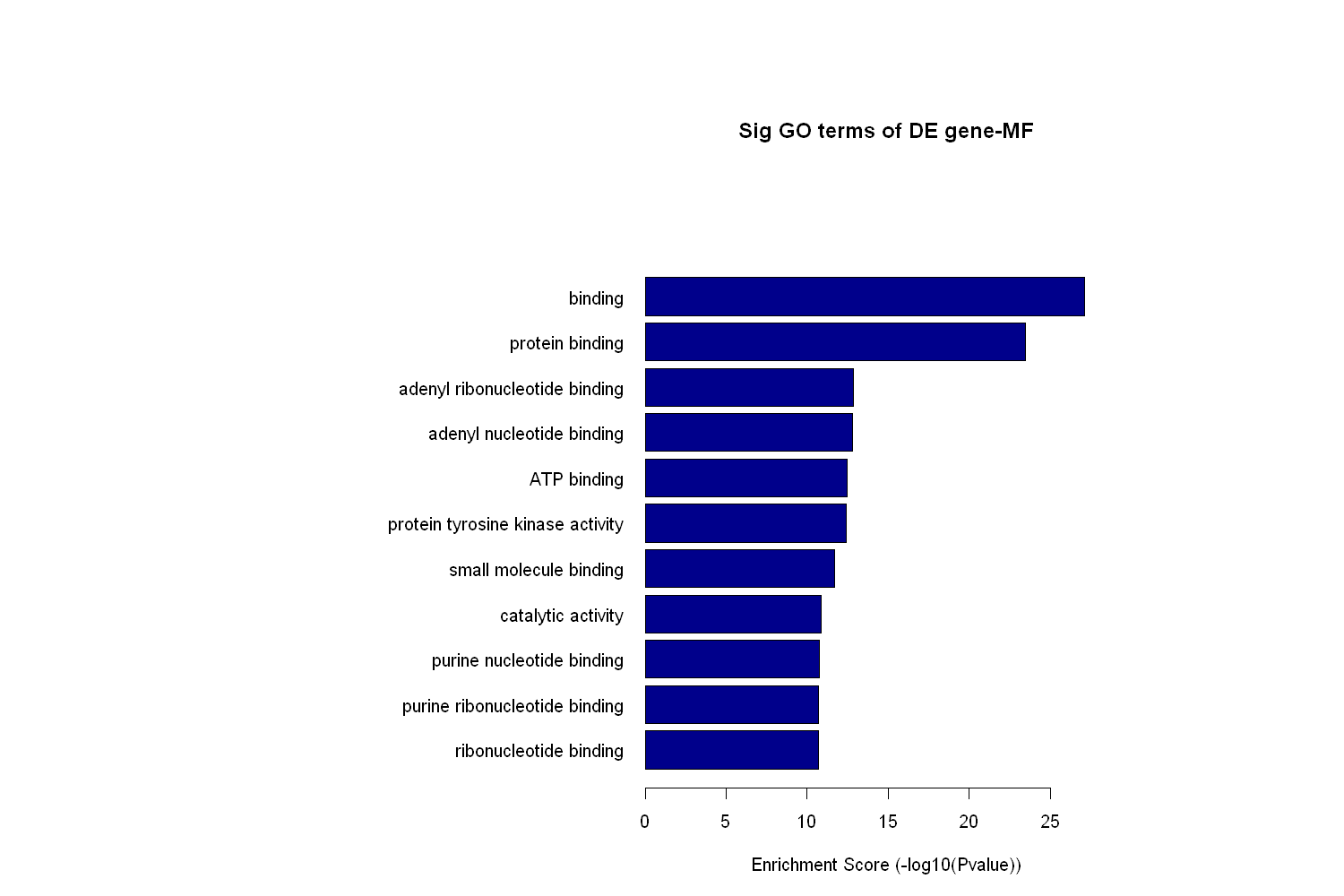


**Table S1. Expression profiles of miRNAs.**

| **Mature miRNA-ID** | **Pre-miRNA_ID** | **Pre-miRNA arm (5p or 3p)** | **Mature miRNA -SEQ** | **TagCount (PRV Fa wild strain infected PK-15 cells)** | **TagCount (Non-infected PK-15 cells)** | **Fold Change (PRV Fa wild strain infected vs Non-infected)** |
| --- | --- | --- | --- | --- | --- | --- |
| ssc-let-7a | ssc-let-7a-1 | 5p | TGAGGTAGTAGGTTGTATAGTT | 10064 | 9120 | 1.1033954 |
| ssc-let-7a | ssc-let-7a-2 | 5p | TGAGGTAGTAGGTTGTATAGTT | 10064 | 9120 | 1.1033954 |
| ssc-let-7c | ssc-let-7c | 5p | TGAGGTAGTAGGTTGTATGGTT | 1098 | 1637 | 0.672738312 |
| ssc-let-7d-5p | ssc-let-7d | 5p | AGAGGTAGTAGGTTGCATAGTT | 271 | 1773 | 0.157599551 |
| ssc-let-7d-3p | ssc-let-7d | 3p | CTATACGACCTGCTGCCTTTCT |  | 8 | 0.555555556 |
| ssc-let-7e | ssc-let-7e | 5p | TGAGGTAGGAGGTTGTATAGTT | 455 | 522 | 0.87406015 |
| ssc-let-7f | ssc-let-7f-1 | 5p | TGAGGTAGTAGATTGTATAGTT | 16178 | 66172 | 0.244598229 |
| ssc-let-7f | ssc-let-7f-2 | 5p | TGAGGTAGTAGATTGTATAGTT | 16178 | 66172 | 0.244598229 |
| ssc-let-7g | ssc-let-7g | 5p | TGAGGTAGTAGTTTGTACAGTT | 5165 | 3374 | 1.529255319 |
| ssc-let-7i | ssc-let-7i | 5p | TGAGGTAGTAGTTTGTGCT | 6862 | 3203 | 2.13881108 |
| ssc-miR-1 | ssc-mir-1 | 3p | TGGAATGTAAAGAAGTATGTA | 112 | 61 | 1.718309859 |
| ssc-miR-100 | ssc-mir-100 | 5p | AACCCGTAGATCCGAACTTGTG | 2446 | 7099 | 0.345477564 |
| ssc-miR-101 | ssc-mir-101-1 | 3p | TACAGTACTGTGATAACTGAA | 397 | 484 | 0.82388664 |
| ssc-miR-101 | ssc-mir-101-2 | 3p | TACAGTACTGTGATAACTGAA | 397 | 484 | 0.82388664 |
| ssc-miR-103 | ssc-mir-103-1 | 3p | AGCAGCATTGTACAGGGCTATGA | 886 | 697 | 1.267326733 |
| ssc-miR-103 | ssc-mir-103-2 | 3p | AGCAGCATTGTACAGGGCTATGA | 886 | 697 | 1.267326733 |
| ssc-miR-105-2 | ssc-mir-105-2 | 5p | TCAAATGCTCAGACTCCTTG |  |  |  |
| ssc-miR-106a | ssc-mir-106a | 5p | AAAAGTGCTTACAGTGCAGGTAGC | 2 |  | 1.2 |
| ssc-miR-107 | ssc-mir-107 | 3p | AGCAGCATTGTACAGGGCTATCA | 104 | 31 | 2.780487805 |
| ssc-miR-10a-5p | ssc-mir-10a | 5p | TACCCTGTAGATCCGAATTTGT | 25671 | 3471 | 7.377477736 |
| ssc-miR-10a-3p | ssc-mir-10a | 3p | CAAATTCGTATCTAGGGGAAT | 255 | 63 | 3.630136986 |
| ssc-miR-10b | ssc-mir-10b | 5p | TACCCTGTAGAACCGAATTTGT | 174810 | 1162 | 149.1638225 |
| ssc-miR-122 | ssc-mir-122 | 5p | TGGAGTGTGACAATGGTGTTTGT | 8 | 10 | 0.9 |
| ssc-miR-1224 | ssc-mir-1224 | 3p | CACCTCCTCTCTCCTCAGGT |  |  |  |
| ssc-miR-1249 | ssc-mir-1249-1 | 3p | ACGCCCTTCCCCCCCTTCTTCA | 2 | 1 | 1.090909091 |
| ssc-miR-1249 | ssc-mir-1249-2 | 3p | ACGCCCTTCCCCCCCTTCTTCA | 2 | 1 | 1.090909091 |
| ssc-miR-124a | ssc-mir-124a-1 | 3p | TAAGGCACGCGGTGAATGCCA |  | 5 | 0.666666667 |
| ssc-miR-124a | ssc-mir-124a-2 | 3p | TAAGGCACGCGGTGAATGCCA |  | 5 | 0.666666667 |
| ssc-miR-125a | ssc-mir-125a | 5p | TCCCTGAGACCCTTTAACCTGTG | 23 | 5 | 2.2 |
| ssc-miR-125b | ssc-mir-125b-1 | 5p | TCCCTGAGACCCTAACTTGTGA | 83 | 582 | 0.157094595 |
| ssc-miR-125b | ssc-mir-125b-2 | 5p | TCCCTGAGACCCTAACTTGTGA | 83 | 582 | 0.157094595 |
| ssc-miR-126-5p | ssc-mir-126 | 5p | CATTATTACTTTTGGTACGCG | 43 | 2 | 4.416666667 |
| ssc-miR-126-3p | ssc-mir-126 | 3p | TCGTACCGTGAGTAATAATGCG | 73 | 9 | 4.368421053 |
| ssc-miR-127 | ssc-mir-127 | 3p | TCGGATCCGTCTGAGCTTGGCT |  | 2 | 0.833333333 |
| ssc-miR-1271 | ssc-mir-1271 | 3p | TGCCTGCTATGTGCCAGGCA |  | 5 | 0.666666667 |
| ssc-miR-1277 | ssc-mir-1277 | 3p | TACGTAGATATATATGTATTTT |  |  |  |
| ssc-miR-128 | ssc-mir-128-1 | 3p | TCACAGTGAACCGGTCTCTTT | 1185 | 248 | 4.631782946 |
| ssc-miR-128 | ssc-mir-128-2 | 3p | TCACAGTGAACCGGTCTCTTT | 1185 | 248 | 4.631782946 |
| ssc-miR-1285 | ssc-mir-1285 | 3p | CTGGGCAACATAGCGAGACCCCGT |  | 27 | 0.27027027 |
| ssc-miR-1296-5p | ssc-mir-1296 | 5p | TTAGGGCCCTGGCTCCATCTCC | 6 |  | 1.6 |
| ssc-miR-1296-3p | ssc-mir-1296 | 3p | GAGTGGGGTTTTGACCCTAACC |  |  |  |
| ssc-miR-129a | ssc-mir-129a | 3p | AAGCCCTTACCCCAAAAAGCAT |  | 6 | 0.625 |
| ssc-miR-129b | ssc-mir-129b | 5p | CTTTTTGCGGTCTGGGCTTGC | 45 | 72 | 0.670731707 |
| ssc-miR-1306-5p | ssc-mir-1306 | 5p | CCACCTCCCCTGCAAACGTCCA |  | 7 | 0.588235294 |
| ssc-miR-1306-3p | ssc-mir-1306 | 3p | ACGTTGGCTCTGGTGGTGATG | 14 | 69 | 0.303797468 |
| ssc-miR-1307 | ssc-mir-1307 | 3p | ACTCGGCGTGGCGTCGGTCGTG | 4 | 107 | 0.11965812 |
| ssc-miR-130a | ssc-mir-130a | 3p | CAGTGCAATGTTAAAAGGGCAT | 29 | 3 | 3 |
| ssc-miR-130b | ssc-mir-130b | 3p | CAGTGCAATGATGAAAGGGCAT | 94 | 27 | 2.810810811 |
| ssc-miR-132 | ssc-mir-132 | 3p | TAACAGTCTACAGCCATGGTCG | 4 | 6 | 0.875 |
| ssc-miR-133a-5p | ssc-mir-133a-1 | 5p | AGCTGGTAAAATGGAACCAAAT |  |  |  |
| ssc-miR-133a-3p | ssc-mir-133a-1 | 3p | TTGGTCCCCTTCAACCAGCTG | 2 | 2 | 1 |
| ssc-miR-133a-5p | ssc-mir-133a-2 | 5p | AGCTGGTAAAATGGAACCAAAT |  |  |  |
| ssc-miR-133a-3p | ssc-mir-133a-2 | 3p | TTGGTCCCCTTCAACCAGCTG | 2 | 2 | 1 |
| ssc-miR-133b | ssc-mir-133b | 3p | TTTGGTCCCCTTCAACCAGCTAT |  |  |  |
| ssc-miR-1343 | ssc-mir-1343 | 3p | CTCCTGGGGCCCGCACTCTCGC | 4 | 4 | 1 |
| ssc-miR-135 | ssc-mir-135-1 | 5p | TATGGCTTTTTATTCCTATGTGA | 4 | 235 | 0.057142857 |
| ssc-miR-135 | ssc-mir-135-2 | 5p | TATGGCTTTTTATTCCTATGTGA | 4 | 235 | 0.057142857 |
| ssc-miR-136 | ssc-mir-136 | 5p | ACTCCATTTGTTTTGATGATGGA |  |  |  |
| ssc-miR-137 | ssc-mir-137 | 3p | TTATTGCTTAAGAATACGCGTAG |  |  |  |
| ssc-miR-138 | ssc-mir-138 | 5p | AGCTGGTGTTGTGAATCAGGC | 7 |  | 1.7 |
| ssc-miR-139-5p | ssc-mir-139 | 5p | TCTACAGTGCACGTGTCTCCAG | 141 | 44 | 2.796296296 |
| ssc-miR-139-3p | ssc-mir-139 | 3p | TGGAGACGCGGCCCTGTTGGAGT | 2 | 1 | 1.090909091 |
| ssc-miR-140-5p | ssc-mir-140 | 5p | AGTGGTTTTACCCTATGGTAG | 4 |  | 1.4 |
| ssc-miR-140-3p | ssc-mir-140 | 3p | TACCACAGGGTAGAACCACGGAC | 512 | 1026 | 0.503861004 |
| ssc-miR-142-5p | ssc-mir-142 | 5p | CATAAAGTAGAAAGCACTACT | 9 | 17 | 0.703703704 |
| ssc-miR-142-3p | ssc-mir-142 | 3p | TGTAGTGTTTCCTACTTTATGG |  | 238 | 0.040322581 |
| ssc-miR-143-5p | ssc-mir-143 | 5p | GGTGCAGTGCTGCATCTCTGG |  |  |  |
| ssc-miR-143-3p | ssc-mir-143 | 3p | TGAGATGAAGCACTGTAGCTC | 26 | 92 | 0.352941176 |
| ssc-miR-144 | ssc-mir-144 | 3p | TACAGTATAGATGATGTAC | 2 |  | 1.2 |
| ssc-miR-145-5p | ssc-mir-145 | 5p | GTCCAGTTTTCCCAGGAATCCCTT |  | 87 | 0.103092784 |
| ssc-miR-145-3p | ssc-mir-145 | 3p | GGATTCCTGGAAATACTGTTCT |  |  |  |
| ssc-miR-1468 | ssc-mir-1468 | 5p | CTCCGTTTGCCTGTTTTGCTGA |  |  |  |
| ssc-miR-146a-5p | ssc-mir-146a | 5p | TGAGAACTGAATTCCATGGGTT | 258 | 1767 | 0.150815982 |
| ssc-miR-146a-3p | ssc-mir-146a | 3p | CCTGTGAAGTTTAGTTCTTCAG |  |  |  |
| ssc-miR-146b | ssc-mir-146b | 5p | TGAGAACTGAATTCCATAGGC | 887 | 4 | 64.07142857 |
| ssc-miR-148a-5p | ssc-mir-148a | 5p | AAAGTTCTGAGACACTCCGACT |  |  |  |
| ssc-miR-148a-3p | ssc-mir-148a | 3p | TCAGTGCACTACAGAACTTTGT | 11 | 182 | 0.109375 |
| ssc-miR-148b-5p | ssc-mir-148b | 5p | GAAGTTCTGTTATACACTCAGGC | 32 | 5 | 2.8 |
| ssc-miR-148b-3p | ssc-mir-148b | 3p | TCAGTGCATCACAGAACTTTGT | 376 | 1616 | 0.237392374 |
| ssc-miR-149 | ssc-mir-149 | 5p | TCTGGCTCCGTGTCTTCACTCCC | 11 | 3 | 1.615384615 |
| ssc-miR-150 | ssc-mir-150-1 | 5p | TCTCCCAACCCTTGTACCAGTG |  |  |  |
| ssc-miR-150 | ssc-mir-150-2 | 5p | TCTCCCAACCCTTGTACCAGTG |  |  |  |
| ssc-miR-151-5p | ssc-mir-151 | 5p | TCGAGGAGCTCACAGTCTAGT | 459 | 149 | 2.949685535 |
| ssc-miR-151-3p | ssc-mir-151 | 3p | CTAGACTGAAGCTCCTTGAGGA | 1063 | 3522 | 0.303793884 |
| ssc-miR-152 | ssc-mir-152 | 3p | TCAGTGCATGACAGAACTTGG | 247 | 9880 | 0.025985844 |
| ssc-miR-153 | ssc-mir-153 | 3p | TTGCATAGTCACAAAAGTGA |  |  |  |
| ssc-miR-155-5p | ssc-mir-155 | 5p | TTAATGCTAATTGTGATAGGGG | 4372 | 62 | 60.86111111 |
| ssc-miR-155-3p | ssc-mir-155 | 3p | TCCTACATGTTAGCATTAACA |  | 3 | 0.769230769 |
| ssc-miR-15a | ssc-mir-15a | 5p | TAGCAGCACATAATGGTTTGT | 42 | 156 | 0.313253012 |
| ssc-miR-15b | ssc-mir-15b | 5p | TAGCAGCACATCATGGTTTACA | 222 | 812 | 0.282238443 |
| ssc-miR-16 | ssc-mir-16-1 | 5p | TAGCAGCACGTAAATATTGGCG | 13628 | 1891 | 7.174118885 |
| ssc-miR-16 | ssc-mir-16-2 | 5p | TAGCAGCACGTAAATATTGGCG | 13628 | 1891 | 7.174118885 |
| ssc-miR-17-5p | ssc-mir-17 | 5p | CAAAGTGCTTACAGTGCAGGTAG | 5297 | 438 | 11.84598214 |
| ssc-miR-17-3p | ssc-mir-17 | 3p | ACTGCAGTGAAGGCACTTGTAG | 20 | 15 | 1.2 |
| ssc-miR-181a | ssc-mir-181a-1 | 5p | AACATTCAACGCTGTCGGTGAGTT | 278 | 147 | 1.834394904 |
| ssc-miR-181a | ssc-mir-181a-2 | 5p | AACATTCAACGCTGTCGGTGAGTT | 278 | 147 | 1.834394904 |
| ssc-miR-181b | ssc-mir-181b-1 | 5p | AACATTCATTGCTGTCGGTGGGTT | 91 | 125 | 0.748148148 |
| ssc-miR-181b | ssc-mir-181b-2 | 5p | AACATTCATTGCTGTCGGTGGGTT | 91 | 125 | 0.748148148 |
| ssc-miR-181c | ssc-mir-181c | 5p | AACATTCAACCTGTCGGTGAGT |  |  |  |
| ssc-miR-181d-5p | ssc-mir-181d | 5p | AACATTCATTGTTGTCGGTGGGTT | 3 | 3 | 1 |
| ssc-miR-181d-3p | ssc-mir-181d | 3p | CCCACCGAGGGATGAATGTCAC |  |  |  |
| ssc-miR-182 | ssc-mir-182 | 5p | TTTGGCAATGGTAGAACTCACACT | 12259 | 4616 | 2.652183312 |
| ssc-miR-183 | ssc-mir-183 | 5p | TATGGCACTGGTAGAATTCACTG | 196 | 3132 | 0.065563335 |
| ssc-miR-1839-5p | ssc-mir-1839 | 5p | AAGGTAGATAGAACAGGTCTTG | 2 | 3 | 0.923076923 |
| ssc-miR-1839-3p | ssc-mir-1839 | 3p | AGACCTACTTTTCTACCAACA |  |  |  |
| ssc-miR-184 | ssc-mir-184 | 3p | TGGACGGAGAACTGATAAGGGT | 195 | 359 | 0.555555556 |
| ssc-miR-185 | ssc-mir-185 | 5p | TGGAGAGAAAGGCAGTTCCTGA | 12 | 1621 | 0.013488657 |
| ssc-miR-186 | ssc-mir-186 | 5p | CAAAGAATTCTCCTTTTGGGCTT | 326 | 113 | 2.731707317 |
| ssc-miR-187 | ssc-mir-187 | 3p | TCGTGTCTTGTGTTGCAGCCGG |  | 3 | 0.769230769 |
| ssc-miR-18a | ssc-mir-18a | 5p | TAAGGTGCATCTAGTGCAGATA | 350 | 9541 | 0.037692388 |
| ssc-miR-18b | ssc-mir-18b | 5p | TAAGGTGCATCTAGTGCAGTTAG | 2 |  | 1.2 |
| ssc-miR-190a | ssc-mir-190a | 5p | TGATATGTTTGATATATTAGG | 29 | 1 | 3.545454545 |
| ssc-miR-190b | ssc-mir-190b | 5p | TGATATGTTTGATATTGGGTTG | 23 | 1 | 3 |
| ssc-miR-191 | ssc-mir-191 | 5p | CAACGGAATCCCAAAAGCAGCTG | 3729 | 156 | 22.52409639 |
| ssc-miR-192 | ssc-mir-192 | 5p | CTGACCTATGAATTGACAGCC | 16353 | 102 | 146.0982143 |
| ssc-miR-193a-5p | ssc-mir-193a | 5p | TGGGTCTTTGCGGGCGAGATGA |  | 201 | 0.047393365 |
| ssc-miR-193a-3p | ssc-mir-193a | 3p | AACTGGCCTACAAAGTCCCAGT | 4 | 45 | 0.254545455 |
| ssc-miR-194a | ssc-mir-194a | 5p | TGTAACAGCAACTCCATGTGG | 13 | 541 | 0.041742287 |
| ssc-miR-194b-5p | ssc-mir-194b | 5p | TGTAACAGCGACTCCATGTGGA |  | 27 | 0.27027027 |
| ssc-miR-194b-3p | ssc-mir-194b | 3p | CCAGTGGAGATGCTGTTACCTT |  |  |  |
| ssc-miR-195 | ssc-mir-195 | 5p | TAGCAGCACAGAAATATTGGC | 9 | 192 | 0.094059406 |
| ssc-miR-196a | ssc-mir-196a-1 | 5p | TAGGTAGTTTCATGTTGTTGGG |  |  |  |
| ssc-miR-196a | ssc-mir-196a-2 | 5p | TAGGTAGTTTCATGTTGTTGGG |  |  |  |
| ssc-miR-196b-5p | ssc-mir-196b-1 | 5p | TAGGTAGTTTCCTGTTGTTGGG | 121 | 984 | 0.131790744 |
| ssc-miR-196b-3p | ssc-mir-196b-1 | 3p | CGACAGCACGACACTGCCTTCA |  |  |  |
| ssc-miR-196b | ssc-mir-196b-2 | 5p | TAGGTAGTTTCCTGTTGTTGGG | 121 | 984 | 0.131790744 |
| ssc-miR-199a-5p | ssc-mir-199a-1 | 5p | CCCAGTGTTCAGACTACCTGTTC |  |  |  |
| ssc-miR-199a-3p | ssc-mir-199a-1 | 3p | ACAGTAGTCTGCACATTGGTTA |  | 4 | 0.714285714 |
| ssc-miR-199a-5p | ssc-mir-199a-2 | 5p | CCCAGTGTTCAGACTACCTGTTC |  |  |  |
| ssc-miR-199a-3p | ssc-mir-199a-2 | 3p | ACAGTAGTCTGCACATTGGTTA |  | 4 | 0.714285714 |
| ssc-miR-199b-5p | ssc-mir-199b | 5p | CCCAGTGTTTAGACTATCTGTT | 3 |  | 1.3 |
| ssc-miR-199b-3p | ssc-mir-199b | 3p | TACAGTAGTCTGCACATTGGTT |  | 4 | 0.714285714 |
| ssc-miR-19a | ssc-mir-19a | 3p | TGTGCAAATCTATGCAAAACTGA | 203 | 6339 | 0.03354859 |
| ssc-miR-19b | ssc-mir-19b-1 | 3p | TGTGCAAATCCATGCAAAACTGA | 365 | 14587 | 0.02569021 |
| ssc-miR-19b | ssc-mir-19b-2 | 3p | TGTGCAAATCCATGCAAAACTGA | 365 | 14587 | 0.02569021 |
| ssc-miR-202-5p | ssc-mir-202 | 5p | TTCCTATGCATATACTTCTTT |  |  |  |
| ssc-miR-202-3p | ssc-mir-202 | 3p | AGAGGTGTAGGCATGGGAA |  |  |  |
| ssc-miR-204 | ssc-mir-204 | 5p | TTCCCTTTGTCATCCTATGCCT |  |  |  |
| ssc-miR-205 | ssc-mir-205 | 5p | TCCTTCATTCCACCGGAGTCTG |  |  |  |
| ssc-miR-206 | ssc-mir-206 | 3p | TGGAATGTAAGGAAGTGTGTGA | 2 |  | 1.2 |
| ssc-miR-208b | ssc-mir-208b | 3p | ATAAGACGAACAAAAGGTTTGT |  |  |  |
| ssc-miR-20a | ssc-mir-20a | 5p | TAAAGTGCTTATAGTGCAGGTA | 2946 | 116 | 23.46031746 |
| ssc-miR-20b | ssc-mir-20b-1 | 5p | CAAAGTGCTCACAGTGCAGGTAG |  |  |  |
| ssc-miR-20b | ssc-mir-20b-2 | 5p | CAAAGTGCTCACAGTGCAGGTAG |  |  |  |
| ssc-miR-21 | ssc-mir-21 | 5p | TAGCTTATCAGACTGATGTTGA | 38972 | 382830 | 0.101823216 |
| ssc-miR-210 | ssc-mir-210 | 3p | CTGTGCGTGTGACAGCGGCTGA | 304 | 123 | 2.360902256 |
| ssc-miR-212 | ssc-mir-212 | 5p | ACCTTGGCTCTAGACTGCTTACT |  |  |  |
| ssc-miR-214 | ssc-mir-214 | 3p | ACAGCAGGCACAGACAGGCAG |  |  |  |
| ssc-miR-215 | ssc-mir-215 | 5p | ATGACCTATGAATTGACAGAC |  | 31 | 0.243902439 |
| ssc-miR-216 | ssc-mir-216-1 | 5p | TAATCTCAGCTGGCAACTGTGAG |  |  |  |
| ssc-miR-216 | ssc-mir-216-2 | 5p | TAATCTCAGCTGGCAACTGTGAG |  |  |  |
| ssc-miR-217 | ssc-mir-217-1 | 5p | TACTGCATCAGGAACTGATTGGAT |  |  |  |
| ssc-miR-217 | ssc-mir-217-2 | 5p | TACTGCATCAGGAACTGATTGGAT |  |  |  |
| ssc-miR-218-5p | ssc-mir-218-1 | 5p | TTGTGCTTGATCTAACCATGT | 2 | 7 | 0.705882353 |
| ssc-miR-218-3p | ssc-mir-218-1 | 3p | ATGGTTCTGTCAAGCACCATG |  |  |  |
| ssc-miR-218 | ssc-mir-218-2 | 5p | TTGTGCTTGATCTAACCATGT | 2 | 7 | 0.705882353 |
| ssc-miR-218b | ssc-mir-218b | 5p | TTGTGCTTGATCTAACCATGTG | 2 | 7 | 0.705882353 |
| ssc-miR-219 | ssc-mir-219 | 3p | AGAGTTGAGTCTGGACGTCCCG |  | 2 | 0.833333333 |
| ssc-miR-22-5p | ssc-mir-22 | 5p | AGTTCTTCAGTGGCAAGCTTTA | 3 | 1 | 1.181818182 |
| ssc-miR-22-3p | ssc-mir-22 | 3p | AAGCTGCCAGTTGAAGAACTGT | 1102 | 365 | 2.965333333 |
| ssc-miR-221-5p | ssc-mir-221 | 5p | ACCTGGCATACAATGTAGATTTCTGT | 2 | 39 | 0.244897959 |
| ssc-miR-221-3p | ssc-mir-221 | 3p | AGCTACATTGTCTGCTGGGTTT | 845 | 147 | 5.445859873 |
| ssc-miR-222 | ssc-mir-222 | 3p | AGCTACATCTGGCTACTGGGTCTC | 113 | 284 | 0.418367347 |
| ssc-miR-224 | ssc-mir-224 | 5p | CAAGTCACTAGTGGTTCCGTTTA | 3 |  | 1.3 |
| ssc-miR-2320-5p | ssc-mir-2320 | 5p | TGGCACAGGGTCCAGCTGTCGG |  | 114 | 0.080645161 |
| ssc-miR-2320-3p | ssc-mir-2320 | 3p | CGATGATGGTCCCTGTGTTTG | 39 | 51 | 0.803278689 |
| ssc-miR-2366 | ssc-mir-2366-1 | 3p | TGGGTCACAGAAGAGGGTCTGG |  |  |  |
| ssc-miR-2366 | ssc-mir-2366-2 | 3p | TGGGTCACAGAAGAGGGTCTGG |  |  |  |
| ssc-miR-23a | ssc-mir-23a | 3p | ATCACATTGCCAGGGATTTCC | 140 | 635 | 0.23255814 |
| ssc-miR-23b | ssc-mir-23b | 3p | ATCACATTGCCAGGGATTACCA | 67 | 143 | 0.503267974 |
| ssc-miR-24-1-5p | ssc-mir-24-1 | 5p | GTGCCTACTGAGCTGAAACACAGT | 11 |  | 2.1 |
| ssc-miR-24-3p | ssc-mir-24-1 | 3p | TGGCTCAGTTCAGCAGGAACAG | 61 | 10251 | 0.006919404 |
| ssc-miR-24-2-5p | ssc-mir-24-2 | 5p | GTGCCTACTGAGCTGATATCAGT |  |  |  |
| ssc-miR-24-3p | ssc-mir-24-2 | 3p | TGGCTCAGTTCAGCAGGAACAG | 61 | 10251 | 0.006919404 |
| ssc-miR-2411 | ssc-mir-2411 | 5p | TGGAGTGACTGTCAGATGCAGC | 22 | 12 | 1.454545455 |
| ssc-miR-2483 | ssc-mir-2483 | 3p | AAACATCTGGTTGGTTGAGAGA | 2 | 13 | 0.52173913 |
| ssc-miR-26a | ssc-mir-26a | 5p | TTCAAGTAATCCAGGATAGGCT | 14568 | 3198 | 4.544264339 |
| ssc-miR-27a | ssc-mir-27a | 3p | TTCACAGTGGCTAAGTTCCGC | 5247 | 549 | 9.404293381 |
| ssc-miR-27b-5p | ssc-mir-27b | 5p | AGAGCTTAGCTGATTGGTGAACA | 4 | 63 | 0.191780822 |
| ssc-miR-27b-3p | ssc-mir-27b | 3p | TTCACAGTGGCTAAGTTCTGC | 6403 | 172 | 35.23626374 |
| ssc-miR-28-5p | ssc-mir-28 | 5p | AAGGAGCTCACAGTCTATTGAG | 7 | 232 | 0.070247934 |
| ssc-miR-28-3p | ssc-mir-28 | 3p | CACTAGATTGTGAGCTCCTGGA | 815 | 617 | 1.315789474 |
| ssc-miR-296-5p | ssc-mir-296 | 5p | GAGGGCCCCCCCCAATCCTGT |  | 1 | 0.909090909 |
| ssc-miR-296-3p | ssc-mir-296 | 3p | AGGGTTGGGCGGAGGCTTTCC | 49 | 3 | 4.538461538 |
| ssc-miR-299 | ssc-mir-299 | 5p | ATGGTTTACCGTCCCACATAC |  |  |  |
| ssc-miR-29a | ssc-mir-29a | 3p | CTAGCACCATCTGAAATCGGTTA | 89 | 3939 | 0.025069638 |
| ssc-miR-29b | ssc-mir-29b-1 | 3p | TAGCACCATTTGAAATCAGTGTT | 55 | 209 | 0.296803653 |
| ssc-miR-29b | ssc-mir-29b-2 | 3p | TAGCACCATTTGAAATCAGTGTT | 55 | 209 | 0.296803653 |
| ssc-miR-29c | ssc-mir-29c | 3p | TAGCACCATTTGAAATCGGTTA | 11 | 366 | 0.055851064 |
| ssc-miR-301 | ssc-mir-301 | 3p | CAGTCCAATAGTATTGTCAAAGC |  | 17 | 0.37037037 |
| ssc-miR-30a-5p | ssc-mir-30a | 5p | TGTAAACATCCTCGACTGGAAG | 103201 | 6320 | 16.30505529 |
| ssc-miR-30a-3p | ssc-mir-30a | 3p | CTTTCAGTCGGATGTTTGCAGC | 145 | 1134 | 0.13548951 |
| ssc-miR-30b-5p | ssc-mir-30b | 5p | TGTAAACATCCTACACTCAGCT | 469 | 191 | 2.383084577 |
| ssc-miR-30b-3p | ssc-mir-30b | 3p | CTGGGAGGTGGATGTTTACTT |  | 1 | 0.909090909 |
| ssc-miR-30c-5p | ssc-mir-30c-1 | 5p | TGTAAACATCCTACACTCTCAGC | 985 | 209 | 4.543378995 |
| ssc-miR-30c-1-3p | ssc-mir-30c-1 | 3p | CTGGGAGAGGGTTGTTTACT |  |  |  |
| ssc-miR-30c-5p | ssc-mir-30c-2 | 5p | TGTAAACATCCTACACTCTCAGC | 985 | 209 | 4.543378995 |
| ssc-miR-30c-3p | ssc-mir-30c-2 | 3p | CTGGGAGAAGGCTGTTTACTCT | 28 | 36 | 0.826086957 |
| ssc-miR-30d | ssc-mir-30d | 5p | TGTAAACATCCCCGACTGGAAGCT | 10678 | 1786 | 5.951002227 |
| ssc-miR-30e-5p | ssc-mir-30e | 5p | TGTAAACATCCTTGACTGGAAGCT | 1795 | 812 | 2.195863747 |
| ssc-miR-30e-3p | ssc-mir-30e | 3p | CTTTCAGTCGGATGTTTACAGC | 59 | 347 | 0.193277311 |
| ssc-miR-31 | ssc-mir-31 | 5p | AGGCAAGATGCTGGCATAGCTG | 32 | 3118 | 0.01342711 |
| ssc-miR-32 | ssc-mir-32 | 5p | TATTGCACATTACTAAGTTGC |  | 79 | 0.112359551 |
| ssc-miR-320 | ssc-mir-320 | 3p | AAAAGCTGGGTTGAGAGGGCGAA | 509 | 321 | 1.567975831 |
| ssc-miR-323 | ssc-mir-323 | 3p | GCACATTACACGGTCGACCTCT | 4 | 1 | 1.272727273 |
| ssc-miR-324 | ssc-mir-324 | 5p | CGCATCCCCTAGGGCATTGGTGT | 3 | 42 | 0.25 |
| ssc-miR-325 | ssc-mir-325 | 5p | CCTAGTAGGTGTTCAGTAAGTGT |  |  |  |
| ssc-miR-326 | ssc-mir-326 | 3p | CCTCTGGGCCCTTCCTCCAG |  |  |  |
| ssc-miR-328 | ssc-mir-328 | 3p | CTGGCCCTCTCTGCCCTTCCGT |  | 2 | 0.833333333 |
| ssc-miR-331-5p | ssc-mir-331 | 5p | TCTAGGTATGGTCCCAGGGAT | 3 | 1 | 1.181818182 |
| ssc-miR-331-3p | ssc-mir-331 | 3p | GCCCCTGGGCCTATCCTAGAA | 2 | 27 | 0.324324324 |
| ssc-miR-335 | ssc-mir-335 | 5p | TCAAGAGCAATAACGAAAAATG | 45 | 74 | 0.654761905 |
| ssc-miR-338 | ssc-mir-338 | 3p | TCCAGCATCAGTGATTTTGTTG | 2 | 5 | 0.8 |
| ssc-miR-339-5p | ssc-mir-339-1 | 5p | TCCCTGTCCTCCAGGAGCTCAC | 85 | 1 | 8.636363636 |
| ssc-miR-339-3p | ssc-mir-339-1 | 3p | AGCTCCTCGAGGCCAGAGCCC |  |  |  |
| ssc-miR-339 | ssc-mir-339-2 | 5p | TCCCTGTCCTCCAGGAGCTCA | 85 | 1 | 8.636363636 |
| ssc-miR-340 | ssc-mir-340-1 | 5p | TTATAAAGCAATGAGACTGATT | 463 | 227 | 1.995780591 |
| ssc-miR-340 | ssc-mir-340-2 | 5p | TTATAAAGCAATGAGACTGATT | 463 | 227 | 1.995780591 |
| ssc-miR-342 | ssc-mir-342 | 3p | TCTCACACAGAAATCGCACCCGTCA | 4 | 9 | 0.736842105 |
| ssc-miR-345-5p | ssc-mir-345-1 | 5p | GCTGACTCCTAGTCCAGTGC | 2 | 2 | 1 |
| ssc-miR-345-3p | ssc-mir-345-1 | 3p | CCCTGAACTAGGGGTCTGGAG |  |  |  |
| ssc-miR-345-5p | ssc-mir-345-2 | 5p | GCTGACTCCTAGTCCAGTGC | 2 | 2 | 1 |
| ssc-miR-345-3p | ssc-mir-345-2 | 3p | CCCTGAACTAGGGGTCTGGAG |  |  |  |
| ssc-miR-34a | ssc-mir-34a | 5p | TGGCAGTGTCTTAGCTGGTTGT | 892 | 37 | 19.19148936 |
| ssc-miR-34c | ssc-mir-34c-1 | 5p | AGGCAGTGTAGTTAGCTGATTGC | 7 |  | 1.7 |
| ssc-miR-34c | ssc-mir-34c-2 | 5p | AGGCAGTGTAGTTAGCTGATTGC | 7 |  | 1.7 |
| ssc-miR-361-5p | ssc-mir-361 | 5p | TTATCAGAATCTCCAGGGGTAC | 9 | 704 | 0.026610644 |
| ssc-miR-361-3p | ssc-mir-361 | 3p | CCCCCAGGTGTGATTCTGATTTGC | 105 | 29 | 2.948717949 |
| ssc-miR-3613 | ssc-mir-3613 | 5p | TGTTGTACTTTTTTTTTTGT | 20 | 6 | 1.875 |
| ssc-miR-362 | ssc-mir-362 | 5p | AATCCTTGGAACCTAGGTGTGAGTG | 10 | 8 | 1.111111111 |
| ssc-miR-363 | ssc-mir-363-1 | 3p | AATTGCACGGTATCCATCTGTAA |  |  |  |
| ssc-miR-363 | ssc-mir-363-2 | 3p | AATTGCACGGTATCCATCTGTAA |  |  |  |
| ssc-miR-365-3p | ssc-mir-365-1 | 3p | TAATGCCCCTAAAAATCCTTAT | 16 | 21 | 0.838709677 |
| ssc-miR-365-5p | ssc-mir-365-2 | 5p | GAGGGACTTTCAGGGGCAGCTGT | 3 | 3 | 1 |
| ssc-miR-365-3p | ssc-mir-365-2 | 3p | TAATGCCCCTAAAAATCCTTAT | 16 | 21 | 0.838709677 |
| ssc-miR-369 | ssc-mir-369 | 3p | AATAATACATGGTTGATCTTT |  | 11 | 0.476190476 |
| ssc-miR-370 | ssc-mir-370 | 3p | GCCTGCTGGGGTGGAACCTGGT |  |  |  |
| ssc-miR-374a-5p | ssc-mir-374a | 5p | TTATAATACAACCTGATAAGTG | 23 | 72 | 0.402439024 |
| ssc-miR-374a-3p | ssc-mir-374a | 3p | CTTATCAGGTTGTATTGTAATT | 207 | 89 | 2.191919192 |
| ssc-miR-374b-5p | ssc-mir-374b | 5p | ATATAATACAACCTGCTAAGTG | 12 | 6 | 1.375 |
| ssc-miR-374b-3p | ssc-mir-374b | 3p | CTTATCAGGTTGTATTATCATT | 8 | 34 | 0.409090909 |
| ssc-miR-376a-5p | ssc-mir-376a | 5p | GTAGATTCTCCTTCTATGAGTAC |  |  |  |
| ssc-miR-376a-3p | ssc-mir-376a | 3p | ATCATAGAGGAAAATCCACGT |  |  |  |
| ssc-miR-376b | ssc-mir-376b | 5p | GTGGCTATTCCTTCTATGTTTA |  |  |  |
| ssc-miR-376c | ssc-mir-376c | 5p | GTGGATATTCCTTCTATGTTTA |  |  |  |
| ssc-miR-378 | ssc-mir-378-1 | 3p | ACTGGACTTGGAGTCAGAAGGC | 2110 | 927 | 2.262540021 |
| ssc-miR-378 | ssc-mir-378-2 | 3p | ACTGGACTTGGAGTCAGAAGGC | 2110 | 927 | 2.262540021 |
| ssc-miR-381 | ssc-mir-381 | 5p | AGCGAGGTTGCCCTTTGTATATT |  |  |  |
| ssc-miR-382 | ssc-mir-382 | 5p | AAGTTGTTCGTGGTGGATTCG | 8 | 3 | 1.384615385 |
| ssc-miR-383 | ssc-mir-383 | 3p | CCACAGCACTGCCTGGTCAGA |  | 38 | 0.208333333 |
| ssc-miR-411 | ssc-mir-411 | 3p | ATGTAACACGGTCCACTAAC | 10 | 8 | 1.111111111 |
| ssc-miR-421-5p | ssc-mir-421 | 5p | CCTCATTAAATGTTTGTTGAATGA |  |  |  |
| ssc-miR-421-3p | ssc-mir-421 | 3p | ATCAACAGACATTAATTGGGCGC | 21 | 38 | 0.645833333 |
| ssc-miR-423-5p | ssc-mir-423 | 5p | TGAGGGGCAGAGAGCGAGACTTT | 226 | 2493 | 0.094286856 |
| ssc-miR-423-3p | ssc-mir-423 | 3p | AGCTCGGTCTGAGGCCCCTCAGT | 248 | 246 | 1.0078125 |
| ssc-miR-424-5p | ssc-mir-424 | 5p | CAGCAGCAATTCATGTTTTGAA | 10 | 2182 | 0.009124088 |
| ssc-miR-424-3p | ssc-mir-424 | 3p | CAAAACGTGAGGCGCTGCTAT | 42 |  | 5.2 |
| ssc-miR-425-5p | ssc-mir-425 | 5p | AATGACACGATCACTCCCGTTGA | 90 | 438 | 0.223214286 |
| ssc-miR-425-3p | ssc-mir-425 | 3p | ATCGGGAATGTCGTGTCCGCCC | 47 | 3 | 4.384615385 |
| ssc-miR-429 | ssc-mir-429 | 3p | TAATACTGTCTGGTAATGCCGT | 44 | 130 | 0.385714286 |
| ssc-miR-432-5p | ssc-mir-432 | 5p | TCTTGGAGTAGGTCATTGGGT |  |  |  |
| ssc-miR-432-3p | ssc-mir-432 | 3p | TGGATGGCTCCTCCATGGCT |  |  |  |
| ssc-miR-4331 | ssc-mir-4331 | 3p | TGTGGCTGTGGTGTAGGCCAGC | 72 | 2 | 6.833333333 |
| ssc-miR-4332 | ssc-mir-4332 | 3p | CACGGCCGCCGCCGGGCGCC |  | 10 | 0.5 |
| ssc-miR-4333 | ssc-mir-4333 | 5p | ATACCTGCATGTTAGTCTTTGGTTCT |  |  |  |
| ssc-miR-4334-5p | ssc-mir-4334 | 5p | CCCTGGAGTGACGGGGGTG |  |  |  |
| ssc-miR-4334-3p | ssc-mir-4334 | 3p | TCCCTGTCCTCCAGGAGCTC | 85 | 1 | 8.636363636 |
| ssc-miR-4335 | ssc-mir-4335 | 3p | GTGCCCAGCGCTGCAGGGCA |  |  |  |
| ssc-miR-4336 | ssc-mir-4336 | 5p | CAACTCTGTGGTTTCCTTTACTCATAG |  |  |  |
| ssc-miR-4337 | ssc-mir-4337 | 5p | AGGGTATATAAGCCTTCACTGG |  |  |  |
| ssc-miR-4338 | ssc-mir-4338 | 5p | ATGTTCAGTCTCAGTGGGAACC |  |  |  |
| ssc-miR-4339 | ssc-mir-4339 | 5p | GCTCTGAGCTGCCCCTCCTCGTCC |  |  |  |
| ssc-miR-450a | ssc-mir-450a | 5p | TTTTGCGATGTGTTCCTAATAT | 16 | 5 | 1.733333333 |
| ssc-miR-450b-5p | ssc-mir-450b | 5p | TTTTGCAATATGTTCCTGAATA | 7 | 40 | 0.34 |
| ssc-miR-450b-3p | ssc-mir-450b | 3p | TTGGGAACATTTTGCATCCAT | 2 |  | 1.2 |
| ssc-miR-450c-5p | ssc-mir-450c | 5p | TTTTGCGATGTGTTCCTAATAC | 164 | 6 | 10.875 |
| ssc-miR-450c-3p | ssc-mir-450c | 3p | ATTGGGAACATTTTGCATTCGT | 2 | 1 | 1.090909091 |
| ssc-miR-451 | ssc-mir-451 | 5p | AAACCGTTACCATTACTGAGTT | 8 |  | 1.8 |
| ssc-miR-452 | ssc-mir-452 | 5p | AACTGTTTGCAGAGGAAACTGA |  |  |  |
| ssc-miR-455-5p | ssc-mir-455 | 5p | TATGTGCCTTTGGACTACATCG | 35 | 3 | 3.461538462 |
| ssc-miR-455-3p | ssc-mir-455 | 3p | GCAGTCCATGGGCATATACAC | 10 |  | 2 |
| ssc-miR-484 | ssc-mir-484 | 3p | CCCAGGGGGCGACCCAGGCT |  |  |  |
| ssc-miR-486 | ssc-mir-486-1 | 3p | TCCTGTACTGAGCTGCCCCGAG | 142 | 8 | 8.444444444 |
| ssc-miR-486 | ssc-mir-486-2 | 5p | TCCTGTACTGAGCTGCCCCGAG | 142 | 8 | 8.444444444 |
| ssc-miR-487b | ssc-mir-487b | 5p | GTGGTTATCCCTGTCCTGTTCG |  |  |  |
| ssc-miR-489 | ssc-mir-489 | 3p | AGTGACATCACATATACGGCGG | 16 | 158 | 0.154761905 |
| ssc-miR-490-5p | ssc-mir-490-1 | 5p | CCATGGATCCCCAGGTGGGT |  | 14 | 0.416666667 |
| ssc-miR-490-3p | ssc-mir-490-1 | 3p | CAACCTGGAGGACTCCATGCTG | 2 | 24 | 0.352941176 |
| ssc-miR-490 | ssc-mir-490-2 | 3p | CAACCTGGAGGACTCCATGCTG | 2 | 24 | 0.352941176 |
| ssc-miR-491 | ssc-mir-491 | 5p | AGTGGGGAACCCTTCCATGAGG |  | 10 | 0.5 |
| ssc-miR-493-5p | ssc-mir-493 | 5p | TTGTACATGGTAGGCTTTCATT |  |  |  |
| ssc-miR-493-3p | ssc-mir-493 | 3p | TGAAGGTCTACTGTGTGCCAGG |  |  |  |
| ssc-miR-494 | ssc-mir-494 | 5p | AGGTTGTCGTGTTGTCTTCTCT |  |  |  |
| ssc-miR-497 | ssc-mir-497 | 5p | CAGCAGCACACTGTGGTTTGT | 11 | 3 | 1.615384615 |
| ssc-miR-499-5p | ssc-mir-499 | 5p | TTAAGACTTGCAGTGATGTTT | 4 | 472 | 0.029045643 |
| ssc-miR-499-3p | ssc-mir-499 | 3p | AACATCACAGCAAGTCTGTGCT |  | 1 | 0.909090909 |
| ssc-miR-500 | ssc-mir-500 | 3p | ATGCACCTGGGCAAGGATTCT | 29 | 31 | 0.951219512 |
| ssc-miR-503 | ssc-mir-503 | 5p | TAGCAGCGGGAACAGTACTGCAG | 11 | 67 | 0.272727273 |
| ssc-miR-504 | ssc-mir-504 | 5p | AGACCCTGGTCTGCACTCTATCT |  |  |  |
| ssc-miR-505 | ssc-mir-505 | 3p | TCAACACTTGCTGGTTTCCTCT | 37 | 79 | 0.528089888 |
| ssc-miR-532-5p | ssc-mir-532 | 5p | CATGCCTTGAGTGTAGGACCGT | 324 | 564 | 0.581881533 |
| ssc-miR-532-3p | ssc-mir-532 | 3p | CCTCCCACACCCAAGGCTTGCA | 4 | 3 | 1.076923077 |
| ssc-miR-542-5p | ssc-mir-542 | 5p | TCGGGGATCATCATGTCACGA |  | 277 | 0.034843206 |
| ssc-miR-542-3p | ssc-mir-542 | 3p | TGTGACAGATTGATAACTGAAA | 79 | 1446 | 0.061126374 |
| ssc-miR-545-5p | ssc-mir-545 | 5p | TCAGTAAATGTTTATTGGATG | 13 | 19 | 0.793103448 |
| ssc-miR-545-3p | ssc-mir-545 | 3p | ATCAACAAACATTTATTGTGTG |  | 5 | 0.666666667 |
| ssc-miR-551a | ssc-mir-551a | 3p | GCGACCCACTCTTGGTTTCC |  |  |  |
| ssc-miR-574 | ssc-mir-574 | 3p | CACGCTCATGCACACACCCACA | 9 | 6 | 1.1875 |
| ssc-miR-582 | ssc-mir-582 | 3p | TAACCGGTTGAACAACTGAACC | 7 | 1 | 1.545454545 |
| ssc-miR-615 | ssc-mir-615 | 3p | TCCGAGCCTGGGTCTCCCTCT |  |  |  |
| ssc-miR-628 | ssc-mir-628 | 5p | ATGCTGACATATTTACTAGAGG |  | 3 | 0.769230769 |
| ssc-miR-652 | ssc-mir-652 | 5p | ACAACCCTAGGAGAGGGTGCCATTCA |  |  |  |
| ssc-miR-664-5p | ssc-mir-664 | 5p | CAGGCTAGGAGAAGTGATTGGAT | 33 | 2 | 3.583333333 |
| ssc-miR-664-3p | ssc-mir-664 | 3p | TATTCATTTATCTCCCAGCCTACA |  |  |  |
| ssc-miR-671-5p | ssc-mir-671 | 5p | AGGAAGCCCTGGAGGGGCTGGAGG |  | 52 | 0.161290323 |
| ssc-miR-671-3p | ssc-mir-671 | 3p | TCCGGTTCTCAGGGCTCCACC |  | 1 | 0.909090909 |
| ssc-miR-676-5p | ssc-mir-676-1 | 5p | CTCTTCAATCTCAGGACTCGCA |  |  |  |
| ssc-miR-676-3p | ssc-mir-676-1 | 3p | CCGTCCTAAGGTTGTTGAGTT |  |  |  |
| ssc-miR-676-3p | ssc-mir-676-2 | 3p | CCGTCCTAAGGTTGTTGAGTT |  |  |  |
| ssc-miR-7 | ssc-mir-7-1 | 5p | TGGAAGACTAGTGATTTTGTTGTT | 4565 | 489 | 9.168336673 |
| ssc-miR-7 | ssc-mir-7-2 | 5p | TGGAAGACTAGTGATTTTGTTGTT | 4565 | 489 | 9.168336673 |
| ssc-miR-708-5p | ssc-mir-708 | 5p | AAGGAGCTTACAATCTAGCTGGG |  | 9 | 0.526315789 |
| ssc-miR-708-3p | ssc-mir-708 | 3p | CAACTAGACTGTGAGCTTCTAGA |  | 1 | 0.909090909 |
| ssc-miR-744 | ssc-mir-744 | 5p | TGCGGGGCTAGGGCTAACAGCA | 58 | 42 | 1.307692308 |
| ssc-miR-758 | ssc-mir-758 | 3p | TTTGTGACCTGGTCCACTAAC |  | 2 | 0.833333333 |
| ssc-miR-769-5p | ssc-mir-769 | 5p | TGAGACCTCTGGGTTCTGAGC | 223 | 55 | 3.584615385 |
| ssc-miR-769-3p | ssc-mir-769 | 3p | CTGGGATCTCTGGGGTCTTGGTT | 4 | 1 | 1.272727273 |
| ssc-miR-874 | ssc-mir-874 | 3p | CTGCCCTGGCCCGAGGGACCGAC |  |  |  |
| ssc-miR-885-5p | ssc-mir-885 | 5p | TCCATTACACTACCCTGCCTCT |  |  |  |
| ssc-miR-885-3p | ssc-mir-885 | 3p | AGGCAGCGGGGTGTAGTGGAT | 6 | 1 | 1.454545455 |
| ssc-miR-9-1 | ssc-mir-9-1 | 5p | TCTTTGGTTATCTAGCTGTATGA | 49 | 68 | 0.756410256 |
| ssc-miR-9-2 | ssc-mir-9-2 | 5p | TCTTTGGTTATCTAGCTGTATGA | 49 | 68 | 0.756410256 |
| ssc-miR-9 | ssc-mir-9-3 | 5p | TCTTTGGTTATCTAGCTGTATG | 49 | 68 | 0.756410256 |
| ssc-miR-92a | ssc-mir-92a-1 | 3p | TATTGCACTTGTCCCGGCCTGT | 3205 | 1148 | 2.776338515 |
| ssc-miR-92a | ssc-mir-92a-2 | 3p | TATTGCACTTGTCCCGGCCTGT | 3205 | 1148 | 2.776338515 |
| ssc-miR-92b-5p | ssc-mir-92b | 5p | AGGGACGGGACGCGGTGCAGTGTT | 6 | 1 | 1.454545455 |
| ssc-miR-92b-3p | ssc-mir-92b | 3p | TATTGCACTCGTCCCGGCCTCC | 1680 | 133 | 11.81818182 |
| ssc-miR-935 | ssc-mir-935 | 3p | CCAGTTACCGCTTCCGCTACCGC |  |  |  |
| ssc-miR-95 | ssc-mir-95 | 3p | TTCAACGGGTATTTATTGAGCA |  | 1 | 0.909090909 |
| ssc-miR-98 | ssc-mir-98 | 5p | TGAGGTAGTAAGTTGTATTGTT | 4415 | 2376 | 1.854568315 |
| ssc-miR-99a | ssc-mir-99a | 5p | AACCCGTAGATCCGATCTTGTG | 1262 | 307 | 4.012618297 |
| ssc-miR-99b | ssc-mir-99b | 5p | CACCCGTAGAACCGACCTTGCG | 45 | 132 | 0.387323944 |
| ssc-miR-novel-chr10_5263 | ssc-mir-novel-chr10_5263 | 3p | CACATGGAGTCGCTGTTACAGCT |  |  |  |
| ssc-miR-novel-chr10_5287 | ssc-mir-novel-chr10_5287 | 3p | ACTAGTGCTGTCAGAGACGCC |  |  |  |
| ssc-miR-novel-chr10_5425 | ssc-mir-novel-chr10_5425 | 3p | TACGAATTTCAGGAATACAGC |  | 3 | 0.769230769 |
| ssc-miR-novel-chr10_5436 | ssc-mir-novel-chr10_5436 | 5p | GAGCTGTGATGAGAATCCTCTGAGC |  |  |  |
| ssc-miR-novel-chr10_5472 | ssc-mir-novel-chr10_5472 | 5p | ACGAGAAAGGAGGAGGG |  |  |  |
| ssc-miR-novel-chr10_5540 | ssc-mir-novel-chr10_5540 | 5p | GCAGGAACTTGTGAGTCTCCT |  | 1 | 0.909090909 |
| ssc-miR-novel-chr10_5541 | ssc-mir-novel-chr10_5541 | 3p | TGGTGGTTTACAAAGTAATTC |  |  |  |
| ssc-miR-novel-chr10_5602 | ssc-mir-novel-chr10_5602 | 5p | TGAGTGTGTGTGTGTGAGTGTGTGT | 63 | 1 | 6.636363636 |
| ssc-miR-novel-chr10_5614 | ssc-mir-novel-chr10_5614 | 5p | GCGGGCCCACGGGGGCC |  | 52 | 0.161290323 |
| ssc-miR-novel-chr10_5682 | ssc-mir-novel-chr10_5682 | 3p | CCGGCCGGGCGCGAGCC | 13 | 3 | 1.769230769 |
| ssc-miR-novel-chr10_5914 | ssc-mir-novel-chr10_5914 | 3p | CACATGGAGTCGCTGTTACAGCT |  |  |  |
| ssc-miR-novel-chr10_5917 | ssc-mir-novel-chr10_5917 | 5p | TTCAGGGTCCAGGATTGCTATAG |  | 1 | 0.909090909 |
| ssc-miR-novel-chr10_6036 | ssc-mir-novel-chr10_6036 | 3p | TACGAATTTCAGGAATACAGC |  | 3 | 0.769230769 |
| ssc-miR-novel-chr10_6047 | ssc-mir-novel-chr10_6047 | 5p | TGGATGGGAGTCGGTGGGCAGC |  |  |  |
| ssc-miR-novel-chr10_6071 | ssc-mir-novel-chr10_6071 | 5p | GTGGATTTTTGGAGTTGGG |  | 2 | 0.833333333 |
| ssc-miR-novel-chr10_6138 | ssc-mir-novel-chr10_6138 | 3p | TGGTGGTTTACAAAGTAATTC |  |  |  |
| ssc-miR-novel-chr10_6327 | ssc-mir-novel-chr10_6327 | 5p | AGTGTGTGGGCGCCGGACGCT |  | 17 | 0.37037037 |
| ssc-miR-novel-chr11_6393 | ssc-mir-novel-chr11_6393 | 3p | CGGGGCCGGGGGTGGGG |  |  |  |
| ssc-miR-novel-chr11_6456 | ssc-mir-novel-chr11_6456 | 5p | CTCGCGGGAAAACTTGTATGTG |  |  |  |
| ssc-miR-novel-chr11_6577 | ssc-mir-novel-chr11_6577 | 3p | CTGAATGGAATTGTCTCAGCCT |  |  |  |
| ssc-miR-novel-chr11_6750 | ssc-mir-novel-chr11_6750 | 5p | CAGGGTCGGGCCTGGTTA | 2 | 547 | 0.021543986 |
| ssc-miR-novel-chr11_6755 | ssc-mir-novel-chr11_6755 | 3p | TGTCCCACCAGAGTCGCCA |  | 7 | 0.588235294 |
| ssc-miR-novel-chr11_6826 | ssc-mir-novel-chr11_6826 | 5p | ACTTTCCCGGGATTTGGAGCG |  |  |  |
| ssc-miR-novel-chr11_6850 | ssc-mir-novel-chr11_6850 | 5p | TCCTGGAGGACGTGCTGTGC | 2 |  | 1.2 |
| ssc-miR-novel-chr11_6942 | ssc-mir-novel-chr11_6942 | 3p | TGACTCACTCTGTTGTGCAGC |  |  |  |
| ssc-miR-novel-chr11_7050 | ssc-mir-novel-chr11_7050 | 3p | AGCGTGGGCTGCGGGCCGCT |  |  |  |
| ssc-miR-novel-chr11_7060 | ssc-mir-novel-chr11_7060 | 3p | CGCGCTTAGGGTTCCCGGCAT |  |  |  |
| ssc-miR-novel-chr11_7116 | ssc-mir-novel-chr11_7116 | 3p | CTGAATGGAATTGTCTCAGCCT |  |  |  |
| ssc-miR-novel-chr11_7251 | ssc-mir-novel-chr11_7251 | 5p | ACATTTAAGGAGGTGCTTGCT |  | 1 | 0.909090909 |
| ssc-miR-novel-chr12_7407 | ssc-mir-novel-chr12_7407 | 3p | CGGCGGCGGCGGCGACT | 23 | 47 | 0.578947368 |
| ssc-miR-novel-chr12_7455 | ssc-mir-novel-chr12_7455 | 3p | TGCTTGGACAGTGCCTGGCCTGC |  | 1 | 0.909090909 |
| ssc-miR-novel-chr12_7490 | ssc-mir-novel-chr12_7490 | 5p | TTTGCAGTAACAGGTGTGAAC |  | 14 | 0.416666667 |
| ssc-miR-novel-chr12_7511 | ssc-mir-novel-chr12_7511 | 3p | CTTTGTCTTAATCTCTGTGGTT |  |  |  |
| ssc-miR-novel-chr12_7711 | ssc-mir-novel-chr12_7711 | 3p | TGCGTCTCACCTGCCTGACAGG |  |  |  |
| ssc-miR-novel-chr12_7741 | ssc-mir-novel-chr12_7741 | 3p | GGAGCGGGCGGGCGGTC | 11 |  | 2.1 |
| ssc-miR-novel-chr12_7775 | ssc-mir-novel-chr12_7775 | 3p | TGAAAGGACGTAAAACAGGCCC |  |  |  |
| ssc-miR-novel-chr12_7779 | ssc-mir-novel-chr12_7779 | 3p | TGAAAGGACGTAAAACAGGCCC |  |  |  |
| ssc-miR-novel-chr12_7811 | ssc-mir-novel-chr12_7811 | 3p | TGCGTCTCACCTGCCTGACAGG |  |  |  |
| ssc-miR-novel-chr12_7898 | ssc-mir-novel-chr12_7898 | 5p | TCCTGATTTGCCGAGGCCTGAGGG | 8 |  | 1.8 |
| ssc-miR-novel-chr12_7955 | ssc-mir-novel-chr12_7955 | 3p | CGGCTGCGAGCAGACGGTC |  |  |  |
| ssc-miR-novel-chr12_7961 | ssc-mir-novel-chr12_7961 | 3p | CAGTGCAATAGTATTGTCAAAG | 1700 | 191 | 8.507462687 |
| ssc-miR-novel-chr12_7963 | ssc-mir-novel-chr12_7963 | 3p | TAGTGCAATATTGCTTATAGGGT | 26 | 43 | 0.679245283 |
| ssc-miR-novel-chr12_7964 | ssc-mir-novel-chr12_7964 | 5p | TAGTGCAATATTGCTTATAGGGT | 26 | 43 | 0.679245283 |
| ssc-miR-novel-chr12_8144 | ssc-mir-novel-chr12_8144 | 5p | GACCATGGCTGTAGACTGTTA | 7 | 1 | 1.545454545 |
| ssc-miR-novel-chr12_8161 | ssc-mir-novel-chr12_8161 | 3p | CAGGCCTGGAGCTCTGCCTGCT | 19 |  | 2.9 |
| ssc-miR-novel-chr12_8220 | ssc-mir-novel-chr12_8220 | 5p | GGTGAGCACTCTGGACT |  | 4 | 0.714285714 |
| ssc-miR-novel-chr12_8221 | ssc-mir-novel-chr12_8221 | 3p | CGGGACCTGGTGCCCCTGTCGC |  |  |  |
| ssc-miR-novel-chr12_8235 | ssc-mir-novel-chr12_8235 | 3p | CACACACAGATCATTTCGTAGA |  |  |  |
| ssc-miR-novel-chr12_8265 | ssc-mir-novel-chr12_8265 | 3p | TTACAGTATTAGTCGCTTTT |  | 51 | 0.163934426 |
| ssc-miR-novel-chr12_8282 | ssc-mir-novel-chr12_8282 | 5p | TATCCCCATGGAGTCTGTTGCC | 2 |  | 1.2 |
| ssc-miR-novel-chr12_8290 | ssc-mir-novel-chr12_8290 | 5p | TATCCCCATGGAGTCTGTTGCC | 2 |  | 1.2 |
| ssc-miR-novel-chr12_8302 | ssc-mir-novel-chr12_8302 | 5p | GGTGAGCACTCTGGACT |  | 4 | 0.714285714 |
| ssc-miR-novel-chr12_8361 | ssc-mir-novel-chr12_8361 | 5p | CAGGGCACCGGCCTCTGCGTGGG |  |  |  |
| ssc-miR-novel-chr12_8369 | ssc-mir-novel-chr12_8369 | 5p | TCAACAAAATCACTGATGCTGGA |  | 3 | 0.769230769 |
| ssc-miR-novel-chr12_8374 | ssc-mir-novel-chr12_8374 | 3p | CAGGGCTTGGGGAGCAGAGAGA | 8 | 1 | 1.636363636 |
| ssc-miR-novel-chr12_8420 | ssc-mir-novel-chr12_8420 | 3p | TGGCTCAGCTCAGCAGGAG |  | 9 | 0.526315789 |
| ssc-miR-novel-chr12_8530 | ssc-mir-novel-chr12_8530 | 3p | TGCTCTGGAGTCAAGTCAGGA |  |  |  |
| ssc-miR-novel-chr12_8533 | ssc-mir-novel-chr12_8533 | 5p | CAGTGAATGGAGCCCTGAGA |  |  |  |
| ssc-miR-novel-chr12_8560 | ssc-mir-novel-chr12_8560 | 3p | TACAGCTTCTTGGAGTTCCCGTGT |  |  |  |
| ssc-miR-novel-chr12_8568 | ssc-mir-novel-chr12_8568 | 3p | CAGGACATGGAGAAAGGC |  |  |  |
| ssc-miR-novel-chr12_8591 | ssc-mir-novel-chr12_8591 | 5p | ACGGGATTGTAAAGGCAGAGCG |  |  |  |
| ssc-miR-novel-chr12_8594 | ssc-mir-novel-chr12_8594 | 3p | ACTGGGAGCAATGGAACGGCGA |  | 1 | 0.909090909 |
| ssc-miR-novel-chr12_8618 | ssc-mir-novel-chr12_8618 | 3p | CACGTGCGTGCTGGTATCTGG |  | 1 | 0.909090909 |
| ssc-miR-novel-chr12_8656 | ssc-mir-novel-chr12_8656 | 5p | TTTACGTCCTTTCACCTAGTTT |  |  |  |
| ssc-miR-novel-chr12_8665 | ssc-mir-novel-chr12_8665 | 5p | TCAGGATGAGACTCAAGGGGC |  |  |  |
| ssc-miR-novel-chr12_8771 | ssc-mir-novel-chr12_8771 | 5p | ACTGGACTTGGAGTCAGAAG | 33 | 12 | 1.954545455 |
| ssc-miR-novel-chr12_8979 | ssc-mir-novel-chr12_8979 | 5p | AGCTGGGCTGCTGTTCTCGAGGT |  |  |  |
| ssc-miR-novel-chr12_8980 | ssc-mir-novel-chr12_8980 | 3p | AGCTGGGCTGCTGTTCTCGAGGT |  |  |  |
| ssc-miR-novel-chr12_8997 | ssc-mir-novel-chr12_8997 | 5p | CACGGGTTCGATCCCTGGTGTGGGC |  |  |  |
| ssc-miR-novel-chr12_9131 | ssc-mir-novel-chr12_9131 | 5p | TGGGGTGGGGGCGTCGGGCC |  |  |  |
| ssc-miR-novel-chr12_9188 | ssc-mir-novel-chr12_9188 | 3p | AGCGCGGCTGGGCCTCCCCGA |  |  |  |
| ssc-miR-novel-chr13_10019 | ssc-mir-novel-chr13_10019 | 3p | GCGACCCATACTTGGTTTCAGA |  |  |  |
| ssc-miR-novel-chr13_10041 | ssc-mir-novel-chr13_10041 | 3p | TTGGAGTTTTGGAGCTGGG |  | 2 | 0.833333333 |
| ssc-miR-novel-chr13_10108 | ssc-mir-novel-chr13_10108 | 5p | AGGAGCAGGAGTCTGGGCTGAGG |  |  |  |
| ssc-miR-novel-chr13_10170 | ssc-mir-novel-chr13_10170 | 5p | AAGCAGGATTTAGACTACAATAT | 10 | 7 | 1.176470588 |
| ssc-miR-novel-chr13_10187 | ssc-mir-novel-chr13_10187 | 3p | AGAGGGCTGTGGGAGAGA |  |  |  |
| ssc-miR-novel-chr13_10484 | ssc-mir-novel-chr13_10484 | 5p | ACAGTGGCTGTGGCTCG | 3 | 2 | 1.083333333 |
| ssc-miR-novel-chr13_10658 | ssc-mir-novel-chr13_10658 | 5p | TCAGTAACAAAGATTCATCCTTG |  |  |  |
| ssc-miR-novel-chr13_10858 | ssc-mir-novel-chr13_10858 | 3p | CCTTCTCTGTAGGCCAGGGGCCCAG |  |  |  |
| ssc-miR-novel-chr13_10861 | ssc-mir-novel-chr13_10861 | 5p | TTCAAGTAACCCAGGATAGGCT | 138 | 1483 | 0.09912927 |
| ssc-miR-novel-chr13_10908 | ssc-mir-novel-chr13_10908 | 3p | GGCCGGGCCCGCCCCCGC | 16 | 4 | 1.857142857 |
| ssc-miR-novel-chr13_10965 | ssc-mir-novel-chr13_10965 | 5p | GCTGTCTCTGTATGAATGTG |  |  |  |
| ssc-miR-novel-chr13_10983 | ssc-mir-novel-chr13_10983 | 5p | TGGGTGAGAGCACAGCAGAACT |  | 1 | 0.909090909 |
| ssc-miR-novel-chr13_11069 | ssc-mir-novel-chr13_11069 | 5p | TCTGGCTGTGGTGTAGA |  |  |  |
| ssc-miR-novel-chr13_11147 | ssc-mir-novel-chr13_11147 | 3p | AATCTGTCCACATATGGTGGTA |  |  |  |
| ssc-miR-novel-chr13_11172 | ssc-mir-novel-chr13_11172 | 3p | CCCTGTCTTTTGCTTCTCCTTT |  |  |  |
| ssc-miR-novel-chr13_11226 | ssc-mir-novel-chr13_11226 | 3p | CTTCCTACCCAGTCCGGC |  |  |  |
| ssc-miR-novel-chr13_11289 | ssc-mir-novel-chr13_11289 | 5p | AGGGTCTAGGTCGCAGATACAGC |  | 3 | 0.769230769 |
| ssc-miR-novel-chr13_11457 | ssc-mir-novel-chr13_11457 | 5p | TCTGGCTTTAATCTCTGTCTTG | 2 | 3 | 0.923076923 |
| ssc-miR-novel-chr13_11481 | ssc-mir-novel-chr13_11481 | 5p | TCTGGCTGTGGTGTAGACC | 11 | 1 | 1.909090909 |
| ssc-miR-novel-chr13_11522 | ssc-mir-novel-chr13_11522 | 3p | CTGGTCTGTGTCTTTGTGAGAGC |  |  |  |
| ssc-miR-novel-chr13_11595 | ssc-mir-novel-chr13_11595 | 5p | AAGCCAGAGTCAGGGGACACTGT |  |  |  |
| ssc-miR-novel-chr13_11601 | ssc-mir-novel-chr13_11601 | 5p | TATCTGCCTGTATATATGCCT |  |  |  |
| ssc-miR-novel-chr13_11602 | ssc-mir-novel-chr13_11602 | 3p | TATCTGCCTGTATATATGCCT |  |  |  |
| ssc-miR-novel-chr13_11720 | ssc-mir-novel-chr13_11720 | 3p | TGGGTGGCAAATGGTGGGTTTGA |  | 1 | 0.909090909 |
| ssc-miR-novel-chr13_11871 | ssc-mir-novel-chr13_11871 | 5p | GCGCAGCACATCATGGTTTA |  |  |  |
| ssc-miR-novel-chr13_11899 | ssc-mir-novel-chr13_11899 | 5p | ATTCTGTTAGAAAAATGCAAGA | 4 | 2 | 1.166666667 |
| ssc-miR-novel-chr13_11944 | ssc-mir-novel-chr13_11944 | 5p | TTTACATTGATTTCATATTGCT |  |  |  |
| ssc-miR-novel-chr13_12020 | ssc-mir-novel-chr13_12020 | 3p | AGACCTGGATTCATCAGCC |  |  |  |
| ssc-miR-novel-chr13_12094 | ssc-mir-novel-chr13_12094 | 3p | GCCTTGGCCTCTGCACCTGGTC |  |  |  |
| ssc-miR-novel-chr13_12101 | ssc-mir-novel-chr13_12101 | 5p | AAGGAGCTGAGGACGGAGAAGGA | 8 | 4 | 1.285714286 |
| ssc-miR-novel-chr13_9205 | ssc-mir-novel-chr13_9205 | 3p | ATTCTTTGCTGGATGGCATT |  |  |  |
| ssc-miR-novel-chr13_9254 | ssc-mir-novel-chr13_9254 | 5p | GAAATGAAGAACCCAGA |  | 1 | 0.909090909 |
| ssc-miR-novel-chr13_9348 | ssc-mir-novel-chr13_9348 | 5p | CTTGGAATTTTGCAGTGTCCA |  | 2 | 0.833333333 |
| ssc-miR-novel-chr13_9358 | ssc-mir-novel-chr13_9358 | 5p | AAGGTGGCTAATGCTTAGTGAGT |  |  |  |
| ssc-miR-novel-chr13_9430 | ssc-mir-novel-chr13_9430 | 5p | TTTGCTCTGCTCCTGCCACAT | 2 |  | 1.2 |
| ssc-miR-novel-chr13_9441 | ssc-mir-novel-chr13_9441 | 3p | GAGCGGGGCGCGCGGTCG | 9 | 2 | 1.583333333 |
| ssc-miR-novel-chr13_9442 | ssc-mir-novel-chr13_9442 | 5p | GAGCGGGGCGCGCGGTCG |  | 2 | 0.833333333 |
| ssc-miR-novel-chr13_9467 | ssc-mir-novel-chr13_9467 | 3p | TGTCCAAGACTGCAGGTCAGT |  |  |  |
| ssc-miR-novel-chr13_9486 | ssc-mir-novel-chr13_9486 | 3p | CCAGAGCACTCAAAAAGATGGC |  |  |  |
| ssc-miR-novel-chr13_9502 | ssc-mir-novel-chr13_9502 | 5p | TCCCACAGGGGGACGTGAGGCAG |  |  |  |
| ssc-miR-novel-chr13_9520 | ssc-mir-novel-chr13_9520 | 5p | TCCCACAGGGGGACGTGAGGCAG |  |  |  |
| ssc-miR-novel-chr13_9841 | ssc-mir-novel-chr13_9841 | 3p | CTGCGTCGGAGGGCGGCCGGAA |  |  |  |
| ssc-miR-novel-chr13_9887 | ssc-mir-novel-chr13_9887 | 3p | CAGGCATGAAGTCAGGTTAG |  |  |  |
| ssc-miR-novel-chr13_9979 | ssc-mir-novel-chr13_9979 | 3p | TTGTCAGTAGTTGCATGCAGGGA |  | 1 | 0.909090909 |
| ssc-miR-novel-chr13_9996 | ssc-mir-novel-chr13_9996 | 5p | AGGCATGGTCAAGAGGAAATC |  |  |  |
| ssc-miR-novel-chr14_12177 | ssc-mir-novel-chr14_12177 | 3p | TTTGTCTGTGGTGTAGGCCCGC | 18 | 5 | 1.866666667 |
| ssc-miR-novel-chr14_12179 | ssc-mir-novel-chr14_12179 | 3p | TTTGTCTGTGGTGTAGGCCCGC | 18 | 5 | 1.866666667 |
| ssc-miR-novel-chr14_12181 | ssc-mir-novel-chr14_12181 | 3p | TTTGTCTGTGGTGTAGGCCCGC | 18 | 5 | 1.866666667 |
| ssc-miR-novel-chr14_12274 | ssc-mir-novel-chr14_12274 | 5p | CAGATGGAGGCGTGGGT |  | 15 | 0.4 |
| ssc-miR-novel-chr14_12484 | ssc-mir-novel-chr14_12484 | 5p | AGCCAGGATTGTGGTGGCAGCCAG |  |  |  |
| ssc-miR-novel-chr14_12513 | ssc-mir-novel-chr14_12513 | 3p | TGGAAGGACACAGGAAGCCT |  |  |  |
| ssc-miR-novel-chr14_12542 | ssc-mir-novel-chr14_12542 | 5p | TTCCAGGGAAGAAAGGAGGAAC |  |  |  |
| ssc-miR-novel-chr14_12589 | ssc-mir-novel-chr14_12589 | 3p | AGCAGGACTGGCAGCTCTGGGCCTC |  |  |  |
| ssc-miR-novel-chr14_12636 | ssc-mir-novel-chr14_12636 | 5p | GTAGTACTTCTTGTTTGGATGCA |  |  |  |
| ssc-miR-novel-chr14_12638 | ssc-mir-novel-chr14_12638 | 5p | GTAGTACTTCTTGTTTGGATGCA |  |  |  |
| ssc-miR-novel-chr14_12801 | ssc-mir-novel-chr14_12801 | 3p | CTCTCTGACAGTCTCTGAAAGC |  |  |  |
| ssc-miR-novel-chr14_12811 | ssc-mir-novel-chr14_12811 | 3p | TGGGCTGTTCTTTTGTCTCTGAG |  |  |  |
| ssc-miR-novel-chr14_12851 | ssc-mir-novel-chr14_12851 | 3p | GACAGATCTCTCCTCCCACAGC | 4 |  | 1.4 |
| ssc-miR-novel-chr14_12856 | ssc-mir-novel-chr14_12856 | 5p | ATCTGGGATCTGCGGCCCCTGC |  |  |  |
| ssc-miR-novel-chr14_12862 | ssc-mir-novel-chr14_12862 | 5p | AAGGAAAGAATTCTAGTGGGG |  |  |  |
| ssc-miR-novel-chr14_12918 | ssc-mir-novel-chr14_12918 | 5p | CAGGAAACACTGGTGGAGG | 91 | 99 | 0.926605505 |
| ssc-miR-novel-chr14_13301 | ssc-mir-novel-chr14_13301 | 3p | CCAAACCAGTTGTGCCTGTAGA |  |  |  |
| ssc-miR-novel-chr14_13321 | ssc-mir-novel-chr14_13321 | 3p | CTGGAACTTGGCTGTGTCTCT |  |  |  |
| ssc-miR-novel-chr14_13411 | ssc-mir-novel-chr14_13411 | 3p | CGGGACGGGTGTCGGGG | 8 | 8 | 1 |
| ssc-miR-novel-chr14_13412 | ssc-mir-novel-chr14_13412 | 5p | CGGGACGGGTGTCGGGG | 8 | 8 | 1 |
| ssc-miR-novel-chr14_13435 | ssc-mir-novel-chr14_13435 | 3p | AGCTCCTCGAGGCCAGA |  |  |  |
| ssc-miR-novel-chr14_13436 | ssc-mir-novel-chr14_13436 | 5p | AGCTCCTCGAGGCCAGA |  |  |  |
| ssc-miR-novel-chr14_13460 | ssc-mir-novel-chr14_13460 | 3p | TCTATCGGGTGTACCTGTCCT | 7 |  | 1.7 |
| ssc-miR-novel-chr14_13655 | ssc-mir-novel-chr14_13655 | 5p | TGAAGGCTGGCCTAGAAATCT |  |  |  |
| ssc-miR-novel-chr14_13668 | ssc-mir-novel-chr14_13668 | 3p | TAAACTGAGGCCTCTGTGGGGC |  |  |  |
| ssc-miR-novel-chr14_13772 | ssc-mir-novel-chr14_13772 | 3p | TTCTCCAAGGACACTCTGTCTCT |  |  |  |
| ssc-miR-novel-chr14_13888 | ssc-mir-novel-chr14_13888 | 3p | CAGTGCAATGATATTGTCAAAGC | 556 | 58 | 8.323529412 |
| ssc-miR-novel-chr14_14550 | ssc-mir-novel-chr14_14550 | 3p | GAGGTTAACGGAGTGACA |  | 1 | 0.909090909 |
| ssc-miR-novel-chr14_14572 | ssc-mir-novel-chr14_14572 | 3p | TGGCACCAGCACTGGCGGTGGC |  | 9 | 0.526315789 |
| ssc-miR-novel-chr14_14582 | ssc-mir-novel-chr14_14582 | 3p | GTGGTTAAGGCTTTGGA |  | 16 | 0.384615385 |
| ssc-miR-novel-chr14_14584 | ssc-mir-novel-chr14_14584 | 3p | CAGACCCTGAGCTGCCTCTAGA |  | 2 | 0.833333333 |
| ssc-miR-novel-chr14_14614 | ssc-mir-novel-chr14_14614 | 3p | AGGAGTGTGGAAGGAGTG |  |  |  |
| ssc-miR-novel-chr15_14859 | ssc-mir-novel-chr15_14859 | 3p | CTGCTCTCCTGTCTCCGCTCAGG |  |  |  |
| ssc-miR-novel-chr15_14871 | ssc-mir-novel-chr15_14871 | 3p | TGCATGACTGTAGAAAACCTGT |  |  |  |
| ssc-miR-novel-chr15_14873 | ssc-mir-novel-chr15_14873 | 3p | TGCATGACTGTAGAAAACCTGT |  |  |  |
| ssc-miR-novel-chr15_14905 | ssc-mir-novel-chr15_14905 | 3p | CTTCTGTGATGGTGAACTGAGA |  |  |  |
| ssc-miR-novel-chr15_14929 | ssc-mir-novel-chr15_14929 | 3p | CTGGCTGGAGAACTTCTACATG |  |  |  |
| ssc-miR-novel-chr15_15025 | ssc-mir-novel-chr15_15025 | 3p | GAGCGTAGTGTTCGCCGGCATA |  |  |  |
| ssc-miR-novel-chr15_15523 | ssc-mir-novel-chr15_15523 | 3p | TTTGTTCGTTCGGCTCGCGTGA | 130 | 58 | 2.058823529 |
| ssc-miR-novel-chr15_15531 | ssc-mir-novel-chr15_15531 | 3p | AGCCCGGCCGTACCCTGCAGG |  |  |  |
| ssc-miR-novel-chr15_15607 | ssc-mir-novel-chr15_15607 | 3p | GAGGCGGCCCCGGCCCGGTAT |  |  |  |
| ssc-miR-novel-chr15_15633 | ssc-mir-novel-chr15_15633 | 3p | TCAGCAGGTCAGGTTCCATAAT |  | 4 | 0.714285714 |
| ssc-miR-novel-chr15_15673 | ssc-mir-novel-chr15_15673 | 3p | GCATTGGGGGTTCAGGGG | 14 | 15 | 0.96 |
| ssc-miR-novel-chr15_15705 | ssc-mir-novel-chr15_15705 | 3p | TCTTGGACCCCTTCTCACAGGA | 3 |  | 1.3 |
| ssc-miR-novel-chr15_15729 | ssc-mir-novel-chr15_15729 | 3p | TCTATCGGGTGTACCTGTCCT | 7 |  | 1.7 |
| ssc-miR-novel-chr15_15788 | ssc-mir-novel-chr15_15788 | 3p | TCCCAGCTGGTCATTAATCCT | 2 | 3 | 0.923076923 |
| ssc-miR-novel-chr15_15866 | ssc-mir-novel-chr15_15866 | 3p | TTGTTCTGGTTGATGGGTGGGA |  |  |  |
| ssc-miR-novel-chr15_16235 | ssc-mir-novel-chr15_16235 | 5p | ATGGGATAATCACACCCCTGGGG |  |  |  |
| ssc-miR-novel-chr15_16275 | ssc-mir-novel-chr15_16275 | 5p | AGCCGGCGGCGGCGGCGA | 2 | 24 | 0.352941176 |
| ssc-miR-novel-chr15_16277 | ssc-mir-novel-chr15_16277 | 5p | AGCCGGCGGCGGCGGCGA | 2 | 24 | 0.352941176 |
| ssc-miR-novel-chr15_16436 | ssc-mir-novel-chr15_16436 | 3p | TTCGCGGCCCGCGCTGGGAGC |  |  |  |
| ssc-miR-novel-chr15_16518 | ssc-mir-novel-chr15_16518 | 3p | GGGCGGACTGTTAAACCG |  |  |  |
| ssc-miR-novel-chr15_16730 | ssc-mir-novel-chr15_16730 | 3p | TCAGCAGGTCAGGTTCCATAAT |  | 4 | 0.714285714 |
| ssc-miR-novel-chr15_16769 | ssc-mir-novel-chr15_16769 | 5p | TGCAGATGATGTGAGAGA |  |  |  |
| ssc-miR-novel-chr16_16851 | ssc-mir-novel-chr16_16851 | 3p | AGAGCTGAGGGCAGAGTCCAGGA |  |  |  |
| ssc-miR-novel-chr16_16917 | ssc-mir-novel-chr16_16917 | 3p | TTGGTGTACACTGGAATAGCT | 18 | 83 | 0.301075269 |
| ssc-miR-novel-chr16_16920 | ssc-mir-novel-chr16_16920 | 5p | ATGGACCACAAACTCAAATT |  |  |  |
| ssc-miR-novel-chr16_16922 | ssc-mir-novel-chr16_16922 | 5p | TGGTCTAGCGGTTAGGA |  | 11 | 0.476190476 |
| ssc-miR-novel-chr16_17112 | ssc-mir-novel-chr16_17112 | 5p | GGTGATGATGACGATGAAGCTGAAA |  | 7 | 0.588235294 |
| ssc-miR-novel-chr16_17181 | ssc-mir-novel-chr16_17181 | 3p | TCTGCCTGTAGGAATGCTGTAGG |  |  |  |
| ssc-miR-novel-chr16_17182 | ssc-mir-novel-chr16_17182 | 5p | TCTGCCTGTAGGAATGCTGTAGG |  |  |  |
| ssc-miR-novel-chr16_17203 | ssc-mir-novel-chr16_17203 | 3p | TGTGGGGCTGATGACACT | 2 |  | 1.2 |
| ssc-miR-novel-chr16_17338 | ssc-mir-novel-chr16_17338 | 5p | CGTGCGTCTCTGGGTGTGATGTCG |  |  |  |
| ssc-miR-novel-chr16_17347 | ssc-mir-novel-chr16_17347 | 3p | TGGAAGGACACAGGAAGCCT |  |  |  |
| ssc-miR-novel-chr16_17372 | ssc-mir-novel-chr16_17372 | 3p | GTCCTCGGCTGGGAGGAGGA |  |  |  |
| ssc-miR-novel-chr16_17391 | ssc-mir-novel-chr16_17391 | 5p | TGAGGTAGTAGGCTGTGTGG | 12 | 23 | 0.666666667 |
| ssc-miR-novel-chr16_17392 | ssc-mir-novel-chr16_17392 | 3p | TGAGGTAGTAGGCTGTGTGG | 193 | 137 | 1.380952381 |
| ssc-miR-novel-chr16_17559 | ssc-mir-novel-chr16_17559 | 5p | TGGCAGTGTATTGTTAGCTGGT | 40 |  | 5 |
| ssc-miR-novel-chr16_17561 | ssc-mir-novel-chr16_17561 | 5p | AGGCAGTGTATTGTTAGCTGGCT | 2 |  | 1.2 |
| ssc-miR-novel-chr16_17571 | ssc-mir-novel-chr16_17571 | 5p | TGGGTGGAAGAAGTGACC |  |  |  |
| ssc-miR-novel-chr16_17722 | ssc-mir-novel-chr16_17722 | 3p | TAGCCTGACGCTGATGATTGT |  |  |  |
| ssc-miR-novel-chr16_17741 | ssc-mir-novel-chr16_17741 | 5p | TAAGAAATAGGTCATTAACAGTA |  |  |  |
| ssc-miR-novel-chr16_17764 | ssc-mir-novel-chr16_17764 | 3p | TGTGGGGCTGATGACACT | 2 |  | 1.2 |
| ssc-miR-novel-chr16_17872 | ssc-mir-novel-chr16_17872 | 3p | AGGGGGAGGCAGGGGGGG |  |  |  |
| ssc-miR-novel-chr16_17883 | ssc-mir-novel-chr16_17883 | 5p | GCCCCCGGTGGCGGGGGGG |  | 1 | 0.909090909 |
| ssc-miR-novel-chr17_17993 | ssc-mir-novel-chr17_17993 | 3p | CGGGGCAGCTCAGTACAGGAC |  |  |  |
| ssc-miR-novel-chr17_17994 | ssc-mir-novel-chr17_17994 | 5p | CGGGGCAGCTCAGTACAGGAC |  |  |  |
| ssc-miR-novel-chr17_18002 | ssc-mir-novel-chr17_18002 | 5p | CGGTGGGGTGCAGTGCTGGACT |  | 2 | 0.833333333 |
| ssc-miR-novel-chr17_18117 | ssc-mir-novel-chr17_18117 | 5p | CTGGGCTGCCTCTGGGT |  | 1 | 0.909090909 |
| ssc-miR-novel-chr17_18122 | ssc-mir-novel-chr17_18122 | 3p | CAAGGCTCCACCTGCGCCCAAG |  |  |  |
| ssc-miR-novel-chr17_18126 | ssc-mir-novel-chr17_18126 | 5p | AGTGGTGAAATGTATTTAGGAC |  |  |  |
| ssc-miR-novel-chr17_18148 | ssc-mir-novel-chr17_18148 | 5p | TACAGTGGCTGTGGCTC | 3 | 2 | 1.083333333 |
| ssc-miR-novel-chr17_18173 | ssc-mir-novel-chr17_18173 | 3p | AATGATGCCCCTTAGAGTTGAGC |  |  |  |
| ssc-miR-novel-chr17_18175 | ssc-mir-novel-chr17_18175 | 3p | AATGATGCCCCTTAGAGTTGAGC |  |  |  |
| ssc-miR-novel-chr17_18195 | ssc-mir-novel-chr17_18195 | 5p | GGGTTAAGGATCTGGTGT |  |  |  |
| ssc-miR-novel-chr17_18444 | ssc-mir-novel-chr17_18444 | 5p | TATGGTCACTGCTGTGACACGGGT |  | 2 | 0.833333333 |
| ssc-miR-novel-chr17_18693 | ssc-mir-novel-chr17_18693 | 5p | AGTGGTGAAATGTATTTAGGAC |  |  |  |
| ssc-miR-novel-chr17_18954 | ssc-mir-novel-chr17_18954 | 3p | GGTTTTGGTCCTCGGCC | 23 | 8 | 1.833333333 |
| ssc-miR-novel-chr17_18970 | ssc-mir-novel-chr17_18970 | 3p | TGTGTCCTCAGTAACCT | 13 |  | 2.3 |
| ssc-miR-novel-chr17_18987 | ssc-mir-novel-chr17_18987 | 5p | AGGGTTGGGCGGAGGCTTT | 49 | 3 | 4.538461538 |
| ssc-miR-novel-chr18_19035 | ssc-mir-novel-chr18_19035 | 3p | GCGGGGGTGGCGGCGGG |  | 3 | 0.769230769 |
| ssc-miR-novel-chr18_19202 | ssc-mir-novel-chr18_19202 | 5p | TTGCCCCGTCGAAGCTGTCGGTG | 2 |  | 1.2 |
| ssc-miR-novel-chr18_19204 | ssc-mir-novel-chr18_19204 | 5p | TTGCCCCGTCGAAGCTGTCGGTG | 2 |  | 1.2 |
| ssc-miR-novel-chr18_19238 | ssc-mir-novel-chr18_19238 | 5p | TTTGGCACTAGCACATTTTTGCT | 17 | 116 | 0.214285714 |
| ssc-miR-novel-chr18_19266 | ssc-mir-novel-chr18_19266 | 5p | TTGTGTCAATATGCGATGATGT |  |  |  |
| ssc-miR-novel-chr18_19287 | ssc-mir-novel-chr18_19287 | 3p | TGGGGTGTGGTGGTGGTGGG |  |  |  |
| ssc-miR-novel-chr18_19390 | ssc-mir-novel-chr18_19390 | 5p | TGATGTCAGGATGAAGCTTGGACT |  |  |  |
| ssc-miR-novel-chr18_19420 | ssc-mir-novel-chr18_19420 | 5p | TTCTGGAAGATGTAGTCTGGA |  |  |  |
| ssc-miR-novel-chr18_19428 | ssc-mir-novel-chr18_19428 | 5p | TGAGCGGACAAGTTTGAGCCCCT |  |  |  |
| ssc-miR-novel-chr18_19466 | ssc-mir-novel-chr18_19466 | 5p | ACGAAGAACATTTGTGTAGCT |  |  |  |
| ssc-miR-novel-chr18_19490 | ssc-mir-novel-chr18_19490 | 3p | GTTCCCTCGTGGCTCAATGGGT |  |  |  |
| ssc-miR-novel-chr18_19647 | ssc-mir-novel-chr18_19647 | 5p | TGAGGGAGGAGTATGTGCTGTG |  |  |  |
| ssc-miR-novel-chr18_19712 | ssc-mir-novel-chr18_19712 | 3p | AACATGGAAGTGGATTGAGG |  |  |  |
| ssc-miR-novel-chr18_19745 | ssc-mir-novel-chr18_19745 | 5p | TTATGGCCCTTCGGTAATTCACT | 6 |  | 1.6 |
| ssc-miR-novel-chr18_19771 | ssc-mir-novel-chr18_19771 | 5p | TGAACAGAGGTCATGAACAGAGC |  | 1 | 0.909090909 |
| ssc-miR-novel-chr18_19783 | ssc-mir-novel-chr18_19783 | 5p | TTGTGTCAATATGCGATGATGT |  |  |  |
| ssc-miR-novel-chr18_19904 | ssc-mir-novel-chr18_19904 | 3p | TTCCCTTTGGGCCCTCAGG |  |  |  |
| ssc-miR-novel-chr18_20001 | ssc-mir-novel-chr18_20001 | 5p | CAAGGAGGAGTCAGATGAGGCTG |  |  |  |
| ssc-miR-novel-chr18_20013 | ssc-mir-novel-chr18_20013 | 5p | TACTGCAAAGTGATTGAGGAGC |  |  |  |
| ssc-miR-novel-chr18_20025 | ssc-mir-novel-chr18_20025 | 3p | GTTCCCTCGTGGCTCAATGGGT |  |  |  |
| ssc-miR-novel-chr1_1003 | ssc-mir-novel-chr1_1003 | 3p | TGGACTAACTGTGGTATTGGGA |  |  |  |
| ssc-miR-novel-chr1_1012 | ssc-mir-novel-chr1_1012 | 5p | TTCAGGTAAAAGACACACACT | 2 | 2 | 1 |
| ssc-miR-novel-chr1_1152 | ssc-mir-novel-chr1_1152 | 5p | AGGCCCTTACATAGTCAGACT |  |  |  |
| ssc-miR-novel-chr1_1175 | ssc-mir-novel-chr1_1175 | 3p | ACTGTGTGTGAGGAAGTC |  |  |  |
| ssc-miR-novel-chr1_1345 | ssc-mir-novel-chr1_1345 | 3p | TAACCCGACATTCAAGGCCTGT |  |  |  |
| ssc-miR-novel-chr1_1497 | ssc-mir-novel-chr1_1497 | 3p | TACCACAGGGCAGAACCACGT |  |  |  |
| ssc-miR-novel-chr1_1514 | ssc-mir-novel-chr1_1514 | 5p | TCTGATTTGGATTCCAGA |  |  |  |
| ssc-miR-novel-chr1_1558 | ssc-mir-novel-chr1_1558 | 5p | CGGGGCGGGCGGTCGCCCGGGGC |  | 80 | 0.111111111 |
| ssc-miR-novel-chr1_1613 | ssc-mir-novel-chr1_1613 | 3p | ATAAAACTTAGTCGCTGCT |  |  |  |
| ssc-miR-novel-chr1_1798 | ssc-mir-novel-chr1_1798 | 5p | TGGAAACACTTCTGCACAAACT | 3 | 1 | 1.181818182 |
| ssc-miR-novel-chr1_1972 | ssc-mir-novel-chr1_1972 | 5p | TTCCCTTTGTCATCCTTTGCCC |  |  |  |
| ssc-miR-novel-chr1_2074 | ssc-mir-novel-chr1_2074 | 5p | TTTAGTGTGATAATGGCGTTTG |  |  |  |
| ssc-miR-novel-chr1_2132 | ssc-mir-novel-chr1_2132 | 5p | TCGTTAGTTCTCTGAGGCTCCGA |  |  |  |
| ssc-miR-novel-chr1_2249 | ssc-mir-novel-chr1_2249 | 3p | AGACCGGGGACCGGAGCGAGCAGA |  | 1 | 0.909090909 |
| ssc-miR-novel-chr1_2311 | ssc-mir-novel-chr1_2311 | 5p | ATTTGGTGGATTGTGGCAGTA |  |  |  |
| ssc-miR-novel-chr1_2360 | ssc-mir-novel-chr1_2360 | 5p | CGGCAGACTGGGACTTGTTGTT | 2 |  | 1.2 |
| ssc-miR-novel-chr1_2748 | ssc-mir-novel-chr1_2748 | 5p | AAAGGAGAGGAAAGAAGG |  |  |  |
| ssc-miR-novel-chr1_2775 | ssc-mir-novel-chr1_2775 | 3p | ACTAGAAATTGGGTAGGACAGA |  |  |  |
| ssc-miR-novel-chr1_2959 | ssc-mir-novel-chr1_2959 | 3p | TGGGGTGGGGCTCTCAACCTCCAGA |  |  |  |
| ssc-miR-novel-chr1_2989 | ssc-mir-novel-chr1_2989 | 3p | GCGCGTCTTTTGGTGCTCTGCAG |  |  |  |
| ssc-miR-novel-chr1_3279 | ssc-mir-novel-chr1_3279 | 5p | CTCTAGAGGTGGATTTGCTGTGCTG |  |  |  |
| ssc-miR-novel-chr1_3280 | ssc-mir-novel-chr1_3280 | 3p | CTCTAGAGGTGGATTTGCTGTGCTG |  |  |  |
| ssc-miR-novel-chr1_3515 | ssc-mir-novel-chr1_3515 | 5p | AAACATTAGAATGTCTGAGTGG |  | 2 | 0.833333333 |
| ssc-miR-novel-chr1_3810 | ssc-mir-novel-chr1_3810 | 3p | ATAAAACTTAGTCGCTGCT |  |  |  |
| ssc-miR-novel-chr1_3836 | ssc-mir-novel-chr1_3836 | 3p | CGCCGGGGGGGCGGGGG |  | 1 | 0.909090909 |
| ssc-miR-novel-chr1_3896 | ssc-mir-novel-chr1_3896 | 3p | CAATTTAGGTTAATGACTGTC |  | 1 | 0.909090909 |
| ssc-miR-novel-chr1_3924 | ssc-mir-novel-chr1_3924 | 3p | TTGCCTGGGACTCTGGAACC |  | 1 | 0.909090909 |
| ssc-miR-novel-chr1_4044 | ssc-mir-novel-chr1_4044 | 3p | ACTGGTTGGATTGGAGACTAGG |  |  |  |
| ssc-miR-novel-chr1_4317 | ssc-mir-novel-chr1_4317 | 5p | CTGCCGTTGGGTCTGGGGTGT |  | 3 | 0.769230769 |
| ssc-miR-novel-chr1_4339 | ssc-mir-novel-chr1_4339 | 5p | TCGTTAGTTCTCTGAGGCTCCGA |  |  |  |
| ssc-miR-novel-chr1_4468 | ssc-mir-novel-chr1_4468 | 3p | AACTGTTCTACCTCTGAGTAGC |  |  |  |
| ssc-miR-novel-chr1_4539 | ssc-mir-novel-chr1_4539 | 5p | AAGGTTACTTGTTAGTTCAGGA |  |  |  |
| ssc-miR-novel-chr1_4584 | ssc-mir-novel-chr1_4584 | 3p | TATGGAGGGTAGACTGTACT |  | 2 | 0.833333333 |
| ssc-miR-novel-chr1_4647 | ssc-mir-novel-chr1_4647 | 5p | CAGAGCTGTTTGTGAACTGCAGGT | 9 | 14 | 0.791666667 |
| ssc-miR-novel-chr1_4720 | ssc-mir-novel-chr1_4720 | 3p | CTGGAGGAAGAGCTCCCTG |  | 1 | 0.909090909 |
| ssc-miR-novel-chr1_4762 | ssc-mir-novel-chr1_4762 | 3p | AGAGTCGGCTGCGCGGAGCC |  |  |  |
| ssc-miR-novel-chr1_4788 | ssc-mir-novel-chr1_4788 | 3p | TAGCTAAGTGCAAATATGTCCAT |  |  |  |
| ssc-miR-novel-chr1_4860 | ssc-mir-novel-chr1_4860 | 3p | ACTAGAAATTGGGTAGGACAGA |  |  |  |
| ssc-miR-novel-chr1_4929 | ssc-mir-novel-chr1_4929 | 3p | AGGCTGGGGGTGGGGCC |  |  |  |
| ssc-miR-novel-chr1_5015 | ssc-mir-novel-chr1_5015 | 5p | ACAGTCAACGGTCGGTGGTTT |  | 8 | 0.555555556 |
| ssc-miR-novel-chr1_5092 | ssc-mir-novel-chr1_5092 | 3p | CAGAAGGGGAGTCGGAGCAGA |  |  |  |
| ssc-miR-novel-chr1_5095 | ssc-mir-novel-chr1_5095 | 5p | TCTGGAGGACGCTAGAGCTGGGC |  |  |  |
| ssc-miR-novel-chr1_5102 | ssc-mir-novel-chr1_5102 | 3p | AGAATTGTGGCTGGACATCTGT |  | 2 | 0.833333333 |
| ssc-miR-novel-chr1_5156 | ssc-mir-novel-chr1_5156 | 3p | TGCAGTTGCCGGGAAGCGGGC |  |  |  |
| ssc-miR-novel-chr1_5195 | ssc-mir-novel-chr1_5195 | 5p | CATTATTACTCACGGTACGAGT |  |  |  |
| ssc-miR-novel-chr1_5196 | ssc-mir-novel-chr1_5196 | 3p | CATTATTACTCACGGTACGAGT |  |  |  |
| ssc-miR-novel-chr1_5208 | ssc-mir-novel-chr1_5208 | 3p | AAGGGTGGGTGGCCACTGTGC |  |  |  |
| ssc-miR-novel-chr1_946 | ssc-mir-novel-chr1_946 | 5p | TCCCTGTGGTCTAGGGGTTAGGA | 2 | 57 | 0.179104478 |
| ssc-miR-novel-chr2_20089 | ssc-mir-novel-chr2_20089 | 5p | CTGGGCGGACGTGGGGT |  |  |  |
| ssc-miR-novel-chr2_20131 | ssc-mir-novel-chr2_20131 | 3p | CCTCTCTTCCCTCACGGTCGCT |  |  |  |
| ssc-miR-novel-chr2_20138 | ssc-mir-novel-chr2_20138 | 5p | GACCTGTGATGTCGTTTCTGAGA |  | 1 | 0.909090909 |
| ssc-miR-novel-chr2_20190 | ssc-mir-novel-chr2_20190 | 5p | CTCGGGCCTGCGCGCGCGAGGA |  | 1 | 0.909090909 |
| ssc-miR-novel-chr2_20212 | ssc-mir-novel-chr2_20212 | 5p | GACTGGAAGTCTCAGGGGGTGT | 2 |  | 1.2 |
| ssc-miR-novel-chr2_20240 | ssc-mir-novel-chr2_20240 | 5p | ATGTGTGGGTCGGCACCGA |  |  |  |
| ssc-miR-novel-chr2_20261 | ssc-mir-novel-chr2_20261 | 3p | GGGCAAGCCTGCGGAGGTGTGG |  | 3 | 0.769230769 |
| ssc-miR-novel-chr2_20299 | ssc-mir-novel-chr2_20299 | 3p | CATTCTCTGGTGGCCTAGCAGG |  | 2 | 0.833333333 |
| ssc-miR-novel-chr2_20300 | ssc-mir-novel-chr2_20300 | 5p | CATTCTCTGGTGGCCTAGCAGG |  | 1 | 0.909090909 |
| ssc-miR-novel-chr2_20315 | ssc-mir-novel-chr2_20315 | 5p | CCACCCTCATCGCTGCCTGTGTGT |  |  |  |
| ssc-miR-novel-chr2_20345 | ssc-mir-novel-chr2_20345 | 3p | CTTTGTGGTCCTGTTCTT |  |  |  |
| ssc-miR-novel-chr2_20448 | ssc-mir-novel-chr2_20448 | 5p | GTGGGGAAGAACTACAAGACAGCT | 289 | 3 | 23 |
| ssc-miR-novel-chr2_20504 | ssc-mir-novel-chr2_20504 | 5p | AGTCTCAGTGTGGTCTTCAGAGA |  |  |  |
| ssc-miR-novel-chr2_20597 | ssc-mir-novel-chr2_20597 | 5p | TCGTTGGACACAGATCACTACACT |  | 3 | 0.769230769 |
| ssc-miR-novel-chr2_20662 | ssc-mir-novel-chr2_20662 | 5p | CAGTTCACAGTTCATAGGAGGATG |  |  |  |
| ssc-miR-novel-chr2_20682 | ssc-mir-novel-chr2_20682 | 5p | ATTCGGGTGCAGGGACTGAGAGC |  |  |  |
| ssc-miR-novel-chr2_20831 | ssc-mir-novel-chr2_20831 | 3p | CCTGGGGGTCTGAGGGGC | 6 |  | 1.6 |
| ssc-miR-novel-chr2_20848 | ssc-mir-novel-chr2_20848 | 5p | TAACCAATGTGCAGACTACTGT |  | 2 | 0.833333333 |
| ssc-miR-novel-chr2_20962 | ssc-mir-novel-chr2_20962 | 5p | TCCTGTGATGATCTCCCTGAGC |  |  |  |
| ssc-miR-novel-chr2_20965 | ssc-mir-novel-chr2_20965 | 3p | TGAGATGAGAGCCCTTGGCCACT | 17 | 1 | 2.454545455 |
| ssc-miR-novel-chr2_21078 | ssc-mir-novel-chr2_21078 | 5p | TAGTTGCTGTGGGAAGTGAGC |  |  |  |
| ssc-miR-novel-chr2_21186 | ssc-mir-novel-chr2_21186 | 5p | TAAAATGCTATTCTGTCACTGT |  | 1 | 0.909090909 |
| ssc-miR-novel-chr2_21261 | ssc-mir-novel-chr2_21261 | 3p | TGACAATGATGTATCCACTGAGC |  | 1 | 0.909090909 |
| ssc-miR-novel-chr2_21343 | ssc-mir-novel-chr2_21343 | 3p | ACTGAAGTGATGGTCTTGGGAC | 2 |  | 1.2 |
| ssc-miR-novel-chr2_21352 | ssc-mir-novel-chr2_21352 | 5p | ATGTTGGACTCAGAACTCTCACTC |  | 19 | 0.344827586 |
| ssc-miR-novel-chr2_21472 | ssc-mir-novel-chr2_21472 | 5p | CGCGTGCGCGTCGGGTC | 4201 | 30 | 105.275 |
| ssc-miR-novel-chr2_21617 | ssc-mir-novel-chr2_21617 | 3p | TGGATTTTTGGAGCTGG | 140 | 362 | 0.403225806 |
| ssc-miR-novel-chr2_21624 | ssc-mir-novel-chr2_21624 | 5p | TGAGATGAAGCACTGTAGCT | 26 | 92 | 0.352941176 |
| ssc-miR-novel-chr2_21667 | ssc-mir-novel-chr2_21667 | 3p | TTCACTGGTGCCCATGGAGGA |  |  |  |
| ssc-miR-novel-chr2_21682 | ssc-mir-novel-chr2_21682 | 3p | AGCAGCGGCGGCGATGGCAGGCT |  |  |  |
| ssc-miR-novel-chr2_21748 | ssc-mir-novel-chr2_21748 | 3p | CCTCTCTTCCCTCACGGTCGCT |  |  |  |
| ssc-miR-novel-chr2_21765 | ssc-mir-novel-chr2_21765 | 5p | AGCACTTACGGATTCTGGGGG |  | 2 | 0.833333333 |
| ssc-miR-novel-chr2_21774 | ssc-mir-novel-chr2_21774 | 3p | TTATCCGGGCTTGTGCTGAAGC | 8 | 1 | 1.636363636 |
| ssc-miR-novel-chr2_21790 | ssc-mir-novel-chr2_21790 | 3p | AGCACTGATGGAGCCTGAGC |  |  |  |
| ssc-miR-novel-chr2_21820 | ssc-mir-novel-chr2_21820 | 3p | TTTGTTGGCTCCTCTGAAGTGA | 928 | 160 | 5.517647059 |
| ssc-miR-novel-chr2_21982 | ssc-mir-novel-chr2_21982 | 3p | TTTCCTCATATTCATTCAGGAGT | 7 |  | 1.7 |
| ssc-miR-novel-chr2_22148 | ssc-mir-novel-chr2_22148 | 3p | TAGCTCCCTGTGCCAGTCCTGA |  | 1 | 0.909090909 |
| ssc-miR-novel-chr2_22149 | ssc-mir-novel-chr2_22149 | 5p | ATGGTTGGGAGGATTCTCAGA |  | 1 | 0.909090909 |
| ssc-miR-novel-chr2_22156 | ssc-mir-novel-chr2_22156 | 3p | TGTGTCTGTGACATCTGCTGGCA | 31 | 3 | 3.153846154 |
| ssc-miR-novel-chr2_22194 | ssc-mir-novel-chr2_22194 | 3p | TAGCTCTGCCTTGTCTCCCCAGC |  |  |  |
| ssc-miR-novel-chr2_22201 | ssc-mir-novel-chr2_22201 | 5p | TACTGTGGTGTCCTGTGTCTCTG |  |  |  |
| ssc-miR-novel-chr2_22202 | ssc-mir-novel-chr2_22202 | 3p | TACTGTGGTGTCCTGTGTCTCTG |  | 1 | 0.909090909 |
| ssc-miR-novel-chr2_22309 | ssc-mir-novel-chr2_22309 | 5p | AGGATACGTCGCAGACGTGGTT |  | 15 | 0.4 |
| ssc-miR-novel-chr2_22316 | ssc-mir-novel-chr2_22316 | 3p | CTGCCTGGACCCCTGCTCACCGC |  |  |  |
| ssc-miR-novel-chr2_22420 | ssc-mir-novel-chr2_22420 | 3p | AGCCTCTCTGCTCCTTCCCAGG |  |  |  |
| ssc-miR-novel-chr2_22433 | ssc-mir-novel-chr2_22433 | 5p | TCAGGGAGGGAACACAGGGAGC |  |  |  |
| ssc-miR-novel-chr2_22438 | ssc-mir-novel-chr2_22438 | 3p | CATGGCCCTCCACCCCTGCAGG |  |  |  |
| ssc-miR-novel-chr2_22453 | ssc-mir-novel-chr2_22453 | 5p | GTCTCGGGCTCTTGCGACCCA |  |  |  |
| ssc-miR-novel-chr2_22508 | ssc-mir-novel-chr2_22508 | 3p | GAGTCAGAGCTGCAGGTTGTGG |  | 1 | 0.909090909 |
| ssc-miR-novel-chr2_22514 | ssc-mir-novel-chr2_22514 | 3p | TCTGTGCTCTGAGAGGGGC |  |  |  |
| ssc-miR-novel-chr2_22525 | ssc-mir-novel-chr2_22525 | 5p | CGTGGGTGTGTGATGGCT |  |  |  |
| ssc-miR-novel-chr2_22565 | ssc-mir-novel-chr2_22565 | 5p | ACATTTAAGGAGGTGCTTGCT |  | 1 | 0.909090909 |
| ssc-miR-novel-chr2_22609 | ssc-mir-novel-chr2_22609 | 5p | GGAAAAAAGAAATGGAGATGG |  |  |  |
| ssc-miR-novel-chr2_22652 | ssc-mir-novel-chr2_22652 | 3p | AGGCTCTGATTGGATTGGGTG |  |  |  |
| ssc-miR-novel-chr2_22879 | ssc-mir-novel-chr2_22879 | 5p | CGCGTGCGCGTCGGGTC | 4201 | 30 | 105.275 |
| ssc-miR-novel-chr2_23068 | ssc-mir-novel-chr2_23068 | 5p | TTCCGGTCATTGTCTGTACGTGG | 3 |  | 1.3 |
| ssc-miR-novel-chr2_23096 | ssc-mir-novel-chr2_23096 | 3p | TTCACTGGTGCCCATGGAGGA |  |  |  |
| ssc-miR-novel-chr3_23111 | ssc-mir-novel-chr3_23111 | 3p | GCAGAGCAGCGGCAGGAGG |  |  |  |
| ssc-miR-novel-chr3_23137 | ssc-mir-novel-chr3_23137 | 3p | CTCTGCCACACCACCCGCCTGC |  |  |  |
| ssc-miR-novel-chr3_23139 | ssc-mir-novel-chr3_23139 | 3p | CTGCGGTGCTGTGTGCTTTGGT |  | 3 | 0.769230769 |
| ssc-miR-novel-chr3_23263 | ssc-mir-novel-chr3_23263 | 3p | AGCTGGTCTGGGAGTTCCCGGGT |  | 3 | 0.769230769 |
| ssc-miR-novel-chr3_23271 | ssc-mir-novel-chr3_23271 | 3p | TAATTTTATGTATAAGCTAGT | 41 | 17 | 1.888888889 |
| ssc-miR-novel-chr3_23293 | ssc-mir-novel-chr3_23293 | 3p | AGTGGGAGCTGTGTTGACTG |  |  |  |
| ssc-miR-novel-chr3_23295 | ssc-mir-novel-chr3_23295 | 3p | AGTGGGAGCTGTGTTGACTG |  |  |  |
| ssc-miR-novel-chr3_23361 | ssc-mir-novel-chr3_23361 | 3p | AGGAAACTCTGGGGGTGG | 9 | 24 | 0.558823529 |
| ssc-miR-novel-chr3_23365 | ssc-mir-novel-chr3_23365 | 3p | CTCCCTGCTCTTCGGCTCAGAGT |  |  |  |
| ssc-miR-novel-chr3_23411 | ssc-mir-novel-chr3_23411 | 3p | ATCTGTCTGTGTCTCTGAGCAG |  |  |  |
| ssc-miR-novel-chr3_23413 | ssc-mir-novel-chr3_23413 | 3p | GTCAGGATGGCCGAGTGGTCTAAGG |  | 33 | 0.23255814 |
| ssc-miR-novel-chr3_23465 | ssc-mir-novel-chr3_23465 | 3p | CAGGGTCGGGCCTGGTTA | 27 |  | 3.7 |
| ssc-miR-novel-chr3_23507 | ssc-mir-novel-chr3_23507 | 3p | TCTGAGACGGACATCTGGTCC |  |  |  |
| ssc-miR-novel-chr3_23547 | ssc-mir-novel-chr3_23547 | 3p | CTGGGCTGTGCCGGGTGC |  | 1 | 0.909090909 |
| ssc-miR-novel-chr3_23548 | ssc-mir-novel-chr3_23548 | 5p | CTGGGCTGTGCCGGGTGC |  | 1 | 0.909090909 |
| ssc-miR-novel-chr3_23558 | ssc-mir-novel-chr3_23558 | 5p | CGGGGCTGGGCGCGCGC |  | 2 | 0.833333333 |
| ssc-miR-novel-chr3_23681 | ssc-mir-novel-chr3_23681 | 3p | CAGACCAGCAGGATGTGGACT | 2 |  | 1.2 |
| ssc-miR-novel-chr3_23694 | ssc-mir-novel-chr3_23694 | 5p | GGGCATCCCTGTAGGAGCT |  |  |  |
| ssc-miR-novel-chr3_23708 | ssc-mir-novel-chr3_23708 | 5p | AACACATGTGTGCTGAGTGGAACT |  | 1 | 0.909090909 |
| ssc-miR-novel-chr3_23955 | ssc-mir-novel-chr3_23955 | 3p | TGTTGTTCTGAAGGTGA |  |  |  |
| ssc-miR-novel-chr3_24036 | ssc-mir-novel-chr3_24036 | 5p | AGGCTGAGGCTGGAGGA |  | 4 | 0.714285714 |
| ssc-miR-novel-chr3_24093 | ssc-mir-novel-chr3_24093 | 3p | GCACCTTTATTCTTCTAACTG |  |  |  |
| ssc-miR-novel-chr3_24096 | ssc-mir-novel-chr3_24096 | 5p | CTCCCGTGCTGATCAGTAGTGGG | 7 |  | 1.7 |
| ssc-miR-novel-chr3_24244 | ssc-mir-novel-chr3_24244 | 5p | TTCCCTTTGTAATCCTATGCAT |  |  |  |
| ssc-miR-novel-chr3_24308 | ssc-mir-novel-chr3_24308 | 5p | CCAGTGATCAGGTTACGATGGATT | 7 | 2 | 1.416666667 |
| ssc-miR-novel-chr3_24388 | ssc-mir-novel-chr3_24388 | 3p | GCAGAGCAGCGGCAGGAGG |  |  |  |
| ssc-miR-novel-chr3_24518 | ssc-mir-novel-chr3_24518 | 5p | CCGGGAACTCCCAGACCAGCTTC |  |  |  |
| ssc-miR-novel-chr3_24548 | ssc-mir-novel-chr3_24548 | 3p | ACATTCAGAGAACTGTAAACGCC | 3 |  | 1.3 |
| ssc-miR-novel-chr3_24599 | ssc-mir-novel-chr3_24599 | 5p | GTAGACCCGGGACTTCGCTG |  |  |  |
| ssc-miR-novel-chr3_24618 | ssc-mir-novel-chr3_24618 | 3p | CTGCCCACCTGGGTGAGAAGGA |  | 4 | 0.714285714 |
| ssc-miR-novel-chr3_24739 | ssc-mir-novel-chr3_24739 | 5p | TGCAGGACCTCAGAGCAGCT |  |  |  |
| ssc-miR-novel-chr3_24773 | ssc-mir-novel-chr3_24773 | 5p | AGGAGTGGTTGCTGGCAGTGTGC |  |  |  |
| ssc-miR-novel-chr3_24811 | ssc-mir-novel-chr3_24811 | 5p | TGGCTCTGCGAGGTCGGCTCA | 87 | 108 | 0.822033898 |
| ssc-miR-novel-chr3_24812 | ssc-mir-novel-chr3_24812 | 3p | TGGCTCTGCGAGGTCGGCTCA | 87 | 108 | 0.822033898 |
| ssc-miR-novel-chr3_24814 | ssc-mir-novel-chr3_24814 | 3p | CGAGCGGGCCCGGACGCTGCTGGC |  |  |  |
| ssc-miR-novel-chr3_24923 | ssc-mir-novel-chr3_24923 | 5p | GGGCATCCCTGTAGGAGCT |  |  |  |
| ssc-miR-novel-chr3_24929 | ssc-mir-novel-chr3_24929 | 5p | ACACTCAGCATGCACATGGAGGCT |  |  |  |
| ssc-miR-novel-chr3_24974 | ssc-mir-novel-chr3_24974 | 3p | CTGGGAGCAGTTGCCGGATGTG |  | 1 | 0.909090909 |
| ssc-miR-novel-chr3_25028 | ssc-mir-novel-chr3_25028 | 3p | TGTGGCTGTGGCCTGCAACT | 24 | 11 | 1.619047619 |
| ssc-miR-novel-chr3_25115 | ssc-mir-novel-chr3_25115 | 5p | TGGTTGGAGGAGCTGAGAGCGC |  |  |  |
| ssc-miR-novel-chr3_25160 | ssc-mir-novel-chr3_25160 | 3p | TAGTGCAGGTTGGATCCCTAGC |  | 1 | 0.909090909 |
| ssc-miR-novel-chr3_25222 | ssc-mir-novel-chr3_25222 | 5p | GCGTCACCAGCCGAGCCGG |  |  |  |
| ssc-miR-novel-chr3_25240 | ssc-mir-novel-chr3_25240 | 3p | TTCACAGGGGCTCAGTTC |  |  |  |
| ssc-miR-novel-chr3_25266 | ssc-mir-novel-chr3_25266 | 3p | AAGAGGGGCTTGGAGCTGGGCCT |  |  |  |
| ssc-miR-novel-chr3_25267 | ssc-mir-novel-chr3_25267 | 5p | AGATTCAGTCAACCGCGGATC |  |  |  |
| ssc-miR-novel-chr3_25338 | ssc-mir-novel-chr3_25338 | 3p | GATATGAGAGTGTTGGTCCTGA | 4 |  | 1.4 |
| ssc-miR-novel-chr3_25350 | ssc-mir-novel-chr3_25350 | 3p | AGTGGCTGTGGCTCGACCCCT | 9 |  | 1.9 |
| ssc-miR-novel-chr3_25488 | ssc-mir-novel-chr3_25488 | 3p | CCCGGGGCTGCAGAAAGA |  |  |  |
| ssc-miR-novel-chr3_25504 | ssc-mir-novel-chr3_25504 | 3p | TTTCGGACCACAGCCTGGGCATC |  |  |  |
| ssc-miR-novel-chr3_25572 | ssc-mir-novel-chr3_25572 | 3p | CTGTCCTGGGCCTGTCAGAGTCTCC |  |  |  |
| ssc-miR-novel-chr4_25596 | ssc-mir-novel-chr4_25596 | 5p | GTGAAGGGTGCAGGTTCGGG |  |  |  |
| ssc-miR-novel-chr4_25600 | ssc-mir-novel-chr4_25600 | 5p | GCGCCGCGCTGGGAGCCCTGCGGA | 3 |  | 1.3 |
| ssc-miR-novel-chr4_25612 | ssc-mir-novel-chr4_25612 | 5p | CCCACGGTCCACCACTTTGCTGT |  | 2 | 0.833333333 |
| ssc-miR-novel-chr4_25615 | ssc-mir-novel-chr4_25615 | 3p | CGTTGTGATGAAGTGCTGAGG |  | 2 | 0.833333333 |
| ssc-miR-novel-chr4_25671 | ssc-mir-novel-chr4_25671 | 3p | GGGTCGGGGCGGGGCGG |  | 1 | 0.909090909 |
| ssc-miR-novel-chr4_25720 | ssc-mir-novel-chr4_25720 | 5p | AGGAGAAAGCAGACGGGCTGCA | 12 | 7 | 1.294117647 |
| ssc-miR-novel-chr4_25914 | ssc-mir-novel-chr4_25914 | 5p | TATACCTCAGTTTTATCAGGTG |  |  |  |
| ssc-miR-novel-chr4_25916 | ssc-mir-novel-chr4_25916 | 5p | TTTGATAAGCTGACATGGGACA |  |  |  |
| ssc-miR-novel-chr4_26000 | ssc-mir-novel-chr4_26000 | 5p | TTCCTGAGTCGGACTGGGCTGC |  |  |  |
| ssc-miR-novel-chr4_26125 | ssc-mir-novel-chr4_26125 | 3p | ATGGATTGTCTTCTGGGTACT |  |  |  |
| ssc-miR-novel-chr4_26129 | ssc-mir-novel-chr4_26129 | 3p | TCAAGGTCCGCTGTGAACACGG |  |  |  |
| ssc-miR-novel-chr4_26236 | ssc-mir-novel-chr4_26236 | 5p | CTAGAGTGGATTCTGCA |  |  |  |
| ssc-miR-novel-chr4_26332 | ssc-mir-novel-chr4_26332 | 5p | GTCACCTGGCAGGTACCTCTTT |  |  |  |
| ssc-miR-novel-chr4_26365 | ssc-mir-novel-chr4_26365 | 3p | TCATGAGAAAGTGCCTGGAAC |  |  |  |
| ssc-miR-novel-chr4_26369 | ssc-mir-novel-chr4_26369 | 3p | CGGACAGCTCGAAAGGGG |  |  |  |
| ssc-miR-novel-chr4_26379 | ssc-mir-novel-chr4_26379 | 3p | TCGGCGCCCCACCCTCTCTAGC |  |  |  |
| ssc-miR-novel-chr4_26443 | ssc-mir-novel-chr4_26443 | 3p | ACAGGACCCTACCCATCAAGA |  | 2 | 0.833333333 |
| ssc-miR-novel-chr4_26606 | ssc-mir-novel-chr4_26606 | 5p | TGGGGTTCAAAAGGACTCAAGA |  | 1 | 0.909090909 |
| ssc-miR-novel-chr4_26620 | ssc-mir-novel-chr4_26620 | 5p | TGGGGTTCAAAAGGACTCAAGA |  | 1 | 0.909090909 |
| ssc-miR-novel-chr4_26624 | ssc-mir-novel-chr4_26624 | 5p | TGGGGTTCAAAAGGACTCAAGA |  | 1 | 0.909090909 |
| ssc-miR-novel-chr4_26691 | ssc-mir-novel-chr4_26691 | 3p | GCCAGCCGGTGTTCATGCCCCATA |  |  |  |
| ssc-miR-novel-chr4_26743 | ssc-mir-novel-chr4_26743 | 3p | TGAGAGGAAGCACTGTAGGA |  |  |  |
| ssc-miR-novel-chr4_26821 | ssc-mir-novel-chr4_26821 | 3p | TCAGAAGTGGGTCCAGGAATCT |  |  |  |
| ssc-miR-novel-chr4_26845 | ssc-mir-novel-chr4_26845 | 3p | TCTTCCCAGGCTCTGTCTGAAC |  |  |  |
| ssc-miR-novel-chr4_26898 | ssc-mir-novel-chr4_26898 | 5p | AGTCCACTCGTCTCACTGAGA |  |  |  |
| ssc-miR-novel-chr4_26969 | ssc-mir-novel-chr4_26969 | 5p | CGGGGTGTGGGGAGGGC | 2 | 58 | 0.176470588 |
| ssc-miR-novel-chr4_27089 | ssc-mir-novel-chr4_27089 | 5p | CCTCTGGGCCACAGGAGAACT |  | 6 | 0.625 |
| ssc-miR-novel-chr4_27349 | ssc-mir-novel-chr4_27349 | 5p | TCACCTGTTGGATATTCT |  |  |  |
| ssc-miR-novel-chr4_27402 | ssc-mir-novel-chr4_27402 | 3p | ATGGATTGTCTTCTGGGTACT |  |  |  |
| ssc-miR-novel-chr4_27454 | ssc-mir-novel-chr4_27454 | 3p | CAGGACCAGGTGAGCGCCAGC | 3 |  | 1.3 |
| ssc-miR-novel-chr4_27574 | ssc-mir-novel-chr4_27574 | 3p | TCCCAAGACTCCTTTGATCCCT | 2 |  | 1.2 |
| ssc-miR-novel-chr4_27701 | ssc-mir-novel-chr4_27701 | 3p | TGTTGTCCTCCGAATCTGAAT |  |  |  |
| ssc-miR-novel-chr4_27704 | ssc-mir-novel-chr4_27704 | 3p | TCTCTGACTCTCTGACCTCCCAGG |  |  |  |
| ssc-miR-novel-chr4_27733 | ssc-mir-novel-chr4_27733 | 5p | CTGTGTGGTGTAGGGAGAAGCT |  |  |  |
| ssc-miR-novel-chr4_27870 | ssc-mir-novel-chr4_27870 | 5p | CACTCTGGACTCTGAATC | 3 | 11 | 0.619047619 |
| ssc-miR-novel-chr4_27873 | ssc-mir-novel-chr4_27873 | 5p | TGGGGTTCCGAAGGACTAAGA |  |  |  |
| ssc-miR-novel-chr4_27885 | ssc-mir-novel-chr4_27885 | 5p | TGGGGTTCCGAAGGACTAAGA |  |  |  |
| ssc-miR-novel-chr4_27887 | ssc-mir-novel-chr4_27887 | 5p | TGGGGTTCCGAAGGACTAAGA |  |  |  |
| ssc-miR-novel-chr4_27893 | ssc-mir-novel-chr4_27893 | 5p | TGAGAACTGAATTCGATGGGA |  | 7 | 0.588235294 |
| ssc-miR-novel-chr4_27928 | ssc-mir-novel-chr4_27928 | 3p | TGAGACACAGGGCGGGCTGTGCT |  | 1 | 0.909090909 |
| ssc-miR-novel-chr4_27930 | ssc-mir-novel-chr4_27930 | 3p | TATATATATATGATTCACGATG |  |  |  |
| ssc-miR-novel-chr4_27970 | ssc-mir-novel-chr4_27970 | 3p | CAGTCGGGACGCTTCCTTCTT |  |  |  |
| ssc-miR-novel-chr4_28073 | ssc-mir-novel-chr4_28073 | 5p | ACGGAGGCCTCTTACCAGCA |  |  |  |
| ssc-miR-novel-chr4_28122 | ssc-mir-novel-chr4_28122 | 3p | CGGCTCTGGGTCTGTGGGGAGC | 18 | 19 | 0.965517241 |
| ssc-miR-novel-chr5_28279 | ssc-mir-novel-chr5_28279 | 3p | ATGCGGAACCTGCGGATACGG | 61 | 177 | 0.379679144 |
| ssc-miR-novel-chr5_28309 | ssc-mir-novel-chr5_28309 | 3p | TCAGTGGGCACCCAGCCGGAGT |  |  |  |
| ssc-miR-novel-chr5_28311 | ssc-mir-novel-chr5_28311 | 3p | TCAGTGGGCACCCAGCCGGAGT |  |  |  |
| ssc-miR-novel-chr5_28315 | ssc-mir-novel-chr5_28315 | 3p | CTCAGACCTCAGGCTTGGAGCC |  |  |  |
| ssc-miR-novel-chr5_28386 | ssc-mir-novel-chr5_28386 | 5p | TGGGACTTATAGGTGAACCAGA |  |  |  |
| ssc-miR-novel-chr5_28455 | ssc-mir-novel-chr5_28455 | 3p | TGGGAAGTGTCCAGGTTGGGA |  |  |  |
| ssc-miR-novel-chr5_28480 | ssc-mir-novel-chr5_28480 | 5p | TTAGGAGAGGAGCTGAGAAAGGG |  |  |  |
| ssc-miR-novel-chr5_28483 | ssc-mir-novel-chr5_28483 | 3p | TGATGTTTATCCGAATCCTCAGA |  | 1 | 0.909090909 |
| ssc-miR-novel-chr5_28488 | ssc-mir-novel-chr5_28488 | 5p | TTAGGAGAGGAGCTGAGAAAGGG |  |  |  |
| ssc-miR-novel-chr5_28715 | ssc-mir-novel-chr5_28715 | 3p | TGGTGGCAGAGCCAGGTAA |  |  |  |
| ssc-miR-novel-chr5_28736 | ssc-mir-novel-chr5_28736 | 5p | AAGGATGGGAAGGAGGGG |  |  |  |
| ssc-miR-novel-chr5_28811 | ssc-mir-novel-chr5_28811 | 3p | CAACACTGTGCTGGAAGATGGA | 3 | 5 | 0.866666667 |
| ssc-miR-novel-chr5_28846 | ssc-mir-novel-chr5_28846 | 5p | TGGGACTGAAGGCCGCGGCCTCC |  |  |  |
| ssc-miR-novel-chr5_28892 | ssc-mir-novel-chr5_28892 | 5p | CGCAGGGCTCGGGTCGGCTGCCT | 4 |  | 1.4 |
| ssc-miR-novel-chr5_29091 | ssc-mir-novel-chr5_29091 | 3p | TGCAAGGTCGGACCGCTGTGACC |  |  |  |
| ssc-miR-novel-chr5_29097 | ssc-mir-novel-chr5_29097 | 3p | TGCAAGGTCGGACCGCTGTGACC |  |  |  |
| ssc-miR-novel-chr5_29128 | ssc-mir-novel-chr5_29128 | 3p | TGGCTCTGCGAGGTCGGCT | 103 | 27 | 3.054054054 |
| ssc-miR-novel-chr5_29297 | ssc-mir-novel-chr5_29297 | 5p | GCGGGGGTGGCGGCGGG |  | 34 | 0.227272727 |
| ssc-miR-novel-chr5_29298 | ssc-mir-novel-chr5_29298 | 3p | GCGGGGGTGGCGGCGGG |  | 3 | 0.769230769 |
| ssc-miR-novel-chr5_29391 | ssc-mir-novel-chr5_29391 | 5p | TGCGTTCATTCTGTTTGGCCT |  |  |  |
| ssc-miR-novel-chr5_29426 | ssc-mir-novel-chr5_29426 | 3p | TGATGTTTATCCGAATCCTCAGA |  | 1 | 0.909090909 |
| ssc-miR-novel-chr5_29480 | ssc-mir-novel-chr5_29480 | 3p | TCTGAGATGTGACCTGGGCAT | 29 | 7 | 2.294117647 |
| ssc-miR-novel-chr5_29487 | ssc-mir-novel-chr5_29487 | 5p | GACTTAATGGCTGGCTGGGAGG |  | 2 | 0.833333333 |
| ssc-miR-novel-chr5_29564 | ssc-mir-novel-chr5_29564 | 3p | CAGTGTGGGACCTTGGGCCTCC |  |  |  |
| ssc-miR-novel-chr5_29627 | ssc-mir-novel-chr5_29627 | 5p | TCTCCAGTGAGACAGTCTCT | 6 |  | 1.6 |
| ssc-miR-novel-chr5_29674 | ssc-mir-novel-chr5_29674 | 3p | TAACACTGTCTGGTAAAGATG | 131 | 354 | 0.387362637 |
| ssc-miR-novel-chr5_29676 | ssc-mir-novel-chr5_29676 | 3p | TAATACTGCCGGGTAATGATGGA |  | 199 | 0.04784689 |
| ssc-miR-novel-chr5_29710 | ssc-mir-novel-chr5_29710 | 3p | TGTCGATGATGGCAGTGCTGAGG |  |  |  |
| ssc-miR-novel-chr5_29774 | ssc-mir-novel-chr5_29774 | 3p | TCGGCTGCAGACACCACGCC |  |  |  |
| ssc-miR-novel-chr5_29781 | ssc-mir-novel-chr5_29781 | 5p | TAAAGTTATATAAGGGTTTTTG | 3 |  | 1.3 |
| ssc-miR-novel-chr5_29793 | ssc-mir-novel-chr5_29793 | 5p | AAATGAAAAGGATTGGTTTCT |  |  |  |
| ssc-miR-novel-chr5_29857 | ssc-mir-novel-chr5_29857 | 5p | GCAGCAGGTCTCCAAGGGG | 2 | 736 | 0.016085791 |
| ssc-miR-novel-chr5_29868 | ssc-mir-novel-chr5_29868 | 3p | TCTCAGGGATGGTGGAAATAGCCT |  |  |  |
| ssc-miR-novel-chr5_29905 | ssc-mir-novel-chr5_29905 | 5p | ACTCTAGCTGCCAAAGGCGCT |  | 1 | 0.909090909 |
| ssc-miR-novel-chr5_30018 | ssc-mir-novel-chr5_30018 | 3p | CAAGGCCACTGACTGAAGAGCAGA |  |  |  |
| ssc-miR-novel-chr5_30028 | ssc-mir-novel-chr5_30028 | 3p | TGACTCCCGGCCTGCTGGCAGG |  |  |  |
| ssc-miR-novel-chr6_30041 | ssc-mir-novel-chr6_30041 | 3p | GGCTGGGAGCCTGGGCTTCGTGG |  |  |  |
| ssc-miR-novel-chr6_30050 | ssc-mir-novel-chr6_30050 | 5p | TGAGGGCCTCGCCAGCCCCCGGC |  |  |  |
| ssc-miR-novel-chr6_30116 | ssc-mir-novel-chr6_30116 | 3p | CACGAGAAACCGCGTCCCGCCAGG |  |  |  |
| ssc-miR-novel-chr6_30192 | ssc-mir-novel-chr6_30192 | 5p | TCCTCTGGAGGCTCGAGAAGA |  |  |  |
| ssc-miR-novel-chr6_30243 | ssc-mir-novel-chr6_30243 | 5p | AGGAGGAAGAAGAAACTG |  |  |  |
| ssc-miR-novel-chr6_30244 | ssc-mir-novel-chr6_30244 | 5p | AGGAGGAAGAAGAAACTG |  |  |  |
| ssc-miR-novel-chr6_30278 | ssc-mir-novel-chr6_30278 | 5p | AGGAAAGTGTGGTGGAGG |  |  |  |
| ssc-miR-novel-chr6_30281 | ssc-mir-novel-chr6_30281 | 3p | ATCCGGGAGCTGGGAGCC |  |  |  |
| ssc-miR-novel-chr6_30340 | ssc-mir-novel-chr6_30340 | 5p | TCCCGGGGTGTGGGGAGG | 7 | 7 | 1 |
| ssc-miR-novel-chr6_30650 | ssc-mir-novel-chr6_30650 | 5p | ACTCAAACTGTGGGGGCACTT | 7 |  | 1.7 |
| ssc-miR-novel-chr6_30675 | ssc-mir-novel-chr6_30675 | 3p | GACTCCTGCCCCTCTCCCACAGG |  |  |  |
| ssc-miR-novel-chr6_30729 | ssc-mir-novel-chr6_30729 | 3p | TAATACTGCCTGGTAATGATGA | 6 | 1317 | 0.012057272 |
| ssc-miR-novel-chr6_30750 | ssc-mir-novel-chr6_30750 | 5p | CGGGGAGGCTGTGCAGCGCGGC | 2 |  | 1.2 |
| ssc-miR-novel-chr6_30792 | ssc-mir-novel-chr6_30792 | 5p | GGGGCCGGGGGTGGGGCC |  |  |  |
| ssc-miR-novel-chr6_30857 | ssc-mir-novel-chr6_30857 | 3p | CATGGATGTGGTGATGTGG | 286 | 530 | 0.548148148 |
| ssc-miR-novel-chr6_30884 | ssc-mir-novel-chr6_30884 | 5p | TAGCAGGAGGAGGACTCTG |  |  |  |
| ssc-miR-novel-chr6_30922 | ssc-mir-novel-chr6_30922 | 5p | ACCAACGTGGATACCCCGGG |  |  |  |
| ssc-miR-novel-chr6_30930 | ssc-mir-novel-chr6_30930 | 5p | TTGCTGTGATGACAAAT | 7 | 9 | 0.894736842 |
| ssc-miR-novel-chr6_30947 | ssc-mir-novel-chr6_30947 | 3p | CGGGCGGGAGCGGCCGGG | 2 | 4 | 0.857142857 |
| ssc-miR-novel-chr6_31079 | ssc-mir-novel-chr6_31079 | 3p | TGAGATGAAGCTCTGTGTCT |  |  |  |
| ssc-miR-novel-chr6_31216 | ssc-mir-novel-chr6_31216 | 5p | GTTTTGGTGGGGGCCGGGG |  | 51 | 0.163934426 |
| ssc-miR-novel-chr6_31226 | ssc-mir-novel-chr6_31226 | 5p | CAAGTCTTTGGGTTCCAG | 4 | 7 | 0.823529412 |
| ssc-miR-novel-chr6_31268 | ssc-mir-novel-chr6_31268 | 5p | TCATGATATAGAGGTAAATAGT | 3 | 1 | 1.181818182 |
| ssc-miR-novel-chr6_31382 | ssc-mir-novel-chr6_31382 | 5p | CTATGTGCCTGAGAACTT |  |  |  |
| ssc-miR-novel-chr6_31392 | ssc-mir-novel-chr6_31392 | 5p | TCTCAGGATGAGGTAAGATTGCT |  |  |  |
| ssc-miR-novel-chr6_31419 | ssc-mir-novel-chr6_31419 | 3p | TGCTGTGATGGGGGCTCTGAGA |  |  |  |
| ssc-miR-novel-chr6_31436 | ssc-mir-novel-chr6_31436 | 5p | CGCTTCGGCGGCGGGGG | 118 | 16 | 4.923076923 |
| ssc-miR-novel-chr6_31474 | ssc-mir-novel-chr6_31474 | 5p | AGGTGGGATCCCGAGGC | 8 | 150 | 0.1125 |
| ssc-miR-novel-chr6_31487 | ssc-mir-novel-chr6_31487 | 3p | CAAGTGCTAATGTTGGGA |  |  |  |
| ssc-miR-novel-chr6_31513 | ssc-mir-novel-chr6_31513 | 5p | CTCCCCCCCTTCCCGGG |  |  |  |
| ssc-miR-novel-chr6_31604 | ssc-mir-novel-chr6_31604 | 3p | TGTGTGTGGGCGCCGGACGCC | 11 | 25 | 0.6 |
| ssc-miR-novel-chr6_31692 | ssc-mir-novel-chr6_31692 | 3p | TCTGGCTGTGGTGTAGACCGTC | 65 | 3 | 5.769230769 |
| ssc-miR-novel-chr6_31694 | ssc-mir-novel-chr6_31694 | 3p | TTGCAGTGTGCTGAAACCTCGGC |  | 2 | 0.833333333 |
| ssc-miR-novel-chr6_31755 | ssc-mir-novel-chr6_31755 | 5p | TCCTCTGGAGGCTCGAGAAGA |  |  |  |
| ssc-miR-novel-chr6_31759 | ssc-mir-novel-chr6_31759 | 5p | TGGCTTGTACTCTAGGTG |  |  |  |
| ssc-miR-novel-chr6_31814 | ssc-mir-novel-chr6_31814 | 3p | TGGGTGGAGGAAAGCGG |  |  |  |
| ssc-miR-novel-chr6_31822 | ssc-mir-novel-chr6_31822 | 3p | GAGTTCTGGGCTGCAGTG | 7 | 2 | 1.416666667 |
| ssc-miR-novel-chr6_31936 | ssc-mir-novel-chr6_31936 | 3p | ACTGCTGTGCTTTCTGCCTGC |  |  |  |
| ssc-miR-novel-chr6_32013 | ssc-mir-novel-chr6_32013 | 5p | CGGAGTGGGTCGGTTTAAGG |  |  |  |
| ssc-miR-novel-chr6_32075 | ssc-mir-novel-chr6_32075 | 5p | ATGATGGAGGAGCGAGCGGCCG |  |  |  |
| ssc-miR-novel-chr6_32122 | ssc-mir-novel-chr6_32122 | 3p | TTCGCCGCGCAGCCTGCTGGGA |  |  |  |
| ssc-miR-novel-chr6_32142 | ssc-mir-novel-chr6_32142 | 3p | GACACACACAGCGCTCCTGGCC | 4 |  | 1.4 |
| ssc-miR-novel-chr6_32152 | ssc-mir-novel-chr6_32152 | 3p | GACGGGCAGTGTGCTAGGATCC |  |  |  |
| ssc-miR-novel-chr6_32276 | ssc-mir-novel-chr6_32276 | 3p | CTGGGCCCGGGTGGAGC |  | 28 | 0.263157895 |
| ssc-miR-novel-chr6_32385 | ssc-mir-novel-chr6_32385 | 5p | TTCCCTTTGTCATCCTTTGCCC |  |  |  |
| ssc-miR-novel-chr6_32446 | ssc-mir-novel-chr6_32446 | 3p | ACTAGAGCGATTGGAAGG |  |  |  |
| ssc-miR-novel-chr6_32462 | ssc-mir-novel-chr6_32462 | 3p | TTGGCATGAATGTCGCGCTGG |  |  |  |
| ssc-miR-novel-chr6_32491 | ssc-mir-novel-chr6_32491 | 5p | CAAAGCGCCAGAGAGGCGGGGA |  |  |  |
| ssc-miR-novel-chr6_32492 | ssc-mir-novel-chr6_32492 | 3p | CAAAGCGCCAGAGAGGCGGGGA |  |  |  |
| ssc-miR-novel-chr6_32552 | ssc-mir-novel-chr6_32552 | 3p | TTGGACTGGAGGTGAGGC |  |  |  |
| ssc-miR-novel-chr6_32557 | ssc-mir-novel-chr6_32557 | 5p | AGCTTATCAGACTGGTGTAG |  |  |  |
| ssc-miR-novel-chr6_32953 | ssc-mir-novel-chr6_32953 | 5p | AAAGGGACACAGGAGGCT |  |  |  |
| ssc-miR-novel-chr7_33108 | ssc-mir-novel-chr7_33108 | 5p | TCTGGTCCAGACACTGTGGAGC |  |  |  |
| ssc-miR-novel-chr7_33374 | ssc-mir-novel-chr7_33374 | 5p | CTTCCAGCAAGGAGTCTCAGA |  |  |  |
| ssc-miR-novel-chr7_33433 | ssc-mir-novel-chr7_33433 | 3p | TCGAGAATTGCGTTTGGACAAT |  |  |  |
| ssc-miR-novel-chr7_33466 | ssc-mir-novel-chr7_33466 | 5p | TGCAGGTGGAGCCCAGGGGA |  |  |  |
| ssc-miR-novel-chr7_33502 | ssc-mir-novel-chr7_33502 | 5p | TGTCATGCTGGGGAGTGTAGTGA |  | 1 | 0.909090909 |
| ssc-miR-novel-chr7_33539 | ssc-mir-novel-chr7_33539 | 3p | TTTTTTGCTGGAACATTTCTG |  |  |  |
| ssc-miR-novel-chr7_33544 | ssc-mir-novel-chr7_33544 | 5p | TAGGACACAAAATGTAGGAAGGGC |  |  |  |
| ssc-miR-novel-chr7_33656 | ssc-mir-novel-chr7_33656 | 3p | AACGGAAACAATCCAAAACTGT |  |  |  |
| ssc-miR-novel-chr7_33696 | ssc-mir-novel-chr7_33696 | 5p | TCTGGTTCTGTGACCCTGCCT | 7 |  | 1.7 |
| ssc-miR-novel-chr7_33739 | ssc-mir-novel-chr7_33739 | 3p | ATCTGAAAGTACTGGGGGCCT |  |  |  |
| ssc-miR-novel-chr7_33821 | ssc-mir-novel-chr7_33821 | 3p | CAAAACAGAGAATCAAGACTGA | 4 |  | 1.4 |
| ssc-miR-novel-chr7_33839 | ssc-mir-novel-chr7_33839 | 3p | CCGCCTCCTCTCGCCGCC | 2 | 1 | 1.090909091 |
| ssc-miR-novel-chr7_33933 | ssc-mir-novel-chr7_33933 | 3p | AGTAGATTCATGGATACTCT |  |  |  |
| ssc-miR-novel-chr7_33975 | ssc-mir-novel-chr7_33975 | 3p | TGCATCTGTGGCGTAGGCCAGT | 37 | 105 | 0.408695652 |
| ssc-miR-novel-chr7_34009 | ssc-mir-novel-chr7_34009 | 3p | TCCAGGGTGCTCGCCCCAGC |  |  |  |
| ssc-miR-novel-chr7_34024 | ssc-mir-novel-chr7_34024 | 5p | TGATGCCTGGCATTTTGTGTGT |  |  |  |
| ssc-miR-novel-chr7_34189 | ssc-mir-novel-chr7_34189 | 3p | GTTCAAGTCCAGTTCTG |  |  |  |
| ssc-miR-novel-chr7_34218 | ssc-mir-novel-chr7_34218 | 5p | GTGGTCAAGGCCTGTCGCCGCTGT |  |  |  |
| ssc-miR-novel-chr7_34297 | ssc-mir-novel-chr7_34297 | 3p | TGCATCTGTGGCGTAGGCCAGT | 37 | 105 | 0.408695652 |
| ssc-miR-novel-chr7_34314 | ssc-mir-novel-chr7_34314 | 3p | CCTTTCTGTGTTCGAGGCC |  |  |  |
| ssc-miR-novel-chr7_34318 | ssc-mir-novel-chr7_34318 | 3p | AGGCCCTGGAAAATGACTG |  |  |  |
| ssc-miR-novel-chr7_34332 | ssc-mir-novel-chr7_34332 | 3p | TCTAGCATCGAGCACCCGCCT |  |  |  |
| ssc-miR-novel-chr7_34491 | ssc-mir-novel-chr7_34491 | 5p | TACGTGCTAGTTCTGTACTGGG |  |  |  |
| ssc-miR-novel-chr7_34798 | ssc-mir-novel-chr7_34798 | 3p | AGTCCCATCTGGGTCGCCA | 4 |  | 1.4 |
| ssc-miR-novel-chr7_34809 | ssc-mir-novel-chr7_34809 | 5p | CAGGTGGTGGACTTTCAGC |  |  |  |
| ssc-miR-novel-chr7_34819 | ssc-mir-novel-chr7_34819 | 5p | TCTCTGGGCCTGTGTCT |  |  |  |
| ssc-miR-novel-chr7_34820 | ssc-mir-novel-chr7_34820 | 3p | TCTCTGGGCCTGTGTCT |  |  |  |
| ssc-miR-novel-chr7_34843 | ssc-mir-novel-chr7_34843 | 5p | GCGGGAGCTCTGTCGCT |  |  |  |
| ssc-miR-novel-chr7_34845 | ssc-mir-novel-chr7_34845 | 5p | AAAGGATGGATTGGACAGGCCT |  |  |  |
| ssc-miR-novel-chr7_34976 | ssc-mir-novel-chr7_34976 | 3p | TGAGAACTCTGCTGAAGG |  |  |  |
| ssc-miR-novel-chr7_35006 | ssc-mir-novel-chr7_35006 | 3p | TTCCAGTGGATCTGGGGATGGA |  |  |  |
| ssc-miR-novel-chr7_35053 | ssc-mir-novel-chr7_35053 | 5p | TGCTGTAATTTACTCTCACC |  |  |  |
| ssc-miR-novel-chr7_35084 | ssc-mir-novel-chr7_35084 | 3p | CAAAACAGAGAATCAAGACTGA | 4 |  | 1.4 |
| ssc-miR-novel-chr7_35242 | ssc-mir-novel-chr7_35242 | 3p | ATAGCCTTGTTTGTTTTCTAGG |  |  |  |
| ssc-miR-novel-chr7_35271 | ssc-mir-novel-chr7_35271 | 5p | TGGTGTAGATCTCAGACGCAGC |  | 8 | 0.555555556 |
| ssc-miR-novel-chr7_35273 | ssc-mir-novel-chr7_35273 | 5p | TGATGCCTGGCATTTTGTGTGT |  |  |  |
| ssc-miR-novel-chr7_35375 | ssc-mir-novel-chr7_35375 | 5p | TTGCCCGAGAGCTTGGACCG | 2 |  | 1.2 |
| ssc-miR-novel-chr7_35436 | ssc-mir-novel-chr7_35436 | 3p | GTGCACCTGGGCAAGGAT |  |  |  |
| ssc-miR-novel-chr7_35438 | ssc-mir-novel-chr7_35438 | 3p | GTGCACCTGGGCAAGGAT |  |  |  |
| ssc-miR-novel-chr7_35451 | ssc-mir-novel-chr7_35451 | 5p | TGTGCTGTGCAGTAGGACC | 2 |  | 1.2 |
| ssc-miR-novel-chr7_35509 | ssc-mir-novel-chr7_35509 | 5p | GGGCGGGGGTCCGCCGG |  | 5 | 0.666666667 |
| ssc-miR-novel-chr7_35579 | ssc-mir-novel-chr7_35579 | 5p | GTGCCCAGAGTCGGAAGG |  |  |  |
| ssc-miR-novel-chr8_35622 | ssc-mir-novel-chr8_35622 | 5p | CCGGCCGCGCGCGCGCG |  | 1 | 0.909090909 |
| ssc-miR-novel-chr8_35968 | ssc-mir-novel-chr8_35968 | 5p | TGAGCAAGTAGATTGTATAG |  | 1 | 0.909090909 |
| ssc-miR-novel-chr8_36091 | ssc-mir-novel-chr8_36091 | 3p | TATAAATTGGAATATCTT |  |  |  |
| ssc-miR-novel-chr8_36140 | ssc-mir-novel-chr8_36140 | 5p | GTGTATGTGCTTGGCTG |  |  |  |
| ssc-miR-novel-chr8_36342 | ssc-mir-novel-chr8_36342 | 5p | TGAGCCACAGAAACTCCAGGAC |  |  |  |
| ssc-miR-novel-chr8_36412 | ssc-mir-novel-chr8_36412 | 5p | GTGAGGGGCAGAGAGAGAGA |  |  |  |
| ssc-miR-novel-chr8_36469 | ssc-mir-novel-chr8_36469 | 3p | TGGACTGGATGACAATCTGCAGG |  |  |  |
| ssc-miR-novel-chr8_36485 | ssc-mir-novel-chr8_36485 | 3p | CAGATGGATGTAGATACAGATC |  |  |  |
| ssc-miR-novel-chr8_36503 | ssc-mir-novel-chr8_36503 | 3p | TTCTGGGAACCGGTTTTGCTGCT |  |  |  |
| ssc-miR-novel-chr8_36529 | ssc-mir-novel-chr8_36529 | 3p | CCTCCGGTCCGCCTGCGGTGG |  |  |  |
| ssc-miR-novel-chr8_36585 | ssc-mir-novel-chr8_36585 | 5p | AGATGGATTTTTGGAGATGG | 3 | 19 | 0.448275862 |
| ssc-miR-novel-chr8_36601 | ssc-mir-novel-chr8_36601 | 5p | CAGCAATTTATTTTCCAGTGA |  |  |  |
| ssc-miR-novel-chr8_36618 | ssc-mir-novel-chr8_36618 | 3p | TCTGTGGTTTGTTGAATCTGTCG |  |  |  |
| ssc-miR-novel-chr8_36650 | ssc-mir-novel-chr8_36650 | 3p | CTCAAGTGATGCCTCTGCTT |  |  |  |
| ssc-miR-novel-chr8_36861 | ssc-mir-novel-chr8_36861 | 5p | AACTGGATGTAAGATAGAATCAT |  |  |  |
| ssc-miR-novel-chr8_37121 | ssc-mir-novel-chr8_37121 | 5p | AACTGGATGTAAGATAGAATCAT |  |  |  |
| ssc-miR-novel-chr8_37190 | ssc-mir-novel-chr8_37190 | 3p | TCTGTGAACTAGAAACCTCTGG | 136 | 148 | 0.924050633 |
| ssc-miR-novel-chr8_37196 | ssc-mir-novel-chr8_37196 | 3p | CTCCTGGCTGGCTCGCCA |  | 1 | 0.909090909 |
| ssc-miR-novel-chr8_37351 | ssc-mir-novel-chr8_37351 | 5p | GGGCGGGGGTCCGCCGG |  | 5 | 0.666666667 |
| ssc-miR-novel-chr9_37440 | ssc-mir-novel-chr9_37440 | 5p | CTCCTGGCTGGCTCGCCA |  | 1 | 0.909090909 |
| ssc-miR-novel-chr9_37600 | ssc-mir-novel-chr9_37600 | 5p | AGGAAGCCCTGGAGGGG |  |  |  |
| ssc-miR-novel-chr9_37672 | ssc-mir-novel-chr9_37672 | 5p | TGGCAGTGGAATTAGTGATTGT |  |  |  |
| ssc-miR-novel-chr9_37686 | ssc-mir-novel-chr9_37686 | 5p | ACTGGCCTTGGAGTCAGAAGT |  |  |  |
| ssc-miR-novel-chr9_37717 | ssc-mir-novel-chr9_37717 | 3p | AGGGCCCTGGCAGGGTGGGA |  |  |  |
| ssc-miR-novel-chr9_37731 | ssc-mir-novel-chr9_37731 | 3p | GCAGCAGAGGAAAGGGTTC |  |  |  |
| ssc-miR-novel-chr9_37946 | ssc-mir-novel-chr9_37946 | 5p | CTGAGAGATGGGAGAGTG |  |  |  |
| ssc-miR-novel-chr9_37990 | ssc-mir-novel-chr9_37990 | 5p | TATGGCTTTTCATTCCTATGTGA | 13 | 1752 | 0.013053348 |
| ssc-miR-novel-chr9_38021 | ssc-mir-novel-chr9_38021 | 3p | TCTGGCGGGAAGTTGTGGTC | 7 |  | 1.7 |
| ssc-miR-novel-chr9_38059 | ssc-mir-novel-chr9_38059 | 3p | CGATACAGAGGACTGACTGTAC | 2 |  | 1.2 |
| ssc-miR-novel-chr9_38293 | ssc-mir-novel-chr9_38293 | 3p | CACAGCAAGTGTAGACAGGCA |  |  |  |
| ssc-miR-novel-chr9_38296 | ssc-mir-novel-chr9_38296 | 5p | TAACCAATGTGCAGACTACTGT |  | 2 | 0.833333333 |
| ssc-miR-novel-chr9_38471 | ssc-mir-novel-chr9_38471 | 3p | TCTCCTTGCGAGTCTCTGCTGCC |  |  |  |
| ssc-miR-novel-chr9_38476 | ssc-mir-novel-chr9_38476 | 5p | CAGCCTGGCTTCTCCTGACATGG |  |  |  |
| ssc-miR-novel-chr9_38517 | ssc-mir-novel-chr9_38517 | 3p | CACGCTTGTGTCGTTGGAGTGGC |  |  |  |
| ssc-miR-novel-chr9_38594 | ssc-mir-novel-chr9_38594 | 3p | TCGTGCACAGATGTGGTCTCG |  |  |  |
| ssc-miR-novel-chr9_38736 | ssc-mir-novel-chr9_38736 | 3p | GACTCCAAAGTCTGCCTC |  |  |  |
| ssc-miR-novel-chr9_38827 | ssc-mir-novel-chr9_38827 | 5p | AGGCAGTGTAATTAGCTGATTGT | 4 |  | 1.4 |
| ssc-miR-novel-chr9_38871 | ssc-mir-novel-chr9_38871 | 5p | AGAGACCTGCTGATACAGCTG |  |  |  |
| ssc-miR-novel-chr9_38959 | ssc-mir-novel-chr9_38959 | 5p | TACCTGGTTGATCCTGC | 7 | 40 | 0.34 |
| ssc-miR-novel-chr9_39012 | ssc-mir-novel-chr9_39012 | 3p | CTCCCTGAGCTGTCTCTG |  |  |  |
| ssc-miR-novel-chr9_39041 | ssc-mir-novel-chr9_39041 | 5p | TATGGCTTTTCATTCCTATGTGA | 13 | 1752 | 0.013053348 |
| ssc-miR-novel-chr9_39126 | ssc-mir-novel-chr9_39126 | 3p | TCACTGGAGTTTTGTTTCAACA | 4 | 5 | 0.933333333 |
| ssc-miR-novel-chr9_39325 | ssc-mir-novel-chr9_39325 | 5p | CCATCTGTGGGATTATGACTG | 4 | 14 | 0.583333333 |
| ssc-miR-novel-chr9_39438 | ssc-mir-novel-chr9_39438 | 3p | TTGAAAGGCTATTTCTTGGTC |  |  |  |
| ssc-miR-novel-chr9_39553 | ssc-mir-novel-chr9_39553 | 5p | TCTGTACGTGGGAGGGTGTGT |  |  |  |
| ssc-miR-novel-chr9_39595 | ssc-mir-novel-chr9_39595 | 5p | TCGGCAGCTGCTTGGCGTCCGAGG |  | 8 | 0.555555556 |
| ssc-miR-novel-chrPRV_425 | ssc-mir-novel-chrPRV_425 | 3p | TCTCACCCCTGGGTCCGTCGC | 809 |  | 81.9 |
| ssc-miR-novel-chrPRV_428 | ssc-mir-novel-chrPRV_428 | 5p | CTCATCCCGTCAGACCTGCGCC | 842 |  | 85.2 |
| ssc-miR-novel-chrPRV_434 | ssc-mir-novel-chrPRV_434 | 5p | CGTACCGACCCGCCTACCAGGCA | 37 |  | 4.7 |
| ssc-miR-novel-chrPRV_435 | ssc-mir-novel-chrPRV_435 | 3p | ATGAGTGGATGGATGGAGGCGA | 307 |  | 31.7 |
| ssc-miR-novel-chrPRV_441 | ssc-mir-novel-chrPRV_441 | 5p | ACCCGCGGATGGCGAGGATG | 10 |  | 2 |
| ssc-miR-novel-chrX_39845 | ssc-mir-novel-chrX_39845 | 3p | CGGCGGCGACTCTGGACTC | 307 | 171 | 1.751381215 |
| ssc-miR-novel-chrX_39944 | ssc-mir-novel-chrX_39944 | 3p | CTCCCACGTGCAGGGTTTGCA | 2 | 5 | 0.8 |
| ssc-miR-novel-chrX_39950 | ssc-mir-novel-chrX_39950 | 5p | TAATCCTTGCTACCTGGGTGAGA | 8 | 89 | 0.181818182 |
| ssc-miR-novel-chrX_39952 | ssc-mir-novel-chrX_39952 | 5p | TACCCATTGCATATCGGAGTTG | 370 | 424 | 0.875576037 |
| ssc-miR-novel-chrX_39953 | ssc-mir-novel-chrX_39953 | 3p | ATGCACCTGGGCAAGGATT |  | 6 | 0.625 |
| ssc-miR-novel-chrX_39968 | ssc-mir-novel-chrX_39968 | 3p | CTCCAAGCATCGTGACCCAGGTT | 3 |  | 1.3 |
| ssc-miR-novel-chrX_39978 | ssc-mir-novel-chrX_39978 | 3p | CTCCAAGCATCGTGACCCAGGTT | 3 |  | 1.3 |
| ssc-miR-novel-chrX_40068 | ssc-mir-novel-chrX_40068 | 5p | TGATAATACAACCTGATAAGTGC |  |  |  |
| ssc-miR-novel-chrX_40069 | ssc-mir-novel-chrX_40069 | 5p | TTACAATACAACCTGATAAGTGC |  |  |  |
| ssc-miR-novel-chrX_40077 | ssc-mir-novel-chrX_40077 | 3p | ATTCCTAGAAATTGTTCACAAT |  |  |  |
| ssc-miR-novel-chrX_40247 | ssc-mir-novel-chrX_40247 | 3p | TACCCAGAGCGTGCAGTGTGA |  | 7 | 0.588235294 |
| ssc-miR-novel-chrX_40249 | ssc-mir-novel-chrX_40249 | 5p | AGGTCCTCAATAAGTATTTGTT |  |  |  |
| ssc-miR-novel-chrX_40252 | ssc-mir-novel-chrX_40252 | 5p | TTCATTCGGCTGTCCAGATGTA | 48 | 69 | 0.734177215 |
| ssc-miR-novel-chrX_40254 | ssc-mir-novel-chrX_40254 | 5p | TGAGTACCGCCATGTCTGTTGGGA |  | 2 | 0.833333333 |
| ssc-miR-novel-chrX_40255 | ssc-mir-novel-chrX_40255 | 3p | TTGCATATGTAGGATGTCCCA | 7 | 1 | 1.545454545 |
| ssc-miR-novel-chrX_40262 | ssc-mir-novel-chrX_40262 | 5p | CCACCAGAAAGAGACTGTAGGG |  |  |  |
| ssc-miR-novel-chrX_40264 | ssc-mir-novel-chrX_40264 | 5p | CCACCAGAAAGAGACTGTAGGG |  |  |  |
| ssc-miR-novel-chrX_40266 | ssc-mir-novel-chrX_40266 | 5p | CCACCAGAAAGAGACTGTAGGG |  |  |  |
| ssc-miR-novel-chrX_40268 | ssc-mir-novel-chrX_40268 | 5p | CCACCAGAAAGAGACTGTAGGG |  |  |  |
| ssc-miR-novel-chrX_40461 | ssc-mir-novel-chrX_40461 | 3p | CAGAGGCCAGAGGGCAGGACGCT |  |  |  |
| ssc-miR-novel-chrX_40477 | ssc-mir-novel-chrX_40477 | 3p | CAGCAAGGGTGGTCCAGGGAGT |  |  |  |
| ssc-miR-novel-chrX_40484 | ssc-mir-novel-chrX_40484 | 5p | AAGGGCGGGAGCGGCGGG | 90 | 10 | 5 |
| ssc-miR-novel-chrX_40486 | ssc-mir-novel-chrX_40486 | 5p | TGCACGGAGCAGCAGGGTCTGA |  | 1 | 0.909090909 |
| ssc-miR-novel-chrX_40490 | ssc-mir-novel-chrX_40490 | 5p | GTAGGGCCGGCCCGCCCGGGA |  |  |  |
| ssc-miR-novel-chrX_40522 | ssc-mir-novel-chrX_40522 | 3p | CATGCCTTGAGTGTAGGAC | 31 | 18 | 1.464285714 |
| ssc-miR-novel-chrX_40528 | ssc-mir-novel-chrX_40528 | 3p | AACCAGACTCTGAGAGCAGGACT |  |  |  |
| ssc-miR-novel-chrX_40533 | ssc-mir-novel-chrX_40533 | 5p | GAAAATGGATGGCACTGGAGT | 8 | 20 | 0.6 |
| ssc-miR-novel-chrX_40539 | ssc-mir-novel-chrX_40539 | 5p | TGGTCTAGCGGTTAGGA |  | 11 | 0.476190476 |
| ssc-miR-novel-chrX_40608 | ssc-mir-novel-chrX_40608 | 5p | GGTGGAGGCATGTAGTTCCTAA |  |  |  |
| ssc-miR-novel-chrX_40705 | ssc-mir-novel-chrX_40705 | 5p | AGCTACATTGTCTGCTGGGTTT | 845 | 147 | 5.445859873 |
| ssc-miR-novel-chrX_40783 | ssc-mir-novel-chrX_40783 | 5p | GTATGTGAGCGGGGGGCTGGTGGGA |  | 3 | 0.769230769 |
| ssc-miR-novel-chrX_40912 | ssc-mir-novel-chrX_40912 | 3p | AAAGCTGTGGATTCTGGCAAATGG |  |  |  |
| ssc-miR-novel-chrX_41000 | ssc-mir-novel-chrX_41000 | 5p | CACCTGGGAGGATCGGAG |  |  |  |
| ssc-miR-novel-chrX_41021 | ssc-mir-novel-chrX_41021 | 5p | CCACCAGAAAGAGACTGTAGGG |  |  |  |
| ssc-miR-novel-chrX_41061 | ssc-mir-novel-chrX_41061 | 5p | CCTGGACTTGAAGTCAGAAGGC |  | 7 | 0.588235294 |
| ssc-miR-novel-chrX_41131 | ssc-mir-novel-chrX_41131 | 5p | TGGAAGGTGGATCTGGGC |  |  |  |
| ssc-miR-novel-chrX_41178 | ssc-mir-novel-chrX_41178 | 3p | TGAACGGTGCCTGTGTGGCTAGA |  |  |  |
| ssc-miR-novel-chrX_41179 | ssc-mir-novel-chrX_41179 | 5p | TATCCAGACAGGTGCTGTTCT |  |  |  |
| ssc-miR-novel-chrX_41184 | ssc-mir-novel-chrX_41184 | 3p | TGAACGGCGCCTGTGTGGTTAGA |  | 2 | 0.833333333 |
| ssc-miR-novel-chrX_41186 | ssc-mir-novel-chrX_41186 | 3p | TGAACAGGGCCTTTCTGGGTCGAG |  | 1 | 0.909090909 |
| ssc-miR-novel-chrX_41188 | ssc-mir-novel-chrX_41188 | 3p | CAACAGTCCCTGCCTGGGTAGA |  |  |  |
| ssc-miR-novel-chrX_41190 | ssc-mir-novel-chrX_41190 | 3p | AATGGCGCTTTTTTGTGAAGA | 2 | 431 | 0.027210884 |
| ssc-miR-novel-chrX_41192 | ssc-mir-novel-chrX_41192 | 3p | TGAATGGCGCCTTTCTGAGTAGA |  | 17 | 0.37037037 |
| ssc-miR-novel-chrX_41194 | ssc-mir-novel-chrX_41194 | 3p | TGATTGGCACCTCTTTGAGTGA |  | 30 | 0.25 |
| ssc-miR-novel-chrX_41196 | ssc-mir-novel-chrX_41196 | 3p | ACTGTCACCTTTTTGAGTAGA |  | 9 | 0.526315789 |
| ssc-miR-novel-chrX_41200 | ssc-mir-novel-chrX_41200 | 3p | TGATTGACACCTCTGTTAGTGGA | 2 | 16 | 0.461538462 |
| ssc-miR-novel-chrX_41202 | ssc-mir-novel-chrX_41202 | 3p | TGATTGGCACCTCTTGGAGTGA |  | 29 | 0.256410256 |
| ssc-miR-novel-GL892353-1_41323 | ssc-mir-novel-GL892353-1_41323 | 5p | CGACATGGACACGCGTCATGA |  |  |  |
| ssc-miR-novel-GL892520-2_41438 | ssc-mir-novel-GL892520-2_41438 | 5p | GGTGATGATGACGATGAAGCTGAAA |  | 7 | 0.588235294 |
| ssc-miR-novel-GL892841-1_41670 | ssc-mir-novel-GL892841-1_41670 | 5p | GAGACCATGGAGAGAAAAA |  |  |  |
| ssc-miR-novel-GL892848-2_41682 | ssc-mir-novel-GL892848-2_41682 | 5p | GTGGTCAAGGCCTGTCGCCGCTGT |  |  |  |
| ssc-miR-novel-GL892871-2_41708 | ssc-mir-novel-GL892871-2_41708 | 5p | TTCAAGTAATTCAGGATAGGTT | 3921 | 451 | 8.527114967 |
| ssc-miR-novel-GL892918-2_41750 | ssc-mir-novel-GL892918-2_41750 | 5p | CTAGAGTGGATTCTGCA |  |  |  |
| ssc-miR-novel-GL892946-2_41815 | ssc-mir-novel-GL892946-2_41815 | 3p | TCGGAGCAGCGCGCGAGA | 6 | 4 | 1.142857143 |
| ssc-miR-novel-GL892961-2_41842 | ssc-mir-novel-GL892961-2_41842 | 3p | CAGACCCTGAGCTGCCTCTAGA |  | 2 | 0.833333333 |
| ssc-miR-novel-GL893103-2_41962 | ssc-mir-novel-GL893103-2_41962 | 3p | AGTCCTCTCCTGGGCACCT | 11 |  | 2.1 |
| ssc-miR-novel-GL893138-2_41998 | ssc-mir-novel-GL893138-2_41998 | 5p | GGCTCCCTCCACCCGCC | 3 | 6 | 0.8125 |
| ssc-miR-novel-GL893173-1_42029 | ssc-mir-novel-GL893173-1_42029 | 3p | CCTTCGAGGATGCGGATGTCACC |  |  |  |
| ssc-miR-novel-GL893222-2_42067 | ssc-mir-novel-GL893222-2_42067 | 3p | AGAGGGCTGTGGGAGAGA |  |  |  |
| ssc-miR-novel-GL893230-2_42082 | ssc-mir-novel-GL893230-2_42082 | 3p | CTGTGCTTCTGCTCGGA |  |  |  |
| ssc-miR-novel-GL893233-1_42087 | ssc-mir-novel-GL893233-1_42087 | 5p | ATTGAGAACACTGACATAACA |  |  |  |
| ssc-miR-novel-GL893271-1_42121 | ssc-mir-novel-GL893271-1_42121 | 5p | AGAGGTGCGGCTTTGGCTGGA |  | 1 | 0.909090909 |
| ssc-miR-novel-GL893334-2_42198 | ssc-mir-novel-GL893334-2_42198 | 5p | TCTGGAGGACGCTAGAGCTGGGC |  |  |  |
| ssc-miR-novel-GL893334-2_42199 | ssc-mir-novel-GL893334-2_42199 | 3p | CAGAAGGGGAGTCGGAGCAGA |  |  |  |
| ssc-miR-novel-GL893571-2_42428 | ssc-mir-novel-GL893571-2_42428 | 5p | CACGGGTTCGATCCCTGGTGTGGGC |  |  |  |
| ssc-miR-novel-GL893616-2_42510 | ssc-mir-novel-GL893616-2_42510 | 3p | CTCAGACCTCAGGCTTGGAGCC |  |  |  |
| ssc-miR-novel-GL893653-2_42520 | ssc-mir-novel-GL893653-2_42520 | 5p | TTCAGGGTCCAGGATTGCTATAG |  | 1 | 0.909090909 |
| ssc-miR-novel-GL893741-1_42616 | ssc-mir-novel-GL893741-1_42616 | 5p | GGTGAGCACTCTGGACT |  | 4 | 0.714285714 |
| ssc-miR-novel-GL893763-1_42633 | ssc-mir-novel-GL893763-1_42633 | 3p | TCTTTCCTTGATTAAAACTGG |  |  |  |
| ssc-miR-novel-GL893833-1_42684 | ssc-mir-novel-GL893833-1_42684 | 3p | TTTTTGATGGGAGAAGAGAGA |  |  |  |
| ssc-miR-novel-GL894036-2_42873 | ssc-mir-novel-GL894036-2_42873 | 3p | CAAACCAGCACGTCAAGGGCCC |  |  |  |
| ssc-miR-novel-GL894094-2_42949 | ssc-mir-novel-GL894094-2_42949 | 3p | GCATTGGGGGTTCAGGGG | 14 | 15 | 0.96 |
| ssc-miR-novel-GL894224-1_43070 | ssc-mir-novel-GL894224-1_43070 | 5p | CTTTGGATCTCTGGTGACAGG |  |  |  |
| ssc-miR-novel-GL894231-1_43077 | ssc-mir-novel-GL894231-1_43077 | 5p | TGGTCGACCAGTTGGAAAGTAAT | 3 | 5 | 0.866666667 |
| ssc-miR-novel-GL894231-1_43090 | ssc-mir-novel-GL894231-1_43090 | 3p | CCCAATACACGGTCGATCTCT | 2 | 3 | 0.923076923 |
| ssc-miR-novel-GL894231-1_43091 | ssc-mir-novel-GL894231-1_43091 | 5p | AGAGGCTGGCCGTGATGAATTCG |  | 1 | 0.909090909 |
| ssc-miR-novel-GL894231-1_43093 | ssc-mir-novel-GL894231-1_43093 | 5p | TGTGACTGGTTGACCAGAGGGT |  |  |  |
| ssc-miR-novel-GL894231-1_43098 | ssc-mir-novel-GL894231-1_43098 | 3p | TGTATGTCAACTGATCCACAGT |  | 4 | 0.714285714 |
| ssc-miR-novel-GL894231-1_43100 | ssc-mir-novel-GL894231-1_43100 | 3p | ATAATACATGGTTAACCTCTTT |  |  |  |
| ssc-miR-novel-GL894231-1_43101 | ssc-mir-novel-GL894231-1_43101 | 5p | GGAGAAATTATCCTTGGTGTGTT |  | 1 | 0.909090909 |
| ssc-miR-novel-GL894231-1_43114 | ssc-mir-novel-GL894231-1_43114 | 3p | ATCATAGAGGAAAATCCACA |  |  |  |
| ssc-miR-novel-GL894266-1_43136 | ssc-mir-novel-GL894266-1_43136 | 5p | TTGTTTCTTGCATGTGCTCTGATT |  |  |  |
| ssc-miR-novel-GL894404-1_43244 | ssc-mir-novel-GL894404-1_43244 | 3p | TTGTGCGTTGGCAGGATGGGCCGGA |  |  |  |
| ssc-miR-novel-GL894430-2_43273 | ssc-mir-novel-GL894430-2_43273 | 3p | ATAAAACTTAGTCGCTGCT |  |  |  |
| ssc-miR-novel-GL894430-2_43278 | ssc-mir-novel-GL894430-2_43278 | 3p | ATAAAACTTAGTCGCTGCT |  |  |  |
| ssc-miR-novel-GL894520-2_43333 | ssc-mir-novel-GL894520-2_43333 | 3p | CTCAAGTGATGCCTCTGCTT |  |  |  |
| ssc-miR-novel-GL894542-2_43355 | ssc-mir-novel-GL894542-2_43355 | 5p | TTTGCTCTGCTCCTGCCACAT | 2 |  | 1.2 |
| ssc-miR-novel-GL894570-2_43376 | ssc-mir-novel-GL894570-2_43376 | 3p | TGTTGTCCTCCGAATCTGAAT |  |  |  |
| ssc-miR-novel-GL894677-1_43524 | ssc-mir-novel-GL894677-1_43524 | 3p | TTCACTTTGGATTTTCAGCC |  |  |  |
| ssc-miR-novel-GL894726-2_43559 | ssc-mir-novel-GL894726-2_43559 | 3p | CGGGGCAGCTCAGTACAGGAC |  |  |  |
| ssc-miR-novel-GL894726-2_43560 | ssc-mir-novel-GL894726-2_43560 | 5p | CGGGGCAGCTCAGTACAGGAC |  |  |  |
| ssc-miR-novel-GL894850-1_43647 | ssc-mir-novel-GL894850-1_43647 | 5p | CAGGACTGGTGACTGGGGTG |  |  |  |
| ssc-miR-novel-GL894875-2_43662 | ssc-mir-novel-GL894875-2_43662 | 5p | TGAGGTGGTAGATGGTATAG |  |  |  |
| ssc-miR-novel-GL894932-2_43717 | ssc-mir-novel-GL894932-2_43717 | 5p | TTCCAGGGAAGAAAGGAGGAAC |  |  |  |
| ssc-miR-novel-GL895030-2_43791 | ssc-mir-novel-GL895030-2_43791 | 5p | GCAGAGCAGCGGCAGGAGG |  |  |  |
| ssc-miR-novel-GL895143-1_43893 | ssc-mir-novel-GL895143-1_43893 | 5p | TTCCTGGGTCTCGTGGTCTCAGTCT |  |  |  |
| ssc-miR-novel-GL895351-2_44043 | ssc-mir-novel-GL895351-2_44043 | 5p | GCGCAGCACATCATGGTTTA |  |  |  |
| ssc-miR-novel-GL895485-2_44130 | ssc-mir-novel-GL895485-2_44130 | 3p | AGGCTCTGATTGGATTGGGTG |  |  |  |
| ssc-miR-novel-GL895563-1_44191 | ssc-mir-novel-GL895563-1_44191 | 3p | TAGCCTGACGCTGATGATTGT |  |  |  |
| ssc-miR-novel-GL895621-2_44210 | ssc-mir-novel-GL895621-2_44210 | 5p | TCTTCCATGTGCCACGGGTGTAGCT |  |  |  |
| ssc-miR-novel-GL896133-1_44557 | ssc-mir-novel-GL896133-1_44557 | 5p | TGCTCAGAGGTCGGAGGTGGAG |  | 1 | 0.909090909 |
| ssc-miR-novel-GL896157-1_44574 | ssc-mir-novel-GL896157-1_44574 | 3p | TGGGCTCCAAACCCCTGTCCAGG |  |  |  |
| ssc-miR-novel-GL896208-1_44612 | ssc-mir-novel-GL896208-1_44612 | 3p | TCTGGCCTTGGCCTCAGGCCTGGC |  |  |  |
| ssc-miR-novel-GL896241-2_44636 | ssc-mir-novel-GL896241-2_44636 | 5p | TTCTGGAAGATGTAGTCTGGA |  |  |  |
| ssc-miR-novel-GL896292-1_44714 | ssc-mir-novel-GL896292-1_44714 | 3p | CTTCTCTGTTACTTCCCTCAGG |  |  |  |
| ssc-miR-novel-GL896302-1_44731 | ssc-mir-novel-GL896302-1_44731 | 3p | GCAAAGCACACGGCCTGCAGAGA |  | 22 | 0.3125 |
| ssc-miR-novel-GL896425-1_44856 | ssc-mir-novel-GL896425-1_44856 | 3p | TGGTGCCTGACGTCTTGGCAGT |  |  |  |
| ssc-miR-novel-GL896485-1_44921 | ssc-mir-novel-GL896485-1_44921 | 3p | AGAGGAGCCGCGACAGAGCCGG | 2 | 6 | 0.75 |
| ssc-miR-novel-GL896501-1_44926 | ssc-mir-novel-GL896501-1_44926 | 5p | TGTGGGGCCACGCCCTCAGGCTGT | 2 | 1 | 1.090909091 |
| ssc-miR-novel-JH118484-1_41578 | ssc-mir-novel-JH118484-1_41578 | 5p | CCGGACTCTCTAAATCTCACCT |  |  |  |
| ssc-miR-novel-JH118486-1_41595 | ssc-mir-novel-JH118486-1_41595 | 3p | TTATCCGGGCTTGTGCTGAAGC | 8 | 1 | 1.636363636 |
| ssc-miR-novel-JH118486-1_41599 | ssc-mir-novel-JH118486-1_41599 | 5p | AGCACTTACGGATTCTGGGGG |  | 2 | 0.833333333 |
| ssc-miR-novel-JH118494-1_41628 | ssc-mir-novel-JH118494-1_41628 | 3p | TCTGAGATGTGACCTGGGCAT | 29 | 7 | 2.294117647 |
| ssc-miR-novel-JH118511-1_41721 | ssc-mir-novel-JH118511-1_41721 | 3p | ATTGGGACTGAGACACG |  |  |  |
| ssc-miR-novel-JH118523-1_41818 | ssc-mir-novel-JH118523-1_41818 | 5p | AAGCCAGAGTCAGGGGACACTGT |  |  |  |
| ssc-miR-novel-JH118527-1_41847 | ssc-mir-novel-JH118527-1_41847 | 3p | CTCACGTCCATGGTCAGCACCGTG |  |  |  |
| ssc-miR-novel-JH118570-1_42024 | ssc-mir-novel-JH118570-1_42024 | 3p | CTCTCGGCTGCTGAGGCCT |  |  |  |
| ssc-miR-novel-JH118585-1_42104 | ssc-mir-novel-JH118585-1_42104 | 5p | TCTGGTCCAGACACTGTGGAGC |  |  |  |
| ssc-miR-novel-JH118620-1_42301 | ssc-mir-novel-JH118620-1_42301 | 3p | AATGATGCCCCTTAGAGTTGAGC |  |  |  |
| ssc-miR-novel-JH118636-1_42370 | ssc-mir-novel-JH118636-1_42370 | 3p | AGAGGGAGTAGGTTTCATA |  |  |  |
| ssc-miR-novel-JH118644-1_42413 | ssc-mir-novel-JH118644-1_42413 | 3p | GACTCCAAAGTCTGCCTC |  |  |  |
| ssc-miR-novel-JH118647-1_42435 | ssc-mir-novel-JH118647-1_42435 | 3p | TGTGTGGCTAAGTGGTAGATTT |  | 2 | 0.833333333 |
| ssc-miR-novel-JH118654-1_42487 | ssc-mir-novel-JH118654-1_42487 | 5p | TGGCAGTGTATTGTTAGCTGGT | 40 |  | 5 |
| ssc-miR-novel-JH118655-1_42490 | ssc-mir-novel-JH118655-1_42490 | 5p | AACACATGTGTGCTGAGTGGAACT |  | 1 | 0.909090909 |
| ssc-miR-novel-JH118656-1_42504 | ssc-mir-novel-JH118656-1_42504 | 5p | TGGCTCATTTCAGCAGGAAG |  | 6 | 0.625 |
| ssc-miR-novel-JH118676-1_42617 | ssc-mir-novel-JH118676-1_42617 | 3p | ACTGTGTGTGAGGAAGTC |  |  |  |
| ssc-miR-novel-JH118774-1_43297 | ssc-mir-novel-JH118774-1_43297 | 3p | TAACCCGACATTCAAGGCCTGT |  |  |  |
| ssc-miR-novel-JH118788-1_43363 | ssc-mir-novel-JH118788-1_43363 | 5p | AAAGGATGGATTGGACAGGCCT |  |  |  |
| ssc-miR-novel-JH118806-1_43515 | ssc-mir-novel-JH118806-1_43515 | 3p | ATTCATATGCAGCTGTAGGAA |  |  |  |
| ssc-miR-novel-JH118806-1_43518 | ssc-mir-novel-JH118806-1_43518 | 3p | ATTCATATGCAGCTGTAGGAA |  |  |  |
| ssc-miR-novel-JH118928-1_44242 | ssc-mir-novel-JH118928-1_44242 | 3p | TCATTCTCCTTCTTTGACCAGA |  |  |  |
| ssc-miR-novel-JH118939-1_44269 | ssc-mir-novel-JH118939-1_44269 | 5p | GACTTAATGGCTGGCTGGGAGG |  | 2 | 0.833333333 |
| ssc-miR-novel-JH118943-1_44283 | ssc-mir-novel-JH118943-1_44283 | 3p | TGAATTACTGTGGATTCTTGG |  |  |  |
| ssc-miR-novel-JH118951-1_44333 | ssc-mir-novel-JH118951-1_44333 | 3p | TCAAGGTCCGCTGTGAACACGG |  |  |  |
| ssc-miR-novel-JH118990-1_44625 | ssc-mir-novel-JH118990-1_44625 | 3p | CACGCTTGTGTCGTTGGAGTGGC |  |  |  |
| ssc-miR-novel-JH118993-1_44650 | ssc-mir-novel-JH118993-1_44650 | 3p | TTGGACTGGAGGTGAGGC |  |  |  |
| ssc-miR-novel-JH118993-1_44655 | ssc-mir-novel-JH118993-1_44655 | 5p | AGCTTATCAGACTGGTGTAG |  |  |  |

| **Mature miRNA-ID** | **Pre-miRNA_ID** | **Pre-miRNA arm (5p or 3p)** | **Mature miRNA -SEQ** | **TagCount (PRV FaΔgE/gI strain infected PK-15 cells)** | **TagCount (Non-infected PK-15 cells)** | **Fold Change (PRV FaΔgE/gI strain infected vs Non-infected)** |
| --- | --- | --- | --- | --- | --- | --- |
| ssc-let-7a | ssc-let-7a-1 | 5p | TGAGGTAGTAGGTTGTATAGTT | 9095 | 9120 | 0.997261774 |
| ssc-let-7a | ssc-let-7a-2 | 5p | TGAGGTAGTAGGTTGTATAGTT | 9095 | 9120 | 0.997261774 |
| ssc-let-7c | ssc-let-7c | 5p | TGAGGTAGTAGGTTGTATGGTT | 995 | 1637 | 0.610200364 |
| ssc-let-7d-5p | ssc-let-7d | 5p | AGAGGTAGTAGGTTGCATAGTT | 372 | 1773 | 0.214245653 |
| ssc-let-7d-3p | ssc-let-7d | 3p | CTATACGACCTGCTGCCTTTCT | 3 | 8 | 0.722222222 |
| ssc-let-7e | ssc-let-7e | 5p | TGAGGTAGGAGGTTGTATAGTT | 595 | 522 | 1.137218045 |
| ssc-let-7f | ssc-let-7f-1 | 5p | TGAGGTAGTAGATTGTATAGTT | 16471 | 66172 | 0.249025415 |
| ssc-let-7f | ssc-let-7f-2 | 5p | TGAGGTAGTAGATTGTATAGTT | 16471 | 66172 | 0.249025415 |
| ssc-let-7g | ssc-let-7g | 5p | TGAGGTAGTAGTTTGTACAGTT | 2526 | 3374 | 0.749408983 |
| ssc-let-7i | ssc-let-7i | 5p | TGAGGTAGTAGTTTGTGCT | 8500 | 3203 | 2.648615002 |
| ssc-miR-1 | ssc-mir-1 | 3p | TGGAATGTAAAGAAGTATGTA | 56 | 61 | 0.929577465 |
| ssc-miR-100 | ssc-mir-100 | 5p | AACCCGTAGATCCGAACTTGTG | 170 | 7099 | 0.025320017 |
| ssc-miR-101 | ssc-mir-101-1 | 3p | TACAGTACTGTGATAACTGAA | 324 | 484 | 0.67611336 |
| ssc-miR-101 | ssc-mir-101-2 | 3p | TACAGTACTGTGATAACTGAA | 324 | 484 | 0.67611336 |
| ssc-miR-103 | ssc-mir-103-1 | 3p | AGCAGCATTGTACAGGGCTATGA | 1338 | 697 | 1.906647808 |
| ssc-miR-103 | ssc-mir-103-2 | 3p | AGCAGCATTGTACAGGGCTATGA | 1338 | 697 | 1.906647808 |
| ssc-miR-105-2 | ssc-mir-105-2 | 5p | TCAAATGCTCAGACTCCTTG |  |  |  |
| ssc-miR-106a | ssc-mir-106a | 5p | AAAAGTGCTTACAGTGCAGGTAGC |  |  |  |
| ssc-miR-107 | ssc-mir-107 | 3p | AGCAGCATTGTACAGGGCTATCA | 235 | 31 | 5.975609756 |
| ssc-miR-10a-5p | ssc-mir-10a | 5p | TACCCTGTAGATCCGAATTTGT | 15805 | 3471 | 4.543234703 |
| ssc-miR-10a-3p | ssc-mir-10a | 3p | CAAATTCGTATCTAGGGGAAT | 72 | 63 | 1.123287671 |
| ssc-miR-10b | ssc-mir-10b | 5p | TACCCTGTAGAACCGAATTTGT | 139310 | 1162 | 118.8737201 |
| ssc-miR-122 | ssc-mir-122 | 5p | TGGAGTGTGACAATGGTGTTTGT |  | 10 | 0.5 |
| ssc-miR-1224 | ssc-mir-1224 | 3p | CACCTCCTCTCTCCTCAGGT |  |  |  |
| ssc-miR-1249 | ssc-mir-1249-1 | 3p | ACGCCCTTCCCCCCCTTCTTCA |  | 1 | 0.909090909 |
| ssc-miR-1249 | ssc-mir-1249-2 | 3p | ACGCCCTTCCCCCCCTTCTTCA |  | 1 | 0.909090909 |
| ssc-miR-124a | ssc-mir-124a-1 | 3p | TAAGGCACGCGGTGAATGCCA |  | 5 | 0.666666667 |
| ssc-miR-124a | ssc-mir-124a-2 | 3p | TAAGGCACGCGGTGAATGCCA |  | 5 | 0.666666667 |
| ssc-miR-125a | ssc-mir-125a | 5p | TCCCTGAGACCCTTTAACCTGTG | 38 | 5 | 3.2 |
| ssc-miR-125b | ssc-mir-125b-1 | 5p | TCCCTGAGACCCTAACTTGTGA | 56 | 582 | 0.111486486 |
| ssc-miR-125b | ssc-mir-125b-2 | 5p | TCCCTGAGACCCTAACTTGTGA | 56 | 582 | 0.111486486 |
| ssc-miR-126-5p | ssc-mir-126 | 5p | CATTATTACTTTTGGTACGCG | 36 | 2 | 3.833333333 |
| ssc-miR-126-3p | ssc-mir-126 | 3p | TCGTACCGTGAGTAATAATGCG | 17 | 9 | 1.421052632 |
| ssc-miR-127 | ssc-mir-127 | 3p | TCGGATCCGTCTGAGCTTGGCT | 2 | 2 | 1 |
| ssc-miR-1271 | ssc-mir-1271 | 3p | TGCCTGCTATGTGCCAGGCA |  | 5 | 0.666666667 |
| ssc-miR-1277 | ssc-mir-1277 | 3p | TACGTAGATATATATGTATTTT |  |  |  |
| ssc-miR-128 | ssc-mir-128-1 | 3p | TCACAGTGAACCGGTCTCTTT | 147 | 248 | 0.608527132 |
| ssc-miR-128 | ssc-mir-128-2 | 3p | TCACAGTGAACCGGTCTCTTT | 147 | 248 | 0.608527132 |
| ssc-miR-1285 | ssc-mir-1285 | 3p | CTGGGCAACATAGCGAGACCCCGT |  | 27 | 0.27027027 |
| ssc-miR-1296-5p | ssc-mir-1296 | 5p | TTAGGGCCCTGGCTCCATCTCC | 2 |  | 1.2 |
| ssc-miR-1296-3p | ssc-mir-1296 | 3p | GAGTGGGGTTTTGACCCTAACC |  |  |  |
| ssc-miR-129a | ssc-mir-129a | 3p | AAGCCCTTACCCCAAAAAGCAT | 3 | 6 | 0.8125 |
| ssc-miR-129b | ssc-mir-129b | 5p | CTTTTTGCGGTCTGGGCTTGC | 137 | 72 | 1.792682927 |
| ssc-miR-1306-5p | ssc-mir-1306 | 5p | CCACCTCCCCTGCAAACGTCCA | 5 | 7 | 0.882352941 |
| ssc-miR-1306-3p | ssc-mir-1306 | 3p | ACGTTGGCTCTGGTGGTGATG |  | 69 | 0.126582278 |
| ssc-miR-1307 | ssc-mir-1307 | 3p | ACTCGGCGTGGCGTCGGTCGTG | 9 | 107 | 0.162393162 |
| ssc-miR-130a | ssc-mir-130a | 3p | CAGTGCAATGTTAAAAGGGCAT | 33 | 3 | 3.307692308 |
| ssc-miR-130b | ssc-mir-130b | 3p | CAGTGCAATGATGAAAGGGCAT | 32 | 27 | 1.135135135 |
| ssc-miR-132 | ssc-mir-132 | 3p | TAACAGTCTACAGCCATGGTCG | 20 | 6 | 1.875 |
| ssc-miR-133a-5p | ssc-mir-133a-1 | 5p | AGCTGGTAAAATGGAACCAAAT |  |  |  |
| ssc-miR-133a-3p | ssc-mir-133a-1 | 3p | TTGGTCCCCTTCAACCAGCTG |  | 2 | 0.833333333 |
| ssc-miR-133a-5p | ssc-mir-133a-2 | 5p | AGCTGGTAAAATGGAACCAAAT |  |  |  |
| ssc-miR-133a-3p | ssc-mir-133a-2 | 3p | TTGGTCCCCTTCAACCAGCTG |  | 2 | 0.833333333 |
| ssc-miR-133b | ssc-mir-133b | 3p | TTTGGTCCCCTTCAACCAGCTAT |  |  |  |
| ssc-miR-1343 | ssc-mir-1343 | 3p | CTCCTGGGGCCCGCACTCTCGC | 13 | 4 | 1.642857143 |
| ssc-miR-135 | ssc-mir-135-1 | 5p | TATGGCTTTTTATTCCTATGTGA |  | 235 | 0.040816327 |
| ssc-miR-135 | ssc-mir-135-2 | 5p | TATGGCTTTTTATTCCTATGTGA |  | 235 | 0.040816327 |
| ssc-miR-136 | ssc-mir-136 | 5p | ACTCCATTTGTTTTGATGATGGA |  |  |  |
| ssc-miR-137 | ssc-mir-137 | 3p | TTATTGCTTAAGAATACGCGTAG |  |  |  |
| ssc-miR-138 | ssc-mir-138 | 5p | AGCTGGTGTTGTGAATCAGGC |  |  |  |
| ssc-miR-139-5p | ssc-mir-139 | 5p | TCTACAGTGCACGTGTCTCCAG | 7 | 44 | 0.314814815 |
| ssc-miR-139-3p | ssc-mir-139 | 3p | TGGAGACGCGGCCCTGTTGGAGT | 3 | 1 | 1.181818182 |
| ssc-miR-140-5p | ssc-mir-140 | 5p | AGTGGTTTTACCCTATGGTAG | 47 |  | 5.7 |
| ssc-miR-140-3p | ssc-mir-140 | 3p | TACCACAGGGTAGAACCACGGAC | 591 | 1026 | 0.58011583 |
| ssc-miR-142-5p | ssc-mir-142 | 5p | CATAAAGTAGAAAGCACTACT | 18 | 17 | 1.037037037 |
| ssc-miR-142-3p | ssc-mir-142 | 3p | TGTAGTGTTTCCTACTTTATGG | 22 | 238 | 0.129032258 |
| ssc-miR-143-5p | ssc-mir-143 | 5p | GGTGCAGTGCTGCATCTCTGG |  |  |  |
| ssc-miR-143-3p | ssc-mir-143 | 3p | TGAGATGAAGCACTGTAGCTC | 63 | 92 | 0.715686275 |
| ssc-miR-144 | ssc-mir-144 | 3p | TACAGTATAGATGATGTAC | 7 |  | 1.7 |
| ssc-miR-145-5p | ssc-mir-145 | 5p | GTCCAGTTTTCCCAGGAATCCCTT |  | 87 | 0.103092784 |
| ssc-miR-145-3p | ssc-mir-145 | 3p | GGATTCCTGGAAATACTGTTCT |  |  |  |
| ssc-miR-1468 | ssc-mir-1468 | 5p | CTCCGTTTGCCTGTTTTGCTGA |  |  |  |
| ssc-miR-146a-5p | ssc-mir-146a | 5p | TGAGAACTGAATTCCATGGGTT | 429 | 1767 | 0.247045582 |
| ssc-miR-146a-3p | ssc-mir-146a | 3p | CCTGTGAAGTTTAGTTCTTCAG |  |  |  |
| ssc-miR-146b | ssc-mir-146b | 5p | TGAGAACTGAATTCCATAGGC | 868 | 4 | 62.71428571 |
| ssc-miR-148a-5p | ssc-mir-148a | 5p | AAAGTTCTGAGACACTCCGACT | 2 |  | 1.2 |
| ssc-miR-148a-3p | ssc-mir-148a | 3p | TCAGTGCACTACAGAACTTTGT | 31 | 182 | 0.213541667 |
| ssc-miR-148b-5p | ssc-mir-148b | 5p | GAAGTTCTGTTATACACTCAGGC | 11 | 5 | 1.4 |
| ssc-miR-148b-3p | ssc-mir-148b | 3p | TCAGTGCATCACAGAACTTTGT | 768 | 1616 | 0.478474785 |
| ssc-miR-149 | ssc-mir-149 | 5p | TCTGGCTCCGTGTCTTCACTCCC |  | 3 | 0.769230769 |
| ssc-miR-150 | ssc-mir-150-1 | 5p | TCTCCCAACCCTTGTACCAGTG |  |  |  |
| ssc-miR-150 | ssc-mir-150-2 | 5p | TCTCCCAACCCTTGTACCAGTG |  |  |  |
| ssc-miR-151-5p | ssc-mir-151 | 5p | TCGAGGAGCTCACAGTCTAGT | 524 | 149 | 3.358490566 |
| ssc-miR-151-3p | ssc-mir-151 | 3p | CTAGACTGAAGCTCCTTGAGGA | 1262 | 3522 | 0.3601359 |
| ssc-miR-152 | ssc-mir-152 | 3p | TCAGTGCATGACAGAACTTGG | 345 | 9880 | 0.035894843 |
| ssc-miR-153 | ssc-mir-153 | 3p | TTGCATAGTCACAAAAGTGA |  |  |  |
| ssc-miR-155-5p | ssc-mir-155 | 5p | TTAATGCTAATTGTGATAGGGG | 753 | 62 | 10.59722222 |
| ssc-miR-155-3p | ssc-mir-155 | 3p | TCCTACATGTTAGCATTAACA |  | 3 | 0.769230769 |
| ssc-miR-15a | ssc-mir-15a | 5p | TAGCAGCACATAATGGTTTGT | 2455 | 156 | 14.84939759 |
| ssc-miR-15b | ssc-mir-15b | 5p | TAGCAGCACATCATGGTTTACA | 432 | 812 | 0.537712895 |
| ssc-miR-16 | ssc-mir-16-1 | 5p | TAGCAGCACGTAAATATTGGCG | 6340 | 1891 | 3.340347186 |
| ssc-miR-16 | ssc-mir-16-2 | 5p | TAGCAGCACGTAAATATTGGCG | 6340 | 1891 | 3.340347186 |
| ssc-miR-17-5p | ssc-mir-17 | 5p | CAAAGTGCTTACAGTGCAGGTAG | 932 | 438 | 2.102678571 |
| ssc-miR-17-3p | ssc-mir-17 | 3p | ACTGCAGTGAAGGCACTTGTAG |  | 15 | 0.4 |
| ssc-miR-181a | ssc-mir-181a-1 | 5p | AACATTCAACGCTGTCGGTGAGTT | 917 | 147 | 5.904458599 |
| ssc-miR-181a | ssc-mir-181a-2 | 5p | AACATTCAACGCTGTCGGTGAGTT | 917 | 147 | 5.904458599 |
| ssc-miR-181b | ssc-mir-181b-1 | 5p | AACATTCATTGCTGTCGGTGGGTT | 254 | 125 | 1.955555556 |
| ssc-miR-181b | ssc-mir-181b-2 | 5p | AACATTCATTGCTGTCGGTGGGTT | 254 | 125 | 1.955555556 |
| ssc-miR-181c | ssc-mir-181c | 5p | AACATTCAACCTGTCGGTGAGT | 2 |  | 1.2 |
| ssc-miR-181d-5p | ssc-mir-181d | 5p | AACATTCATTGTTGTCGGTGGGTT | 5 | 3 | 1.153846154 |
| ssc-miR-181d-3p | ssc-mir-181d | 3p | CCCACCGAGGGATGAATGTCAC |  |  |  |
| ssc-miR-182 | ssc-mir-182 | 5p | TTTGGCAATGGTAGAACTCACACT | 23942 | 4616 | 5.17769131 |
| ssc-miR-183 | ssc-mir-183 | 5p | TATGGCACTGGTAGAATTCACTG | 249 | 3132 | 0.082431572 |
| ssc-miR-1839-5p | ssc-mir-1839 | 5p | AAGGTAGATAGAACAGGTCTTG | 6 | 3 | 1.230769231 |
| ssc-miR-1839-3p | ssc-mir-1839 | 3p | AGACCTACTTTTCTACCAACA |  |  |  |
| ssc-miR-184 | ssc-mir-184 | 3p | TGGACGGAGAACTGATAAGGGT | 88 | 359 | 0.265582656 |
| ssc-miR-185 | ssc-mir-185 | 5p | TGGAGAGAAAGGCAGTTCCTGA | 21 | 1621 | 0.019006744 |
| ssc-miR-186 | ssc-mir-186 | 5p | CAAAGAATTCTCCTTTTGGGCTT | 711 | 113 | 5.861788618 |
| ssc-miR-187 | ssc-mir-187 | 3p | TCGTGTCTTGTGTTGCAGCCGG |  | 3 | 0.769230769 |
| ssc-miR-18a | ssc-mir-18a | 5p | TAAGGTGCATCTAGTGCAGATA | 18 | 9541 | 0.00293163 |
| ssc-miR-18b | ssc-mir-18b | 5p | TAAGGTGCATCTAGTGCAGTTAG |  |  |  |
| ssc-miR-190a | ssc-mir-190a | 5p | TGATATGTTTGATATATTAGG | 40 | 1 | 4.545454545 |
| ssc-miR-190b | ssc-mir-190b | 5p | TGATATGTTTGATATTGGGTTG | 19 | 1 | 2.636363636 |
| ssc-miR-191 | ssc-mir-191 | 5p | CAACGGAATCCCAAAAGCAGCTG | 3403 | 156 | 20.56024096 |
| ssc-miR-192 | ssc-mir-192 | 5p | CTGACCTATGAATTGACAGCC | 14048 | 102 | 125.5178571 |
| ssc-miR-193a-5p | ssc-mir-193a | 5p | TGGGTCTTTGCGGGCGAGATGA | 4 | 201 | 0.066350711 |
| ssc-miR-193a-3p | ssc-mir-193a | 3p | AACTGGCCTACAAAGTCCCAGT | 21 | 45 | 0.563636364 |
| ssc-miR-194a | ssc-mir-194a | 5p | TGTAACAGCAACTCCATGTGG | 27 | 541 | 0.067150635 |
| ssc-miR-194b-5p | ssc-mir-194b | 5p | TGTAACAGCGACTCCATGTGGA |  | 27 | 0.27027027 |
| ssc-miR-194b-3p | ssc-mir-194b | 3p | CCAGTGGAGATGCTGTTACCTT |  |  |  |
| ssc-miR-195 | ssc-mir-195 | 5p | TAGCAGCACAGAAATATTGGC | 7 | 192 | 0.084158416 |
| ssc-miR-196a | ssc-mir-196a-1 | 5p | TAGGTAGTTTCATGTTGTTGGG | 5 |  | 1.5 |
| ssc-miR-196a | ssc-mir-196a-2 | 5p | TAGGTAGTTTCATGTTGTTGGG | 5 |  | 1.5 |
| ssc-miR-196b-5p | ssc-mir-196b-1 | 5p | TAGGTAGTTTCCTGTTGTTGGG | 577 | 984 | 0.59054326 |
| ssc-miR-196b-3p | ssc-mir-196b-1 | 3p | CGACAGCACGACACTGCCTTCA |  |  |  |
| ssc-miR-196b | ssc-mir-196b-2 | 5p | TAGGTAGTTTCCTGTTGTTGGG | 577 | 984 | 0.59054326 |
| ssc-miR-199a-5p | ssc-mir-199a-1 | 5p | CCCAGTGTTCAGACTACCTGTTC |  |  |  |
| ssc-miR-199a-3p | ssc-mir-199a-1 | 3p | ACAGTAGTCTGCACATTGGTTA | 2 | 4 | 0.857142857 |
| ssc-miR-199a-5p | ssc-mir-199a-2 | 5p | CCCAGTGTTCAGACTACCTGTTC |  |  |  |
| ssc-miR-199a-3p | ssc-mir-199a-2 | 3p | ACAGTAGTCTGCACATTGGTTA | 2 | 4 | 0.857142857 |
| ssc-miR-199b-5p | ssc-mir-199b | 5p | CCCAGTGTTTAGACTATCTGTT |  |  |  |
| ssc-miR-199b-3p | ssc-mir-199b | 3p | TACAGTAGTCTGCACATTGGTT | 2 | 4 | 0.857142857 |
| ssc-miR-19a | ssc-mir-19a | 3p | TGTGCAAATCTATGCAAAACTGA | 142 | 6339 | 0.023940778 |
| ssc-miR-19b | ssc-mir-19b-1 | 3p | TGTGCAAATCCATGCAAAACTGA | 303 | 14587 | 0.021442762 |
| ssc-miR-19b | ssc-mir-19b-2 | 3p | TGTGCAAATCCATGCAAAACTGA | 303 | 14587 | 0.021442762 |
| ssc-miR-202-5p | ssc-mir-202 | 5p | TTCCTATGCATATACTTCTTT |  |  |  |
| ssc-miR-202-3p | ssc-mir-202 | 3p | AGAGGTGTAGGCATGGGAA |  |  |  |
| ssc-miR-204 | ssc-mir-204 | 5p | TTCCCTTTGTCATCCTATGCCT | 2 |  | 1.2 |
| ssc-miR-205 | ssc-mir-205 | 5p | TCCTTCATTCCACCGGAGTCTG |  |  |  |
| ssc-miR-206 | ssc-mir-206 | 3p | TGGAATGTAAGGAAGTGTGTGA |  |  |  |
| ssc-miR-208b | ssc-mir-208b | 3p | ATAAGACGAACAAAAGGTTTGT |  |  |  |
| ssc-miR-20a | ssc-mir-20a | 5p | TAAAGTGCTTATAGTGCAGGTA | 748 | 116 | 6.015873016 |
| ssc-miR-20b | ssc-mir-20b-1 | 5p | CAAAGTGCTCACAGTGCAGGTAG |  |  |  |
| ssc-miR-20b | ssc-mir-20b-2 | 5p | CAAAGTGCTCACAGTGCAGGTAG |  |  |  |
| ssc-miR-21 | ssc-mir-21 | 5p | TAGCTTATCAGACTGATGTTGA | 60378 | 382830 | 0.157736914 |
| ssc-miR-210 | ssc-mir-210 | 3p | CTGTGCGTGTGACAGCGGCTGA | 107 | 123 | 0.879699248 |
| ssc-miR-212 | ssc-mir-212 | 5p | ACCTTGGCTCTAGACTGCTTACT | 5 |  | 1.5 |
| ssc-miR-214 | ssc-mir-214 | 3p | ACAGCAGGCACAGACAGGCAG |  |  |  |
| ssc-miR-215 | ssc-mir-215 | 5p | ATGACCTATGAATTGACAGAC |  | 31 | 0.243902439 |
| ssc-miR-216 | ssc-mir-216-1 | 5p | TAATCTCAGCTGGCAACTGTGAG |  |  |  |
| ssc-miR-216 | ssc-mir-216-2 | 5p | TAATCTCAGCTGGCAACTGTGAG |  |  |  |
| ssc-miR-217 | ssc-mir-217-1 | 5p | TACTGCATCAGGAACTGATTGGAT |  |  |  |
| ssc-miR-217 | ssc-mir-217-2 | 5p | TACTGCATCAGGAACTGATTGGAT |  |  |  |
| ssc-miR-218-5p | ssc-mir-218-1 | 5p | TTGTGCTTGATCTAACCATGT |  | 7 | 0.588235294 |
| ssc-miR-218-3p | ssc-mir-218-1 | 3p | ATGGTTCTGTCAAGCACCATG |  |  |  |
| ssc-miR-218 | ssc-mir-218-2 | 5p | TTGTGCTTGATCTAACCATGT |  | 7 | 0.588235294 |
| ssc-miR-218b | ssc-mir-218b | 5p | TTGTGCTTGATCTAACCATGTG |  | 7 | 0.588235294 |
| ssc-miR-219 | ssc-mir-219 | 3p | AGAGTTGAGTCTGGACGTCCCG | 8 | 2 | 1.5 |
| ssc-miR-22-5p | ssc-mir-22 | 5p | AGTTCTTCAGTGGCAAGCTTTA | 8 | 1 | 1.636363636 |
| ssc-miR-22-3p | ssc-mir-22 | 3p | AAGCTGCCAGTTGAAGAACTGT | 964 | 365 | 2.597333333 |
| ssc-miR-221-5p | ssc-mir-221 | 5p | ACCTGGCATACAATGTAGATTTCTGT |  | 39 | 0.204081633 |
| ssc-miR-221-3p | ssc-mir-221 | 3p | AGCTACATTGTCTGCTGGGTTT | 2545 | 147 | 16.27388535 |
| ssc-miR-222 | ssc-mir-222 | 3p | AGCTACATCTGGCTACTGGGTCTC | 283 | 284 | 0.996598639 |
| ssc-miR-224 | ssc-mir-224 | 5p | CAAGTCACTAGTGGTTCCGTTTA | 4 |  | 1.4 |
| ssc-miR-2320-5p | ssc-mir-2320 | 5p | TGGCACAGGGTCCAGCTGTCGG |  | 114 | 0.080645161 |
| ssc-miR-2320-3p | ssc-mir-2320 | 3p | CGATGATGGTCCCTGTGTTTG | 33 | 51 | 0.704918033 |
| ssc-miR-2366 | ssc-mir-2366-1 | 3p | TGGGTCACAGAAGAGGGTCTGG |  |  |  |
| ssc-miR-2366 | ssc-mir-2366-2 | 3p | TGGGTCACAGAAGAGGGTCTGG |  |  |  |
| ssc-miR-23a | ssc-mir-23a | 3p | ATCACATTGCCAGGGATTTCC | 222 | 635 | 0.359689922 |
| ssc-miR-23b | ssc-mir-23b | 3p | ATCACATTGCCAGGGATTACCA | 74 | 143 | 0.549019608 |
| ssc-miR-24-1-5p | ssc-mir-24-1 | 5p | GTGCCTACTGAGCTGAAACACAGT | 21 |  | 3.1 |
| ssc-miR-24-3p | ssc-mir-24-1 | 3p | TGGCTCAGTTCAGCAGGAACAG | 212 | 10251 | 0.021635318 |
| ssc-miR-24-2-5p | ssc-mir-24-2 | 5p | GTGCCTACTGAGCTGATATCAGT |  |  |  |
| ssc-miR-24-3p | ssc-mir-24-2 | 3p | TGGCTCAGTTCAGCAGGAACAG | 212 | 10251 | 0.021635318 |
| ssc-miR-2411 | ssc-mir-2411 | 5p | TGGAGTGACTGTCAGATGCAGC | 48 | 12 | 2.636363636 |
| ssc-miR-2483 | ssc-mir-2483 | 3p | AAACATCTGGTTGGTTGAGAGA | 3 | 13 | 0.565217391 |
| ssc-miR-26a | ssc-mir-26a | 5p | TTCAAGTAATCCAGGATAGGCT | 5371 | 3198 | 1.677369077 |
| ssc-miR-27a | ssc-mir-27a | 3p | TTCACAGTGGCTAAGTTCCGC | 5054 | 549 | 9.059033989 |
| ssc-miR-27b-5p | ssc-mir-27b | 5p | AGAGCTTAGCTGATTGGTGAACA | 3 | 63 | 0.178082192 |
| ssc-miR-27b-3p | ssc-mir-27b | 3p | TTCACAGTGGCTAAGTTCTGC | 6994 | 172 | 38.48351648 |
| ssc-miR-28-5p | ssc-mir-28 | 5p | AAGGAGCTCACAGTCTATTGAG | 13 | 232 | 0.095041322 |
| ssc-miR-28-3p | ssc-mir-28 | 3p | CACTAGATTGTGAGCTCCTGGA | 752 | 617 | 1.215311005 |
| ssc-miR-296-5p | ssc-mir-296 | 5p | GAGGGCCCCCCCCAATCCTGT | 7 | 1 | 1.545454545 |
| ssc-miR-296-3p | ssc-mir-296 | 3p | AGGGTTGGGCGGAGGCTTTCC | 126 | 3 | 10.46153846 |
| ssc-miR-299 | ssc-mir-299 | 5p | ATGGTTTACCGTCCCACATAC |  |  |  |
| ssc-miR-29a | ssc-mir-29a | 3p | CTAGCACCATCTGAAATCGGTTA | 81 | 3939 | 0.023043809 |
| ssc-miR-29b | ssc-mir-29b-1 | 3p | TAGCACCATTTGAAATCAGTGTT | 45 | 209 | 0.251141553 |
| ssc-miR-29b | ssc-mir-29b-2 | 3p | TAGCACCATTTGAAATCAGTGTT | 45 | 209 | 0.251141553 |
| ssc-miR-29c | ssc-mir-29c | 3p | TAGCACCATTTGAAATCGGTTA | 4 | 366 | 0.037234043 |
| ssc-miR-301 | ssc-mir-301 | 3p | CAGTCCAATAGTATTGTCAAAGC |  | 17 | 0.37037037 |
| ssc-miR-30a-5p | ssc-mir-30a | 5p | TGTAAACATCCTCGACTGGAAG | 172369 | 6320 | 27.23206951 |
| ssc-miR-30a-3p | ssc-mir-30a | 3p | CTTTCAGTCGGATGTTTGCAGC | 620 | 1134 | 0.550699301 |
| ssc-miR-30b-5p | ssc-mir-30b | 5p | TGTAAACATCCTACACTCAGCT | 1405 | 191 | 7.039800995 |
| ssc-miR-30b-3p | ssc-mir-30b | 3p | CTGGGAGGTGGATGTTTACTT | 9 | 1 | 1.727272727 |
| ssc-miR-30c-5p | ssc-mir-30c-1 | 5p | TGTAAACATCCTACACTCTCAGC | 3158 | 209 | 14.46575342 |
| ssc-miR-30c-1-3p | ssc-mir-30c-1 | 3p | CTGGGAGAGGGTTGTTTACT |  |  |  |
| ssc-miR-30c-5p | ssc-mir-30c-2 | 5p | TGTAAACATCCTACACTCTCAGC | 3158 | 209 | 14.46575342 |
| ssc-miR-30c-3p | ssc-mir-30c-2 | 3p | CTGGGAGAAGGCTGTTTACTCT | 15 | 36 | 0.543478261 |
| ssc-miR-30d | ssc-mir-30d | 5p | TGTAAACATCCCCGACTGGAAGCT | 11943 | 1786 | 6.655345212 |
| ssc-miR-30e-5p | ssc-mir-30e | 5p | TGTAAACATCCTTGACTGGAAGCT | 2510 | 812 | 3.065693431 |
| ssc-miR-30e-3p | ssc-mir-30e | 3p | CTTTCAGTCGGATGTTTACAGC | 152 | 347 | 0.453781513 |
| ssc-miR-31 | ssc-mir-31 | 5p | AGGCAAGATGCTGGCATAGCTG | 51 | 3118 | 0.019501279 |
| ssc-miR-32 | ssc-mir-32 | 5p | TATTGCACATTACTAAGTTGC | 2 | 79 | 0.134831461 |
| ssc-miR-320 | ssc-mir-320 | 3p | AAAAGCTGGGTTGAGAGGGCGAA | 2942 | 321 | 8.918429003 |
| ssc-miR-323 | ssc-mir-323 | 3p | GCACATTACACGGTCGACCTCT | 5 | 1 | 1.363636364 |
| ssc-miR-324 | ssc-mir-324 | 5p | CGCATCCCCTAGGGCATTGGTGT | 3 | 42 | 0.25 |
| ssc-miR-325 | ssc-mir-325 | 5p | CCTAGTAGGTGTTCAGTAAGTGT |  |  |  |
| ssc-miR-326 | ssc-mir-326 | 3p | CCTCTGGGCCCTTCCTCCAG |  |  |  |
| ssc-miR-328 | ssc-mir-328 | 3p | CTGGCCCTCTCTGCCCTTCCGT | 2 | 2 | 1 |
| ssc-miR-331-5p | ssc-mir-331 | 5p | TCTAGGTATGGTCCCAGGGAT | 7 | 1 | 1.545454545 |
| ssc-miR-331-3p | ssc-mir-331 | 3p | GCCCCTGGGCCTATCCTAGAA | 4 | 27 | 0.378378378 |
| ssc-miR-335 | ssc-mir-335 | 5p | TCAAGAGCAATAACGAAAAATG | 45 | 74 | 0.654761905 |
| ssc-miR-338 | ssc-mir-338 | 3p | TCCAGCATCAGTGATTTTGTTG |  | 5 | 0.666666667 |
| ssc-miR-339-5p | ssc-mir-339-1 | 5p | TCCCTGTCCTCCAGGAGCTCAC | 185 | 1 | 17.72727273 |
| ssc-miR-339-3p | ssc-mir-339-1 | 3p | AGCTCCTCGAGGCCAGAGCCC |  |  |  |
| ssc-miR-339 | ssc-mir-339-2 | 5p | TCCCTGTCCTCCAGGAGCTCA | 185 | 1 | 17.72727273 |
| ssc-miR-340 | ssc-mir-340-1 | 5p | TTATAAAGCAATGAGACTGATT | 664 | 227 | 2.843881857 |
| ssc-miR-340 | ssc-mir-340-2 | 5p | TTATAAAGCAATGAGACTGATT | 664 | 227 | 2.843881857 |
| ssc-miR-342 | ssc-mir-342 | 3p | TCTCACACAGAAATCGCACCCGTCA | 5 | 9 | 0.789473684 |
| ssc-miR-345-5p | ssc-mir-345-1 | 5p | GCTGACTCCTAGTCCAGTGC | 2 | 2 | 1 |
| ssc-miR-345-3p | ssc-mir-345-1 | 3p | CCCTGAACTAGGGGTCTGGAG | 4 |  | 1.4 |
| ssc-miR-345-5p | ssc-mir-345-2 | 5p | GCTGACTCCTAGTCCAGTGC | 2 | 2 | 1 |
| ssc-miR-345-3p | ssc-mir-345-2 | 3p | CCCTGAACTAGGGGTCTGGAG | 4 |  | 1.4 |
| ssc-miR-34a | ssc-mir-34a | 5p | TGGCAGTGTCTTAGCTGGTTGT | 584 | 37 | 12.63829787 |
| ssc-miR-34c | ssc-mir-34c-1 | 5p | AGGCAGTGTAGTTAGCTGATTGC | 3 |  | 1.3 |
| ssc-miR-34c | ssc-mir-34c-2 | 5p | AGGCAGTGTAGTTAGCTGATTGC | 3 |  | 1.3 |
| ssc-miR-361-5p | ssc-mir-361 | 5p | TTATCAGAATCTCCAGGGGTAC | 12 | 704 | 0.030812325 |
| ssc-miR-361-3p | ssc-mir-361 | 3p | CCCCCAGGTGTGATTCTGATTTGC | 14 | 29 | 0.615384615 |
| ssc-miR-3613 | ssc-mir-3613 | 5p | TGTTGTACTTTTTTTTTTGT | 47 | 6 | 3.5625 |
| ssc-miR-362 | ssc-mir-362 | 5p | AATCCTTGGAACCTAGGTGTGAGTG | 18 | 8 | 1.555555556 |
| ssc-miR-363 | ssc-mir-363-1 | 3p | AATTGCACGGTATCCATCTGTAA |  |  |  |
| ssc-miR-363 | ssc-mir-363-2 | 3p | AATTGCACGGTATCCATCTGTAA |  |  |  |
| ssc-miR-365-3p | ssc-mir-365-1 | 3p | TAATGCCCCTAAAAATCCTTAT | 39 | 21 | 1.580645161 |
| ssc-miR-365-5p | ssc-mir-365-2 | 5p | GAGGGACTTTCAGGGGCAGCTGT | 4 | 3 | 1.076923077 |
| ssc-miR-365-3p | ssc-mir-365-2 | 3p | TAATGCCCCTAAAAATCCTTAT | 39 | 21 | 1.580645161 |
| ssc-miR-369 | ssc-mir-369 | 3p | AATAATACATGGTTGATCTTT |  | 11 | 0.476190476 |
| ssc-miR-370 | ssc-mir-370 | 3p | GCCTGCTGGGGTGGAACCTGGT |  |  |  |
| ssc-miR-374a-5p | ssc-mir-374a | 5p | TTATAATACAACCTGATAAGTG | 26 | 72 | 0.43902439 |
| ssc-miR-374a-3p | ssc-mir-374a | 3p | CTTATCAGGTTGTATTGTAATT | 220 | 89 | 2.323232323 |
| ssc-miR-374b-5p | ssc-mir-374b | 5p | ATATAATACAACCTGCTAAGTG | 28 | 6 | 2.375 |
| ssc-miR-374b-3p | ssc-mir-374b | 3p | CTTATCAGGTTGTATTATCATT | 12 | 34 | 0.5 |
| ssc-miR-376a-5p | ssc-mir-376a | 5p | GTAGATTCTCCTTCTATGAGTAC |  |  |  |
| ssc-miR-376a-3p | ssc-mir-376a | 3p | ATCATAGAGGAAAATCCACGT |  |  |  |
| ssc-miR-376b | ssc-mir-376b | 5p | GTGGCTATTCCTTCTATGTTTA |  |  |  |
| ssc-miR-376c | ssc-mir-376c | 5p | GTGGATATTCCTTCTATGTTTA |  |  |  |
| ssc-miR-378 | ssc-mir-378-1 | 3p | ACTGGACTTGGAGTCAGAAGGC | 4001 | 927 | 4.280683031 |
| ssc-miR-378 | ssc-mir-378-2 | 3p | ACTGGACTTGGAGTCAGAAGGC | 4001 | 927 | 4.280683031 |
| ssc-miR-381 | ssc-mir-381 | 5p | AGCGAGGTTGCCCTTTGTATATT |  |  |  |
| ssc-miR-382 | ssc-mir-382 | 5p | AAGTTGTTCGTGGTGGATTCG | 11 | 3 | 1.615384615 |
| ssc-miR-383 | ssc-mir-383 | 3p | CCACAGCACTGCCTGGTCAGA | 2 | 38 | 0.25 |
| ssc-miR-411 | ssc-mir-411 | 3p | ATGTAACACGGTCCACTAAC | 5 | 8 | 0.833333333 |
| ssc-miR-421-5p | ssc-mir-421 | 5p | CCTCATTAAATGTTTGTTGAATGA |  |  |  |
| ssc-miR-421-3p | ssc-mir-421 | 3p | ATCAACAGACATTAATTGGGCGC | 30 | 38 | 0.833333333 |
| ssc-miR-423-5p | ssc-mir-423 | 5p | TGAGGGGCAGAGAGCGAGACTTT | 594 | 2493 | 0.241310427 |
| ssc-miR-423-3p | ssc-mir-423 | 3p | AGCTCGGTCTGAGGCCCCTCAGT | 363 | 246 | 1.45703125 |
| ssc-miR-424-5p | ssc-mir-424 | 5p | CAGCAGCAATTCATGTTTTGAA | 57 | 2182 | 0.030565693 |
| ssc-miR-424-3p | ssc-mir-424 | 3p | CAAAACGTGAGGCGCTGCTAT | 6 |  | 1.6 |
| ssc-miR-425-5p | ssc-mir-425 | 5p | AATGACACGATCACTCCCGTTGA | 70 | 438 | 0.178571429 |
| ssc-miR-425-3p | ssc-mir-425 | 3p | ATCGGGAATGTCGTGTCCGCCC | 62 | 3 | 5.538461538 |
| ssc-miR-429 | ssc-mir-429 | 3p | TAATACTGTCTGGTAATGCCGT | 102 | 130 | 0.8 |
| ssc-miR-432-5p | ssc-mir-432 | 5p | TCTTGGAGTAGGTCATTGGGT | 3 |  | 1.3 |
| ssc-miR-432-3p | ssc-mir-432 | 3p | TGGATGGCTCCTCCATGGCT |  |  |  |
| ssc-miR-4331 | ssc-mir-4331 | 3p | TGTGGCTGTGGTGTAGGCCAGC | 29 | 2 | 3.25 |
| ssc-miR-4332 | ssc-mir-4332 | 3p | CACGGCCGCCGCCGGGCGCC | 12 | 10 | 1.1 |
| ssc-miR-4333 | ssc-mir-4333 | 5p | ATACCTGCATGTTAGTCTTTGGTTCT |  |  |  |
| ssc-miR-4334-5p | ssc-mir-4334 | 5p | CCCTGGAGTGACGGGGGTG |  |  |  |
| ssc-miR-4334-3p | ssc-mir-4334 | 3p | TCCCTGTCCTCCAGGAGCTC | 185 | 1 | 17.72727273 |
| ssc-miR-4335 | ssc-mir-4335 | 3p | GTGCCCAGCGCTGCAGGGCA |  |  |  |
| ssc-miR-4336 | ssc-mir-4336 | 5p | CAACTCTGTGGTTTCCTTTACTCATAG |  |  |  |
| ssc-miR-4337 | ssc-mir-4337 | 5p | AGGGTATATAAGCCTTCACTGG |  |  |  |
| ssc-miR-4338 | ssc-mir-4338 | 5p | ATGTTCAGTCTCAGTGGGAACC |  |  |  |
| ssc-miR-4339 | ssc-mir-4339 | 5p | GCTCTGAGCTGCCCCTCCTCGTCC |  |  |  |
| ssc-miR-450a | ssc-mir-450a | 5p | TTTTGCGATGTGTTCCTAATAT | 13 | 5 | 1.533333333 |
| ssc-miR-450b-5p | ssc-mir-450b | 5p | TTTTGCAATATGTTCCTGAATA | 14 | 40 | 0.48 |
| ssc-miR-450b-3p | ssc-mir-450b | 3p | TTGGGAACATTTTGCATCCAT |  |  |  |
| ssc-miR-450c-5p | ssc-mir-450c | 5p | TTTTGCGATGTGTTCCTAATAC | 123 | 6 | 8.3125 |
| ssc-miR-450c-3p | ssc-mir-450c | 3p | ATTGGGAACATTTTGCATTCGT | 3 | 1 | 1.181818182 |
| ssc-miR-451 | ssc-mir-451 | 5p | AAACCGTTACCATTACTGAGTT | 23 |  | 3.3 |
| ssc-miR-452 | ssc-mir-452 | 5p | AACTGTTTGCAGAGGAAACTGA |  |  |  |
| ssc-miR-455-5p | ssc-mir-455 | 5p | TATGTGCCTTTGGACTACATCG | 21 | 3 | 2.384615385 |
| ssc-miR-455-3p | ssc-mir-455 | 3p | GCAGTCCATGGGCATATACAC | 6 |  | 1.6 |
| ssc-miR-484 | ssc-mir-484 | 3p | CCCAGGGGGCGACCCAGGCT |  |  |  |
| ssc-miR-486 | ssc-mir-486-1 | 3p | TCCTGTACTGAGCTGCCCCGAG | 28 | 8 | 2.111111111 |
| ssc-miR-486 | ssc-mir-486-2 | 5p | TCCTGTACTGAGCTGCCCCGAG | 28 | 8 | 2.111111111 |
| ssc-miR-487b | ssc-mir-487b | 5p | GTGGTTATCCCTGTCCTGTTCG |  |  |  |
| ssc-miR-489 | ssc-mir-489 | 3p | AGTGACATCACATATACGGCGG | 114 | 158 | 0.738095238 |
| ssc-miR-490-5p | ssc-mir-490-1 | 5p | CCATGGATCCCCAGGTGGGT | 227 | 14 | 9.875 |
| ssc-miR-490-3p | ssc-mir-490-1 | 3p | CAACCTGGAGGACTCCATGCTG | 11 | 24 | 0.617647059 |
| ssc-miR-490 | ssc-mir-490-2 | 3p | CAACCTGGAGGACTCCATGCTG | 11 | 24 | 0.617647059 |
| ssc-miR-491 | ssc-mir-491 | 5p | AGTGGGGAACCCTTCCATGAGG |  | 10 | 0.5 |
| ssc-miR-493-5p | ssc-mir-493 | 5p | TTGTACATGGTAGGCTTTCATT |  |  |  |
| ssc-miR-493-3p | ssc-mir-493 | 3p | TGAAGGTCTACTGTGTGCCAGG |  |  |  |
| ssc-miR-494 | ssc-mir-494 | 5p | AGGTTGTCGTGTTGTCTTCTCT |  |  |  |
| ssc-miR-497 | ssc-mir-497 | 5p | CAGCAGCACACTGTGGTTTGT | 9 | 3 | 1.461538462 |
| ssc-miR-499-5p | ssc-mir-499 | 5p | TTAAGACTTGCAGTGATGTTT |  | 472 | 0.020746888 |
| ssc-miR-499-3p | ssc-mir-499 | 3p | AACATCACAGCAAGTCTGTGCT |  | 1 | 0.909090909 |
| ssc-miR-500 | ssc-mir-500 | 3p | ATGCACCTGGGCAAGGATTCT | 89 | 31 | 2.414634146 |
| ssc-miR-503 | ssc-mir-503 | 5p | TAGCAGCGGGAACAGTACTGCAG | 33 | 67 | 0.558441558 |
| ssc-miR-504 | ssc-mir-504 | 5p | AGACCCTGGTCTGCACTCTATCT |  |  |  |
| ssc-miR-505 | ssc-mir-505 | 3p | TCAACACTTGCTGGTTTCCTCT | 180 | 79 | 2.134831461 |
| ssc-miR-532-5p | ssc-mir-532 | 5p | CATGCCTTGAGTGTAGGACCGT | 600 | 564 | 1.06271777 |
| ssc-miR-532-3p | ssc-mir-532 | 3p | CCTCCCACACCCAAGGCTTGCA | 8 | 3 | 1.384615385 |
| ssc-miR-542-5p | ssc-mir-542 | 5p | TCGGGGATCATCATGTCACGA | 9 | 277 | 0.066202091 |
| ssc-miR-542-3p | ssc-mir-542 | 3p | TGTGACAGATTGATAACTGAAA | 69 | 1446 | 0.054258242 |
| ssc-miR-545-5p | ssc-mir-545 | 5p | TCAGTAAATGTTTATTGGATG | 43 | 19 | 1.827586207 |
| ssc-miR-545-3p | ssc-mir-545 | 3p | ATCAACAAACATTTATTGTGTG | 11 | 5 | 1.4 |
| ssc-miR-551a | ssc-mir-551a | 3p | GCGACCCACTCTTGGTTTCC | 2 |  | 1.2 |
| ssc-miR-574 | ssc-mir-574 | 3p | CACGCTCATGCACACACCCACA |  | 6 | 0.625 |
| ssc-miR-582 | ssc-mir-582 | 3p | TAACCGGTTGAACAACTGAACC | 7 | 1 | 1.545454545 |
| ssc-miR-615 | ssc-mir-615 | 3p | TCCGAGCCTGGGTCTCCCTCT |  |  |  |
| ssc-miR-628 | ssc-mir-628 | 5p | ATGCTGACATATTTACTAGAGG |  | 3 | 0.769230769 |
| ssc-miR-652 | ssc-mir-652 | 5p | ACAACCCTAGGAGAGGGTGCCATTCA |  |  |  |
| ssc-miR-664-5p | ssc-mir-664 | 5p | CAGGCTAGGAGAAGTGATTGGAT | 29 | 2 | 3.25 |
| ssc-miR-664-3p | ssc-mir-664 | 3p | TATTCATTTATCTCCCAGCCTACA |  |  |  |
| ssc-miR-671-5p | ssc-mir-671 | 5p | AGGAAGCCCTGGAGGGGCTGGAGG |  | 52 | 0.161290323 |
| ssc-miR-671-3p | ssc-mir-671 | 3p | TCCGGTTCTCAGGGCTCCACC |  | 1 | 0.909090909 |
| ssc-miR-676-5p | ssc-mir-676-1 | 5p | CTCTTCAATCTCAGGACTCGCA |  |  |  |
| ssc-miR-676-3p | ssc-mir-676-1 | 3p | CCGTCCTAAGGTTGTTGAGTT |  |  |  |
| ssc-miR-676-3p | ssc-mir-676-2 | 3p | CCGTCCTAAGGTTGTTGAGTT |  |  |  |
| ssc-miR-7 | ssc-mir-7-1 | 5p | TGGAAGACTAGTGATTTTGTTGTT | 4286 | 489 | 8.609218437 |
| ssc-miR-7 | ssc-mir-7-2 | 5p | TGGAAGACTAGTGATTTTGTTGTT | 4286 | 489 | 8.609218437 |
| ssc-miR-708-5p | ssc-mir-708 | 5p | AAGGAGCTTACAATCTAGCTGGG |  | 9 | 0.526315789 |
| ssc-miR-708-3p | ssc-mir-708 | 3p | CAACTAGACTGTGAGCTTCTAGA | 2 | 1 | 1.090909091 |
| ssc-miR-744 | ssc-mir-744 | 5p | TGCGGGGCTAGGGCTAACAGCA | 119 | 42 | 2.480769231 |
| ssc-miR-758 | ssc-mir-758 | 3p | TTTGTGACCTGGTCCACTAAC |  | 2 | 0.833333333 |
| ssc-miR-769-5p | ssc-mir-769 | 5p | TGAGACCTCTGGGTTCTGAGC | 316 | 55 | 5.015384615 |
| ssc-miR-769-3p | ssc-mir-769 | 3p | CTGGGATCTCTGGGGTCTTGGTT | 16 | 1 | 2.363636364 |
| ssc-miR-874 | ssc-mir-874 | 3p | CTGCCCTGGCCCGAGGGACCGAC |  |  |  |
| ssc-miR-885-5p | ssc-mir-885 | 5p | TCCATTACACTACCCTGCCTCT | 4 |  | 1.4 |
| ssc-miR-885-3p | ssc-mir-885 | 3p | AGGCAGCGGGGTGTAGTGGAT | 9 | 1 | 1.727272727 |
| ssc-miR-9-1 | ssc-mir-9-1 | 5p | TCTTTGGTTATCTAGCTGTATGA | 91 | 68 | 1.294871795 |
| ssc-miR-9-2 | ssc-mir-9-2 | 5p | TCTTTGGTTATCTAGCTGTATGA | 91 | 68 | 1.294871795 |
| ssc-miR-9 | ssc-mir-9-3 | 5p | TCTTTGGTTATCTAGCTGTATG | 91 | 68 | 1.294871795 |
| ssc-miR-92a | ssc-mir-92a-1 | 3p | TATTGCACTTGTCCCGGCCTGT | 4317 | 1148 | 3.736614853 |
| ssc-miR-92a | ssc-mir-92a-2 | 3p | TATTGCACTTGTCCCGGCCTGT | 4317 | 1148 | 3.736614853 |
| ssc-miR-92b-5p | ssc-mir-92b | 5p | AGGGACGGGACGCGGTGCAGTGTT | 16 | 1 | 2.363636364 |
| ssc-miR-92b-3p | ssc-mir-92b | 3p | TATTGCACTCGTCCCGGCCTCC | 1644 | 133 | 11.56643357 |
| ssc-miR-935 | ssc-mir-935 | 3p | CCAGTTACCGCTTCCGCTACCGC |  |  |  |
| ssc-miR-95 | ssc-mir-95 | 3p | TTCAACGGGTATTTATTGAGCA | 2 | 1 | 1.090909091 |
| ssc-miR-98 | ssc-mir-98 | 5p | TGAGGTAGTAAGTTGTATTGTT | 6455 | 2376 | 2.709555742 |
| ssc-miR-99a | ssc-mir-99a | 5p | AACCCGTAGATCCGATCTTGTG | 24 | 307 | 0.107255521 |
| ssc-miR-99b | ssc-mir-99b | 5p | CACCCGTAGAACCGACCTTGCG | 58 | 132 | 0.478873239 |
| ssc-miR-novel-chr10_5263 | ssc-mir-novel-chr10_5263 | 3p | CACATGGAGTCGCTGTTACAGCT |  |  |  |
| ssc-miR-novel-chr10_5287 | ssc-mir-novel-chr10_5287 | 3p | ACTAGTGCTGTCAGAGACGCC |  |  |  |
| ssc-miR-novel-chr10_5425 | ssc-mir-novel-chr10_5425 | 3p | TACGAATTTCAGGAATACAGC |  | 3 | 0.769230769 |
| ssc-miR-novel-chr10_5436 | ssc-mir-novel-chr10_5436 | 5p | GAGCTGTGATGAGAATCCTCTGAGC |  |  |  |
| ssc-miR-novel-chr10_5472 | ssc-mir-novel-chr10_5472 | 5p | ACGAGAAAGGAGGAGGG |  |  |  |
| ssc-miR-novel-chr10_5540 | ssc-mir-novel-chr10_5540 | 5p | GCAGGAACTTGTGAGTCTCCT |  | 1 | 0.909090909 |
| ssc-miR-novel-chr10_5541 | ssc-mir-novel-chr10_5541 | 3p | TGGTGGTTTACAAAGTAATTC |  |  |  |
| ssc-miR-novel-chr10_5602 | ssc-mir-novel-chr10_5602 | 5p | TGAGTGTGTGTGTGTGAGTGTGTGT | 27 | 1 | 3.363636364 |
| ssc-miR-novel-chr10_5614 | ssc-mir-novel-chr10_5614 | 5p | GCGGGCCCACGGGGGCC | 3 | 52 | 0.209677419 |
| ssc-miR-novel-chr10_5682 | ssc-mir-novel-chr10_5682 | 3p | CCGGCCGGGCGCGAGCC | 10 | 3 | 1.538461538 |
| ssc-miR-novel-chr10_5914 | ssc-mir-novel-chr10_5914 | 3p | CACATGGAGTCGCTGTTACAGCT |  |  |  |
| ssc-miR-novel-chr10_5917 | ssc-mir-novel-chr10_5917 | 5p | TTCAGGGTCCAGGATTGCTATAG | 2 | 1 | 1.090909091 |
| ssc-miR-novel-chr10_6036 | ssc-mir-novel-chr10_6036 | 3p | TACGAATTTCAGGAATACAGC |  | 3 | 0.769230769 |
| ssc-miR-novel-chr10_6047 | ssc-mir-novel-chr10_6047 | 5p | TGGATGGGAGTCGGTGGGCAGC |  |  |  |
| ssc-miR-novel-chr10_6071 | ssc-mir-novel-chr10_6071 | 5p | GTGGATTTTTGGAGTTGGG |  | 2 | 0.833333333 |
| ssc-miR-novel-chr10_6138 | ssc-mir-novel-chr10_6138 | 3p | TGGTGGTTTACAAAGTAATTC |  |  |  |
| ssc-miR-novel-chr10_6327 | ssc-mir-novel-chr10_6327 | 5p | AGTGTGTGGGCGCCGGACGCT |  | 17 | 0.37037037 |
| ssc-miR-novel-chr11_6393 | ssc-mir-novel-chr11_6393 | 3p | CGGGGCCGGGGGTGGGG |  |  |  |
| ssc-miR-novel-chr11_6456 | ssc-mir-novel-chr11_6456 | 5p | CTCGCGGGAAAACTTGTATGTG |  |  |  |
| ssc-miR-novel-chr11_6577 | ssc-mir-novel-chr11_6577 | 3p | CTGAATGGAATTGTCTCAGCCT | 30 |  | 4 |
| ssc-miR-novel-chr11_6750 | ssc-mir-novel-chr11_6750 | 5p | CAGGGTCGGGCCTGGTTA | 16 | 547 | 0.046678636 |
| ssc-miR-novel-chr11_6755 | ssc-mir-novel-chr11_6755 | 3p | TGTCCCACCAGAGTCGCCA |  | 7 | 0.588235294 |
| ssc-miR-novel-chr11_6826 | ssc-mir-novel-chr11_6826 | 5p | ACTTTCCCGGGATTTGGAGCG | 3 |  | 1.3 |
| ssc-miR-novel-chr11_6850 | ssc-mir-novel-chr11_6850 | 5p | TCCTGGAGGACGTGCTGTGC | 4 |  | 1.4 |
| ssc-miR-novel-chr11_6942 | ssc-mir-novel-chr11_6942 | 3p | TGACTCACTCTGTTGTGCAGC |  |  |  |
| ssc-miR-novel-chr11_7050 | ssc-mir-novel-chr11_7050 | 3p | AGCGTGGGCTGCGGGCCGCT |  |  |  |
| ssc-miR-novel-chr11_7060 | ssc-mir-novel-chr11_7060 | 3p | CGCGCTTAGGGTTCCCGGCAT |  |  |  |
| ssc-miR-novel-chr11_7116 | ssc-mir-novel-chr11_7116 | 3p | CTGAATGGAATTGTCTCAGCCT | 30 |  | 4 |
| ssc-miR-novel-chr11_7251 | ssc-mir-novel-chr11_7251 | 5p | ACATTTAAGGAGGTGCTTGCT | 4 | 1 | 1.272727273 |
| ssc-miR-novel-chr12_7407 | ssc-mir-novel-chr12_7407 | 3p | CGGCGGCGGCGGCGACT | 4 | 47 | 0.245614035 |
| ssc-miR-novel-chr12_7455 | ssc-mir-novel-chr12_7455 | 3p | TGCTTGGACAGTGCCTGGCCTGC |  | 1 | 0.909090909 |
| ssc-miR-novel-chr12_7490 | ssc-mir-novel-chr12_7490 | 5p | TTTGCAGTAACAGGTGTGAAC |  | 14 | 0.416666667 |
| ssc-miR-novel-chr12_7511 | ssc-mir-novel-chr12_7511 | 3p | CTTTGTCTTAATCTCTGTGGTT |  |  |  |
| ssc-miR-novel-chr12_7711 | ssc-mir-novel-chr12_7711 | 3p | TGCGTCTCACCTGCCTGACAGG |  |  |  |
| ssc-miR-novel-chr12_7741 | ssc-mir-novel-chr12_7741 | 3p | GGAGCGGGCGGGCGGTC | 4 |  | 1.4 |
| ssc-miR-novel-chr12_7775 | ssc-mir-novel-chr12_7775 | 3p | TGAAAGGACGTAAAACAGGCCC |  |  |  |
| ssc-miR-novel-chr12_7779 | ssc-mir-novel-chr12_7779 | 3p | TGAAAGGACGTAAAACAGGCCC |  |  |  |
| ssc-miR-novel-chr12_7811 | ssc-mir-novel-chr12_7811 | 3p | TGCGTCTCACCTGCCTGACAGG |  |  |  |
| ssc-miR-novel-chr12_7898 | ssc-mir-novel-chr12_7898 | 5p | TCCTGATTTGCCGAGGCCTGAGGG | 4 |  | 1.4 |
| ssc-miR-novel-chr12_7955 | ssc-mir-novel-chr12_7955 | 3p | CGGCTGCGAGCAGACGGTC |  |  |  |
| ssc-miR-novel-chr12_7961 | ssc-mir-novel-chr12_7961 | 3p | CAGTGCAATAGTATTGTCAAAG | 2126 | 191 | 10.62686567 |
| ssc-miR-novel-chr12_7963 | ssc-mir-novel-chr12_7963 | 3p | TAGTGCAATATTGCTTATAGGGT | 141 | 43 | 2.849056604 |
| ssc-miR-novel-chr12_7964 | ssc-mir-novel-chr12_7964 | 5p | TAGTGCAATATTGCTTATAGGGT | 141 | 43 | 2.849056604 |
| ssc-miR-novel-chr12_8144 | ssc-mir-novel-chr12_8144 | 5p | GACCATGGCTGTAGACTGTTA | 8 | 1 | 1.636363636 |
| ssc-miR-novel-chr12_8161 | ssc-mir-novel-chr12_8161 | 3p | CAGGCCTGGAGCTCTGCCTGCT | 38 |  | 4.8 |
| ssc-miR-novel-chr12_8220 | ssc-mir-novel-chr12_8220 | 5p | GGTGAGCACTCTGGACT |  | 4 | 0.714285714 |
| ssc-miR-novel-chr12_8221 | ssc-mir-novel-chr12_8221 | 3p | CGGGACCTGGTGCCCCTGTCGC |  |  |  |
| ssc-miR-novel-chr12_8235 | ssc-mir-novel-chr12_8235 | 3p | CACACACAGATCATTTCGTAGA |  |  |  |
| ssc-miR-novel-chr12_8265 | ssc-mir-novel-chr12_8265 | 3p | TTACAGTATTAGTCGCTTTT | 2 | 51 | 0.196721311 |
| ssc-miR-novel-chr12_8282 | ssc-mir-novel-chr12_8282 | 5p | TATCCCCATGGAGTCTGTTGCC | 67 |  | 7.7 |
| ssc-miR-novel-chr12_8290 | ssc-mir-novel-chr12_8290 | 5p | TATCCCCATGGAGTCTGTTGCC | 67 |  | 7.7 |
| ssc-miR-novel-chr12_8302 | ssc-mir-novel-chr12_8302 | 5p | GGTGAGCACTCTGGACT |  | 4 | 0.714285714 |
| ssc-miR-novel-chr12_8361 | ssc-mir-novel-chr12_8361 | 5p | CAGGGCACCGGCCTCTGCGTGGG |  |  |  |
| ssc-miR-novel-chr12_8369 | ssc-mir-novel-chr12_8369 | 5p | TCAACAAAATCACTGATGCTGGA |  | 3 | 0.769230769 |
| ssc-miR-novel-chr12_8374 | ssc-mir-novel-chr12_8374 | 3p | CAGGGCTTGGGGAGCAGAGAGA | 5 | 1 | 1.363636364 |
| ssc-miR-novel-chr12_8420 | ssc-mir-novel-chr12_8420 | 3p | TGGCTCAGCTCAGCAGGAG |  | 9 | 0.526315789 |
| ssc-miR-novel-chr12_8530 | ssc-mir-novel-chr12_8530 | 3p | TGCTCTGGAGTCAAGTCAGGA | 2 |  | 1.2 |
| ssc-miR-novel-chr12_8533 | ssc-mir-novel-chr12_8533 | 5p | CAGTGAATGGAGCCCTGAGA |  |  |  |
| ssc-miR-novel-chr12_8560 | ssc-mir-novel-chr12_8560 | 3p | TACAGCTTCTTGGAGTTCCCGTGT |  |  |  |
| ssc-miR-novel-chr12_8568 | ssc-mir-novel-chr12_8568 | 3p | CAGGACATGGAGAAAGGC |  |  |  |
| ssc-miR-novel-chr12_8591 | ssc-mir-novel-chr12_8591 | 5p | ACGGGATTGTAAAGGCAGAGCG |  |  |  |
| ssc-miR-novel-chr12_8594 | ssc-mir-novel-chr12_8594 | 3p | ACTGGGAGCAATGGAACGGCGA | 5 | 1 | 1.363636364 |
| ssc-miR-novel-chr12_8618 | ssc-mir-novel-chr12_8618 | 3p | CACGTGCGTGCTGGTATCTGG |  | 1 | 0.909090909 |
| ssc-miR-novel-chr12_8656 | ssc-mir-novel-chr12_8656 | 5p | TTTACGTCCTTTCACCTAGTTT |  |  |  |
| ssc-miR-novel-chr12_8665 | ssc-mir-novel-chr12_8665 | 5p | TCAGGATGAGACTCAAGGGGC |  |  |  |
| ssc-miR-novel-chr12_8771 | ssc-mir-novel-chr12_8771 | 5p | ACTGGACTTGGAGTCAGAAG | 76 | 12 | 3.909090909 |
| ssc-miR-novel-chr12_8979 | ssc-mir-novel-chr12_8979 | 5p | AGCTGGGCTGCTGTTCTCGAGGT | 4 |  | 1.4 |
| ssc-miR-novel-chr12_8980 | ssc-mir-novel-chr12_8980 | 3p | AGCTGGGCTGCTGTTCTCGAGGT | 4 |  | 1.4 |
| ssc-miR-novel-chr12_8997 | ssc-mir-novel-chr12_8997 | 5p | CACGGGTTCGATCCCTGGTGTGGGC |  |  |  |
| ssc-miR-novel-chr12_9131 | ssc-mir-novel-chr12_9131 | 5p | TGGGGTGGGGGCGTCGGGCC |  |  |  |
| ssc-miR-novel-chr12_9188 | ssc-mir-novel-chr12_9188 | 3p | AGCGCGGCTGGGCCTCCCCGA |  |  |  |
| ssc-miR-novel-chr13_10019 | ssc-mir-novel-chr13_10019 | 3p | GCGACCCATACTTGGTTTCAGA |  |  |  |
| ssc-miR-novel-chr13_10041 | ssc-mir-novel-chr13_10041 | 3p | TTGGAGTTTTGGAGCTGGG |  | 2 | 0.833333333 |
| ssc-miR-novel-chr13_10108 | ssc-mir-novel-chr13_10108 | 5p | AGGAGCAGGAGTCTGGGCTGAGG | 2 |  | 1.2 |
| ssc-miR-novel-chr13_10170 | ssc-mir-novel-chr13_10170 | 5p | AAGCAGGATTTAGACTACAATAT | 21 | 7 | 1.823529412 |
| ssc-miR-novel-chr13_10187 | ssc-mir-novel-chr13_10187 | 3p | AGAGGGCTGTGGGAGAGA | 10 |  | 2 |
| ssc-miR-novel-chr13_10484 | ssc-mir-novel-chr13_10484 | 5p | ACAGTGGCTGTGGCTCG | 9 | 2 | 1.583333333 |
| ssc-miR-novel-chr13_10658 | ssc-mir-novel-chr13_10658 | 5p | TCAGTAACAAAGATTCATCCTTG |  |  |  |
| ssc-miR-novel-chr13_10858 | ssc-mir-novel-chr13_10858 | 3p | CCTTCTCTGTAGGCCAGGGGCCCAG |  |  |  |
| ssc-miR-novel-chr13_10861 | ssc-mir-novel-chr13_10861 | 5p | TTCAAGTAACCCAGGATAGGCT | 671 | 1483 | 0.4561286 |
| ssc-miR-novel-chr13_10908 | ssc-mir-novel-chr13_10908 | 3p | GGCCGGGCCCGCCCCCGC |  | 4 | 0.714285714 |
| ssc-miR-novel-chr13_10965 | ssc-mir-novel-chr13_10965 | 5p | GCTGTCTCTGTATGAATGTG |  |  |  |
| ssc-miR-novel-chr13_10983 | ssc-mir-novel-chr13_10983 | 5p | TGGGTGAGAGCACAGCAGAACT |  | 1 | 0.909090909 |
| ssc-miR-novel-chr13_11069 | ssc-mir-novel-chr13_11069 | 5p | TCTGGCTGTGGTGTAGA |  |  |  |
| ssc-miR-novel-chr13_11147 | ssc-mir-novel-chr13_11147 | 3p | AATCTGTCCACATATGGTGGTA |  |  |  |
| ssc-miR-novel-chr13_11172 | ssc-mir-novel-chr13_11172 | 3p | CCCTGTCTTTTGCTTCTCCTTT | 3 |  | 1.3 |
| ssc-miR-novel-chr13_11226 | ssc-mir-novel-chr13_11226 | 3p | CTTCCTACCCAGTCCGGC |  |  |  |
| ssc-miR-novel-chr13_11289 | ssc-mir-novel-chr13_11289 | 5p | AGGGTCTAGGTCGCAGATACAGC | 2 | 3 | 0.923076923 |
| ssc-miR-novel-chr13_11457 | ssc-mir-novel-chr13_11457 | 5p | TCTGGCTTTAATCTCTGTCTTG | 11 | 3 | 1.615384615 |
| ssc-miR-novel-chr13_11481 | ssc-mir-novel-chr13_11481 | 5p | TCTGGCTGTGGTGTAGACC | 11 | 1 | 1.909090909 |
| ssc-miR-novel-chr13_11522 | ssc-mir-novel-chr13_11522 | 3p | CTGGTCTGTGTCTTTGTGAGAGC |  |  |  |
| ssc-miR-novel-chr13_11595 | ssc-mir-novel-chr13_11595 | 5p | AAGCCAGAGTCAGGGGACACTGT |  |  |  |
| ssc-miR-novel-chr13_11601 | ssc-mir-novel-chr13_11601 | 5p | TATCTGCCTGTATATATGCCT | 6 |  | 1.6 |
| ssc-miR-novel-chr13_11602 | ssc-mir-novel-chr13_11602 | 3p | TATCTGCCTGTATATATGCCT | 6 |  | 1.6 |
| ssc-miR-novel-chr13_11720 | ssc-mir-novel-chr13_11720 | 3p | TGGGTGGCAAATGGTGGGTTTGA | 3 | 1 | 1.181818182 |
| ssc-miR-novel-chr13_11871 | ssc-mir-novel-chr13_11871 | 5p | GCGCAGCACATCATGGTTTA |  |  |  |
| ssc-miR-novel-chr13_11899 | ssc-mir-novel-chr13_11899 | 5p | ATTCTGTTAGAAAAATGCAAGA |  | 2 | 0.833333333 |
| ssc-miR-novel-chr13_11944 | ssc-mir-novel-chr13_11944 | 5p | TTTACATTGATTTCATATTGCT |  |  |  |
| ssc-miR-novel-chr13_12020 | ssc-mir-novel-chr13_12020 | 3p | AGACCTGGATTCATCAGCC |  |  |  |
| ssc-miR-novel-chr13_12094 | ssc-mir-novel-chr13_12094 | 3p | GCCTTGGCCTCTGCACCTGGTC |  |  |  |
| ssc-miR-novel-chr13_12101 | ssc-mir-novel-chr13_12101 | 5p | AAGGAGCTGAGGACGGAGAAGGA | 17 | 4 | 1.928571429 |
| ssc-miR-novel-chr13_9205 | ssc-mir-novel-chr13_9205 | 3p | ATTCTTTGCTGGATGGCATT |  |  |  |
| ssc-miR-novel-chr13_9254 | ssc-mir-novel-chr13_9254 | 5p | GAAATGAAGAACCCAGA |  | 1 | 0.909090909 |
| ssc-miR-novel-chr13_9348 | ssc-mir-novel-chr13_9348 | 5p | CTTGGAATTTTGCAGTGTCCA |  | 2 | 0.833333333 |
| ssc-miR-novel-chr13_9358 | ssc-mir-novel-chr13_9358 | 5p | AAGGTGGCTAATGCTTAGTGAGT |  |  |  |
| ssc-miR-novel-chr13_9430 | ssc-mir-novel-chr13_9430 | 5p | TTTGCTCTGCTCCTGCCACAT |  |  |  |
| ssc-miR-novel-chr13_9441 | ssc-mir-novel-chr13_9441 | 3p | GAGCGGGGCGCGCGGTCG |  | 2 | 0.833333333 |
| ssc-miR-novel-chr13_9442 | ssc-mir-novel-chr13_9442 | 5p | GAGCGGGGCGCGCGGTCG | 6 | 2 | 1.333333333 |
| ssc-miR-novel-chr13_9467 | ssc-mir-novel-chr13_9467 | 3p | TGTCCAAGACTGCAGGTCAGT |  |  |  |
| ssc-miR-novel-chr13_9486 | ssc-mir-novel-chr13_9486 | 3p | CCAGAGCACTCAAAAAGATGGC |  |  |  |
| ssc-miR-novel-chr13_9502 | ssc-mir-novel-chr13_9502 | 5p | TCCCACAGGGGGACGTGAGGCAG |  |  |  |
| ssc-miR-novel-chr13_9520 | ssc-mir-novel-chr13_9520 | 5p | TCCCACAGGGGGACGTGAGGCAG |  |  |  |
| ssc-miR-novel-chr13_9841 | ssc-mir-novel-chr13_9841 | 3p | CTGCGTCGGAGGGCGGCCGGAA |  |  |  |
| ssc-miR-novel-chr13_9887 | ssc-mir-novel-chr13_9887 | 3p | CAGGCATGAAGTCAGGTTAG |  |  |  |
| ssc-miR-novel-chr13_9979 | ssc-mir-novel-chr13_9979 | 3p | TTGTCAGTAGTTGCATGCAGGGA |  | 1 | 0.909090909 |
| ssc-miR-novel-chr13_9996 | ssc-mir-novel-chr13_9996 | 5p | AGGCATGGTCAAGAGGAAATC |  |  |  |
| ssc-miR-novel-chr14_12177 | ssc-mir-novel-chr14_12177 | 3p | TTTGTCTGTGGTGTAGGCCCGC | 23 | 5 | 2.2 |
| ssc-miR-novel-chr14_12179 | ssc-mir-novel-chr14_12179 | 3p | TTTGTCTGTGGTGTAGGCCCGC | 23 | 5 | 2.2 |
| ssc-miR-novel-chr14_12181 | ssc-mir-novel-chr14_12181 | 3p | TTTGTCTGTGGTGTAGGCCCGC | 23 | 5 | 2.2 |
| ssc-miR-novel-chr14_12274 | ssc-mir-novel-chr14_12274 | 5p | CAGATGGAGGCGTGGGT | 9 | 15 | 0.76 |
| ssc-miR-novel-chr14_12484 | ssc-mir-novel-chr14_12484 | 5p | AGCCAGGATTGTGGTGGCAGCCAG |  |  |  |
| ssc-miR-novel-chr14_12513 | ssc-mir-novel-chr14_12513 | 3p | TGGAAGGACACAGGAAGCCT | 3 |  | 1.3 |
| ssc-miR-novel-chr14_12542 | ssc-mir-novel-chr14_12542 | 5p | TTCCAGGGAAGAAAGGAGGAAC | 2 |  | 1.2 |
| ssc-miR-novel-chr14_12589 | ssc-mir-novel-chr14_12589 | 3p | AGCAGGACTGGCAGCTCTGGGCCTC |  |  |  |
| ssc-miR-novel-chr14_12636 | ssc-mir-novel-chr14_12636 | 5p | GTAGTACTTCTTGTTTGGATGCA | 2 |  | 1.2 |
| ssc-miR-novel-chr14_12638 | ssc-mir-novel-chr14_12638 | 5p | GTAGTACTTCTTGTTTGGATGCA | 2 |  | 1.2 |
| ssc-miR-novel-chr14_12801 | ssc-mir-novel-chr14_12801 | 3p | CTCTCTGACAGTCTCTGAAAGC |  |  |  |
| ssc-miR-novel-chr14_12811 | ssc-mir-novel-chr14_12811 | 3p | TGGGCTGTTCTTTTGTCTCTGAG |  |  |  |
| ssc-miR-novel-chr14_12851 | ssc-mir-novel-chr14_12851 | 3p | GACAGATCTCTCCTCCCACAGC |  |  |  |
| ssc-miR-novel-chr14_12856 | ssc-mir-novel-chr14_12856 | 5p | ATCTGGGATCTGCGGCCCCTGC |  |  |  |
| ssc-miR-novel-chr14_12862 | ssc-mir-novel-chr14_12862 | 5p | AAGGAAAGAATTCTAGTGGGG |  |  |  |
| ssc-miR-novel-chr14_12918 | ssc-mir-novel-chr14_12918 | 5p | CAGGAAACACTGGTGGAGG | 111 | 99 | 1.110091743 |
| ssc-miR-novel-chr14_13301 | ssc-mir-novel-chr14_13301 | 3p | CCAAACCAGTTGTGCCTGTAGA |  |  |  |
| ssc-miR-novel-chr14_13321 | ssc-mir-novel-chr14_13321 | 3p | CTGGAACTTGGCTGTGTCTCT |  |  |  |
| ssc-miR-novel-chr14_13411 | ssc-mir-novel-chr14_13411 | 3p | CGGGACGGGTGTCGGGG | 20 | 8 | 1.666666667 |
| ssc-miR-novel-chr14_13412 | ssc-mir-novel-chr14_13412 | 5p | CGGGACGGGTGTCGGGG | 20 | 8 | 1.666666667 |
| ssc-miR-novel-chr14_13435 | ssc-mir-novel-chr14_13435 | 3p | AGCTCCTCGAGGCCAGA |  |  |  |
| ssc-miR-novel-chr14_13436 | ssc-mir-novel-chr14_13436 | 5p | AGCTCCTCGAGGCCAGA |  |  |  |
| ssc-miR-novel-chr14_13460 | ssc-mir-novel-chr14_13460 | 3p | TCTATCGGGTGTACCTGTCCT | 3 |  | 1.3 |
| ssc-miR-novel-chr14_13655 | ssc-mir-novel-chr14_13655 | 5p | TGAAGGCTGGCCTAGAAATCT |  |  |  |
| ssc-miR-novel-chr14_13668 | ssc-mir-novel-chr14_13668 | 3p | TAAACTGAGGCCTCTGTGGGGC | 4 |  | 1.4 |
| ssc-miR-novel-chr14_13772 | ssc-mir-novel-chr14_13772 | 3p | TTCTCCAAGGACACTCTGTCTCT | 4 |  | 1.4 |
| ssc-miR-novel-chr14_13888 | ssc-mir-novel-chr14_13888 | 3p | CAGTGCAATGATATTGTCAAAGC | 595 | 58 | 8.897058824 |
| ssc-miR-novel-chr14_14550 | ssc-mir-novel-chr14_14550 | 3p | GAGGTTAACGGAGTGACA | 14 | 1 | 2.181818182 |
| ssc-miR-novel-chr14_14572 | ssc-mir-novel-chr14_14572 | 3p | TGGCACCAGCACTGGCGGTGGC |  | 9 | 0.526315789 |
| ssc-miR-novel-chr14_14582 | ssc-mir-novel-chr14_14582 | 3p | GTGGTTAAGGCTTTGGA |  | 16 | 0.384615385 |
| ssc-miR-novel-chr14_14584 | ssc-mir-novel-chr14_14584 | 3p | CAGACCCTGAGCTGCCTCTAGA | 3 | 2 | 1.083333333 |
| ssc-miR-novel-chr14_14614 | ssc-mir-novel-chr14_14614 | 3p | AGGAGTGTGGAAGGAGTG |  |  |  |
| ssc-miR-novel-chr15_14859 | ssc-mir-novel-chr15_14859 | 3p | CTGCTCTCCTGTCTCCGCTCAGG |  |  |  |
| ssc-miR-novel-chr15_14871 | ssc-mir-novel-chr15_14871 | 3p | TGCATGACTGTAGAAAACCTGT |  |  |  |
| ssc-miR-novel-chr15_14873 | ssc-mir-novel-chr15_14873 | 3p | TGCATGACTGTAGAAAACCTGT |  |  |  |
| ssc-miR-novel-chr15_14905 | ssc-mir-novel-chr15_14905 | 3p | CTTCTGTGATGGTGAACTGAGA |  |  |  |
| ssc-miR-novel-chr15_14929 | ssc-mir-novel-chr15_14929 | 3p | CTGGCTGGAGAACTTCTACATG |  |  |  |
| ssc-miR-novel-chr15_15025 | ssc-mir-novel-chr15_15025 | 3p | GAGCGTAGTGTTCGCCGGCATA |  |  |  |
| ssc-miR-novel-chr15_15523 | ssc-mir-novel-chr15_15523 | 3p | TTTGTTCGTTCGGCTCGCGTGA | 337 | 58 | 5.102941176 |
| ssc-miR-novel-chr15_15531 | ssc-mir-novel-chr15_15531 | 3p | AGCCCGGCCGTACCCTGCAGG |  |  |  |
| ssc-miR-novel-chr15_15607 | ssc-mir-novel-chr15_15607 | 3p | GAGGCGGCCCCGGCCCGGTAT |  |  |  |
| ssc-miR-novel-chr15_15633 | ssc-mir-novel-chr15_15633 | 3p | TCAGCAGGTCAGGTTCCATAAT |  | 4 | 0.714285714 |
| ssc-miR-novel-chr15_15673 | ssc-mir-novel-chr15_15673 | 3p | GCATTGGGGGTTCAGGGG | 12 | 15 | 0.88 |
| ssc-miR-novel-chr15_15705 | ssc-mir-novel-chr15_15705 | 3p | TCTTGGACCCCTTCTCACAGGA |  |  |  |
| ssc-miR-novel-chr15_15729 | ssc-mir-novel-chr15_15729 | 3p | TCTATCGGGTGTACCTGTCCT | 3 |  | 1.3 |
| ssc-miR-novel-chr15_15788 | ssc-mir-novel-chr15_15788 | 3p | TCCCAGCTGGTCATTAATCCT |  | 3 | 0.769230769 |
| ssc-miR-novel-chr15_15866 | ssc-mir-novel-chr15_15866 | 3p | TTGTTCTGGTTGATGGGTGGGA |  |  |  |
| ssc-miR-novel-chr15_16235 | ssc-mir-novel-chr15_16235 | 5p | ATGGGATAATCACACCCCTGGGG |  |  |  |
| ssc-miR-novel-chr15_16275 | ssc-mir-novel-chr15_16275 | 5p | AGCCGGCGGCGGCGGCGA |  | 24 | 0.294117647 |
| ssc-miR-novel-chr15_16277 | ssc-mir-novel-chr15_16277 | 5p | AGCCGGCGGCGGCGGCGA |  | 24 | 0.294117647 |
| ssc-miR-novel-chr15_16436 | ssc-mir-novel-chr15_16436 | 3p | TTCGCGGCCCGCGCTGGGAGC |  |  |  |
| ssc-miR-novel-chr15_16518 | ssc-mir-novel-chr15_16518 | 3p | GGGCGGACTGTTAAACCG |  |  |  |
| ssc-miR-novel-chr15_16730 | ssc-mir-novel-chr15_16730 | 3p | TCAGCAGGTCAGGTTCCATAAT |  | 4 | 0.714285714 |
| ssc-miR-novel-chr15_16769 | ssc-mir-novel-chr15_16769 | 5p | TGCAGATGATGTGAGAGA |  |  |  |
| ssc-miR-novel-chr16_16851 | ssc-mir-novel-chr16_16851 | 3p | AGAGCTGAGGGCAGAGTCCAGGA |  |  |  |
| ssc-miR-novel-chr16_16917 | ssc-mir-novel-chr16_16917 | 3p | TTGGTGTACACTGGAATAGCT | 37 | 83 | 0.505376344 |
| ssc-miR-novel-chr16_16920 | ssc-mir-novel-chr16_16920 | 5p | ATGGACCACAAACTCAAATT |  |  |  |
| ssc-miR-novel-chr16_16922 | ssc-mir-novel-chr16_16922 | 5p | TGGTCTAGCGGTTAGGA |  | 11 | 0.476190476 |
| ssc-miR-novel-chr16_17112 | ssc-mir-novel-chr16_17112 | 5p | GGTGATGATGACGATGAAGCTGAAA |  | 7 | 0.588235294 |
| ssc-miR-novel-chr16_17181 | ssc-mir-novel-chr16_17181 | 3p | TCTGCCTGTAGGAATGCTGTAGG |  |  |  |
| ssc-miR-novel-chr16_17182 | ssc-mir-novel-chr16_17182 | 5p | TCTGCCTGTAGGAATGCTGTAGG |  |  |  |
| ssc-miR-novel-chr16_17203 | ssc-mir-novel-chr16_17203 | 3p | TGTGGGGCTGATGACACT | 8 |  | 1.8 |
| ssc-miR-novel-chr16_17338 | ssc-mir-novel-chr16_17338 | 5p | CGTGCGTCTCTGGGTGTGATGTCG |  |  |  |
| ssc-miR-novel-chr16_17347 | ssc-mir-novel-chr16_17347 | 3p | TGGAAGGACACAGGAAGCCT | 3 |  | 1.3 |
| ssc-miR-novel-chr16_17372 | ssc-mir-novel-chr16_17372 | 3p | GTCCTCGGCTGGGAGGAGGA |  |  |  |
| ssc-miR-novel-chr16_17391 | ssc-mir-novel-chr16_17391 | 5p | TGAGGTAGTAGGCTGTGTGG | 13 | 23 | 0.696969697 |
| ssc-miR-novel-chr16_17392 | ssc-mir-novel-chr16_17392 | 3p | TGAGGTAGTAGGCTGTGTGG | 202 | 137 | 1.442176871 |
| ssc-miR-novel-chr16_17559 | ssc-mir-novel-chr16_17559 | 5p | TGGCAGTGTATTGTTAGCTGGT | 119 |  | 12.9 |
| ssc-miR-novel-chr16_17561 | ssc-mir-novel-chr16_17561 | 5p | AGGCAGTGTATTGTTAGCTGGCT | 6 |  | 1.6 |
| ssc-miR-novel-chr16_17571 | ssc-mir-novel-chr16_17571 | 5p | TGGGTGGAAGAAGTGACC | 16 |  | 2.6 |
| ssc-miR-novel-chr16_17722 | ssc-mir-novel-chr16_17722 | 3p | TAGCCTGACGCTGATGATTGT |  |  |  |
| ssc-miR-novel-chr16_17741 | ssc-mir-novel-chr16_17741 | 5p | TAAGAAATAGGTCATTAACAGTA |  |  |  |
| ssc-miR-novel-chr16_17764 | ssc-mir-novel-chr16_17764 | 3p | TGTGGGGCTGATGACACT | 8 |  | 1.8 |
| ssc-miR-novel-chr16_17872 | ssc-mir-novel-chr16_17872 | 3p | AGGGGGAGGCAGGGGGGG |  |  |  |
| ssc-miR-novel-chr16_17883 | ssc-mir-novel-chr16_17883 | 5p | GCCCCCGGTGGCGGGGGGG |  | 1 | 0.909090909 |
| ssc-miR-novel-chr17_17993 | ssc-mir-novel-chr17_17993 | 3p | CGGGGCAGCTCAGTACAGGAC |  |  |  |
| ssc-miR-novel-chr17_17994 | ssc-mir-novel-chr17_17994 | 5p | CGGGGCAGCTCAGTACAGGAC |  |  |  |
| ssc-miR-novel-chr17_18002 | ssc-mir-novel-chr17_18002 | 5p | CGGTGGGGTGCAGTGCTGGACT |  | 2 | 0.833333333 |
| ssc-miR-novel-chr17_18117 | ssc-mir-novel-chr17_18117 | 5p | CTGGGCTGCCTCTGGGT |  | 1 | 0.909090909 |
| ssc-miR-novel-chr17_18122 | ssc-mir-novel-chr17_18122 | 3p | CAAGGCTCCACCTGCGCCCAAG |  |  |  |
| ssc-miR-novel-chr17_18126 | ssc-mir-novel-chr17_18126 | 5p | AGTGGTGAAATGTATTTAGGAC |  |  |  |
| ssc-miR-novel-chr17_18148 | ssc-mir-novel-chr17_18148 | 5p | TACAGTGGCTGTGGCTC | 9 | 2 | 1.583333333 |
| ssc-miR-novel-chr17_18173 | ssc-mir-novel-chr17_18173 | 3p | AATGATGCCCCTTAGAGTTGAGC |  |  |  |
| ssc-miR-novel-chr17_18175 | ssc-mir-novel-chr17_18175 | 3p | AATGATGCCCCTTAGAGTTGAGC |  |  |  |
| ssc-miR-novel-chr17_18195 | ssc-mir-novel-chr17_18195 | 5p | GGGTTAAGGATCTGGTGT | 6 |  | 1.6 |
| ssc-miR-novel-chr17_18444 | ssc-mir-novel-chr17_18444 | 5p | TATGGTCACTGCTGTGACACGGGT |  | 2 | 0.833333333 |
| ssc-miR-novel-chr17_18693 | ssc-mir-novel-chr17_18693 | 5p | AGTGGTGAAATGTATTTAGGAC |  |  |  |
| ssc-miR-novel-chr17_18954 | ssc-mir-novel-chr17_18954 | 3p | GGTTTTGGTCCTCGGCC | 50 | 8 | 3.333333333 |
| ssc-miR-novel-chr17_18970 | ssc-mir-novel-chr17_18970 | 3p | TGTGTCCTCAGTAACCT | 82 |  | 9.2 |
| ssc-miR-novel-chr17_18987 | ssc-mir-novel-chr17_18987 | 5p | AGGGTTGGGCGGAGGCTTT | 126 | 3 | 10.46153846 |
| ssc-miR-novel-chr18_19035 | ssc-mir-novel-chr18_19035 | 3p | GCGGGGGTGGCGGCGGG |  | 3 | 0.769230769 |
| ssc-miR-novel-chr18_19202 | ssc-mir-novel-chr18_19202 | 5p | TTGCCCCGTCGAAGCTGTCGGTG |  |  |  |
| ssc-miR-novel-chr18_19204 | ssc-mir-novel-chr18_19204 | 5p | TTGCCCCGTCGAAGCTGTCGGTG |  |  |  |
| ssc-miR-novel-chr18_19238 | ssc-mir-novel-chr18_19238 | 5p | TTTGGCACTAGCACATTTTTGCT | 13 | 116 | 0.182539683 |
| ssc-miR-novel-chr18_19266 | ssc-mir-novel-chr18_19266 | 5p | TTGTGTCAATATGCGATGATGT |  |  |  |
| ssc-miR-novel-chr18_19287 | ssc-mir-novel-chr18_19287 | 3p | TGGGGTGTGGTGGTGGTGGG |  |  |  |
| ssc-miR-novel-chr18_19390 | ssc-mir-novel-chr18_19390 | 5p | TGATGTCAGGATGAAGCTTGGACT |  |  |  |
| ssc-miR-novel-chr18_19420 | ssc-mir-novel-chr18_19420 | 5p | TTCTGGAAGATGTAGTCTGGA |  |  |  |
| ssc-miR-novel-chr18_19428 | ssc-mir-novel-chr18_19428 | 5p | TGAGCGGACAAGTTTGAGCCCCT |  |  |  |
| ssc-miR-novel-chr18_19466 | ssc-mir-novel-chr18_19466 | 5p | ACGAAGAACATTTGTGTAGCT |  |  |  |
| ssc-miR-novel-chr18_19490 | ssc-mir-novel-chr18_19490 | 3p | GTTCCCTCGTGGCTCAATGGGT |  |  |  |
| ssc-miR-novel-chr18_19647 | ssc-mir-novel-chr18_19647 | 5p | TGAGGGAGGAGTATGTGCTGTG |  |  |  |
| ssc-miR-novel-chr18_19712 | ssc-mir-novel-chr18_19712 | 3p | AACATGGAAGTGGATTGAGG |  |  |  |
| ssc-miR-novel-chr18_19745 | ssc-mir-novel-chr18_19745 | 5p | TTATGGCCCTTCGGTAATTCACT | 2 |  | 1.2 |
| ssc-miR-novel-chr18_19771 | ssc-mir-novel-chr18_19771 | 5p | TGAACAGAGGTCATGAACAGAGC | 4 | 1 | 1.272727273 |
| ssc-miR-novel-chr18_19783 | ssc-mir-novel-chr18_19783 | 5p | TTGTGTCAATATGCGATGATGT |  |  |  |
| ssc-miR-novel-chr18_19904 | ssc-mir-novel-chr18_19904 | 3p | TTCCCTTTGGGCCCTCAGG |  |  |  |
| ssc-miR-novel-chr18_20001 | ssc-mir-novel-chr18_20001 | 5p | CAAGGAGGAGTCAGATGAGGCTG | 2 |  | 1.2 |
| ssc-miR-novel-chr18_20013 | ssc-mir-novel-chr18_20013 | 5p | TACTGCAAAGTGATTGAGGAGC |  |  |  |
| ssc-miR-novel-chr18_20025 | ssc-mir-novel-chr18_20025 | 3p | GTTCCCTCGTGGCTCAATGGGT |  |  |  |
| ssc-miR-novel-chr1_1003 | ssc-mir-novel-chr1_1003 | 3p | TGGACTAACTGTGGTATTGGGA |  |  |  |
| ssc-miR-novel-chr1_1012 | ssc-mir-novel-chr1_1012 | 5p | TTCAGGTAAAAGACACACACT |  | 2 | 0.833333333 |
| ssc-miR-novel-chr1_1152 | ssc-mir-novel-chr1_1152 | 5p | AGGCCCTTACATAGTCAGACT |  |  |  |
| ssc-miR-novel-chr1_1175 | ssc-mir-novel-chr1_1175 | 3p | ACTGTGTGTGAGGAAGTC |  |  |  |
| ssc-miR-novel-chr1_1345 | ssc-mir-novel-chr1_1345 | 3p | TAACCCGACATTCAAGGCCTGT |  |  |  |
| ssc-miR-novel-chr1_1497 | ssc-mir-novel-chr1_1497 | 3p | TACCACAGGGCAGAACCACGT |  |  |  |
| ssc-miR-novel-chr1_1514 | ssc-mir-novel-chr1_1514 | 5p | TCTGATTTGGATTCCAGA |  |  |  |
| ssc-miR-novel-chr1_1558 | ssc-mir-novel-chr1_1558 | 5p | CGGGGCGGGCGGTCGCCCGGGGC |  | 80 | 0.111111111 |
| ssc-miR-novel-chr1_1613 | ssc-mir-novel-chr1_1613 | 3p | ATAAAACTTAGTCGCTGCT |  |  |  |
| ssc-miR-novel-chr1_1798 | ssc-mir-novel-chr1_1798 | 5p | TGGAAACACTTCTGCACAAACT | 5 | 1 | 1.363636364 |
| ssc-miR-novel-chr1_1972 | ssc-mir-novel-chr1_1972 | 5p | TTCCCTTTGTCATCCTTTGCCC |  |  |  |
| ssc-miR-novel-chr1_2074 | ssc-mir-novel-chr1_2074 | 5p | TTTAGTGTGATAATGGCGTTTG |  |  |  |
| ssc-miR-novel-chr1_2132 | ssc-mir-novel-chr1_2132 | 5p | TCGTTAGTTCTCTGAGGCTCCGA | 3 |  | 1.3 |
| ssc-miR-novel-chr1_2249 | ssc-mir-novel-chr1_2249 | 3p | AGACCGGGGACCGGAGCGAGCAGA |  | 1 | 0.909090909 |
| ssc-miR-novel-chr1_2311 | ssc-mir-novel-chr1_2311 | 5p | ATTTGGTGGATTGTGGCAGTA |  |  |  |
| ssc-miR-novel-chr1_2360 | ssc-mir-novel-chr1_2360 | 5p | CGGCAGACTGGGACTTGTTGTT |  |  |  |
| ssc-miR-novel-chr1_2748 | ssc-mir-novel-chr1_2748 | 5p | AAAGGAGAGGAAAGAAGG |  |  |  |
| ssc-miR-novel-chr1_2775 | ssc-mir-novel-chr1_2775 | 3p | ACTAGAAATTGGGTAGGACAGA |  |  |  |
| ssc-miR-novel-chr1_2959 | ssc-mir-novel-chr1_2959 | 3p | TGGGGTGGGGCTCTCAACCTCCAGA |  |  |  |
| ssc-miR-novel-chr1_2989 | ssc-mir-novel-chr1_2989 | 3p | GCGCGTCTTTTGGTGCTCTGCAG | 2 |  | 1.2 |
| ssc-miR-novel-chr1_3279 | ssc-mir-novel-chr1_3279 | 5p | CTCTAGAGGTGGATTTGCTGTGCTG |  |  |  |
| ssc-miR-novel-chr1_3280 | ssc-mir-novel-chr1_3280 | 3p | CTCTAGAGGTGGATTTGCTGTGCTG |  |  |  |
| ssc-miR-novel-chr1_3515 | ssc-mir-novel-chr1_3515 | 5p | AAACATTAGAATGTCTGAGTGG |  | 2 | 0.833333333 |
| ssc-miR-novel-chr1_3810 | ssc-mir-novel-chr1_3810 | 3p | ATAAAACTTAGTCGCTGCT |  |  |  |
| ssc-miR-novel-chr1_3836 | ssc-mir-novel-chr1_3836 | 3p | CGCCGGGGGGGCGGGGG |  | 1 | 0.909090909 |
| ssc-miR-novel-chr1_3896 | ssc-mir-novel-chr1_3896 | 3p | CAATTTAGGTTAATGACTGTC |  | 1 | 0.909090909 |
| ssc-miR-novel-chr1_3924 | ssc-mir-novel-chr1_3924 | 3p | TTGCCTGGGACTCTGGAACC |  | 1 | 0.909090909 |
| ssc-miR-novel-chr1_4044 | ssc-mir-novel-chr1_4044 | 3p | ACTGGTTGGATTGGAGACTAGG | 3 |  | 1.3 |
| ssc-miR-novel-chr1_4317 | ssc-mir-novel-chr1_4317 | 5p | CTGCCGTTGGGTCTGGGGTGT |  | 3 | 0.769230769 |
| ssc-miR-novel-chr1_4339 | ssc-mir-novel-chr1_4339 | 5p | TCGTTAGTTCTCTGAGGCTCCGA | 3 |  | 1.3 |
| ssc-miR-novel-chr1_4468 | ssc-mir-novel-chr1_4468 | 3p | AACTGTTCTACCTCTGAGTAGC |  |  |  |
| ssc-miR-novel-chr1_4539 | ssc-mir-novel-chr1_4539 | 5p | AAGGTTACTTGTTAGTTCAGGA |  |  |  |
| ssc-miR-novel-chr1_4584 | ssc-mir-novel-chr1_4584 | 3p | TATGGAGGGTAGACTGTACT |  | 2 | 0.833333333 |
| ssc-miR-novel-chr1_4647 | ssc-mir-novel-chr1_4647 | 5p | CAGAGCTGTTTGTGAACTGCAGGT | 7 | 14 | 0.708333333 |
| ssc-miR-novel-chr1_4720 | ssc-mir-novel-chr1_4720 | 3p | CTGGAGGAAGAGCTCCCTG |  | 1 | 0.909090909 |
| ssc-miR-novel-chr1_4762 | ssc-mir-novel-chr1_4762 | 3p | AGAGTCGGCTGCGCGGAGCC |  |  |  |
| ssc-miR-novel-chr1_4788 | ssc-mir-novel-chr1_4788 | 3p | TAGCTAAGTGCAAATATGTCCAT |  |  |  |
| ssc-miR-novel-chr1_4860 | ssc-mir-novel-chr1_4860 | 3p | ACTAGAAATTGGGTAGGACAGA |  |  |  |
| ssc-miR-novel-chr1_4929 | ssc-mir-novel-chr1_4929 | 3p | AGGCTGGGGGTGGGGCC |  |  |  |
| ssc-miR-novel-chr1_5015 | ssc-mir-novel-chr1_5015 | 5p | ACAGTCAACGGTCGGTGGTTT | 4 | 8 | 0.777777778 |
| ssc-miR-novel-chr1_5092 | ssc-mir-novel-chr1_5092 | 3p | CAGAAGGGGAGTCGGAGCAGA |  |  |  |
| ssc-miR-novel-chr1_5095 | ssc-mir-novel-chr1_5095 | 5p | TCTGGAGGACGCTAGAGCTGGGC |  |  |  |
| ssc-miR-novel-chr1_5102 | ssc-mir-novel-chr1_5102 | 3p | AGAATTGTGGCTGGACATCTGT |  | 2 | 0.833333333 |
| ssc-miR-novel-chr1_5156 | ssc-mir-novel-chr1_5156 | 3p | TGCAGTTGCCGGGAAGCGGGC |  |  |  |
| ssc-miR-novel-chr1_5195 | ssc-mir-novel-chr1_5195 | 5p | CATTATTACTCACGGTACGAGT |  |  |  |
| ssc-miR-novel-chr1_5196 | ssc-mir-novel-chr1_5196 | 3p | CATTATTACTCACGGTACGAGT |  |  |  |
| ssc-miR-novel-chr1_5208 | ssc-mir-novel-chr1_5208 | 3p | AAGGGTGGGTGGCCACTGTGC |  |  |  |
| ssc-miR-novel-chr1_946 | ssc-mir-novel-chr1_946 | 5p | TCCCTGTGGTCTAGGGGTTAGGA | 2 | 57 | 0.179104478 |
| ssc-miR-novel-chr2_20089 | ssc-mir-novel-chr2_20089 | 5p | CTGGGCGGACGTGGGGT |  |  |  |
| ssc-miR-novel-chr2_20131 | ssc-mir-novel-chr2_20131 | 3p | CCTCTCTTCCCTCACGGTCGCT | 4 |  | 1.4 |
| ssc-miR-novel-chr2_20138 | ssc-mir-novel-chr2_20138 | 5p | GACCTGTGATGTCGTTTCTGAGA |  | 1 | 0.909090909 |
| ssc-miR-novel-chr2_20190 | ssc-mir-novel-chr2_20190 | 5p | CTCGGGCCTGCGCGCGCGAGGA |  | 1 | 0.909090909 |
| ssc-miR-novel-chr2_20212 | ssc-mir-novel-chr2_20212 | 5p | GACTGGAAGTCTCAGGGGGTGT |  |  |  |
| ssc-miR-novel-chr2_20240 | ssc-mir-novel-chr2_20240 | 5p | ATGTGTGGGTCGGCACCGA |  |  |  |
| ssc-miR-novel-chr2_20261 | ssc-mir-novel-chr2_20261 | 3p | GGGCAAGCCTGCGGAGGTGTGG |  | 3 | 0.769230769 |
| ssc-miR-novel-chr2_20299 | ssc-mir-novel-chr2_20299 | 3p | CATTCTCTGGTGGCCTAGCAGG | 2 | 2 | 1 |
| ssc-miR-novel-chr2_20300 | ssc-mir-novel-chr2_20300 | 5p | CATTCTCTGGTGGCCTAGCAGG |  | 1 | 0.909090909 |
| ssc-miR-novel-chr2_20315 | ssc-mir-novel-chr2_20315 | 5p | CCACCCTCATCGCTGCCTGTGTGT |  |  |  |
| ssc-miR-novel-chr2_20345 | ssc-mir-novel-chr2_20345 | 3p | CTTTGTGGTCCTGTTCTT |  |  |  |
| ssc-miR-novel-chr2_20448 | ssc-mir-novel-chr2_20448 | 5p | GTGGGGAAGAACTACAAGACAGCT | 114 | 3 | 9.538461538 |
| ssc-miR-novel-chr2_20504 | ssc-mir-novel-chr2_20504 | 5p | AGTCTCAGTGTGGTCTTCAGAGA |  |  |  |
| ssc-miR-novel-chr2_20597 | ssc-mir-novel-chr2_20597 | 5p | TCGTTGGACACAGATCACTACACT | 4 | 3 | 1.076923077 |
| ssc-miR-novel-chr2_20662 | ssc-mir-novel-chr2_20662 | 5p | CAGTTCACAGTTCATAGGAGGATG |  |  |  |
| ssc-miR-novel-chr2_20682 | ssc-mir-novel-chr2_20682 | 5p | ATTCGGGTGCAGGGACTGAGAGC |  |  |  |
| ssc-miR-novel-chr2_20831 | ssc-mir-novel-chr2_20831 | 3p | CCTGGGGGTCTGAGGGGC | 2 |  | 1.2 |
| ssc-miR-novel-chr2_20848 | ssc-mir-novel-chr2_20848 | 5p | TAACCAATGTGCAGACTACTGT |  | 2 | 0.833333333 |
| ssc-miR-novel-chr2_20962 | ssc-mir-novel-chr2_20962 | 5p | TCCTGTGATGATCTCCCTGAGC |  |  |  |
| ssc-miR-novel-chr2_20965 | ssc-mir-novel-chr2_20965 | 3p | TGAGATGAGAGCCCTTGGCCACT | 24 | 1 | 3.090909091 |
| ssc-miR-novel-chr2_21078 | ssc-mir-novel-chr2_21078 | 5p | TAGTTGCTGTGGGAAGTGAGC |  |  |  |
| ssc-miR-novel-chr2_21186 | ssc-mir-novel-chr2_21186 | 5p | TAAAATGCTATTCTGTCACTGT |  | 1 | 0.909090909 |
| ssc-miR-novel-chr2_21261 | ssc-mir-novel-chr2_21261 | 3p | TGACAATGATGTATCCACTGAGC |  | 1 | 0.909090909 |
| ssc-miR-novel-chr2_21343 | ssc-mir-novel-chr2_21343 | 3p | ACTGAAGTGATGGTCTTGGGAC |  |  |  |
| ssc-miR-novel-chr2_21352 | ssc-mir-novel-chr2_21352 | 5p | ATGTTGGACTCAGAACTCTCACTC |  | 19 | 0.344827586 |
| ssc-miR-novel-chr2_21472 | ssc-mir-novel-chr2_21472 | 5p | CGCGTGCGCGTCGGGTC | 153 | 30 | 4.075 |
| ssc-miR-novel-chr2_21617 | ssc-mir-novel-chr2_21617 | 3p | TGGATTTTTGGAGCTGG | 4 | 362 | 0.037634409 |
| ssc-miR-novel-chr2_21624 | ssc-mir-novel-chr2_21624 | 5p | TGAGATGAAGCACTGTAGCT | 63 | 92 | 0.715686275 |
| ssc-miR-novel-chr2_21667 | ssc-mir-novel-chr2_21667 | 3p | TTCACTGGTGCCCATGGAGGA |  |  |  |
| ssc-miR-novel-chr2_21682 | ssc-mir-novel-chr2_21682 | 3p | AGCAGCGGCGGCGATGGCAGGCT |  |  |  |
| ssc-miR-novel-chr2_21748 | ssc-mir-novel-chr2_21748 | 3p | CCTCTCTTCCCTCACGGTCGCT | 4 |  | 1.4 |
| ssc-miR-novel-chr2_21765 | ssc-mir-novel-chr2_21765 | 5p | AGCACTTACGGATTCTGGGGG | 2 | 2 | 1 |
| ssc-miR-novel-chr2_21774 | ssc-mir-novel-chr2_21774 | 3p | TTATCCGGGCTTGTGCTGAAGC | 13 | 1 | 2.090909091 |
| ssc-miR-novel-chr2_21790 | ssc-mir-novel-chr2_21790 | 3p | AGCACTGATGGAGCCTGAGC |  |  |  |
| ssc-miR-novel-chr2_21820 | ssc-mir-novel-chr2_21820 | 3p | TTTGTTGGCTCCTCTGAAGTGA | 836 | 160 | 4.976470588 |
| ssc-miR-novel-chr2_21982 | ssc-mir-novel-chr2_21982 | 3p | TTTCCTCATATTCATTCAGGAGT | 3 |  | 1.3 |
| ssc-miR-novel-chr2_22148 | ssc-mir-novel-chr2_22148 | 3p | TAGCTCCCTGTGCCAGTCCTGA |  | 1 | 0.909090909 |
| ssc-miR-novel-chr2_22149 | ssc-mir-novel-chr2_22149 | 5p | ATGGTTGGGAGGATTCTCAGA |  | 1 | 0.909090909 |
| ssc-miR-novel-chr2_22156 | ssc-mir-novel-chr2_22156 | 3p | TGTGTCTGTGACATCTGCTGGCA | 17 | 3 | 2.076923077 |
| ssc-miR-novel-chr2_22194 | ssc-mir-novel-chr2_22194 | 3p | TAGCTCTGCCTTGTCTCCCCAGC |  |  |  |
| ssc-miR-novel-chr2_22201 | ssc-mir-novel-chr2_22201 | 5p | TACTGTGGTGTCCTGTGTCTCTG |  |  |  |
| ssc-miR-novel-chr2_22202 | ssc-mir-novel-chr2_22202 | 3p | TACTGTGGTGTCCTGTGTCTCTG |  | 1 | 0.909090909 |
| ssc-miR-novel-chr2_22309 | ssc-mir-novel-chr2_22309 | 5p | AGGATACGTCGCAGACGTGGTT |  | 15 | 0.4 |
| ssc-miR-novel-chr2_22316 | ssc-mir-novel-chr2_22316 | 3p | CTGCCTGGACCCCTGCTCACCGC | 2 |  | 1.2 |
| ssc-miR-novel-chr2_22420 | ssc-mir-novel-chr2_22420 | 3p | AGCCTCTCTGCTCCTTCCCAGG |  |  |  |
| ssc-miR-novel-chr2_22433 | ssc-mir-novel-chr2_22433 | 5p | TCAGGGAGGGAACACAGGGAGC |  |  |  |
| ssc-miR-novel-chr2_22438 | ssc-mir-novel-chr2_22438 | 3p | CATGGCCCTCCACCCCTGCAGG |  |  |  |
| ssc-miR-novel-chr2_22453 | ssc-mir-novel-chr2_22453 | 5p | GTCTCGGGCTCTTGCGACCCA |  |  |  |
| ssc-miR-novel-chr2_22508 | ssc-mir-novel-chr2_22508 | 3p | GAGTCAGAGCTGCAGGTTGTGG |  | 1 | 0.909090909 |
| ssc-miR-novel-chr2_22514 | ssc-mir-novel-chr2_22514 | 3p | TCTGTGCTCTGAGAGGGGC | 2 |  | 1.2 |
| ssc-miR-novel-chr2_22525 | ssc-mir-novel-chr2_22525 | 5p | CGTGGGTGTGTGATGGCT |  |  |  |
| ssc-miR-novel-chr2_22565 | ssc-mir-novel-chr2_22565 | 5p | ACATTTAAGGAGGTGCTTGCT | 4 | 1 | 1.272727273 |
| ssc-miR-novel-chr2_22609 | ssc-mir-novel-chr2_22609 | 5p | GGAAAAAAGAAATGGAGATGG | 4 |  | 1.4 |
| ssc-miR-novel-chr2_22652 | ssc-mir-novel-chr2_22652 | 3p | AGGCTCTGATTGGATTGGGTG |  |  |  |
| ssc-miR-novel-chr2_22879 | ssc-mir-novel-chr2_22879 | 5p | CGCGTGCGCGTCGGGTC | 153 | 30 | 4.075 |
| ssc-miR-novel-chr2_23068 | ssc-mir-novel-chr2_23068 | 5p | TTCCGGTCATTGTCTGTACGTGG |  |  |  |
| ssc-miR-novel-chr2_23096 | ssc-mir-novel-chr2_23096 | 3p | TTCACTGGTGCCCATGGAGGA |  |  |  |
| ssc-miR-novel-chr3_23111 | ssc-mir-novel-chr3_23111 | 3p | GCAGAGCAGCGGCAGGAGG |  |  |  |
| ssc-miR-novel-chr3_23137 | ssc-mir-novel-chr3_23137 | 3p | CTCTGCCACACCACCCGCCTGC | 2 |  | 1.2 |
| ssc-miR-novel-chr3_23139 | ssc-mir-novel-chr3_23139 | 3p | CTGCGGTGCTGTGTGCTTTGGT |  | 3 | 0.769230769 |
| ssc-miR-novel-chr3_23263 | ssc-mir-novel-chr3_23263 | 3p | AGCTGGTCTGGGAGTTCCCGGGT |  | 3 | 0.769230769 |
| ssc-miR-novel-chr3_23271 | ssc-mir-novel-chr3_23271 | 3p | TAATTTTATGTATAAGCTAGT | 102 | 17 | 4.148148148 |
| ssc-miR-novel-chr3_23293 | ssc-mir-novel-chr3_23293 | 3p | AGTGGGAGCTGTGTTGACTG |  |  |  |
| ssc-miR-novel-chr3_23295 | ssc-mir-novel-chr3_23295 | 3p | AGTGGGAGCTGTGTTGACTG |  |  |  |
| ssc-miR-novel-chr3_23361 | ssc-mir-novel-chr3_23361 | 3p | AGGAAACTCTGGGGGTGG | 3 | 24 | 0.382352941 |
| ssc-miR-novel-chr3_23365 | ssc-mir-novel-chr3_23365 | 3p | CTCCCTGCTCTTCGGCTCAGAGT |  |  |  |
| ssc-miR-novel-chr3_23411 | ssc-mir-novel-chr3_23411 | 3p | ATCTGTCTGTGTCTCTGAGCAG | 3 |  | 1.3 |
| ssc-miR-novel-chr3_23413 | ssc-mir-novel-chr3_23413 | 3p | GTCAGGATGGCCGAGTGGTCTAAGG |  | 33 | 0.23255814 |
| ssc-miR-novel-chr3_23465 | ssc-mir-novel-chr3_23465 | 3p | CAGGGTCGGGCCTGGTTA | 137 |  | 14.7 |
| ssc-miR-novel-chr3_23507 | ssc-mir-novel-chr3_23507 | 3p | TCTGAGACGGACATCTGGTCC | 2 |  | 1.2 |
| ssc-miR-novel-chr3_23547 | ssc-mir-novel-chr3_23547 | 3p | CTGGGCTGTGCCGGGTGC |  | 1 | 0.909090909 |
| ssc-miR-novel-chr3_23548 | ssc-mir-novel-chr3_23548 | 5p | CTGGGCTGTGCCGGGTGC |  | 1 | 0.909090909 |
| ssc-miR-novel-chr3_23558 | ssc-mir-novel-chr3_23558 | 5p | CGGGGCTGGGCGCGCGC | 6 | 2 | 1.333333333 |
| ssc-miR-novel-chr3_23681 | ssc-mir-novel-chr3_23681 | 3p | CAGACCAGCAGGATGTGGACT | 4 |  | 1.4 |
| ssc-miR-novel-chr3_23694 | ssc-mir-novel-chr3_23694 | 5p | GGGCATCCCTGTAGGAGCT |  |  |  |
| ssc-miR-novel-chr3_23708 | ssc-mir-novel-chr3_23708 | 5p | AACACATGTGTGCTGAGTGGAACT | 2 | 1 | 1.090909091 |
| ssc-miR-novel-chr3_23955 | ssc-mir-novel-chr3_23955 | 3p | TGTTGTTCTGAAGGTGA |  |  |  |
| ssc-miR-novel-chr3_24036 | ssc-mir-novel-chr3_24036 | 5p | AGGCTGAGGCTGGAGGA |  | 4 | 0.714285714 |
| ssc-miR-novel-chr3_24093 | ssc-mir-novel-chr3_24093 | 3p | GCACCTTTATTCTTCTAACTG |  |  |  |
| ssc-miR-novel-chr3_24096 | ssc-mir-novel-chr3_24096 | 5p | CTCCCGTGCTGATCAGTAGTGGG |  |  |  |
| ssc-miR-novel-chr3_24244 | ssc-mir-novel-chr3_24244 | 5p | TTCCCTTTGTAATCCTATGCAT |  |  |  |
| ssc-miR-novel-chr3_24308 | ssc-mir-novel-chr3_24308 | 5p | CCAGTGATCAGGTTACGATGGATT |  | 2 | 0.833333333 |
| ssc-miR-novel-chr3_24388 | ssc-mir-novel-chr3_24388 | 3p | GCAGAGCAGCGGCAGGAGG |  |  |  |
| ssc-miR-novel-chr3_24518 | ssc-mir-novel-chr3_24518 | 5p | CCGGGAACTCCCAGACCAGCTTC |  |  |  |
| ssc-miR-novel-chr3_24548 | ssc-mir-novel-chr3_24548 | 3p | ACATTCAGAGAACTGTAAACGCC | 4 |  | 1.4 |
| ssc-miR-novel-chr3_24599 | ssc-mir-novel-chr3_24599 | 5p | GTAGACCCGGGACTTCGCTG |  |  |  |
| ssc-miR-novel-chr3_24618 | ssc-mir-novel-chr3_24618 | 3p | CTGCCCACCTGGGTGAGAAGGA |  | 4 | 0.714285714 |
| ssc-miR-novel-chr3_24739 | ssc-mir-novel-chr3_24739 | 5p | TGCAGGACCTCAGAGCAGCT |  |  |  |
| ssc-miR-novel-chr3_24773 | ssc-mir-novel-chr3_24773 | 5p | AGGAGTGGTTGCTGGCAGTGTGC |  |  |  |
| ssc-miR-novel-chr3_24811 | ssc-mir-novel-chr3_24811 | 5p | TGGCTCTGCGAGGTCGGCTCA | 114 | 108 | 1.050847458 |
| ssc-miR-novel-chr3_24812 | ssc-mir-novel-chr3_24812 | 3p | TGGCTCTGCGAGGTCGGCTCA | 114 | 108 | 1.050847458 |
| ssc-miR-novel-chr3_24814 | ssc-mir-novel-chr3_24814 | 3p | CGAGCGGGCCCGGACGCTGCTGGC |  |  |  |
| ssc-miR-novel-chr3_24923 | ssc-mir-novel-chr3_24923 | 5p | GGGCATCCCTGTAGGAGCT |  |  |  |
| ssc-miR-novel-chr3_24929 | ssc-mir-novel-chr3_24929 | 5p | ACACTCAGCATGCACATGGAGGCT |  |  |  |
| ssc-miR-novel-chr3_24974 | ssc-mir-novel-chr3_24974 | 3p | CTGGGAGCAGTTGCCGGATGTG |  | 1 | 0.909090909 |
| ssc-miR-novel-chr3_25028 | ssc-mir-novel-chr3_25028 | 3p | TGTGGCTGTGGCCTGCAACT | 38 | 11 | 2.285714286 |
| ssc-miR-novel-chr3_25115 | ssc-mir-novel-chr3_25115 | 5p | TGGTTGGAGGAGCTGAGAGCGC |  |  |  |
| ssc-miR-novel-chr3_25160 | ssc-mir-novel-chr3_25160 | 3p | TAGTGCAGGTTGGATCCCTAGC |  | 1 | 0.909090909 |
| ssc-miR-novel-chr3_25222 | ssc-mir-novel-chr3_25222 | 5p | GCGTCACCAGCCGAGCCGG |  |  |  |
| ssc-miR-novel-chr3_25240 | ssc-mir-novel-chr3_25240 | 3p | TTCACAGGGGCTCAGTTC |  |  |  |
| ssc-miR-novel-chr3_25266 | ssc-mir-novel-chr3_25266 | 3p | AAGAGGGGCTTGGAGCTGGGCCT |  |  |  |
| ssc-miR-novel-chr3_25267 | ssc-mir-novel-chr3_25267 | 5p | AGATTCAGTCAACCGCGGATC | 2 |  | 1.2 |
| ssc-miR-novel-chr3_25338 | ssc-mir-novel-chr3_25338 | 3p | GATATGAGAGTGTTGGTCCTGA | 4 |  | 1.4 |
| ssc-miR-novel-chr3_25350 | ssc-mir-novel-chr3_25350 | 3p | AGTGGCTGTGGCTCGACCCCT | 9 |  | 1.9 |
| ssc-miR-novel-chr3_25488 | ssc-mir-novel-chr3_25488 | 3p | CCCGGGGCTGCAGAAAGA |  |  |  |
| ssc-miR-novel-chr3_25504 | ssc-mir-novel-chr3_25504 | 3p | TTTCGGACCACAGCCTGGGCATC | 3 |  | 1.3 |
| ssc-miR-novel-chr3_25572 | ssc-mir-novel-chr3_25572 | 3p | CTGTCCTGGGCCTGTCAGAGTCTCC |  |  |  |
| ssc-miR-novel-chr4_25596 | ssc-mir-novel-chr4_25596 | 5p | GTGAAGGGTGCAGGTTCGGG |  |  |  |
| ssc-miR-novel-chr4_25600 | ssc-mir-novel-chr4_25600 | 5p | GCGCCGCGCTGGGAGCCCTGCGGA |  |  |  |
| ssc-miR-novel-chr4_25612 | ssc-mir-novel-chr4_25612 | 5p | CCCACGGTCCACCACTTTGCTGT | 2 | 2 | 1 |
| ssc-miR-novel-chr4_25615 | ssc-mir-novel-chr4_25615 | 3p | CGTTGTGATGAAGTGCTGAGG |  | 2 | 0.833333333 |
| ssc-miR-novel-chr4_25671 | ssc-mir-novel-chr4_25671 | 3p | GGGTCGGGGCGGGGCGG |  | 1 | 0.909090909 |
| ssc-miR-novel-chr4_25720 | ssc-mir-novel-chr4_25720 | 5p | AGGAGAAAGCAGACGGGCTGCA | 12 | 7 | 1.294117647 |
| ssc-miR-novel-chr4_25914 | ssc-mir-novel-chr4_25914 | 5p | TATACCTCAGTTTTATCAGGTG |  |  |  |
| ssc-miR-novel-chr4_25916 | ssc-mir-novel-chr4_25916 | 5p | TTTGATAAGCTGACATGGGACA |  |  |  |
| ssc-miR-novel-chr4_26000 | ssc-mir-novel-chr4_26000 | 5p | TTCCTGAGTCGGACTGGGCTGC |  |  |  |
| ssc-miR-novel-chr4_26125 | ssc-mir-novel-chr4_26125 | 3p | ATGGATTGTCTTCTGGGTACT |  |  |  |
| ssc-miR-novel-chr4_26129 | ssc-mir-novel-chr4_26129 | 3p | TCAAGGTCCGCTGTGAACACGG | 4 |  | 1.4 |
| ssc-miR-novel-chr4_26236 | ssc-mir-novel-chr4_26236 | 5p | CTAGAGTGGATTCTGCA |  |  |  |
| ssc-miR-novel-chr4_26332 | ssc-mir-novel-chr4_26332 | 5p | GTCACCTGGCAGGTACCTCTTT |  |  |  |
| ssc-miR-novel-chr4_26365 | ssc-mir-novel-chr4_26365 | 3p | TCATGAGAAAGTGCCTGGAAC | 5 |  | 1.5 |
| ssc-miR-novel-chr4_26369 | ssc-mir-novel-chr4_26369 | 3p | CGGACAGCTCGAAAGGGG |  |  |  |
| ssc-miR-novel-chr4_26379 | ssc-mir-novel-chr4_26379 | 3p | TCGGCGCCCCACCCTCTCTAGC | 3 |  | 1.3 |
| ssc-miR-novel-chr4_26443 | ssc-mir-novel-chr4_26443 | 3p | ACAGGACCCTACCCATCAAGA |  | 2 | 0.833333333 |
| ssc-miR-novel-chr4_26606 | ssc-mir-novel-chr4_26606 | 5p | TGGGGTTCAAAAGGACTCAAGA |  | 1 | 0.909090909 |
| ssc-miR-novel-chr4_26620 | ssc-mir-novel-chr4_26620 | 5p | TGGGGTTCAAAAGGACTCAAGA |  | 1 | 0.909090909 |
| ssc-miR-novel-chr4_26624 | ssc-mir-novel-chr4_26624 | 5p | TGGGGTTCAAAAGGACTCAAGA |  | 1 | 0.909090909 |
| ssc-miR-novel-chr4_26691 | ssc-mir-novel-chr4_26691 | 3p | GCCAGCCGGTGTTCATGCCCCATA |  |  |  |
| ssc-miR-novel-chr4_26743 | ssc-mir-novel-chr4_26743 | 3p | TGAGAGGAAGCACTGTAGGA |  |  |  |
| ssc-miR-novel-chr4_26821 | ssc-mir-novel-chr4_26821 | 3p | TCAGAAGTGGGTCCAGGAATCT | 7 |  | 1.7 |
| ssc-miR-novel-chr4_26845 | ssc-mir-novel-chr4_26845 | 3p | TCTTCCCAGGCTCTGTCTGAAC |  |  |  |
| ssc-miR-novel-chr4_26898 | ssc-mir-novel-chr4_26898 | 5p | AGTCCACTCGTCTCACTGAGA |  |  |  |
| ssc-miR-novel-chr4_26969 | ssc-mir-novel-chr4_26969 | 5p | CGGGGTGTGGGGAGGGC |  | 58 | 0.147058824 |
| ssc-miR-novel-chr4_27089 | ssc-mir-novel-chr4_27089 | 5p | CCTCTGGGCCACAGGAGAACT |  | 6 | 0.625 |
| ssc-miR-novel-chr4_27349 | ssc-mir-novel-chr4_27349 | 5p | TCACCTGTTGGATATTCT | 11 |  | 2.1 |
| ssc-miR-novel-chr4_27402 | ssc-mir-novel-chr4_27402 | 3p | ATGGATTGTCTTCTGGGTACT |  |  |  |
| ssc-miR-novel-chr4_27454 | ssc-mir-novel-chr4_27454 | 3p | CAGGACCAGGTGAGCGCCAGC |  |  |  |
| ssc-miR-novel-chr4_27574 | ssc-mir-novel-chr4_27574 | 3p | TCCCAAGACTCCTTTGATCCCT |  |  |  |
| ssc-miR-novel-chr4_27701 | ssc-mir-novel-chr4_27701 | 3p | TGTTGTCCTCCGAATCTGAAT |  |  |  |
| ssc-miR-novel-chr4_27704 | ssc-mir-novel-chr4_27704 | 3p | TCTCTGACTCTCTGACCTCCCAGG |  |  |  |
| ssc-miR-novel-chr4_27733 | ssc-mir-novel-chr4_27733 | 5p | CTGTGTGGTGTAGGGAGAAGCT |  |  |  |
| ssc-miR-novel-chr4_27870 | ssc-mir-novel-chr4_27870 | 5p | CACTCTGGACTCTGAATC |  | 11 | 0.476190476 |
| ssc-miR-novel-chr4_27873 | ssc-mir-novel-chr4_27873 | 5p | TGGGGTTCCGAAGGACTAAGA |  |  |  |
| ssc-miR-novel-chr4_27885 | ssc-mir-novel-chr4_27885 | 5p | TGGGGTTCCGAAGGACTAAGA |  |  |  |
| ssc-miR-novel-chr4_27887 | ssc-mir-novel-chr4_27887 | 5p | TGGGGTTCCGAAGGACTAAGA |  |  |  |
| ssc-miR-novel-chr4_27893 | ssc-mir-novel-chr4_27893 | 5p | TGAGAACTGAATTCGATGGGA |  | 7 | 0.588235294 |
| ssc-miR-novel-chr4_27928 | ssc-mir-novel-chr4_27928 | 3p | TGAGACACAGGGCGGGCTGTGCT |  | 1 | 0.909090909 |
| ssc-miR-novel-chr4_27930 | ssc-mir-novel-chr4_27930 | 3p | TATATATATATGATTCACGATG |  |  |  |
| ssc-miR-novel-chr4_27970 | ssc-mir-novel-chr4_27970 | 3p | CAGTCGGGACGCTTCCTTCTT |  |  |  |
| ssc-miR-novel-chr4_28073 | ssc-mir-novel-chr4_28073 | 5p | ACGGAGGCCTCTTACCAGCA |  |  |  |
| ssc-miR-novel-chr4_28122 | ssc-mir-novel-chr4_28122 | 3p | CGGCTCTGGGTCTGTGGGGAGC | 76 | 19 | 2.965517241 |
| ssc-miR-novel-chr5_28279 | ssc-mir-novel-chr5_28279 | 3p | ATGCGGAACCTGCGGATACGG | 126 | 177 | 0.727272727 |
| ssc-miR-novel-chr5_28309 | ssc-mir-novel-chr5_28309 | 3p | TCAGTGGGCACCCAGCCGGAGT | 2 |  | 1.2 |
| ssc-miR-novel-chr5_28311 | ssc-mir-novel-chr5_28311 | 3p | TCAGTGGGCACCCAGCCGGAGT | 2 |  | 1.2 |
| ssc-miR-novel-chr5_28315 | ssc-mir-novel-chr5_28315 | 3p | CTCAGACCTCAGGCTTGGAGCC | 3 |  | 1.3 |
| ssc-miR-novel-chr5_28386 | ssc-mir-novel-chr5_28386 | 5p | TGGGACTTATAGGTGAACCAGA |  |  |  |
| ssc-miR-novel-chr5_28455 | ssc-mir-novel-chr5_28455 | 3p | TGGGAAGTGTCCAGGTTGGGA |  |  |  |
| ssc-miR-novel-chr5_28480 | ssc-mir-novel-chr5_28480 | 5p | TTAGGAGAGGAGCTGAGAAAGGG | 2 |  | 1.2 |
| ssc-miR-novel-chr5_28483 | ssc-mir-novel-chr5_28483 | 3p | TGATGTTTATCCGAATCCTCAGA |  | 1 | 0.909090909 |
| ssc-miR-novel-chr5_28488 | ssc-mir-novel-chr5_28488 | 5p | TTAGGAGAGGAGCTGAGAAAGGG | 2 |  | 1.2 |
| ssc-miR-novel-chr5_28715 | ssc-mir-novel-chr5_28715 | 3p | TGGTGGCAGAGCCAGGTAA |  |  |  |
| ssc-miR-novel-chr5_28736 | ssc-mir-novel-chr5_28736 | 5p | AAGGATGGGAAGGAGGGG |  |  |  |
| ssc-miR-novel-chr5_28811 | ssc-mir-novel-chr5_28811 | 3p | CAACACTGTGCTGGAAGATGGA |  | 5 | 0.666666667 |
| ssc-miR-novel-chr5_28846 | ssc-mir-novel-chr5_28846 | 5p | TGGGACTGAAGGCCGCGGCCTCC |  |  |  |
| ssc-miR-novel-chr5_28892 | ssc-mir-novel-chr5_28892 | 5p | CGCAGGGCTCGGGTCGGCTGCCT | 13 |  | 2.3 |
| ssc-miR-novel-chr5_29091 | ssc-mir-novel-chr5_29091 | 3p | TGCAAGGTCGGACCGCTGTGACC |  |  |  |
| ssc-miR-novel-chr5_29097 | ssc-mir-novel-chr5_29097 | 3p | TGCAAGGTCGGACCGCTGTGACC |  |  |  |
| ssc-miR-novel-chr5_29128 | ssc-mir-novel-chr5_29128 | 3p | TGGCTCTGCGAGGTCGGCT | 82 | 27 | 2.486486486 |
| ssc-miR-novel-chr5_29297 | ssc-mir-novel-chr5_29297 | 5p | GCGGGGGTGGCGGCGGG |  | 34 | 0.227272727 |
| ssc-miR-novel-chr5_29298 | ssc-mir-novel-chr5_29298 | 3p | GCGGGGGTGGCGGCGGG |  | 3 | 0.769230769 |
| ssc-miR-novel-chr5_29391 | ssc-mir-novel-chr5_29391 | 5p | TGCGTTCATTCTGTTTGGCCT | 6 |  | 1.6 |
| ssc-miR-novel-chr5_29426 | ssc-mir-novel-chr5_29426 | 3p | TGATGTTTATCCGAATCCTCAGA |  | 1 | 0.909090909 |
| ssc-miR-novel-chr5_29480 | ssc-mir-novel-chr5_29480 | 3p | TCTGAGATGTGACCTGGGCAT | 32 | 7 | 2.470588235 |
| ssc-miR-novel-chr5_29487 | ssc-mir-novel-chr5_29487 | 5p | GACTTAATGGCTGGCTGGGAGG | 39 | 2 | 4.083333333 |
| ssc-miR-novel-chr5_29564 | ssc-mir-novel-chr5_29564 | 3p | CAGTGTGGGACCTTGGGCCTCC |  |  |  |
| ssc-miR-novel-chr5_29627 | ssc-mir-novel-chr5_29627 | 5p | TCTCCAGTGAGACAGTCTCT |  |  |  |
| ssc-miR-novel-chr5_29674 | ssc-mir-novel-chr5_29674 | 3p | TAACACTGTCTGGTAAAGATG | 257 | 354 | 0.733516484 |
| ssc-miR-novel-chr5_29676 | ssc-mir-novel-chr5_29676 | 3p | TAATACTGCCGGGTAATGATGGA | 2 | 199 | 0.057416268 |
| ssc-miR-novel-chr5_29710 | ssc-mir-novel-chr5_29710 | 3p | TGTCGATGATGGCAGTGCTGAGG |  |  |  |
| ssc-miR-novel-chr5_29774 | ssc-mir-novel-chr5_29774 | 3p | TCGGCTGCAGACACCACGCC |  |  |  |
| ssc-miR-novel-chr5_29781 | ssc-mir-novel-chr5_29781 | 5p | TAAAGTTATATAAGGGTTTTTG |  |  |  |
| ssc-miR-novel-chr5_29793 | ssc-mir-novel-chr5_29793 | 5p | AAATGAAAAGGATTGGTTTCT |  |  |  |
| ssc-miR-novel-chr5_29857 | ssc-mir-novel-chr5_29857 | 5p | GCAGCAGGTCTCCAAGGGG | 4 | 736 | 0.018766756 |
| ssc-miR-novel-chr5_29868 | ssc-mir-novel-chr5_29868 | 3p | TCTCAGGGATGGTGGAAATAGCCT |  |  |  |
| ssc-miR-novel-chr5_29905 | ssc-mir-novel-chr5_29905 | 5p | ACTCTAGCTGCCAAAGGCGCT |  | 1 | 0.909090909 |
| ssc-miR-novel-chr5_30018 | ssc-mir-novel-chr5_30018 | 3p | CAAGGCCACTGACTGAAGAGCAGA |  |  |  |
| ssc-miR-novel-chr5_30028 | ssc-mir-novel-chr5_30028 | 3p | TGACTCCCGGCCTGCTGGCAGG |  |  |  |
| ssc-miR-novel-chr6_30041 | ssc-mir-novel-chr6_30041 | 3p | GGCTGGGAGCCTGGGCTTCGTGG |  |  |  |
| ssc-miR-novel-chr6_30050 | ssc-mir-novel-chr6_30050 | 5p | TGAGGGCCTCGCCAGCCCCCGGC |  |  |  |
| ssc-miR-novel-chr6_30116 | ssc-mir-novel-chr6_30116 | 3p | CACGAGAAACCGCGTCCCGCCAGG |  |  |  |
| ssc-miR-novel-chr6_30192 | ssc-mir-novel-chr6_30192 | 5p | TCCTCTGGAGGCTCGAGAAGA |  |  |  |
| ssc-miR-novel-chr6_30243 | ssc-mir-novel-chr6_30243 | 5p | AGGAGGAAGAAGAAACTG |  |  |  |
| ssc-miR-novel-chr6_30244 | ssc-mir-novel-chr6_30244 | 5p | AGGAGGAAGAAGAAACTG |  |  |  |
| ssc-miR-novel-chr6_30278 | ssc-mir-novel-chr6_30278 | 5p | AGGAAAGTGTGGTGGAGG |  |  |  |
| ssc-miR-novel-chr6_30281 | ssc-mir-novel-chr6_30281 | 3p | ATCCGGGAGCTGGGAGCC |  |  |  |
| ssc-miR-novel-chr6_30340 | ssc-mir-novel-chr6_30340 | 5p | TCCCGGGGTGTGGGGAGG | 4 | 7 | 0.823529412 |
| ssc-miR-novel-chr6_30650 | ssc-mir-novel-chr6_30650 | 5p | ACTCAAACTGTGGGGGCACTT | 3 |  | 1.3 |
| ssc-miR-novel-chr6_30675 | ssc-mir-novel-chr6_30675 | 3p | GACTCCTGCCCCTCTCCCACAGG |  |  |  |
| ssc-miR-novel-chr6_30729 | ssc-mir-novel-chr6_30729 | 3p | TAATACTGCCTGGTAATGATGA | 13 | 1317 | 0.017332329 |
| ssc-miR-novel-chr6_30750 | ssc-mir-novel-chr6_30750 | 5p | CGGGGAGGCTGTGCAGCGCGGC |  |  |  |
| ssc-miR-novel-chr6_30792 | ssc-mir-novel-chr6_30792 | 5p | GGGGCCGGGGGTGGGGCC |  |  |  |
| ssc-miR-novel-chr6_30857 | ssc-mir-novel-chr6_30857 | 3p | CATGGATGTGGTGATGTGG | 38 | 530 | 0.088888889 |
| ssc-miR-novel-chr6_30884 | ssc-mir-novel-chr6_30884 | 5p | TAGCAGGAGGAGGACTCTG |  |  |  |
| ssc-miR-novel-chr6_30922 | ssc-mir-novel-chr6_30922 | 5p | ACCAACGTGGATACCCCGGG | 5 |  | 1.5 |
| ssc-miR-novel-chr6_30930 | ssc-mir-novel-chr6_30930 | 5p | TTGCTGTGATGACAAAT | 2 | 9 | 0.631578947 |
| ssc-miR-novel-chr6_30947 | ssc-mir-novel-chr6_30947 | 3p | CGGGCGGGAGCGGCCGGG |  | 4 | 0.714285714 |
| ssc-miR-novel-chr6_31079 | ssc-mir-novel-chr6_31079 | 3p | TGAGATGAAGCTCTGTGTCT |  |  |  |
| ssc-miR-novel-chr6_31216 | ssc-mir-novel-chr6_31216 | 5p | GTTTTGGTGGGGGCCGGGG | 4 | 51 | 0.229508197 |
| ssc-miR-novel-chr6_31226 | ssc-mir-novel-chr6_31226 | 5p | CAAGTCTTTGGGTTCCAG | 2 | 7 | 0.705882353 |
| ssc-miR-novel-chr6_31268 | ssc-mir-novel-chr6_31268 | 5p | TCATGATATAGAGGTAAATAGT | 9 | 1 | 1.727272727 |
| ssc-miR-novel-chr6_31382 | ssc-mir-novel-chr6_31382 | 5p | CTATGTGCCTGAGAACTT |  |  |  |
| ssc-miR-novel-chr6_31392 | ssc-mir-novel-chr6_31392 | 5p | TCTCAGGATGAGGTAAGATTGCT |  |  |  |
| ssc-miR-novel-chr6_31419 | ssc-mir-novel-chr6_31419 | 3p | TGCTGTGATGGGGGCTCTGAGA |  |  |  |
| ssc-miR-novel-chr6_31436 | ssc-mir-novel-chr6_31436 | 5p | CGCTTCGGCGGCGGGGG | 72 | 16 | 3.153846154 |
| ssc-miR-novel-chr6_31474 | ssc-mir-novel-chr6_31474 | 5p | AGGTGGGATCCCGAGGC | 31 | 150 | 0.25625 |
| ssc-miR-novel-chr6_31487 | ssc-mir-novel-chr6_31487 | 3p | CAAGTGCTAATGTTGGGA |  |  |  |
| ssc-miR-novel-chr6_31513 | ssc-mir-novel-chr6_31513 | 5p | CTCCCCCCCTTCCCGGG |  |  |  |
| ssc-miR-novel-chr6_31604 | ssc-mir-novel-chr6_31604 | 3p | TGTGTGTGGGCGCCGGACGCC | 18 | 25 | 0.8 |
| ssc-miR-novel-chr6_31692 | ssc-mir-novel-chr6_31692 | 3p | TCTGGCTGTGGTGTAGACCGTC | 167 | 3 | 13.61538462 |
| ssc-miR-novel-chr6_31694 | ssc-mir-novel-chr6_31694 | 3p | TTGCAGTGTGCTGAAACCTCGGC |  | 2 | 0.833333333 |
| ssc-miR-novel-chr6_31755 | ssc-mir-novel-chr6_31755 | 5p | TCCTCTGGAGGCTCGAGAAGA |  |  |  |
| ssc-miR-novel-chr6_31759 | ssc-mir-novel-chr6_31759 | 5p | TGGCTTGTACTCTAGGTG |  |  |  |
| ssc-miR-novel-chr6_31814 | ssc-mir-novel-chr6_31814 | 3p | TGGGTGGAGGAAAGCGG |  |  |  |
| ssc-miR-novel-chr6_31822 | ssc-mir-novel-chr6_31822 | 3p | GAGTTCTGGGCTGCAGTG | 6 | 2 | 1.333333333 |
| ssc-miR-novel-chr6_31936 | ssc-mir-novel-chr6_31936 | 3p | ACTGCTGTGCTTTCTGCCTGC |  |  |  |
| ssc-miR-novel-chr6_32013 | ssc-mir-novel-chr6_32013 | 5p | CGGAGTGGGTCGGTTTAAGG |  |  |  |
| ssc-miR-novel-chr6_32075 | ssc-mir-novel-chr6_32075 | 5p | ATGATGGAGGAGCGAGCGGCCG |  |  |  |
| ssc-miR-novel-chr6_32122 | ssc-mir-novel-chr6_32122 | 3p | TTCGCCGCGCAGCCTGCTGGGA |  |  |  |
| ssc-miR-novel-chr6_32142 | ssc-mir-novel-chr6_32142 | 3p | GACACACACAGCGCTCCTGGCC |  |  |  |
| ssc-miR-novel-chr6_32152 | ssc-mir-novel-chr6_32152 | 3p | GACGGGCAGTGTGCTAGGATCC |  |  |  |
| ssc-miR-novel-chr6_32276 | ssc-mir-novel-chr6_32276 | 3p | CTGGGCCCGGGTGGAGC |  | 28 | 0.263157895 |
| ssc-miR-novel-chr6_32385 | ssc-mir-novel-chr6_32385 | 5p | TTCCCTTTGTCATCCTTTGCCC |  |  |  |
| ssc-miR-novel-chr6_32446 | ssc-mir-novel-chr6_32446 | 3p | ACTAGAGCGATTGGAAGG |  |  |  |
| ssc-miR-novel-chr6_32462 | ssc-mir-novel-chr6_32462 | 3p | TTGGCATGAATGTCGCGCTGG |  |  |  |
| ssc-miR-novel-chr6_32491 | ssc-mir-novel-chr6_32491 | 5p | CAAAGCGCCAGAGAGGCGGGGA |  |  |  |
| ssc-miR-novel-chr6_32492 | ssc-mir-novel-chr6_32492 | 3p | CAAAGCGCCAGAGAGGCGGGGA |  |  |  |
| ssc-miR-novel-chr6_32552 | ssc-mir-novel-chr6_32552 | 3p | TTGGACTGGAGGTGAGGC |  |  |  |
| ssc-miR-novel-chr6_32557 | ssc-mir-novel-chr6_32557 | 5p | AGCTTATCAGACTGGTGTAG |  |  |  |
| ssc-miR-novel-chr6_32953 | ssc-mir-novel-chr6_32953 | 5p | AAAGGGACACAGGAGGCT |  |  |  |
| ssc-miR-novel-chr7_33108 | ssc-mir-novel-chr7_33108 | 5p | TCTGGTCCAGACACTGTGGAGC |  |  |  |
| ssc-miR-novel-chr7_33374 | ssc-mir-novel-chr7_33374 | 5p | CTTCCAGCAAGGAGTCTCAGA |  |  |  |
| ssc-miR-novel-chr7_33433 | ssc-mir-novel-chr7_33433 | 3p | TCGAGAATTGCGTTTGGACAAT |  |  |  |
| ssc-miR-novel-chr7_33466 | ssc-mir-novel-chr7_33466 | 5p | TGCAGGTGGAGCCCAGGGGA |  |  |  |
| ssc-miR-novel-chr7_33502 | ssc-mir-novel-chr7_33502 | 5p | TGTCATGCTGGGGAGTGTAGTGA |  | 1 | 0.909090909 |
| ssc-miR-novel-chr7_33539 | ssc-mir-novel-chr7_33539 | 3p | TTTTTTGCTGGAACATTTCTG |  |  |  |
| ssc-miR-novel-chr7_33544 | ssc-mir-novel-chr7_33544 | 5p | TAGGACACAAAATGTAGGAAGGGC |  |  |  |
| ssc-miR-novel-chr7_33656 | ssc-mir-novel-chr7_33656 | 3p | AACGGAAACAATCCAAAACTGT |  |  |  |
| ssc-miR-novel-chr7_33696 | ssc-mir-novel-chr7_33696 | 5p | TCTGGTTCTGTGACCCTGCCT | 4 |  | 1.4 |
| ssc-miR-novel-chr7_33739 | ssc-mir-novel-chr7_33739 | 3p | ATCTGAAAGTACTGGGGGCCT | 4 |  | 1.4 |
| ssc-miR-novel-chr7_33821 | ssc-mir-novel-chr7_33821 | 3p | CAAAACAGAGAATCAAGACTGA | 9 |  | 1.9 |
| ssc-miR-novel-chr7_33839 | ssc-mir-novel-chr7_33839 | 3p | CCGCCTCCTCTCGCCGCC | 2 | 1 | 1.090909091 |
| ssc-miR-novel-chr7_33933 | ssc-mir-novel-chr7_33933 | 3p | AGTAGATTCATGGATACTCT | 7 |  | 1.7 |
| ssc-miR-novel-chr7_33975 | ssc-mir-novel-chr7_33975 | 3p | TGCATCTGTGGCGTAGGCCAGT | 14 | 105 | 0.208695652 |
| ssc-miR-novel-chr7_34009 | ssc-mir-novel-chr7_34009 | 3p | TCCAGGGTGCTCGCCCCAGC |  |  |  |
| ssc-miR-novel-chr7_34024 | ssc-mir-novel-chr7_34024 | 5p | TGATGCCTGGCATTTTGTGTGT |  |  |  |
| ssc-miR-novel-chr7_34189 | ssc-mir-novel-chr7_34189 | 3p | GTTCAAGTCCAGTTCTG | 13 |  | 2.3 |
| ssc-miR-novel-chr7_34218 | ssc-mir-novel-chr7_34218 | 5p | GTGGTCAAGGCCTGTCGCCGCTGT | 2 |  | 1.2 |
| ssc-miR-novel-chr7_34297 | ssc-mir-novel-chr7_34297 | 3p | TGCATCTGTGGCGTAGGCCAGT | 14 | 105 | 0.208695652 |
| ssc-miR-novel-chr7_34314 | ssc-mir-novel-chr7_34314 | 3p | CCTTTCTGTGTTCGAGGCC |  |  |  |
| ssc-miR-novel-chr7_34318 | ssc-mir-novel-chr7_34318 | 3p | AGGCCCTGGAAAATGACTG |  |  |  |
| ssc-miR-novel-chr7_34332 | ssc-mir-novel-chr7_34332 | 3p | TCTAGCATCGAGCACCCGCCT |  |  |  |
| ssc-miR-novel-chr7_34491 | ssc-mir-novel-chr7_34491 | 5p | TACGTGCTAGTTCTGTACTGGG |  |  |  |
| ssc-miR-novel-chr7_34798 | ssc-mir-novel-chr7_34798 | 3p | AGTCCCATCTGGGTCGCCA | 16 |  | 2.6 |
| ssc-miR-novel-chr7_34809 | ssc-mir-novel-chr7_34809 | 5p | CAGGTGGTGGACTTTCAGC |  |  |  |
| ssc-miR-novel-chr7_34819 | ssc-mir-novel-chr7_34819 | 5p | TCTCTGGGCCTGTGTCT |  |  |  |
| ssc-miR-novel-chr7_34820 | ssc-mir-novel-chr7_34820 | 3p | TCTCTGGGCCTGTGTCT |  |  |  |
| ssc-miR-novel-chr7_34843 | ssc-mir-novel-chr7_34843 | 5p | GCGGGAGCTCTGTCGCT | 2 |  | 1.2 |
| ssc-miR-novel-chr7_34845 | ssc-mir-novel-chr7_34845 | 5p | AAAGGATGGATTGGACAGGCCT | 2 |  | 1.2 |
| ssc-miR-novel-chr7_34976 | ssc-mir-novel-chr7_34976 | 3p | TGAGAACTCTGCTGAAGG |  |  |  |
| ssc-miR-novel-chr7_35006 | ssc-mir-novel-chr7_35006 | 3p | TTCCAGTGGATCTGGGGATGGA |  |  |  |
| ssc-miR-novel-chr7_35053 | ssc-mir-novel-chr7_35053 | 5p | TGCTGTAATTTACTCTCACC |  |  |  |
| ssc-miR-novel-chr7_35084 | ssc-mir-novel-chr7_35084 | 3p | CAAAACAGAGAATCAAGACTGA | 9 |  | 1.9 |
| ssc-miR-novel-chr7_35242 | ssc-mir-novel-chr7_35242 | 3p | ATAGCCTTGTTTGTTTTCTAGG |  |  |  |
| ssc-miR-novel-chr7_35271 | ssc-mir-novel-chr7_35271 | 5p | TGGTGTAGATCTCAGACGCAGC |  | 8 | 0.555555556 |
| ssc-miR-novel-chr7_35273 | ssc-mir-novel-chr7_35273 | 5p | TGATGCCTGGCATTTTGTGTGT |  |  |  |
| ssc-miR-novel-chr7_35375 | ssc-mir-novel-chr7_35375 | 5p | TTGCCCGAGAGCTTGGACCG | 4 |  | 1.4 |
| ssc-miR-novel-chr7_35436 | ssc-mir-novel-chr7_35436 | 3p | GTGCACCTGGGCAAGGAT |  |  |  |
| ssc-miR-novel-chr7_35438 | ssc-mir-novel-chr7_35438 | 3p | GTGCACCTGGGCAAGGAT |  |  |  |
| ssc-miR-novel-chr7_35451 | ssc-mir-novel-chr7_35451 | 5p | TGTGCTGTGCAGTAGGACC | 2 |  | 1.2 |
| ssc-miR-novel-chr7_35509 | ssc-mir-novel-chr7_35509 | 5p | GGGCGGGGGTCCGCCGG | 2 | 5 | 0.8 |
| ssc-miR-novel-chr7_35579 | ssc-mir-novel-chr7_35579 | 5p | GTGCCCAGAGTCGGAAGG |  |  |  |
| ssc-miR-novel-chr8_35622 | ssc-mir-novel-chr8_35622 | 5p | CCGGCCGCGCGCGCGCG | 9 | 1 | 1.727272727 |
| ssc-miR-novel-chr8_35968 | ssc-mir-novel-chr8_35968 | 5p | TGAGCAAGTAGATTGTATAG |  | 1 | 0.909090909 |
| ssc-miR-novel-chr8_36091 | ssc-mir-novel-chr8_36091 | 3p | TATAAATTGGAATATCTT |  |  |  |
| ssc-miR-novel-chr8_36140 | ssc-mir-novel-chr8_36140 | 5p | GTGTATGTGCTTGGCTG | 3 |  | 1.3 |
| ssc-miR-novel-chr8_36342 | ssc-mir-novel-chr8_36342 | 5p | TGAGCCACAGAAACTCCAGGAC |  |  |  |
| ssc-miR-novel-chr8_36412 | ssc-mir-novel-chr8_36412 | 5p | GTGAGGGGCAGAGAGAGAGA |  |  |  |
| ssc-miR-novel-chr8_36469 | ssc-mir-novel-chr8_36469 | 3p | TGGACTGGATGACAATCTGCAGG |  |  |  |
| ssc-miR-novel-chr8_36485 | ssc-mir-novel-chr8_36485 | 3p | CAGATGGATGTAGATACAGATC |  |  |  |
| ssc-miR-novel-chr8_36503 | ssc-mir-novel-chr8_36503 | 3p | TTCTGGGAACCGGTTTTGCTGCT |  |  |  |
| ssc-miR-novel-chr8_36529 | ssc-mir-novel-chr8_36529 | 3p | CCTCCGGTCCGCCTGCGGTGG |  |  |  |
| ssc-miR-novel-chr8_36585 | ssc-mir-novel-chr8_36585 | 5p | AGATGGATTTTTGGAGATGG | 8 | 19 | 0.620689655 |
| ssc-miR-novel-chr8_36601 | ssc-mir-novel-chr8_36601 | 5p | CAGCAATTTATTTTCCAGTGA |  |  |  |
| ssc-miR-novel-chr8_36618 | ssc-mir-novel-chr8_36618 | 3p | TCTGTGGTTTGTTGAATCTGTCG |  |  |  |
| ssc-miR-novel-chr8_36650 | ssc-mir-novel-chr8_36650 | 3p | CTCAAGTGATGCCTCTGCTT |  |  |  |
| ssc-miR-novel-chr8_36861 | ssc-mir-novel-chr8_36861 | 5p | AACTGGATGTAAGATAGAATCAT |  |  |  |
| ssc-miR-novel-chr8_37121 | ssc-mir-novel-chr8_37121 | 5p | AACTGGATGTAAGATAGAATCAT |  |  |  |
| ssc-miR-novel-chr8_37190 | ssc-mir-novel-chr8_37190 | 3p | TCTGTGAACTAGAAACCTCTGG | 356 | 148 | 2.316455696 |
| ssc-miR-novel-chr8_37196 | ssc-mir-novel-chr8_37196 | 3p | CTCCTGGCTGGCTCGCCA | 2 | 1 | 1.090909091 |
| ssc-miR-novel-chr8_37351 | ssc-mir-novel-chr8_37351 | 5p | GGGCGGGGGTCCGCCGG | 2 | 5 | 0.8 |
| ssc-miR-novel-chr9_37440 | ssc-mir-novel-chr9_37440 | 5p | CTCCTGGCTGGCTCGCCA | 2 | 1 | 1.090909091 |
| ssc-miR-novel-chr9_37600 | ssc-mir-novel-chr9_37600 | 5p | AGGAAGCCCTGGAGGGG |  |  |  |
| ssc-miR-novel-chr9_37672 | ssc-mir-novel-chr9_37672 | 5p | TGGCAGTGGAATTAGTGATTGT |  |  |  |
| ssc-miR-novel-chr9_37686 | ssc-mir-novel-chr9_37686 | 5p | ACTGGCCTTGGAGTCAGAAGT |  |  |  |
| ssc-miR-novel-chr9_37717 | ssc-mir-novel-chr9_37717 | 3p | AGGGCCCTGGCAGGGTGGGA |  |  |  |
| ssc-miR-novel-chr9_37731 | ssc-mir-novel-chr9_37731 | 3p | GCAGCAGAGGAAAGGGTTC |  |  |  |
| ssc-miR-novel-chr9_37946 | ssc-mir-novel-chr9_37946 | 5p | CTGAGAGATGGGAGAGTG | 13 |  | 2.3 |
| ssc-miR-novel-chr9_37990 | ssc-mir-novel-chr9_37990 | 5p | TATGGCTTTTCATTCCTATGTGA | 6 | 1752 | 0.00908059 |
| ssc-miR-novel-chr9_38021 | ssc-mir-novel-chr9_38021 | 3p | TCTGGCGGGAAGTTGTGGTC | 13 |  | 2.3 |
| ssc-miR-novel-chr9_38059 | ssc-mir-novel-chr9_38059 | 3p | CGATACAGAGGACTGACTGTAC |  |  |  |
| ssc-miR-novel-chr9_38293 | ssc-mir-novel-chr9_38293 | 3p | CACAGCAAGTGTAGACAGGCA | 4 |  | 1.4 |
| ssc-miR-novel-chr9_38296 | ssc-mir-novel-chr9_38296 | 5p | TAACCAATGTGCAGACTACTGT |  | 2 | 0.833333333 |
| ssc-miR-novel-chr9_38471 | ssc-mir-novel-chr9_38471 | 3p | TCTCCTTGCGAGTCTCTGCTGCC |  |  |  |
| ssc-miR-novel-chr9_38476 | ssc-mir-novel-chr9_38476 | 5p | CAGCCTGGCTTCTCCTGACATGG | 4 |  | 1.4 |
| ssc-miR-novel-chr9_38517 | ssc-mir-novel-chr9_38517 | 3p | CACGCTTGTGTCGTTGGAGTGGC | 7 |  | 1.7 |
| ssc-miR-novel-chr9_38594 | ssc-mir-novel-chr9_38594 | 3p | TCGTGCACAGATGTGGTCTCG |  |  |  |
| ssc-miR-novel-chr9_38736 | ssc-mir-novel-chr9_38736 | 3p | GACTCCAAAGTCTGCCTC |  |  |  |
| ssc-miR-novel-chr9_38827 | ssc-mir-novel-chr9_38827 | 5p | AGGCAGTGTAATTAGCTGATTGT |  |  |  |
| ssc-miR-novel-chr9_38871 | ssc-mir-novel-chr9_38871 | 5p | AGAGACCTGCTGATACAGCTG |  |  |  |
| ssc-miR-novel-chr9_38959 | ssc-mir-novel-chr9_38959 | 5p | TACCTGGTTGATCCTGC | 4 | 40 | 0.28 |
| ssc-miR-novel-chr9_39012 | ssc-mir-novel-chr9_39012 | 3p | CTCCCTGAGCTGTCTCTG |  |  |  |
| ssc-miR-novel-chr9_39041 | ssc-mir-novel-chr9_39041 | 5p | TATGGCTTTTCATTCCTATGTGA | 6 | 1752 | 0.00908059 |
| ssc-miR-novel-chr9_39126 | ssc-mir-novel-chr9_39126 | 3p | TCACTGGAGTTTTGTTTCAACA | 47 | 5 | 3.8 |
| ssc-miR-novel-chr9_39325 | ssc-mir-novel-chr9_39325 | 5p | CCATCTGTGGGATTATGACTG | 9 | 14 | 0.791666667 |
| ssc-miR-novel-chr9_39438 | ssc-mir-novel-chr9_39438 | 3p | TTGAAAGGCTATTTCTTGGTC |  |  |  |
| ssc-miR-novel-chr9_39553 | ssc-mir-novel-chr9_39553 | 5p | TCTGTACGTGGGAGGGTGTGT | 4 |  | 1.4 |
| ssc-miR-novel-chr9_39595 | ssc-mir-novel-chr9_39595 | 5p | TCGGCAGCTGCTTGGCGTCCGAGG |  | 8 | 0.555555556 |
| ssc-miR-novel-chrPRV_425 | ssc-mir-novel-chrPRV_425 | 3p | TCTCACCCCTGGGTCCGTCGC | 3622 |  | 363.2 |
| ssc-miR-novel-chrPRV_428 | ssc-mir-novel-chrPRV_428 | 5p | CTCATCCCGTCAGACCTGCGCC | 736 |  | 74.6 |
| ssc-miR-novel-chrPRV_434 | ssc-mir-novel-chrPRV_434 | 5p | CGTACCGACCCGCCTACCAGGCA | 13 |  | 2.3 |
| ssc-miR-novel-chrPRV_435 | ssc-mir-novel-chrPRV_435 | 3p | ATGAGTGGATGGATGGAGGCGA | 224 |  | 23.4 |
| ssc-miR-novel-chrPRV_441 | ssc-mir-novel-chrPRV_441 | 5p | ACCCGCGGATGGCGAGGATG | 15 |  | 2.5 |
| ssc-miR-novel-chrX_39845 | ssc-mir-novel-chrX_39845 | 3p | CGGCGGCGACTCTGGACTC | 232 | 171 | 1.337016575 |
| ssc-miR-novel-chrX_39944 | ssc-mir-novel-chrX_39944 | 3p | CTCCCACGTGCAGGGTTTGCA |  | 5 | 0.666666667 |
| ssc-miR-novel-chrX_39950 | ssc-mir-novel-chrX_39950 | 5p | TAATCCTTGCTACCTGGGTGAGA | 4 | 89 | 0.141414141 |
| ssc-miR-novel-chrX_39952 | ssc-mir-novel-chrX_39952 | 5p | TACCCATTGCATATCGGAGTTG | 384 | 424 | 0.907834101 |
| ssc-miR-novel-chrX_39953 | ssc-mir-novel-chrX_39953 | 3p | ATGCACCTGGGCAAGGATT | 3 | 6 | 0.8125 |
| ssc-miR-novel-chrX_39968 | ssc-mir-novel-chrX_39968 | 3p | CTCCAAGCATCGTGACCCAGGTT |  |  |  |
| ssc-miR-novel-chrX_39978 | ssc-mir-novel-chrX_39978 | 3p | CTCCAAGCATCGTGACCCAGGTT |  |  |  |
| ssc-miR-novel-chrX_40068 | ssc-mir-novel-chrX_40068 | 5p | TGATAATACAACCTGATAAGTGC |  |  |  |
| ssc-miR-novel-chrX_40069 | ssc-mir-novel-chrX_40069 | 5p | TTACAATACAACCTGATAAGTGC |  |  |  |
| ssc-miR-novel-chrX_40077 | ssc-mir-novel-chrX_40077 | 3p | ATTCCTAGAAATTGTTCACAAT |  |  |  |
| ssc-miR-novel-chrX_40247 | ssc-mir-novel-chrX_40247 | 3p | TACCCAGAGCGTGCAGTGTGA | 2 | 7 | 0.705882353 |
| ssc-miR-novel-chrX_40249 | ssc-mir-novel-chrX_40249 | 5p | AGGTCCTCAATAAGTATTTGTT |  |  |  |
| ssc-miR-novel-chrX_40252 | ssc-mir-novel-chrX_40252 | 5p | TTCATTCGGCTGTCCAGATGTA | 29 | 69 | 0.493670886 |
| ssc-miR-novel-chrX_40254 | ssc-mir-novel-chrX_40254 | 5p | TGAGTACCGCCATGTCTGTTGGGA |  | 2 | 0.833333333 |
| ssc-miR-novel-chrX_40255 | ssc-mir-novel-chrX_40255 | 3p | TTGCATATGTAGGATGTCCCA | 5 | 1 | 1.363636364 |
| ssc-miR-novel-chrX_40262 | ssc-mir-novel-chrX_40262 | 5p | CCACCAGAAAGAGACTGTAGGG |  |  |  |
| ssc-miR-novel-chrX_40264 | ssc-mir-novel-chrX_40264 | 5p | CCACCAGAAAGAGACTGTAGGG |  |  |  |
| ssc-miR-novel-chrX_40266 | ssc-mir-novel-chrX_40266 | 5p | CCACCAGAAAGAGACTGTAGGG |  |  |  |
| ssc-miR-novel-chrX_40268 | ssc-mir-novel-chrX_40268 | 5p | CCACCAGAAAGAGACTGTAGGG |  |  |  |
| ssc-miR-novel-chrX_40461 | ssc-mir-novel-chrX_40461 | 3p | CAGAGGCCAGAGGGCAGGACGCT |  |  |  |
| ssc-miR-novel-chrX_40477 | ssc-mir-novel-chrX_40477 | 3p | CAGCAAGGGTGGTCCAGGGAGT |  |  |  |
| ssc-miR-novel-chrX_40484 | ssc-mir-novel-chrX_40484 | 5p | AAGGGCGGGAGCGGCGGG | 69 | 10 | 3.95 |
| ssc-miR-novel-chrX_40486 | ssc-mir-novel-chrX_40486 | 5p | TGCACGGAGCAGCAGGGTCTGA | 3 | 1 | 1.181818182 |
| ssc-miR-novel-chrX_40490 | ssc-mir-novel-chrX_40490 | 5p | GTAGGGCCGGCCCGCCCGGGA |  |  |  |
| ssc-miR-novel-chrX_40522 | ssc-mir-novel-chrX_40522 | 3p | CATGCCTTGAGTGTAGGAC | 41 | 18 | 1.821428571 |
| ssc-miR-novel-chrX_40528 | ssc-mir-novel-chrX_40528 | 3p | AACCAGACTCTGAGAGCAGGACT |  |  |  |
| ssc-miR-novel-chrX_40533 | ssc-mir-novel-chrX_40533 | 5p | GAAAATGGATGGCACTGGAGT | 37 | 20 | 1.566666667 |
| ssc-miR-novel-chrX_40539 | ssc-mir-novel-chrX_40539 | 5p | TGGTCTAGCGGTTAGGA |  | 11 | 0.476190476 |
| ssc-miR-novel-chrX_40608 | ssc-mir-novel-chrX_40608 | 5p | GGTGGAGGCATGTAGTTCCTAA |  |  |  |
| ssc-miR-novel-chrX_40705 | ssc-mir-novel-chrX_40705 | 5p | AGCTACATTGTCTGCTGGGTTT | 2545 | 147 | 16.27388535 |
| ssc-miR-novel-chrX_40783 | ssc-mir-novel-chrX_40783 | 5p | GTATGTGAGCGGGGGGCTGGTGGGA |  | 3 | 0.769230769 |
| ssc-miR-novel-chrX_40912 | ssc-mir-novel-chrX_40912 | 3p | AAAGCTGTGGATTCTGGCAAATGG |  |  |  |
| ssc-miR-novel-chrX_41000 | ssc-mir-novel-chrX_41000 | 5p | CACCTGGGAGGATCGGAG |  |  |  |
| ssc-miR-novel-chrX_41021 | ssc-mir-novel-chrX_41021 | 5p | CCACCAGAAAGAGACTGTAGGG |  |  |  |
| ssc-miR-novel-chrX_41061 | ssc-mir-novel-chrX_41061 | 5p | CCTGGACTTGAAGTCAGAAGGC | 2 | 7 | 0.705882353 |
| ssc-miR-novel-chrX_41131 | ssc-mir-novel-chrX_41131 | 5p | TGGAAGGTGGATCTGGGC |  |  |  |
| ssc-miR-novel-chrX_41178 | ssc-mir-novel-chrX_41178 | 3p | TGAACGGTGCCTGTGTGGCTAGA |  |  |  |
| ssc-miR-novel-chrX_41179 | ssc-mir-novel-chrX_41179 | 5p | TATCCAGACAGGTGCTGTTCT |  |  |  |
| ssc-miR-novel-chrX_41184 | ssc-mir-novel-chrX_41184 | 3p | TGAACGGCGCCTGTGTGGTTAGA |  | 2 | 0.833333333 |
| ssc-miR-novel-chrX_41186 | ssc-mir-novel-chrX_41186 | 3p | TGAACAGGGCCTTTCTGGGTCGAG |  | 1 | 0.909090909 |
| ssc-miR-novel-chrX_41188 | ssc-mir-novel-chrX_41188 | 3p | CAACAGTCCCTGCCTGGGTAGA |  |  |  |
| ssc-miR-novel-chrX_41190 | ssc-mir-novel-chrX_41190 | 3p | AATGGCGCTTTTTTGTGAAGA | 2 | 431 | 0.027210884 |
| ssc-miR-novel-chrX_41192 | ssc-mir-novel-chrX_41192 | 3p | TGAATGGCGCCTTTCTGAGTAGA | 6 | 17 | 0.592592593 |
| ssc-miR-novel-chrX_41194 | ssc-mir-novel-chrX_41194 | 3p | TGATTGGCACCTCTTTGAGTGA |  | 30 | 0.25 |
| ssc-miR-novel-chrX_41196 | ssc-mir-novel-chrX_41196 | 3p | ACTGTCACCTTTTTGAGTAGA | 4 | 9 | 0.736842105 |
| ssc-miR-novel-chrX_41200 | ssc-mir-novel-chrX_41200 | 3p | TGATTGACACCTCTGTTAGTGGA |  | 16 | 0.384615385 |
| ssc-miR-novel-chrX_41202 | ssc-mir-novel-chrX_41202 | 3p | TGATTGGCACCTCTTGGAGTGA | 2 | 29 | 0.307692308 |
| ssc-miR-novel-GL892353-1_41323 | ssc-mir-novel-GL892353-1_41323 | 5p | CGACATGGACACGCGTCATGA |  |  |  |
| ssc-miR-novel-GL892520-2_41438 | ssc-mir-novel-GL892520-2_41438 | 5p | GGTGATGATGACGATGAAGCTGAAA |  | 7 | 0.588235294 |
| ssc-miR-novel-GL892841-1_41670 | ssc-mir-novel-GL892841-1_41670 | 5p | GAGACCATGGAGAGAAAAA | 4 |  | 1.4 |
| ssc-miR-novel-GL892848-2_41682 | ssc-mir-novel-GL892848-2_41682 | 5p | GTGGTCAAGGCCTGTCGCCGCTGT | 2 |  | 1.2 |
| ssc-miR-novel-GL892871-2_41708 | ssc-mir-novel-GL892871-2_41708 | 5p | TTCAAGTAATTCAGGATAGGTT | 2094 | 451 | 4.563991323 |
| ssc-miR-novel-GL892918-2_41750 | ssc-mir-novel-GL892918-2_41750 | 5p | CTAGAGTGGATTCTGCA |  |  |  |
| ssc-miR-novel-GL892946-2_41815 | ssc-mir-novel-GL892946-2_41815 | 3p | TCGGAGCAGCGCGCGAGA | 6 | 4 | 1.142857143 |
| ssc-miR-novel-GL892961-2_41842 | ssc-mir-novel-GL892961-2_41842 | 3p | CAGACCCTGAGCTGCCTCTAGA | 3 | 2 | 1.083333333 |
| ssc-miR-novel-GL893103-2_41962 | ssc-mir-novel-GL893103-2_41962 | 3p | AGTCCTCTCCTGGGCACCT |  |  |  |
| ssc-miR-novel-GL893138-2_41998 | ssc-mir-novel-GL893138-2_41998 | 5p | GGCTCCCTCCACCCGCC |  | 6 | 0.625 |
| ssc-miR-novel-GL893173-1_42029 | ssc-mir-novel-GL893173-1_42029 | 3p | CCTTCGAGGATGCGGATGTCACC | 2 |  | 1.2 |
| ssc-miR-novel-GL893222-2_42067 | ssc-mir-novel-GL893222-2_42067 | 3p | AGAGGGCTGTGGGAGAGA | 10 |  | 2 |
| ssc-miR-novel-GL893230-2_42082 | ssc-mir-novel-GL893230-2_42082 | 3p | CTGTGCTTCTGCTCGGA |  |  |  |
| ssc-miR-novel-GL893233-1_42087 | ssc-mir-novel-GL893233-1_42087 | 5p | ATTGAGAACACTGACATAACA |  |  |  |
| ssc-miR-novel-GL893271-1_42121 | ssc-mir-novel-GL893271-1_42121 | 5p | AGAGGTGCGGCTTTGGCTGGA |  | 1 | 0.909090909 |
| ssc-miR-novel-GL893334-2_42198 | ssc-mir-novel-GL893334-2_42198 | 5p | TCTGGAGGACGCTAGAGCTGGGC |  |  |  |
| ssc-miR-novel-GL893334-2_42199 | ssc-mir-novel-GL893334-2_42199 | 3p | CAGAAGGGGAGTCGGAGCAGA |  |  |  |
| ssc-miR-novel-GL893571-2_42428 | ssc-mir-novel-GL893571-2_42428 | 5p | CACGGGTTCGATCCCTGGTGTGGGC |  |  |  |
| ssc-miR-novel-GL893616-2_42510 | ssc-mir-novel-GL893616-2_42510 | 3p | CTCAGACCTCAGGCTTGGAGCC | 3 |  | 1.3 |
| ssc-miR-novel-GL893653-2_42520 | ssc-mir-novel-GL893653-2_42520 | 5p | TTCAGGGTCCAGGATTGCTATAG | 2 | 1 | 1.090909091 |
| ssc-miR-novel-GL893741-1_42616 | ssc-mir-novel-GL893741-1_42616 | 5p | GGTGAGCACTCTGGACT |  | 4 | 0.714285714 |
| ssc-miR-novel-GL893763-1_42633 | ssc-mir-novel-GL893763-1_42633 | 3p | TCTTTCCTTGATTAAAACTGG | 4 |  | 1.4 |
| ssc-miR-novel-GL893833-1_42684 | ssc-mir-novel-GL893833-1_42684 | 3p | TTTTTGATGGGAGAAGAGAGA |  |  |  |
| ssc-miR-novel-GL894036-2_42873 | ssc-mir-novel-GL894036-2_42873 | 3p | CAAACCAGCACGTCAAGGGCCC |  |  |  |
| ssc-miR-novel-GL894094-2_42949 | ssc-mir-novel-GL894094-2_42949 | 3p | GCATTGGGGGTTCAGGGG | 12 | 15 | 0.88 |
| ssc-miR-novel-GL894224-1_43070 | ssc-mir-novel-GL894224-1_43070 | 5p | CTTTGGATCTCTGGTGACAGG |  |  |  |
| ssc-miR-novel-GL894231-1_43077 | ssc-mir-novel-GL894231-1_43077 | 5p | TGGTCGACCAGTTGGAAAGTAAT | 7 | 5 | 1.133333333 |
| ssc-miR-novel-GL894231-1_43090 | ssc-mir-novel-GL894231-1_43090 | 3p | CCCAATACACGGTCGATCTCT |  | 3 | 0.769230769 |
| ssc-miR-novel-GL894231-1_43091 | ssc-mir-novel-GL894231-1_43091 | 5p | AGAGGCTGGCCGTGATGAATTCG |  | 1 | 0.909090909 |
| ssc-miR-novel-GL894231-1_43093 | ssc-mir-novel-GL894231-1_43093 | 5p | TGTGACTGGTTGACCAGAGGGT |  |  |  |
| ssc-miR-novel-GL894231-1_43098 | ssc-mir-novel-GL894231-1_43098 | 3p | TGTATGTCAACTGATCCACAGT |  | 4 | 0.714285714 |
| ssc-miR-novel-GL894231-1_43100 | ssc-mir-novel-GL894231-1_43100 | 3p | ATAATACATGGTTAACCTCTTT | 2 |  | 1.2 |
| ssc-miR-novel-GL894231-1_43101 | ssc-mir-novel-GL894231-1_43101 | 5p | GGAGAAATTATCCTTGGTGTGTT |  | 1 | 0.909090909 |
| ssc-miR-novel-GL894231-1_43114 | ssc-mir-novel-GL894231-1_43114 | 3p | ATCATAGAGGAAAATCCACA |  |  |  |
| ssc-miR-novel-GL894266-1_43136 | ssc-mir-novel-GL894266-1_43136 | 5p | TTGTTTCTTGCATGTGCTCTGATT |  |  |  |
| ssc-miR-novel-GL894404-1_43244 | ssc-mir-novel-GL894404-1_43244 | 3p | TTGTGCGTTGGCAGGATGGGCCGGA |  |  |  |
| ssc-miR-novel-GL894430-2_43273 | ssc-mir-novel-GL894430-2_43273 | 3p | ATAAAACTTAGTCGCTGCT |  |  |  |
| ssc-miR-novel-GL894430-2_43278 | ssc-mir-novel-GL894430-2_43278 | 3p | ATAAAACTTAGTCGCTGCT |  |  |  |
| ssc-miR-novel-GL894520-2_43333 | ssc-mir-novel-GL894520-2_43333 | 3p | CTCAAGTGATGCCTCTGCTT |  |  |  |
| ssc-miR-novel-GL894542-2_43355 | ssc-mir-novel-GL894542-2_43355 | 5p | TTTGCTCTGCTCCTGCCACAT |  |  |  |
| ssc-miR-novel-GL894570-2_43376 | ssc-mir-novel-GL894570-2_43376 | 3p | TGTTGTCCTCCGAATCTGAAT |  |  |  |
| ssc-miR-novel-GL894677-1_43524 | ssc-mir-novel-GL894677-1_43524 | 3p | TTCACTTTGGATTTTCAGCC | 12 |  | 2.2 |
| ssc-miR-novel-GL894726-2_43559 | ssc-mir-novel-GL894726-2_43559 | 3p | CGGGGCAGCTCAGTACAGGAC |  |  |  |
| ssc-miR-novel-GL894726-2_43560 | ssc-mir-novel-GL894726-2_43560 | 5p | CGGGGCAGCTCAGTACAGGAC |  |  |  |
| ssc-miR-novel-GL894850-1_43647 | ssc-mir-novel-GL894850-1_43647 | 5p | CAGGACTGGTGACTGGGGTG |  |  |  |
| ssc-miR-novel-GL894875-2_43662 | ssc-mir-novel-GL894875-2_43662 | 5p | TGAGGTGGTAGATGGTATAG |  |  |  |
| ssc-miR-novel-GL894932-2_43717 | ssc-mir-novel-GL894932-2_43717 | 5p | TTCCAGGGAAGAAAGGAGGAAC | 2 |  | 1.2 |
| ssc-miR-novel-GL895030-2_43791 | ssc-mir-novel-GL895030-2_43791 | 5p | GCAGAGCAGCGGCAGGAGG |  |  |  |
| ssc-miR-novel-GL895143-1_43893 | ssc-mir-novel-GL895143-1_43893 | 5p | TTCCTGGGTCTCGTGGTCTCAGTCT |  |  |  |
| ssc-miR-novel-GL895351-2_44043 | ssc-mir-novel-GL895351-2_44043 | 5p | GCGCAGCACATCATGGTTTA |  |  |  |
| ssc-miR-novel-GL895485-2_44130 | ssc-mir-novel-GL895485-2_44130 | 3p | AGGCTCTGATTGGATTGGGTG |  |  |  |
| ssc-miR-novel-GL895563-1_44191 | ssc-mir-novel-GL895563-1_44191 | 3p | TAGCCTGACGCTGATGATTGT |  |  |  |
| ssc-miR-novel-GL895621-2_44210 | ssc-mir-novel-GL895621-2_44210 | 5p | TCTTCCATGTGCCACGGGTGTAGCT |  |  |  |
| ssc-miR-novel-GL896133-1_44557 | ssc-mir-novel-GL896133-1_44557 | 5p | TGCTCAGAGGTCGGAGGTGGAG |  | 1 | 0.909090909 |
| ssc-miR-novel-GL896157-1_44574 | ssc-mir-novel-GL896157-1_44574 | 3p | TGGGCTCCAAACCCCTGTCCAGG |  |  |  |
| ssc-miR-novel-GL896208-1_44612 | ssc-mir-novel-GL896208-1_44612 | 3p | TCTGGCCTTGGCCTCAGGCCTGGC |  |  |  |
| ssc-miR-novel-GL896241-2_44636 | ssc-mir-novel-GL896241-2_44636 | 5p | TTCTGGAAGATGTAGTCTGGA |  |  |  |
| ssc-miR-novel-GL896292-1_44714 | ssc-mir-novel-GL896292-1_44714 | 3p | CTTCTCTGTTACTTCCCTCAGG |  |  |  |
| ssc-miR-novel-GL896302-1_44731 | ssc-mir-novel-GL896302-1_44731 | 3p | GCAAAGCACACGGCCTGCAGAGA | 4 | 22 | 0.4375 |
| ssc-miR-novel-GL896425-1_44856 | ssc-mir-novel-GL896425-1_44856 | 3p | TGGTGCCTGACGTCTTGGCAGT |  |  |  |
| ssc-miR-novel-GL896485-1_44921 | ssc-mir-novel-GL896485-1_44921 | 3p | AGAGGAGCCGCGACAGAGCCGG | 2 | 6 | 0.75 |
| ssc-miR-novel-GL896501-1_44926 | ssc-mir-novel-GL896501-1_44926 | 5p | TGTGGGGCCACGCCCTCAGGCTGT | 4 | 1 | 1.272727273 |
| ssc-miR-novel-JH118484-1_41578 | ssc-mir-novel-JH118484-1_41578 | 5p | CCGGACTCTCTAAATCTCACCT |  |  |  |
| ssc-miR-novel-JH118486-1_41595 | ssc-mir-novel-JH118486-1_41595 | 3p | TTATCCGGGCTTGTGCTGAAGC | 13 | 1 | 2.090909091 |
| ssc-miR-novel-JH118486-1_41599 | ssc-mir-novel-JH118486-1_41599 | 5p | AGCACTTACGGATTCTGGGGG | 2 | 2 | 1 |
| ssc-miR-novel-JH118494-1_41628 | ssc-mir-novel-JH118494-1_41628 | 3p | TCTGAGATGTGACCTGGGCAT | 32 | 7 | 2.470588235 |
| ssc-miR-novel-JH118511-1_41721 | ssc-mir-novel-JH118511-1_41721 | 3p | ATTGGGACTGAGACACG | 14 |  | 2.4 |
| ssc-miR-novel-JH118523-1_41818 | ssc-mir-novel-JH118523-1_41818 | 5p | AAGCCAGAGTCAGGGGACACTGT |  |  |  |
| ssc-miR-novel-JH118527-1_41847 | ssc-mir-novel-JH118527-1_41847 | 3p | CTCACGTCCATGGTCAGCACCGTG | 2 |  | 1.2 |
| ssc-miR-novel-JH118570-1_42024 | ssc-mir-novel-JH118570-1_42024 | 3p | CTCTCGGCTGCTGAGGCCT |  |  |  |
| ssc-miR-novel-JH118585-1_42104 | ssc-mir-novel-JH118585-1_42104 | 5p | TCTGGTCCAGACACTGTGGAGC |  |  |  |
| ssc-miR-novel-JH118620-1_42301 | ssc-mir-novel-JH118620-1_42301 | 3p | AATGATGCCCCTTAGAGTTGAGC |  |  |  |
| ssc-miR-novel-JH118636-1_42370 | ssc-mir-novel-JH118636-1_42370 | 3p | AGAGGGAGTAGGTTTCATA |  |  |  |
| ssc-miR-novel-JH118644-1_42413 | ssc-mir-novel-JH118644-1_42413 | 3p | GACTCCAAAGTCTGCCTC |  |  |  |
| ssc-miR-novel-JH118647-1_42435 | ssc-mir-novel-JH118647-1_42435 | 3p | TGTGTGGCTAAGTGGTAGATTT |  | 2 | 0.833333333 |
| ssc-miR-novel-JH118654-1_42487 | ssc-mir-novel-JH118654-1_42487 | 5p | TGGCAGTGTATTGTTAGCTGGT | 119 |  | 12.9 |
| ssc-miR-novel-JH118655-1_42490 | ssc-mir-novel-JH118655-1_42490 | 5p | AACACATGTGTGCTGAGTGGAACT | 2 | 1 | 1.090909091 |
| ssc-miR-novel-JH118656-1_42504 | ssc-mir-novel-JH118656-1_42504 | 5p | TGGCTCATTTCAGCAGGAAG | 4 | 6 | 0.875 |
| ssc-miR-novel-JH118676-1_42617 | ssc-mir-novel-JH118676-1_42617 | 3p | ACTGTGTGTGAGGAAGTC |  |  |  |
| ssc-miR-novel-JH118774-1_43297 | ssc-mir-novel-JH118774-1_43297 | 3p | TAACCCGACATTCAAGGCCTGT |  |  |  |
| ssc-miR-novel-JH118788-1_43363 | ssc-mir-novel-JH118788-1_43363 | 5p | AAAGGATGGATTGGACAGGCCT | 2 |  | 1.2 |
| ssc-miR-novel-JH118806-1_43515 | ssc-mir-novel-JH118806-1_43515 | 3p | ATTCATATGCAGCTGTAGGAA |  |  |  |
| ssc-miR-novel-JH118806-1_43518 | ssc-mir-novel-JH118806-1_43518 | 3p | ATTCATATGCAGCTGTAGGAA |  |  |  |
| ssc-miR-novel-JH118928-1_44242 | ssc-mir-novel-JH118928-1_44242 | 3p | TCATTCTCCTTCTTTGACCAGA |  |  |  |
| ssc-miR-novel-JH118939-1_44269 | ssc-mir-novel-JH118939-1_44269 | 5p | GACTTAATGGCTGGCTGGGAGG | 39 | 2 | 4.083333333 |
| ssc-miR-novel-JH118943-1_44283 | ssc-mir-novel-JH118943-1_44283 | 3p | TGAATTACTGTGGATTCTTGG |  |  |  |
| ssc-miR-novel-JH118951-1_44333 | ssc-mir-novel-JH118951-1_44333 | 3p | TCAAGGTCCGCTGTGAACACGG | 4 |  | 1.4 |
| ssc-miR-novel-JH118990-1_44625 | ssc-mir-novel-JH118990-1_44625 | 3p | CACGCTTGTGTCGTTGGAGTGGC | 7 |  | 1.7 |
| ssc-miR-novel-JH118993-1_44650 | ssc-mir-novel-JH118993-1_44650 | 3p | TTGGACTGGAGGTGAGGC |  |  |  |
| ssc-miR-novel-JH118993-1_44655 | ssc-mir-novel-JH118993-1_44655 | 5p | AGCTTATCAGACTGGTGTAG |  |  |  |

| **Mature miRNA-ID** | **Pre-miRNA_ID** | **Pre-miRNA arm (5p or 3p)** | **Mature miRNA -SEQ** | **TagCount (PRV FaΔgE/gI strain infected PK-15 cells)** | **TagCount (****PRV Fa wild strain infected PK-15 cells )** | **Fold Change (PRV FaΔgE/gI strain infected vs PRV Fa wild strain infected)** |
| --- | --- | --- | --- | --- | --- | --- |
| ssc-let-7a | ssc-let-7a-1 | 5p | TGAGGTAGTAGGTTGTATAGTT | 9095 | 10064 | 0.903811793 |
| ssc-let-7a | ssc-let-7a-2 | 5p | TGAGGTAGTAGGTTGTATAGTT | 9095 | 10064 | 0.903811793 |
| ssc-let-7c | ssc-let-7c | 5p | TGAGGTAGTAGGTTGTATGGTT | 995 | 1098 | 0.907039711 |
| ssc-let-7d-5p | ssc-let-7d | 5p | AGAGGTAGTAGGTTGCATAGTT | 372 | 271 | 1.359430605 |
| ssc-let-7d-3p | ssc-let-7d | 3p | CTATACGACCTGCTGCCTTTCT | 3 |  | 1.3 |
| ssc-let-7e | ssc-let-7e | 5p | TGAGGTAGGAGGTTGTATAGTT | 595 | 455 | 1.301075269 |
| ssc-let-7f | ssc-let-7f-1 | 5p | TGAGGTAGTAGATTGTATAGTT | 16471 | 16178 | 1.018099827 |
| ssc-let-7f | ssc-let-7f-2 | 5p | TGAGGTAGTAGATTGTATAGTT | 16471 | 16178 | 1.018099827 |
| ssc-let-7g | ssc-let-7g | 5p | TGAGGTAGTAGTTTGTACAGTT | 2526 | 5165 | 0.490048309 |
| ssc-let-7i | ssc-let-7i | 5p | TGAGGTAGTAGTTTGTGCT | 8500 | 6862 | 1.238358556 |
| ssc-miR-1 | ssc-mir-1 | 3p | TGGAATGTAAAGAAGTATGTA | 56 | 112 | 0.540983607 |
| ssc-miR-100 | ssc-mir-100 | 5p | AACCCGTAGATCCGAACTTGTG | 170 | 2446 | 0.073289902 |
| ssc-miR-101 | ssc-mir-101-1 | 3p | TACAGTACTGTGATAACTGAA | 324 | 397 | 0.820638821 |
| ssc-miR-101 | ssc-mir-101-2 | 3p | TACAGTACTGTGATAACTGAA | 324 | 397 | 0.820638821 |
| ssc-miR-103 | ssc-mir-103-1 | 3p | AGCAGCATTGTACAGGGCTATGA | 1338 | 886 | 1.504464286 |
| ssc-miR-103 | ssc-mir-103-2 | 3p | AGCAGCATTGTACAGGGCTATGA | 1338 | 886 | 1.504464286 |
| ssc-miR-105-2 | ssc-mir-105-2 | 5p | TCAAATGCTCAGACTCCTTG |  |  |  |
| ssc-miR-106a | ssc-mir-106a | 5p | AAAAGTGCTTACAGTGCAGGTAGC |  | 2 | 0.833333333 |
| ssc-miR-107 | ssc-mir-107 | 3p | AGCAGCATTGTACAGGGCTATCA | 235 | 104 | 2.149122807 |
| ssc-miR-10a-5p | ssc-mir-10a | 5p | TACCCTGTAGATCCGAATTTGT | 15805 | 25671 | 0.615824929 |
| ssc-miR-10a-3p | ssc-mir-10a | 3p | CAAATTCGTATCTAGGGGAAT | 72 | 255 | 0.309433962 |
| ssc-miR-10b | ssc-mir-10b | 5p | TACCCTGTAGAACCGAATTTGT | 139310 | 174810 | 0.796933989 |
| ssc-miR-122 | ssc-mir-122 | 5p | TGGAGTGTGACAATGGTGTTTGT |  | 8 | 0.555555556 |
| ssc-miR-1224 | ssc-mir-1224 | 3p | CACCTCCTCTCTCCTCAGGT |  |  |  |
| ssc-miR-1249 | ssc-mir-1249-1 | 3p | ACGCCCTTCCCCCCCTTCTTCA |  | 2 | 0.833333333 |
| ssc-miR-1249 | ssc-mir-1249-2 | 3p | ACGCCCTTCCCCCCCTTCTTCA |  | 2 | 0.833333333 |
| ssc-miR-124a | ssc-mir-124a-1 | 3p | TAAGGCACGCGGTGAATGCCA |  |  |  |
| ssc-miR-124a | ssc-mir-124a-2 | 3p | TAAGGCACGCGGTGAATGCCA |  |  |  |
| ssc-miR-125a | ssc-mir-125a | 5p | TCCCTGAGACCCTTTAACCTGTG | 38 | 23 | 1.454545455 |
| ssc-miR-125b | ssc-mir-125b-1 | 5p | TCCCTGAGACCCTAACTTGTGA | 56 | 83 | 0.709677419 |
| ssc-miR-125b | ssc-mir-125b-2 | 5p | TCCCTGAGACCCTAACTTGTGA | 56 | 83 | 0.709677419 |
| ssc-miR-126-5p | ssc-mir-126 | 5p | CATTATTACTTTTGGTACGCG | 36 | 43 | 0.867924528 |
| ssc-miR-126-3p | ssc-mir-126 | 3p | TCGTACCGTGAGTAATAATGCG | 17 | 73 | 0.325301205 |
| ssc-miR-127 | ssc-mir-127 | 3p | TCGGATCCGTCTGAGCTTGGCT | 2 |  | 1.2 |
| ssc-miR-1271 | ssc-mir-1271 | 3p | TGCCTGCTATGTGCCAGGCA |  |  |  |
| ssc-miR-1277 | ssc-mir-1277 | 3p | TACGTAGATATATATGTATTTT |  |  |  |
| ssc-miR-128 | ssc-mir-128-1 | 3p | TCACAGTGAACCGGTCTCTTT | 147 | 1185 | 0.131380753 |
| ssc-miR-128 | ssc-mir-128-2 | 3p | TCACAGTGAACCGGTCTCTTT | 147 | 1185 | 0.131380753 |
| ssc-miR-1285 | ssc-mir-1285 | 3p | CTGGGCAACATAGCGAGACCCCGT |  |  |  |
| ssc-miR-1296-5p | ssc-mir-1296 | 5p | TTAGGGCCCTGGCTCCATCTCC | 2 | 6 | 0.75 |
| ssc-miR-1296-3p | ssc-mir-1296 | 3p | GAGTGGGGTTTTGACCCTAACC |  |  |  |
| ssc-miR-129a | ssc-mir-129a | 3p | AAGCCCTTACCCCAAAAAGCAT | 3 |  | 1.3 |
| ssc-miR-129b | ssc-mir-129b | 5p | CTTTTTGCGGTCTGGGCTTGC | 137 | 45 | 2.672727273 |
| ssc-miR-1306-5p | ssc-mir-1306 | 5p | CCACCTCCCCTGCAAACGTCCA | 5 |  | 1.5 |
| ssc-miR-1306-3p | ssc-mir-1306 | 3p | ACGTTGGCTCTGGTGGTGATG |  | 14 | 0.416666667 |
| ssc-miR-1307 | ssc-mir-1307 | 3p | ACTCGGCGTGGCGTCGGTCGTG | 9 | 4 | 1.357142857 |
| ssc-miR-130a | ssc-mir-130a | 3p | CAGTGCAATGTTAAAAGGGCAT | 33 | 29 | 1.102564103 |
| ssc-miR-130b | ssc-mir-130b | 3p | CAGTGCAATGATGAAAGGGCAT | 32 | 94 | 0.403846154 |
| ssc-miR-132 | ssc-mir-132 | 3p | TAACAGTCTACAGCCATGGTCG | 20 | 4 | 2.142857143 |
| ssc-miR-133a-5p | ssc-mir-133a-1 | 5p | AGCTGGTAAAATGGAACCAAAT |  |  |  |
| ssc-miR-133a-3p | ssc-mir-133a-1 | 3p | TTGGTCCCCTTCAACCAGCTG |  | 2 | 0.833333333 |
| ssc-miR-133a-5p | ssc-mir-133a-2 | 5p | AGCTGGTAAAATGGAACCAAAT |  |  |  |
| ssc-miR-133a-3p | ssc-mir-133a-2 | 3p | TTGGTCCCCTTCAACCAGCTG |  | 2 | 0.833333333 |
| ssc-miR-133b | ssc-mir-133b | 3p | TTTGGTCCCCTTCAACCAGCTAT |  |  |  |
| ssc-miR-1343 | ssc-mir-1343 | 3p | CTCCTGGGGCCCGCACTCTCGC | 13 | 4 | 1.642857143 |
| ssc-miR-135 | ssc-mir-135-1 | 5p | TATGGCTTTTTATTCCTATGTGA |  | 4 | 0.714285714 |
| ssc-miR-135 | ssc-mir-135-2 | 5p | TATGGCTTTTTATTCCTATGTGA |  | 4 | 0.714285714 |
| ssc-miR-136 | ssc-mir-136 | 5p | ACTCCATTTGTTTTGATGATGGA |  |  |  |
| ssc-miR-137 | ssc-mir-137 | 3p | TTATTGCTTAAGAATACGCGTAG |  |  |  |
| ssc-miR-138 | ssc-mir-138 | 5p | AGCTGGTGTTGTGAATCAGGC |  | 7 | 0.588235294 |
| ssc-miR-139-5p | ssc-mir-139 | 5p | TCTACAGTGCACGTGTCTCCAG | 7 | 141 | 0.112582781 |
| ssc-miR-139-3p | ssc-mir-139 | 3p | TGGAGACGCGGCCCTGTTGGAGT | 3 | 2 | 1.083333333 |
| ssc-miR-140-5p | ssc-mir-140 | 5p | AGTGGTTTTACCCTATGGTAG | 47 | 4 | 4.071428571 |
| ssc-miR-140-3p | ssc-mir-140 | 3p | TACCACAGGGTAGAACCACGGAC | 591 | 512 | 1.151340996 |
| ssc-miR-142-5p | ssc-mir-142 | 5p | CATAAAGTAGAAAGCACTACT | 18 | 9 | 1.473684211 |
| ssc-miR-142-3p | ssc-mir-142 | 3p | TGTAGTGTTTCCTACTTTATGG | 22 |  | 3.2 |
| ssc-miR-143-5p | ssc-mir-143 | 5p | GGTGCAGTGCTGCATCTCTGG |  |  |  |
| ssc-miR-143-3p | ssc-mir-143 | 3p | TGAGATGAAGCACTGTAGCTC | 63 | 26 | 2.027777778 |
| ssc-miR-144 | ssc-mir-144 | 3p | TACAGTATAGATGATGTAC | 7 | 2 | 1.416666667 |
| ssc-miR-145-5p | ssc-mir-145 | 5p | GTCCAGTTTTCCCAGGAATCCCTT |  |  |  |
| ssc-miR-145-3p | ssc-mir-145 | 3p | GGATTCCTGGAAATACTGTTCT |  |  |  |
| ssc-miR-1468 | ssc-mir-1468 | 5p | CTCCGTTTGCCTGTTTTGCTGA |  |  |  |
| ssc-miR-146a-5p | ssc-mir-146a | 5p | TGAGAACTGAATTCCATGGGTT | 429 | 258 | 1.638059701 |
| ssc-miR-146a-3p | ssc-mir-146a | 3p | CCTGTGAAGTTTAGTTCTTCAG |  |  |  |
| ssc-miR-146b | ssc-mir-146b | 5p | TGAGAACTGAATTCCATAGGC | 868 | 887 | 0.978818283 |
| ssc-miR-148a-5p | ssc-mir-148a | 5p | AAAGTTCTGAGACACTCCGACT | 2 |  | 1.2 |
| ssc-miR-148a-3p | ssc-mir-148a | 3p | TCAGTGCACTACAGAACTTTGT | 31 | 11 | 1.952380952 |
| ssc-miR-148b-5p | ssc-mir-148b | 5p | GAAGTTCTGTTATACACTCAGGC | 11 | 32 | 0.5 |
| ssc-miR-148b-3p | ssc-mir-148b | 3p | TCAGTGCATCACAGAACTTTGT | 768 | 376 | 2.015544041 |
| ssc-miR-149 | ssc-mir-149 | 5p | TCTGGCTCCGTGTCTTCACTCCC |  | 11 | 0.476190476 |
| ssc-miR-150 | ssc-mir-150-1 | 5p | TCTCCCAACCCTTGTACCAGTG |  |  |  |
| ssc-miR-150 | ssc-mir-150-2 | 5p | TCTCCCAACCCTTGTACCAGTG |  |  |  |
| ssc-miR-151-5p | ssc-mir-151 | 5p | TCGAGGAGCTCACAGTCTAGT | 524 | 459 | 1.138592751 |
| ssc-miR-151-3p | ssc-mir-151 | 3p | CTAGACTGAAGCTCCTTGAGGA | 1262 | 1063 | 1.185461323 |
| ssc-miR-152 | ssc-mir-152 | 3p | TCAGTGCATGACAGAACTTGG | 345 | 247 | 1.381322957 |
| ssc-miR-153 | ssc-mir-153 | 3p | TTGCATAGTCACAAAAGTGA |  |  |  |
| ssc-miR-155-5p | ssc-mir-155 | 5p | TTAATGCTAATTGTGATAGGGG | 753 | 4372 | 0.174121406 |
| ssc-miR-155-3p | ssc-mir-155 | 3p | TCCTACATGTTAGCATTAACA |  |  |  |
| ssc-miR-15a | ssc-mir-15a | 5p | TAGCAGCACATAATGGTTTGT | 2455 | 42 | 47.40384615 |
| ssc-miR-15b | ssc-mir-15b | 5p | TAGCAGCACATCATGGTTTACA | 432 | 222 | 1.905172414 |
| ssc-miR-16 | ssc-mir-16-1 | 5p | TAGCAGCACGTAAATATTGGCG | 6340 | 13628 | 0.465610793 |
| ssc-miR-16 | ssc-mir-16-2 | 5p | TAGCAGCACGTAAATATTGGCG | 6340 | 13628 | 0.465610793 |
| ssc-miR-17-5p | ssc-mir-17 | 5p | CAAAGTGCTTACAGTGCAGGTAG | 932 | 5297 | 0.177501413 |
| ssc-miR-17-3p | ssc-mir-17 | 3p | ACTGCAGTGAAGGCACTTGTAG |  | 20 | 0.333333333 |
| ssc-miR-181a | ssc-mir-181a-1 | 5p | AACATTCAACGCTGTCGGTGAGTT | 917 | 278 | 3.21875 |
| ssc-miR-181a | ssc-mir-181a-2 | 5p | AACATTCAACGCTGTCGGTGAGTT | 917 | 278 | 3.21875 |
| ssc-miR-181b | ssc-mir-181b-1 | 5p | AACATTCATTGCTGTCGGTGGGTT | 254 | 91 | 2.613861386 |
| ssc-miR-181b | ssc-mir-181b-2 | 5p | AACATTCATTGCTGTCGGTGGGTT | 254 | 91 | 2.613861386 |
| ssc-miR-181c | ssc-mir-181c | 5p | AACATTCAACCTGTCGGTGAGT | 2 |  | 1.2 |
| ssc-miR-181d-5p | ssc-mir-181d | 5p | AACATTCATTGTTGTCGGTGGGTT | 5 | 3 | 1.153846154 |
| ssc-miR-181d-3p | ssc-mir-181d | 3p | CCCACCGAGGGATGAATGTCAC |  |  |  |
| ssc-miR-182 | ssc-mir-182 | 5p | TTTGGCAATGGTAGAACTCACACT | 23942 | 12259 | 1.952237346 |
| ssc-miR-183 | ssc-mir-183 | 5p | TATGGCACTGGTAGAATTCACTG | 249 | 196 | 1.257281553 |
| ssc-miR-1839-5p | ssc-mir-1839 | 5p | AAGGTAGATAGAACAGGTCTTG | 6 | 2 | 1.333333333 |
| ssc-miR-1839-3p | ssc-mir-1839 | 3p | AGACCTACTTTTCTACCAACA |  |  |  |
| ssc-miR-184 | ssc-mir-184 | 3p | TGGACGGAGAACTGATAAGGGT | 88 | 195 | 0.47804878 |
| ssc-miR-185 | ssc-mir-185 | 5p | TGGAGAGAAAGGCAGTTCCTGA | 21 | 12 | 1.409090909 |
| ssc-miR-186 | ssc-mir-186 | 5p | CAAAGAATTCTCCTTTTGGGCTT | 711 | 326 | 2.145833333 |
| ssc-miR-187 | ssc-mir-187 | 3p | TCGTGTCTTGTGTTGCAGCCGG |  |  |  |
| ssc-miR-18a | ssc-mir-18a | 5p | TAAGGTGCATCTAGTGCAGATA | 18 | 350 | 0.077777778 |
| ssc-miR-18b | ssc-mir-18b | 5p | TAAGGTGCATCTAGTGCAGTTAG |  | 2 | 0.833333333 |
| ssc-miR-190a | ssc-mir-190a | 5p | TGATATGTTTGATATATTAGG | 40 | 29 | 1.282051282 |
| ssc-miR-190b | ssc-mir-190b | 5p | TGATATGTTTGATATTGGGTTG | 19 | 23 | 0.878787879 |
| ssc-miR-191 | ssc-mir-191 | 5p | CAACGGAATCCCAAAAGCAGCTG | 3403 | 3729 | 0.912810912 |
| ssc-miR-192 | ssc-mir-192 | 5p | CTGACCTATGAATTGACAGCC | 14048 | 16353 | 0.859133411 |
| ssc-miR-193a-5p | ssc-mir-193a | 5p | TGGGTCTTTGCGGGCGAGATGA | 4 |  | 1.4 |
| ssc-miR-193a-3p | ssc-mir-193a | 3p | AACTGGCCTACAAAGTCCCAGT | 21 | 4 | 2.214285714 |
| ssc-miR-194a | ssc-mir-194a | 5p | TGTAACAGCAACTCCATGTGG | 27 | 13 | 1.608695652 |
| ssc-miR-194b-5p | ssc-mir-194b | 5p | TGTAACAGCGACTCCATGTGGA |  |  |  |
| ssc-miR-194b-3p | ssc-mir-194b | 3p | CCAGTGGAGATGCTGTTACCTT |  |  |  |
| ssc-miR-195 | ssc-mir-195 | 5p | TAGCAGCACAGAAATATTGGC | 7 | 9 | 0.894736842 |
| ssc-miR-196a | ssc-mir-196a-1 | 5p | TAGGTAGTTTCATGTTGTTGGG | 5 |  | 1.5 |
| ssc-miR-196a | ssc-mir-196a-2 | 5p | TAGGTAGTTTCATGTTGTTGGG | 5 |  | 1.5 |
| ssc-miR-196b-5p | ssc-mir-196b-1 | 5p | TAGGTAGTTTCCTGTTGTTGGG | 577 | 121 | 4.480916031 |
| ssc-miR-196b-3p | ssc-mir-196b-1 | 3p | CGACAGCACGACACTGCCTTCA |  |  |  |
| ssc-miR-196b | ssc-mir-196b-2 | 5p | TAGGTAGTTTCCTGTTGTTGGG | 577 | 121 | 4.480916031 |
| ssc-miR-199a-5p | ssc-mir-199a-1 | 5p | CCCAGTGTTCAGACTACCTGTTC |  |  |  |
| ssc-miR-199a-3p | ssc-mir-199a-1 | 3p | ACAGTAGTCTGCACATTGGTTA | 2 |  | 1.2 |
| ssc-miR-199a-5p | ssc-mir-199a-2 | 5p | CCCAGTGTTCAGACTACCTGTTC |  |  |  |
| ssc-miR-199a-3p | ssc-mir-199a-2 | 3p | ACAGTAGTCTGCACATTGGTTA | 2 |  | 1.2 |
| ssc-miR-199b-5p | ssc-mir-199b | 5p | CCCAGTGTTTAGACTATCTGTT |  | 3 | 0.769230769 |
| ssc-miR-199b-3p | ssc-mir-199b | 3p | TACAGTAGTCTGCACATTGGTT | 2 |  | 1.2 |
| ssc-miR-19a | ssc-mir-19a | 3p | TGTGCAAATCTATGCAAAACTGA | 142 | 203 | 0.713615023 |
| ssc-miR-19b | ssc-mir-19b-1 | 3p | TGTGCAAATCCATGCAAAACTGA | 303 | 365 | 0.834666667 |
| ssc-miR-19b | ssc-mir-19b-2 | 3p | TGTGCAAATCCATGCAAAACTGA | 303 | 365 | 0.834666667 |
| ssc-miR-202-5p | ssc-mir-202 | 5p | TTCCTATGCATATACTTCTTT |  |  |  |
| ssc-miR-202-3p | ssc-mir-202 | 3p | AGAGGTGTAGGCATGGGAA |  |  |  |
| ssc-miR-204 | ssc-mir-204 | 5p | TTCCCTTTGTCATCCTATGCCT | 2 |  | 1.2 |
| ssc-miR-205 | ssc-mir-205 | 5p | TCCTTCATTCCACCGGAGTCTG |  |  |  |
| ssc-miR-206 | ssc-mir-206 | 3p | TGGAATGTAAGGAAGTGTGTGA |  | 2 | 0.833333333 |
| ssc-miR-208b | ssc-mir-208b | 3p | ATAAGACGAACAAAAGGTTTGT |  |  |  |
| ssc-miR-20a | ssc-mir-20a | 5p | TAAAGTGCTTATAGTGCAGGTA | 748 | 2946 | 0.256427605 |
| ssc-miR-20b | ssc-mir-20b-1 | 5p | CAAAGTGCTCACAGTGCAGGTAG |  |  |  |
| ssc-miR-20b | ssc-mir-20b-2 | 5p | CAAAGTGCTCACAGTGCAGGTAG |  |  |  |
| ssc-miR-21 | ssc-mir-21 | 5p | TAGCTTATCAGACTGATGTTGA | 60378 | 38972 | 1.549125237 |
| ssc-miR-210 | ssc-mir-210 | 3p | CTGTGCGTGTGACAGCGGCTGA | 107 | 304 | 0.372611465 |
| ssc-miR-212 | ssc-mir-212 | 5p | ACCTTGGCTCTAGACTGCTTACT | 5 |  | 1.5 |
| ssc-miR-214 | ssc-mir-214 | 3p | ACAGCAGGCACAGACAGGCAG |  |  |  |
| ssc-miR-215 | ssc-mir-215 | 5p | ATGACCTATGAATTGACAGAC |  |  |  |
| ssc-miR-216 | ssc-mir-216-1 | 5p | TAATCTCAGCTGGCAACTGTGAG |  |  |  |
| ssc-miR-216 | ssc-mir-216-2 | 5p | TAATCTCAGCTGGCAACTGTGAG |  |  |  |
| ssc-miR-217 | ssc-mir-217-1 | 5p | TACTGCATCAGGAACTGATTGGAT |  |  |  |
| ssc-miR-217 | ssc-mir-217-2 | 5p | TACTGCATCAGGAACTGATTGGAT |  |  |  |
| ssc-miR-218-5p | ssc-mir-218-1 | 5p | TTGTGCTTGATCTAACCATGT |  | 2 | 0.833333333 |
| ssc-miR-218-3p | ssc-mir-218-1 | 3p | ATGGTTCTGTCAAGCACCATG |  |  |  |
| ssc-miR-218 | ssc-mir-218-2 | 5p | TTGTGCTTGATCTAACCATGT |  | 2 | 0.833333333 |
| ssc-miR-218b | ssc-mir-218b | 5p | TTGTGCTTGATCTAACCATGTG |  | 2 | 0.833333333 |
| ssc-miR-219 | ssc-mir-219 | 3p | AGAGTTGAGTCTGGACGTCCCG | 8 |  | 1.8 |
| ssc-miR-22-5p | ssc-mir-22 | 5p | AGTTCTTCAGTGGCAAGCTTTA | 8 | 3 | 1.384615385 |
| ssc-miR-22-3p | ssc-mir-22 | 3p | AAGCTGCCAGTTGAAGAACTGT | 964 | 1102 | 0.875899281 |
| ssc-miR-221-5p | ssc-mir-221 | 5p | ACCTGGCATACAATGTAGATTTCTGT |  | 2 | 0.833333333 |
| ssc-miR-221-3p | ssc-mir-221 | 3p | AGCTACATTGTCTGCTGGGTTT | 2545 | 845 | 2.988304094 |
| ssc-miR-222 | ssc-mir-222 | 3p | AGCTACATCTGGCTACTGGGTCTC | 283 | 113 | 2.382113821 |
| ssc-miR-224 | ssc-mir-224 | 5p | CAAGTCACTAGTGGTTCCGTTTA | 4 | 3 | 1.076923077 |
| ssc-miR-2320-5p | ssc-mir-2320 | 5p | TGGCACAGGGTCCAGCTGTCGG |  |  |  |
| ssc-miR-2320-3p | ssc-mir-2320 | 3p | CGATGATGGTCCCTGTGTTTG | 33 | 39 | 0.87755102 |
| ssc-miR-2366 | ssc-mir-2366-1 | 3p | TGGGTCACAGAAGAGGGTCTGG |  |  |  |
| ssc-miR-2366 | ssc-mir-2366-2 | 3p | TGGGTCACAGAAGAGGGTCTGG |  |  |  |
| ssc-miR-23a | ssc-mir-23a | 3p | ATCACATTGCCAGGGATTTCC | 222 | 140 | 1.546666667 |
| ssc-miR-23b | ssc-mir-23b | 3p | ATCACATTGCCAGGGATTACCA | 74 | 67 | 1.090909091 |
| ssc-miR-24-1-5p | ssc-mir-24-1 | 5p | GTGCCTACTGAGCTGAAACACAGT | 21 | 11 | 1.476190476 |
| ssc-miR-24-3p | ssc-mir-24-1 | 3p | TGGCTCAGTTCAGCAGGAACAG | 212 | 61 | 3.126760563 |
| ssc-miR-24-2-5p | ssc-mir-24-2 | 5p | GTGCCTACTGAGCTGATATCAGT |  |  |  |
| ssc-miR-24-3p | ssc-mir-24-2 | 3p | TGGCTCAGTTCAGCAGGAACAG | 212 | 61 | 3.126760563 |
| ssc-miR-2411 | ssc-mir-2411 | 5p | TGGAGTGACTGTCAGATGCAGC | 48 | 22 | 1.8125 |
| ssc-miR-2483 | ssc-mir-2483 | 3p | AAACATCTGGTTGGTTGAGAGA | 3 | 2 | 1.083333333 |
| ssc-miR-26a | ssc-mir-26a | 5p | TTCAAGTAATCCAGGATAGGCT | 5371 | 14568 | 0.369117849 |
| ssc-miR-27a | ssc-mir-27a | 3p | TTCACAGTGGCTAAGTTCCGC | 5054 | 5247 | 0.963287046 |
| ssc-miR-27b-5p | ssc-mir-27b | 5p | AGAGCTTAGCTGATTGGTGAACA | 3 | 4 | 0.928571429 |
| ssc-miR-27b-3p | ssc-mir-27b | 3p | TTCACAGTGGCTAAGTTCTGC | 6994 | 6403 | 1.092156557 |
| ssc-miR-28-5p | ssc-mir-28 | 5p | AAGGAGCTCACAGTCTATTGAG | 13 | 7 | 1.352941176 |
| ssc-miR-28-3p | ssc-mir-28 | 3p | CACTAGATTGTGAGCTCCTGGA | 752 | 815 | 0.923636364 |
| ssc-miR-296-5p | ssc-mir-296 | 5p | GAGGGCCCCCCCCAATCCTGT | 7 |  | 1.7 |
| ssc-miR-296-3p | ssc-mir-296 | 3p | AGGGTTGGGCGGAGGCTTTCC | 126 | 49 | 2.305084746 |
| ssc-miR-299 | ssc-mir-299 | 5p | ATGGTTTACCGTCCCACATAC |  |  |  |
| ssc-miR-29a | ssc-mir-29a | 3p | CTAGCACCATCTGAAATCGGTTA | 81 | 89 | 0.919191919 |
| ssc-miR-29b | ssc-mir-29b-1 | 3p | TAGCACCATTTGAAATCAGTGTT | 45 | 55 | 0.846153846 |
| ssc-miR-29b | ssc-mir-29b-2 | 3p | TAGCACCATTTGAAATCAGTGTT | 45 | 55 | 0.846153846 |
| ssc-miR-29c | ssc-mir-29c | 3p | TAGCACCATTTGAAATCGGTTA | 4 | 11 | 0.666666667 |
| ssc-miR-301 | ssc-mir-301 | 3p | CAGTCCAATAGTATTGTCAAAGC |  |  |  |
| ssc-miR-30a-5p | ssc-mir-30a | 5p | TGTAAACATCCTCGACTGGAAG | 172369 | 103201 | 1.670161126 |
| ssc-miR-30a-3p | ssc-mir-30a | 3p | CTTTCAGTCGGATGTTTGCAGC | 620 | 145 | 4.064516129 |
| ssc-miR-30b-5p | ssc-mir-30b | 5p | TGTAAACATCCTACACTCAGCT | 1405 | 469 | 2.954070981 |
| ssc-miR-30b-3p | ssc-mir-30b | 3p | CTGGGAGGTGGATGTTTACTT | 9 |  | 1.9 |
| ssc-miR-30c-5p | ssc-mir-30c-1 | 5p | TGTAAACATCCTACACTCTCAGC | 3158 | 985 | 3.183919598 |
| ssc-miR-30c-1-3p | ssc-mir-30c-1 | 3p | CTGGGAGAGGGTTGTTTACT |  |  |  |
| ssc-miR-30c-5p | ssc-mir-30c-2 | 5p | TGTAAACATCCTACACTCTCAGC | 3158 | 985 | 3.183919598 |
| ssc-miR-30c-3p | ssc-mir-30c-2 | 3p | CTGGGAGAAGGCTGTTTACTCT | 15 | 28 | 0.657894737 |
| ssc-miR-30d | ssc-mir-30d | 5p | TGTAAACATCCCCGACTGGAAGCT | 11943 | 10678 | 1.118357036 |
| ssc-miR-30e-5p | ssc-mir-30e | 5p | TGTAAACATCCTTGACTGGAAGCT | 2510 | 1795 | 1.396121884 |
| ssc-miR-30e-3p | ssc-mir-30e | 3p | CTTTCAGTCGGATGTTTACAGC | 152 | 59 | 2.347826087 |
| ssc-miR-31 | ssc-mir-31 | 5p | AGGCAAGATGCTGGCATAGCTG | 51 | 32 | 1.452380952 |
| ssc-miR-32 | ssc-mir-32 | 5p | TATTGCACATTACTAAGTTGC | 2 |  | 1.2 |
| ssc-miR-320 | ssc-mir-320 | 3p | AAAAGCTGGGTTGAGAGGGCGAA | 2942 | 509 | 5.687861272 |
| ssc-miR-323 | ssc-mir-323 | 3p | GCACATTACACGGTCGACCTCT | 5 | 4 | 1.071428571 |
| ssc-miR-324 | ssc-mir-324 | 5p | CGCATCCCCTAGGGCATTGGTGT | 3 | 3 | 1 |
| ssc-miR-325 | ssc-mir-325 | 5p | CCTAGTAGGTGTTCAGTAAGTGT |  |  |  |
| ssc-miR-326 | ssc-mir-326 | 3p | CCTCTGGGCCCTTCCTCCAG |  |  |  |
| ssc-miR-328 | ssc-mir-328 | 3p | CTGGCCCTCTCTGCCCTTCCGT | 2 |  | 1.2 |
| ssc-miR-331-5p | ssc-mir-331 | 5p | TCTAGGTATGGTCCCAGGGAT | 7 | 3 | 1.307692308 |
| ssc-miR-331-3p | ssc-mir-331 | 3p | GCCCCTGGGCCTATCCTAGAA | 4 | 2 | 1.166666667 |
| ssc-miR-335 | ssc-mir-335 | 5p | TCAAGAGCAATAACGAAAAATG | 45 | 45 | 1 |
| ssc-miR-338 | ssc-mir-338 | 3p | TCCAGCATCAGTGATTTTGTTG |  | 2 | 0.833333333 |
| ssc-miR-339-5p | ssc-mir-339-1 | 5p | TCCCTGTCCTCCAGGAGCTCAC | 185 | 85 | 2.052631579 |
| ssc-miR-339-3p | ssc-mir-339-1 | 3p | AGCTCCTCGAGGCCAGAGCCC |  |  |  |
| ssc-miR-339 | ssc-mir-339-2 | 5p | TCCCTGTCCTCCAGGAGCTCA | 185 | 85 | 2.052631579 |
| ssc-miR-340 | ssc-mir-340-1 | 5p | TTATAAAGCAATGAGACTGATT | 664 | 463 | 1.424947146 |
| ssc-miR-340 | ssc-mir-340-2 | 5p | TTATAAAGCAATGAGACTGATT | 664 | 463 | 1.424947146 |
| ssc-miR-342 | ssc-mir-342 | 3p | TCTCACACAGAAATCGCACCCGTCA | 5 | 4 | 1.071428571 |
| ssc-miR-345-5p | ssc-mir-345-1 | 5p | GCTGACTCCTAGTCCAGTGC | 2 | 2 | 1 |
| ssc-miR-345-3p | ssc-mir-345-1 | 3p | CCCTGAACTAGGGGTCTGGAG | 4 |  | 1.4 |
| ssc-miR-345-5p | ssc-mir-345-2 | 5p | GCTGACTCCTAGTCCAGTGC | 2 | 2 | 1 |
| ssc-miR-345-3p | ssc-mir-345-2 | 3p | CCCTGAACTAGGGGTCTGGAG | 4 |  | 1.4 |
| ssc-miR-34a | ssc-mir-34a | 5p | TGGCAGTGTCTTAGCTGGTTGT | 584 | 892 | 0.658536585 |
| ssc-miR-34c | ssc-mir-34c-1 | 5p | AGGCAGTGTAGTTAGCTGATTGC | 3 | 7 | 0.764705882 |
| ssc-miR-34c | ssc-mir-34c-2 | 5p | AGGCAGTGTAGTTAGCTGATTGC | 3 | 7 | 0.764705882 |
| ssc-miR-361-5p | ssc-mir-361 | 5p | TTATCAGAATCTCCAGGGGTAC | 12 | 9 | 1.157894737 |
| ssc-miR-361-3p | ssc-mir-361 | 3p | CCCCCAGGTGTGATTCTGATTTGC | 14 | 105 | 0.208695652 |
| ssc-miR-3613 | ssc-mir-3613 | 5p | TGTTGTACTTTTTTTTTTGT | 47 | 20 | 1.9 |
| ssc-miR-362 | ssc-mir-362 | 5p | AATCCTTGGAACCTAGGTGTGAGTG | 18 | 10 | 1.4 |
| ssc-miR-363 | ssc-mir-363-1 | 3p | AATTGCACGGTATCCATCTGTAA |  |  |  |
| ssc-miR-363 | ssc-mir-363-2 | 3p | AATTGCACGGTATCCATCTGTAA |  |  |  |
| ssc-miR-365-3p | ssc-mir-365-1 | 3p | TAATGCCCCTAAAAATCCTTAT | 39 | 16 | 1.884615385 |
| ssc-miR-365-5p | ssc-mir-365-2 | 5p | GAGGGACTTTCAGGGGCAGCTGT | 4 | 3 | 1.076923077 |
| ssc-miR-365-3p | ssc-mir-365-2 | 3p | TAATGCCCCTAAAAATCCTTAT | 39 | 16 | 1.884615385 |
| ssc-miR-369 | ssc-mir-369 | 3p | AATAATACATGGTTGATCTTT |  |  |  |
| ssc-miR-370 | ssc-mir-370 | 3p | GCCTGCTGGGGTGGAACCTGGT |  |  |  |
| ssc-miR-374a-5p | ssc-mir-374a | 5p | TTATAATACAACCTGATAAGTG | 26 | 23 | 1.090909091 |
| ssc-miR-374a-3p | ssc-mir-374a | 3p | CTTATCAGGTTGTATTGTAATT | 220 | 207 | 1.059907834 |
| ssc-miR-374b-5p | ssc-mir-374b | 5p | ATATAATACAACCTGCTAAGTG | 28 | 12 | 1.727272727 |
| ssc-miR-374b-3p | ssc-mir-374b | 3p | CTTATCAGGTTGTATTATCATT | 12 | 8 | 1.222222222 |
| ssc-miR-376a-5p | ssc-mir-376a | 5p | GTAGATTCTCCTTCTATGAGTAC |  |  |  |
| ssc-miR-376a-3p | ssc-mir-376a | 3p | ATCATAGAGGAAAATCCACGT |  |  |  |
| ssc-miR-376b | ssc-mir-376b | 5p | GTGGCTATTCCTTCTATGTTTA |  |  |  |
| ssc-miR-376c | ssc-mir-376c | 5p | GTGGATATTCCTTCTATGTTTA |  |  |  |
| ssc-miR-378 | ssc-mir-378-1 | 3p | ACTGGACTTGGAGTCAGAAGGC | 4001 | 2110 | 1.891981132 |
| ssc-miR-378 | ssc-mir-378-2 | 3p | ACTGGACTTGGAGTCAGAAGGC | 4001 | 2110 | 1.891981132 |
| ssc-miR-381 | ssc-mir-381 | 5p | AGCGAGGTTGCCCTTTGTATATT |  |  |  |
| ssc-miR-382 | ssc-mir-382 | 5p | AAGTTGTTCGTGGTGGATTCG | 11 | 8 | 1.166666667 |
| ssc-miR-383 | ssc-mir-383 | 3p | CCACAGCACTGCCTGGTCAGA | 2 |  | 1.2 |
| ssc-miR-411 | ssc-mir-411 | 3p | ATGTAACACGGTCCACTAAC | 5 | 10 | 0.75 |
| ssc-miR-421-5p | ssc-mir-421 | 5p | CCTCATTAAATGTTTGTTGAATGA |  |  |  |
| ssc-miR-421-3p | ssc-mir-421 | 3p | ATCAACAGACATTAATTGGGCGC | 30 | 21 | 1.290322581 |
| ssc-miR-423-5p | ssc-mir-423 | 5p | TGAGGGGCAGAGAGCGAGACTTT | 594 | 226 | 2.559322034 |
| ssc-miR-423-3p | ssc-mir-423 | 3p | AGCTCGGTCTGAGGCCCCTCAGT | 363 | 248 | 1.445736434 |
| ssc-miR-424-5p | ssc-mir-424 | 5p | CAGCAGCAATTCATGTTTTGAA | 57 | 10 | 3.35 |
| ssc-miR-424-3p | ssc-mir-424 | 3p | CAAAACGTGAGGCGCTGCTAT | 6 | 42 | 0.307692308 |
| ssc-miR-425-5p | ssc-mir-425 | 5p | AATGACACGATCACTCCCGTTGA | 70 | 90 | 0.8 |
| ssc-miR-425-3p | ssc-mir-425 | 3p | ATCGGGAATGTCGTGTCCGCCC | 62 | 47 | 1.263157895 |
| ssc-miR-429 | ssc-mir-429 | 3p | TAATACTGTCTGGTAATGCCGT | 102 | 44 | 2.074074074 |
| ssc-miR-432-5p | ssc-mir-432 | 5p | TCTTGGAGTAGGTCATTGGGT | 3 |  | 1.3 |
| ssc-miR-432-3p | ssc-mir-432 | 3p | TGGATGGCTCCTCCATGGCT |  |  |  |
| ssc-miR-4331 | ssc-mir-4331 | 3p | TGTGGCTGTGGTGTAGGCCAGC | 29 | 72 | 0.475609756 |
| ssc-miR-4332 | ssc-mir-4332 | 3p | CACGGCCGCCGCCGGGCGCC | 12 |  | 2.2 |
| ssc-miR-4333 | ssc-mir-4333 | 5p | ATACCTGCATGTTAGTCTTTGGTTCT |  |  |  |
| ssc-miR-4334-5p | ssc-mir-4334 | 5p | CCCTGGAGTGACGGGGGTG |  |  |  |
| ssc-miR-4334-3p | ssc-mir-4334 | 3p | TCCCTGTCCTCCAGGAGCTC | 185 | 85 | 2.052631579 |
| ssc-miR-4335 | ssc-mir-4335 | 3p | GTGCCCAGCGCTGCAGGGCA |  |  |  |
| ssc-miR-4336 | ssc-mir-4336 | 5p | CAACTCTGTGGTTTCCTTTACTCATAG |  |  |  |
| ssc-miR-4337 | ssc-mir-4337 | 5p | AGGGTATATAAGCCTTCACTGG |  |  |  |
| ssc-miR-4338 | ssc-mir-4338 | 5p | ATGTTCAGTCTCAGTGGGAACC |  |  |  |
| ssc-miR-4339 | ssc-mir-4339 | 5p | GCTCTGAGCTGCCCCTCCTCGTCC |  |  |  |
| ssc-miR-450a | ssc-mir-450a | 5p | TTTTGCGATGTGTTCCTAATAT | 13 | 16 | 0.884615385 |
| ssc-miR-450b-5p | ssc-mir-450b | 5p | TTTTGCAATATGTTCCTGAATA | 14 | 7 | 1.411764706 |
| ssc-miR-450b-3p | ssc-mir-450b | 3p | TTGGGAACATTTTGCATCCAT |  | 2 | 0.833333333 |
| ssc-miR-450c-5p | ssc-mir-450c | 5p | TTTTGCGATGTGTTCCTAATAC | 123 | 164 | 0.764367816 |
| ssc-miR-450c-3p | ssc-mir-450c | 3p | ATTGGGAACATTTTGCATTCGT | 3 | 2 | 1.083333333 |
| ssc-miR-451 | ssc-mir-451 | 5p | AAACCGTTACCATTACTGAGTT | 23 | 8 | 1.833333333 |
| ssc-miR-452 | ssc-mir-452 | 5p | AACTGTTTGCAGAGGAAACTGA |  |  |  |
| ssc-miR-455-5p | ssc-mir-455 | 5p | TATGTGCCTTTGGACTACATCG | 21 | 35 | 0.688888889 |
| ssc-miR-455-3p | ssc-mir-455 | 3p | GCAGTCCATGGGCATATACAC | 6 | 10 | 0.8 |
| ssc-miR-484 | ssc-mir-484 | 3p | CCCAGGGGGCGACCCAGGCT |  |  |  |
| ssc-miR-486 | ssc-mir-486-1 | 3p | TCCTGTACTGAGCTGCCCCGAG | 28 | 142 | 0.25 |
| ssc-miR-486 | ssc-mir-486-2 | 5p | TCCTGTACTGAGCTGCCCCGAG | 28 | 142 | 0.25 |
| ssc-miR-487b | ssc-mir-487b | 5p | GTGGTTATCCCTGTCCTGTTCG |  |  |  |
| ssc-miR-489 | ssc-mir-489 | 3p | AGTGACATCACATATACGGCGG | 114 | 16 | 4.769230769 |
| ssc-miR-490-5p | ssc-mir-490-1 | 5p | CCATGGATCCCCAGGTGGGT | 227 |  | 23.7 |
| ssc-miR-490-3p | ssc-mir-490-1 | 3p | CAACCTGGAGGACTCCATGCTG | 11 | 2 | 1.75 |
| ssc-miR-490 | ssc-mir-490-2 | 3p | CAACCTGGAGGACTCCATGCTG | 11 | 2 | 1.75 |
| ssc-miR-491 | ssc-mir-491 | 5p | AGTGGGGAACCCTTCCATGAGG |  |  |  |
| ssc-miR-493-5p | ssc-mir-493 | 5p | TTGTACATGGTAGGCTTTCATT |  |  |  |
| ssc-miR-493-3p | ssc-mir-493 | 3p | TGAAGGTCTACTGTGTGCCAGG |  |  |  |
| ssc-miR-494 | ssc-mir-494 | 5p | AGGTTGTCGTGTTGTCTTCTCT |  |  |  |
| ssc-miR-497 | ssc-mir-497 | 5p | CAGCAGCACACTGTGGTTTGT | 9 | 11 | 0.904761905 |
| ssc-miR-499-5p | ssc-mir-499 | 5p | TTAAGACTTGCAGTGATGTTT |  | 4 | 0.714285714 |
| ssc-miR-499-3p | ssc-mir-499 | 3p | AACATCACAGCAAGTCTGTGCT |  |  |  |
| ssc-miR-500 | ssc-mir-500 | 3p | ATGCACCTGGGCAAGGATTCT | 89 | 29 | 2.538461538 |
| ssc-miR-503 | ssc-mir-503 | 5p | TAGCAGCGGGAACAGTACTGCAG | 33 | 11 | 2.047619048 |
| ssc-miR-504 | ssc-mir-504 | 5p | AGACCCTGGTCTGCACTCTATCT |  |  |  |
| ssc-miR-505 | ssc-mir-505 | 3p | TCAACACTTGCTGGTTTCCTCT | 180 | 37 | 4.042553191 |
| ssc-miR-532-5p | ssc-mir-532 | 5p | CATGCCTTGAGTGTAGGACCGT | 600 | 324 | 1.826347305 |
| ssc-miR-532-3p | ssc-mir-532 | 3p | CCTCCCACACCCAAGGCTTGCA | 8 | 4 | 1.285714286 |
| ssc-miR-542-5p | ssc-mir-542 | 5p | TCGGGGATCATCATGTCACGA | 9 |  | 1.9 |
| ssc-miR-542-3p | ssc-mir-542 | 3p | TGTGACAGATTGATAACTGAAA | 69 | 79 | 0.887640449 |
| ssc-miR-545-5p | ssc-mir-545 | 5p | TCAGTAAATGTTTATTGGATG | 43 | 13 | 2.304347826 |
| ssc-miR-545-3p | ssc-mir-545 | 3p | ATCAACAAACATTTATTGTGTG | 11 |  | 2.1 |
| ssc-miR-551a | ssc-mir-551a | 3p | GCGACCCACTCTTGGTTTCC | 2 |  | 1.2 |
| ssc-miR-574 | ssc-mir-574 | 3p | CACGCTCATGCACACACCCACA |  | 9 | 0.526315789 |
| ssc-miR-582 | ssc-mir-582 | 3p | TAACCGGTTGAACAACTGAACC | 7 | 7 | 1 |
| ssc-miR-615 | ssc-mir-615 | 3p | TCCGAGCCTGGGTCTCCCTCT |  |  |  |
| ssc-miR-628 | ssc-mir-628 | 5p | ATGCTGACATATTTACTAGAGG |  |  |  |
| ssc-miR-652 | ssc-mir-652 | 5p | ACAACCCTAGGAGAGGGTGCCATTCA |  |  |  |
| ssc-miR-664-5p | ssc-mir-664 | 5p | CAGGCTAGGAGAAGTGATTGGAT | 29 | 33 | 0.906976744 |
| ssc-miR-664-3p | ssc-mir-664 | 3p | TATTCATTTATCTCCCAGCCTACA |  |  |  |
| ssc-miR-671-5p | ssc-mir-671 | 5p | AGGAAGCCCTGGAGGGGCTGGAGG |  |  |  |
| ssc-miR-671-3p | ssc-mir-671 | 3p | TCCGGTTCTCAGGGCTCCACC |  |  |  |
| ssc-miR-676-5p | ssc-mir-676-1 | 5p | CTCTTCAATCTCAGGACTCGCA |  |  |  |
| ssc-miR-676-3p | ssc-mir-676-1 | 3p | CCGTCCTAAGGTTGTTGAGTT |  |  |  |
| ssc-miR-676-3p | ssc-mir-676-2 | 3p | CCGTCCTAAGGTTGTTGAGTT |  |  |  |
| ssc-miR-7 | ssc-mir-7-1 | 5p | TGGAAGACTAGTGATTTTGTTGTT | 4286 | 4565 | 0.939016393 |
| ssc-miR-7 | ssc-mir-7-2 | 5p | TGGAAGACTAGTGATTTTGTTGTT | 4286 | 4565 | 0.939016393 |
| ssc-miR-708-5p | ssc-mir-708 | 5p | AAGGAGCTTACAATCTAGCTGGG |  |  |  |
| ssc-miR-708-3p | ssc-mir-708 | 3p | CAACTAGACTGTGAGCTTCTAGA | 2 |  | 1.2 |
| ssc-miR-744 | ssc-mir-744 | 5p | TGCGGGGCTAGGGCTAACAGCA | 119 | 58 | 1.897058824 |
| ssc-miR-758 | ssc-mir-758 | 3p | TTTGTGACCTGGTCCACTAAC |  |  |  |
| ssc-miR-769-5p | ssc-mir-769 | 5p | TGAGACCTCTGGGTTCTGAGC | 316 | 223 | 1.399141631 |
| ssc-miR-769-3p | ssc-mir-769 | 3p | CTGGGATCTCTGGGGTCTTGGTT | 16 | 4 | 1.857142857 |
| ssc-miR-874 | ssc-mir-874 | 3p | CTGCCCTGGCCCGAGGGACCGAC |  |  |  |
| ssc-miR-885-5p | ssc-mir-885 | 5p | TCCATTACACTACCCTGCCTCT | 4 |  | 1.4 |
| ssc-miR-885-3p | ssc-mir-885 | 3p | AGGCAGCGGGGTGTAGTGGAT | 9 | 6 | 1.1875 |
| ssc-miR-9-1 | ssc-mir-9-1 | 5p | TCTTTGGTTATCTAGCTGTATGA | 91 | 49 | 1.711864407 |
| ssc-miR-9-2 | ssc-mir-9-2 | 5p | TCTTTGGTTATCTAGCTGTATGA | 91 | 49 | 1.711864407 |
| ssc-miR-9 | ssc-mir-9-3 | 5p | TCTTTGGTTATCTAGCTGTATG | 91 | 49 | 1.711864407 |
| ssc-miR-92a | ssc-mir-92a-1 | 3p | TATTGCACTTGTCCCGGCCTGT | 4317 | 3205 | 1.345878694 |
| ssc-miR-92a | ssc-mir-92a-2 | 3p | TATTGCACTTGTCCCGGCCTGT | 4317 | 3205 | 1.345878694 |
| ssc-miR-92b-5p | ssc-mir-92b | 5p | AGGGACGGGACGCGGTGCAGTGTT | 16 | 6 | 1.625 |
| ssc-miR-92b-3p | ssc-mir-92b | 3p | TATTGCACTCGTCCCGGCCTCC | 1644 | 1680 | 0.978698225 |
| ssc-miR-935 | ssc-mir-935 | 3p | CCAGTTACCGCTTCCGCTACCGC |  |  |  |
| ssc-miR-95 | ssc-mir-95 | 3p | TTCAACGGGTATTTATTGAGCA | 2 |  | 1.2 |
| ssc-miR-98 | ssc-mir-98 | 5p | TGAGGTAGTAAGTTGTATTGTT | 6455 | 4415 | 1.461016949 |
| ssc-miR-99a | ssc-mir-99a | 5p | AACCCGTAGATCCGATCTTGTG | 24 | 1262 | 0.02672956 |
| ssc-miR-99b | ssc-mir-99b | 5p | CACCCGTAGAACCGACCTTGCG | 58 | 45 | 1.236363636 |
| ssc-miR-novel-chr10_5263 | ssc-mir-novel-chr10_5263 | 3p | CACATGGAGTCGCTGTTACAGCT |  |  |  |
| ssc-miR-novel-chr10_5287 | ssc-mir-novel-chr10_5287 | 3p | ACTAGTGCTGTCAGAGACGCC |  |  |  |
| ssc-miR-novel-chr10_5425 | ssc-mir-novel-chr10_5425 | 3p | TACGAATTTCAGGAATACAGC |  |  |  |
| ssc-miR-novel-chr10_5436 | ssc-mir-novel-chr10_5436 | 5p | GAGCTGTGATGAGAATCCTCTGAGC |  |  |  |
| ssc-miR-novel-chr10_5472 | ssc-mir-novel-chr10_5472 | 5p | ACGAGAAAGGAGGAGGG |  |  |  |
| ssc-miR-novel-chr10_5540 | ssc-mir-novel-chr10_5540 | 5p | GCAGGAACTTGTGAGTCTCCT |  |  |  |
| ssc-miR-novel-chr10_5541 | ssc-mir-novel-chr10_5541 | 3p | TGGTGGTTTACAAAGTAATTC |  |  |  |
| ssc-miR-novel-chr10_5602 | ssc-mir-novel-chr10_5602 | 5p | TGAGTGTGTGTGTGTGAGTGTGTGT | 27 | 63 | 0.506849315 |
| ssc-miR-novel-chr10_5614 | ssc-mir-novel-chr10_5614 | 5p | GCGGGCCCACGGGGGCC | 3 |  | 1.3 |
| ssc-miR-novel-chr10_5682 | ssc-mir-novel-chr10_5682 | 3p | CCGGCCGGGCGCGAGCC | 10 | 13 | 0.869565217 |
| ssc-miR-novel-chr10_5914 | ssc-mir-novel-chr10_5914 | 3p | CACATGGAGTCGCTGTTACAGCT |  |  |  |
| ssc-miR-novel-chr10_5917 | ssc-mir-novel-chr10_5917 | 5p | TTCAGGGTCCAGGATTGCTATAG | 2 |  | 1.2 |
| ssc-miR-novel-chr10_6036 | ssc-mir-novel-chr10_6036 | 3p | TACGAATTTCAGGAATACAGC |  |  |  |
| ssc-miR-novel-chr10_6047 | ssc-mir-novel-chr10_6047 | 5p | TGGATGGGAGTCGGTGGGCAGC |  |  |  |
| ssc-miR-novel-chr10_6071 | ssc-mir-novel-chr10_6071 | 5p | GTGGATTTTTGGAGTTGGG |  |  |  |
| ssc-miR-novel-chr10_6138 | ssc-mir-novel-chr10_6138 | 3p | TGGTGGTTTACAAAGTAATTC |  |  |  |
| ssc-miR-novel-chr10_6327 | ssc-mir-novel-chr10_6327 | 5p | AGTGTGTGGGCGCCGGACGCT |  |  |  |
| ssc-miR-novel-chr11_6393 | ssc-mir-novel-chr11_6393 | 3p | CGGGGCCGGGGGTGGGG |  |  |  |
| ssc-miR-novel-chr11_6456 | ssc-mir-novel-chr11_6456 | 5p | CTCGCGGGAAAACTTGTATGTG |  |  |  |
| ssc-miR-novel-chr11_6577 | ssc-mir-novel-chr11_6577 | 3p | CTGAATGGAATTGTCTCAGCCT | 30 |  | 4 |
| ssc-miR-novel-chr11_6750 | ssc-mir-novel-chr11_6750 | 5p | CAGGGTCGGGCCTGGTTA | 16 | 2 | 2.166666667 |
| ssc-miR-novel-chr11_6755 | ssc-mir-novel-chr11_6755 | 3p | TGTCCCACCAGAGTCGCCA |  |  |  |
| ssc-miR-novel-chr11_6826 | ssc-mir-novel-chr11_6826 | 5p | ACTTTCCCGGGATTTGGAGCG | 3 |  | 1.3 |
| ssc-miR-novel-chr11_6850 | ssc-mir-novel-chr11_6850 | 5p | TCCTGGAGGACGTGCTGTGC | 4 | 2 | 1.166666667 |
| ssc-miR-novel-chr11_6942 | ssc-mir-novel-chr11_6942 | 3p | TGACTCACTCTGTTGTGCAGC |  |  |  |
| ssc-miR-novel-chr11_7050 | ssc-mir-novel-chr11_7050 | 3p | AGCGTGGGCTGCGGGCCGCT |  |  |  |
| ssc-miR-novel-chr11_7060 | ssc-mir-novel-chr11_7060 | 3p | CGCGCTTAGGGTTCCCGGCAT |  |  |  |
| ssc-miR-novel-chr11_7116 | ssc-mir-novel-chr11_7116 | 3p | CTGAATGGAATTGTCTCAGCCT | 30 |  | 4 |
| ssc-miR-novel-chr11_7251 | ssc-mir-novel-chr11_7251 | 5p | ACATTTAAGGAGGTGCTTGCT | 4 |  | 1.4 |
| ssc-miR-novel-chr12_7407 | ssc-mir-novel-chr12_7407 | 3p | CGGCGGCGGCGGCGACT | 4 | 23 | 0.424242424 |
| ssc-miR-novel-chr12_7455 | ssc-mir-novel-chr12_7455 | 3p | TGCTTGGACAGTGCCTGGCCTGC |  |  |  |
| ssc-miR-novel-chr12_7490 | ssc-mir-novel-chr12_7490 | 5p | TTTGCAGTAACAGGTGTGAAC |  |  |  |
| ssc-miR-novel-chr12_7511 | ssc-mir-novel-chr12_7511 | 3p | CTTTGTCTTAATCTCTGTGGTT |  |  |  |
| ssc-miR-novel-chr12_7711 | ssc-mir-novel-chr12_7711 | 3p | TGCGTCTCACCTGCCTGACAGG |  |  |  |
| ssc-miR-novel-chr12_7741 | ssc-mir-novel-chr12_7741 | 3p | GGAGCGGGCGGGCGGTC | 4 | 11 | 0.666666667 |
| ssc-miR-novel-chr12_7775 | ssc-mir-novel-chr12_7775 | 3p | TGAAAGGACGTAAAACAGGCCC |  |  |  |
| ssc-miR-novel-chr12_7779 | ssc-mir-novel-chr12_7779 | 3p | TGAAAGGACGTAAAACAGGCCC |  |  |  |
| ssc-miR-novel-chr12_7811 | ssc-mir-novel-chr12_7811 | 3p | TGCGTCTCACCTGCCTGACAGG |  |  |  |
| ssc-miR-novel-chr12_7898 | ssc-mir-novel-chr12_7898 | 5p | TCCTGATTTGCCGAGGCCTGAGGG | 4 | 8 | 0.777777778 |
| ssc-miR-novel-chr12_7955 | ssc-mir-novel-chr12_7955 | 3p | CGGCTGCGAGCAGACGGTC |  |  |  |
| ssc-miR-novel-chr12_7961 | ssc-mir-novel-chr12_7961 | 3p | CAGTGCAATAGTATTGTCAAAG | 2126 | 1700 | 1.249122807 |
| ssc-miR-novel-chr12_7963 | ssc-mir-novel-chr12_7963 | 3p | TAGTGCAATATTGCTTATAGGGT | 141 | 26 | 4.194444444 |
| ssc-miR-novel-chr12_7964 | ssc-mir-novel-chr12_7964 | 5p | TAGTGCAATATTGCTTATAGGGT | 141 | 26 | 4.194444444 |
| ssc-miR-novel-chr12_8144 | ssc-mir-novel-chr12_8144 | 5p | GACCATGGCTGTAGACTGTTA | 8 | 7 | 1.058823529 |
| ssc-miR-novel-chr12_8161 | ssc-mir-novel-chr12_8161 | 3p | CAGGCCTGGAGCTCTGCCTGCT | 38 | 19 | 1.655172414 |
| ssc-miR-novel-chr12_8220 | ssc-mir-novel-chr12_8220 | 5p | GGTGAGCACTCTGGACT |  |  |  |
| ssc-miR-novel-chr12_8221 | ssc-mir-novel-chr12_8221 | 3p | CGGGACCTGGTGCCCCTGTCGC |  |  |  |
| ssc-miR-novel-chr12_8235 | ssc-mir-novel-chr12_8235 | 3p | CACACACAGATCATTTCGTAGA |  |  |  |
| ssc-miR-novel-chr12_8265 | ssc-mir-novel-chr12_8265 | 3p | TTACAGTATTAGTCGCTTTT | 2 |  | 1.2 |
| ssc-miR-novel-chr12_8282 | ssc-mir-novel-chr12_8282 | 5p | TATCCCCATGGAGTCTGTTGCC | 67 | 2 | 6.416666667 |
| ssc-miR-novel-chr12_8290 | ssc-mir-novel-chr12_8290 | 5p | TATCCCCATGGAGTCTGTTGCC | 67 | 2 | 6.416666667 |
| ssc-miR-novel-chr12_8302 | ssc-mir-novel-chr12_8302 | 5p | GGTGAGCACTCTGGACT |  |  |  |
| ssc-miR-novel-chr12_8361 | ssc-mir-novel-chr12_8361 | 5p | CAGGGCACCGGCCTCTGCGTGGG |  |  |  |
| ssc-miR-novel-chr12_8369 | ssc-mir-novel-chr12_8369 | 5p | TCAACAAAATCACTGATGCTGGA |  |  |  |
| ssc-miR-novel-chr12_8374 | ssc-mir-novel-chr12_8374 | 3p | CAGGGCTTGGGGAGCAGAGAGA | 5 | 8 | 0.833333333 |
| ssc-miR-novel-chr12_8420 | ssc-mir-novel-chr12_8420 | 3p | TGGCTCAGCTCAGCAGGAG |  |  |  |
| ssc-miR-novel-chr12_8530 | ssc-mir-novel-chr12_8530 | 3p | TGCTCTGGAGTCAAGTCAGGA | 2 |  | 1.2 |
| ssc-miR-novel-chr12_8533 | ssc-mir-novel-chr12_8533 | 5p | CAGTGAATGGAGCCCTGAGA |  |  |  |
| ssc-miR-novel-chr12_8560 | ssc-mir-novel-chr12_8560 | 3p | TACAGCTTCTTGGAGTTCCCGTGT |  |  |  |
| ssc-miR-novel-chr12_8568 | ssc-mir-novel-chr12_8568 | 3p | CAGGACATGGAGAAAGGC |  |  |  |
| ssc-miR-novel-chr12_8591 | ssc-mir-novel-chr12_8591 | 5p | ACGGGATTGTAAAGGCAGAGCG |  |  |  |
| ssc-miR-novel-chr12_8594 | ssc-mir-novel-chr12_8594 | 3p | ACTGGGAGCAATGGAACGGCGA | 5 |  | 1.5 |
| ssc-miR-novel-chr12_8618 | ssc-mir-novel-chr12_8618 | 3p | CACGTGCGTGCTGGTATCTGG |  |  |  |
| ssc-miR-novel-chr12_8656 | ssc-mir-novel-chr12_8656 | 5p | TTTACGTCCTTTCACCTAGTTT |  |  |  |
| ssc-miR-novel-chr12_8665 | ssc-mir-novel-chr12_8665 | 5p | TCAGGATGAGACTCAAGGGGC |  |  |  |
| ssc-miR-novel-chr12_8771 | ssc-mir-novel-chr12_8771 | 5p | ACTGGACTTGGAGTCAGAAG | 76 | 33 | 2 |
| ssc-miR-novel-chr12_8979 | ssc-mir-novel-chr12_8979 | 5p | AGCTGGGCTGCTGTTCTCGAGGT | 4 |  | 1.4 |
| ssc-miR-novel-chr12_8980 | ssc-mir-novel-chr12_8980 | 3p | AGCTGGGCTGCTGTTCTCGAGGT | 4 |  | 1.4 |
| ssc-miR-novel-chr12_8997 | ssc-mir-novel-chr12_8997 | 5p | CACGGGTTCGATCCCTGGTGTGGGC |  |  |  |
| ssc-miR-novel-chr12_9131 | ssc-mir-novel-chr12_9131 | 5p | TGGGGTGGGGGCGTCGGGCC |  |  |  |
| ssc-miR-novel-chr12_9188 | ssc-mir-novel-chr12_9188 | 3p | AGCGCGGCTGGGCCTCCCCGA |  |  |  |
| ssc-miR-novel-chr13_10019 | ssc-mir-novel-chr13_10019 | 3p | GCGACCCATACTTGGTTTCAGA |  |  |  |
| ssc-miR-novel-chr13_10041 | ssc-mir-novel-chr13_10041 | 3p | TTGGAGTTTTGGAGCTGGG |  |  |  |
| ssc-miR-novel-chr13_10108 | ssc-mir-novel-chr13_10108 | 5p | AGGAGCAGGAGTCTGGGCTGAGG | 2 |  | 1.2 |
| ssc-miR-novel-chr13_10170 | ssc-mir-novel-chr13_10170 | 5p | AAGCAGGATTTAGACTACAATAT | 21 | 10 | 1.55 |
| ssc-miR-novel-chr13_10187 | ssc-mir-novel-chr13_10187 | 3p | AGAGGGCTGTGGGAGAGA | 10 |  | 2 |
| ssc-miR-novel-chr13_10484 | ssc-mir-novel-chr13_10484 | 5p | ACAGTGGCTGTGGCTCG | 9 | 3 | 1.461538462 |
| ssc-miR-novel-chr13_10658 | ssc-mir-novel-chr13_10658 | 5p | TCAGTAACAAAGATTCATCCTTG |  |  |  |
| ssc-miR-novel-chr13_10858 | ssc-mir-novel-chr13_10858 | 3p | CCTTCTCTGTAGGCCAGGGGCCCAG |  |  |  |
| ssc-miR-novel-chr13_10861 | ssc-mir-novel-chr13_10861 | 5p | TTCAAGTAACCCAGGATAGGCT | 671 | 138 | 4.601351351 |
| ssc-miR-novel-chr13_10908 | ssc-mir-novel-chr13_10908 | 3p | GGCCGGGCCCGCCCCCGC |  | 16 | 0.384615385 |
| ssc-miR-novel-chr13_10965 | ssc-mir-novel-chr13_10965 | 5p | GCTGTCTCTGTATGAATGTG |  |  |  |
| ssc-miR-novel-chr13_10983 | ssc-mir-novel-chr13_10983 | 5p | TGGGTGAGAGCACAGCAGAACT |  |  |  |
| ssc-miR-novel-chr13_11069 | ssc-mir-novel-chr13_11069 | 5p | TCTGGCTGTGGTGTAGA |  |  |  |
| ssc-miR-novel-chr13_11147 | ssc-mir-novel-chr13_11147 | 3p | AATCTGTCCACATATGGTGGTA |  |  |  |
| ssc-miR-novel-chr13_11172 | ssc-mir-novel-chr13_11172 | 3p | CCCTGTCTTTTGCTTCTCCTTT | 3 |  | 1.3 |
| ssc-miR-novel-chr13_11226 | ssc-mir-novel-chr13_11226 | 3p | CTTCCTACCCAGTCCGGC |  |  |  |
| ssc-miR-novel-chr13_11289 | ssc-mir-novel-chr13_11289 | 5p | AGGGTCTAGGTCGCAGATACAGC | 2 |  | 1.2 |
| ssc-miR-novel-chr13_11457 | ssc-mir-novel-chr13_11457 | 5p | TCTGGCTTTAATCTCTGTCTTG | 11 | 2 | 1.75 |
| ssc-miR-novel-chr13_11481 | ssc-mir-novel-chr13_11481 | 5p | TCTGGCTGTGGTGTAGACC | 11 | 11 | 1 |
| ssc-miR-novel-chr13_11522 | ssc-mir-novel-chr13_11522 | 3p | CTGGTCTGTGTCTTTGTGAGAGC |  |  |  |
| ssc-miR-novel-chr13_11595 | ssc-mir-novel-chr13_11595 | 5p | AAGCCAGAGTCAGGGGACACTGT |  |  |  |
| ssc-miR-novel-chr13_11601 | ssc-mir-novel-chr13_11601 | 5p | TATCTGCCTGTATATATGCCT | 6 |  | 1.6 |
| ssc-miR-novel-chr13_11602 | ssc-mir-novel-chr13_11602 | 3p | TATCTGCCTGTATATATGCCT | 6 |  | 1.6 |
| ssc-miR-novel-chr13_11720 | ssc-mir-novel-chr13_11720 | 3p | TGGGTGGCAAATGGTGGGTTTGA | 3 |  | 1.3 |
| ssc-miR-novel-chr13_11871 | ssc-mir-novel-chr13_11871 | 5p | GCGCAGCACATCATGGTTTA |  |  |  |
| ssc-miR-novel-chr13_11899 | ssc-mir-novel-chr13_11899 | 5p | ATTCTGTTAGAAAAATGCAAGA |  | 4 | 0.714285714 |
| ssc-miR-novel-chr13_11944 | ssc-mir-novel-chr13_11944 | 5p | TTTACATTGATTTCATATTGCT |  |  |  |
| ssc-miR-novel-chr13_12020 | ssc-mir-novel-chr13_12020 | 3p | AGACCTGGATTCATCAGCC |  |  |  |
| ssc-miR-novel-chr13_12094 | ssc-mir-novel-chr13_12094 | 3p | GCCTTGGCCTCTGCACCTGGTC |  |  |  |
| ssc-miR-novel-chr13_12101 | ssc-mir-novel-chr13_12101 | 5p | AAGGAGCTGAGGACGGAGAAGGA | 17 | 8 | 1.5 |
| ssc-miR-novel-chr13_9205 | ssc-mir-novel-chr13_9205 | 3p | ATTCTTTGCTGGATGGCATT |  |  |  |
| ssc-miR-novel-chr13_9254 | ssc-mir-novel-chr13_9254 | 5p | GAAATGAAGAACCCAGA |  |  |  |
| ssc-miR-novel-chr13_9348 | ssc-mir-novel-chr13_9348 | 5p | CTTGGAATTTTGCAGTGTCCA |  |  |  |
| ssc-miR-novel-chr13_9358 | ssc-mir-novel-chr13_9358 | 5p | AAGGTGGCTAATGCTTAGTGAGT |  |  |  |
| ssc-miR-novel-chr13_9430 | ssc-mir-novel-chr13_9430 | 5p | TTTGCTCTGCTCCTGCCACAT |  | 2 | 0.833333333 |
| ssc-miR-novel-chr13_9441 | ssc-mir-novel-chr13_9441 | 3p | GAGCGGGGCGCGCGGTCG |  | 9 | 0.526315789 |
| ssc-miR-novel-chr13_9442 | ssc-mir-novel-chr13_9442 | 5p | GAGCGGGGCGCGCGGTCG | 6 |  | 1.6 |
| ssc-miR-novel-chr13_9467 | ssc-mir-novel-chr13_9467 | 3p | TGTCCAAGACTGCAGGTCAGT |  |  |  |
| ssc-miR-novel-chr13_9486 | ssc-mir-novel-chr13_9486 | 3p | CCAGAGCACTCAAAAAGATGGC |  |  |  |
| ssc-miR-novel-chr13_9502 | ssc-mir-novel-chr13_9502 | 5p | TCCCACAGGGGGACGTGAGGCAG |  |  |  |
| ssc-miR-novel-chr13_9520 | ssc-mir-novel-chr13_9520 | 5p | TCCCACAGGGGGACGTGAGGCAG |  |  |  |
| ssc-miR-novel-chr13_9841 | ssc-mir-novel-chr13_9841 | 3p | CTGCGTCGGAGGGCGGCCGGAA |  |  |  |
| ssc-miR-novel-chr13_9887 | ssc-mir-novel-chr13_9887 | 3p | CAGGCATGAAGTCAGGTTAG |  |  |  |
| ssc-miR-novel-chr13_9979 | ssc-mir-novel-chr13_9979 | 3p | TTGTCAGTAGTTGCATGCAGGGA |  |  |  |
| ssc-miR-novel-chr13_9996 | ssc-mir-novel-chr13_9996 | 5p | AGGCATGGTCAAGAGGAAATC |  |  |  |
| ssc-miR-novel-chr14_12177 | ssc-mir-novel-chr14_12177 | 3p | TTTGTCTGTGGTGTAGGCCCGC | 23 | 18 | 1.178571429 |
| ssc-miR-novel-chr14_12179 | ssc-mir-novel-chr14_12179 | 3p | TTTGTCTGTGGTGTAGGCCCGC | 23 | 18 | 1.178571429 |
| ssc-miR-novel-chr14_12181 | ssc-mir-novel-chr14_12181 | 3p | TTTGTCTGTGGTGTAGGCCCGC | 23 | 18 | 1.178571429 |
| ssc-miR-novel-chr14_12274 | ssc-mir-novel-chr14_12274 | 5p | CAGATGGAGGCGTGGGT | 9 |  | 1.9 |
| ssc-miR-novel-chr14_12484 | ssc-mir-novel-chr14_12484 | 5p | AGCCAGGATTGTGGTGGCAGCCAG |  |  |  |
| ssc-miR-novel-chr14_12513 | ssc-mir-novel-chr14_12513 | 3p | TGGAAGGACACAGGAAGCCT | 3 |  | 1.3 |
| ssc-miR-novel-chr14_12542 | ssc-mir-novel-chr14_12542 | 5p | TTCCAGGGAAGAAAGGAGGAAC | 2 |  | 1.2 |
| ssc-miR-novel-chr14_12589 | ssc-mir-novel-chr14_12589 | 3p | AGCAGGACTGGCAGCTCTGGGCCTC |  |  |  |
| ssc-miR-novel-chr14_12636 | ssc-mir-novel-chr14_12636 | 5p | GTAGTACTTCTTGTTTGGATGCA | 2 |  | 1.2 |
| ssc-miR-novel-chr14_12638 | ssc-mir-novel-chr14_12638 | 5p | GTAGTACTTCTTGTTTGGATGCA | 2 |  | 1.2 |
| ssc-miR-novel-chr14_12801 | ssc-mir-novel-chr14_12801 | 3p | CTCTCTGACAGTCTCTGAAAGC |  |  |  |
| ssc-miR-novel-chr14_12811 | ssc-mir-novel-chr14_12811 | 3p | TGGGCTGTTCTTTTGTCTCTGAG |  |  |  |
| ssc-miR-novel-chr14_12851 | ssc-mir-novel-chr14_12851 | 3p | GACAGATCTCTCCTCCCACAGC |  | 4 | 0.714285714 |
| ssc-miR-novel-chr14_12856 | ssc-mir-novel-chr14_12856 | 5p | ATCTGGGATCTGCGGCCCCTGC |  |  |  |
| ssc-miR-novel-chr14_12862 | ssc-mir-novel-chr14_12862 | 5p | AAGGAAAGAATTCTAGTGGGG |  |  |  |
| ssc-miR-novel-chr14_12918 | ssc-mir-novel-chr14_12918 | 5p | CAGGAAACACTGGTGGAGG | 111 | 91 | 1.198019802 |
| ssc-miR-novel-chr14_13301 | ssc-mir-novel-chr14_13301 | 3p | CCAAACCAGTTGTGCCTGTAGA |  |  |  |
| ssc-miR-novel-chr14_13321 | ssc-mir-novel-chr14_13321 | 3p | CTGGAACTTGGCTGTGTCTCT |  |  |  |
| ssc-miR-novel-chr14_13411 | ssc-mir-novel-chr14_13411 | 3p | CGGGACGGGTGTCGGGG | 20 | 8 | 1.666666667 |
| ssc-miR-novel-chr14_13412 | ssc-mir-novel-chr14_13412 | 5p | CGGGACGGGTGTCGGGG | 20 | 8 | 1.666666667 |
| ssc-miR-novel-chr14_13435 | ssc-mir-novel-chr14_13435 | 3p | AGCTCCTCGAGGCCAGA |  |  |  |
| ssc-miR-novel-chr14_13436 | ssc-mir-novel-chr14_13436 | 5p | AGCTCCTCGAGGCCAGA |  |  |  |
| ssc-miR-novel-chr14_13460 | ssc-mir-novel-chr14_13460 | 3p | TCTATCGGGTGTACCTGTCCT | 3 | 7 | 0.764705882 |
| ssc-miR-novel-chr14_13655 | ssc-mir-novel-chr14_13655 | 5p | TGAAGGCTGGCCTAGAAATCT |  |  |  |
| ssc-miR-novel-chr14_13668 | ssc-mir-novel-chr14_13668 | 3p | TAAACTGAGGCCTCTGTGGGGC | 4 |  | 1.4 |
| ssc-miR-novel-chr14_13772 | ssc-mir-novel-chr14_13772 | 3p | TTCTCCAAGGACACTCTGTCTCT | 4 |  | 1.4 |
| ssc-miR-novel-chr14_13888 | ssc-mir-novel-chr14_13888 | 3p | CAGTGCAATGATATTGTCAAAGC | 595 | 556 | 1.068904594 |
| ssc-miR-novel-chr14_14550 | ssc-mir-novel-chr14_14550 | 3p | GAGGTTAACGGAGTGACA | 14 |  | 2.4 |
| ssc-miR-novel-chr14_14572 | ssc-mir-novel-chr14_14572 | 3p | TGGCACCAGCACTGGCGGTGGC |  |  |  |
| ssc-miR-novel-chr14_14582 | ssc-mir-novel-chr14_14582 | 3p | GTGGTTAAGGCTTTGGA |  |  |  |
| ssc-miR-novel-chr14_14584 | ssc-mir-novel-chr14_14584 | 3p | CAGACCCTGAGCTGCCTCTAGA | 3 |  | 1.3 |
| ssc-miR-novel-chr14_14614 | ssc-mir-novel-chr14_14614 | 3p | AGGAGTGTGGAAGGAGTG |  |  |  |
| ssc-miR-novel-chr15_14859 | ssc-mir-novel-chr15_14859 | 3p | CTGCTCTCCTGTCTCCGCTCAGG |  |  |  |
| ssc-miR-novel-chr15_14871 | ssc-mir-novel-chr15_14871 | 3p | TGCATGACTGTAGAAAACCTGT |  |  |  |
| ssc-miR-novel-chr15_14873 | ssc-mir-novel-chr15_14873 | 3p | TGCATGACTGTAGAAAACCTGT |  |  |  |
| ssc-miR-novel-chr15_14905 | ssc-mir-novel-chr15_14905 | 3p | CTTCTGTGATGGTGAACTGAGA |  |  |  |
| ssc-miR-novel-chr15_14929 | ssc-mir-novel-chr15_14929 | 3p | CTGGCTGGAGAACTTCTACATG |  |  |  |
| ssc-miR-novel-chr15_15025 | ssc-mir-novel-chr15_15025 | 3p | GAGCGTAGTGTTCGCCGGCATA |  |  |  |
| ssc-miR-novel-chr15_15523 | ssc-mir-novel-chr15_15523 | 3p | TTTGTTCGTTCGGCTCGCGTGA | 337 | 130 | 2.478571429 |
| ssc-miR-novel-chr15_15531 | ssc-mir-novel-chr15_15531 | 3p | AGCCCGGCCGTACCCTGCAGG |  |  |  |
| ssc-miR-novel-chr15_15607 | ssc-mir-novel-chr15_15607 | 3p | GAGGCGGCCCCGGCCCGGTAT |  |  |  |
| ssc-miR-novel-chr15_15633 | ssc-mir-novel-chr15_15633 | 3p | TCAGCAGGTCAGGTTCCATAAT |  |  |  |
| ssc-miR-novel-chr15_15673 | ssc-mir-novel-chr15_15673 | 3p | GCATTGGGGGTTCAGGGG | 12 | 14 | 0.916666667 |
| ssc-miR-novel-chr15_15705 | ssc-mir-novel-chr15_15705 | 3p | TCTTGGACCCCTTCTCACAGGA |  | 3 | 0.769230769 |
| ssc-miR-novel-chr15_15729 | ssc-mir-novel-chr15_15729 | 3p | TCTATCGGGTGTACCTGTCCT | 3 | 7 | 0.764705882 |
| ssc-miR-novel-chr15_15788 | ssc-mir-novel-chr15_15788 | 3p | TCCCAGCTGGTCATTAATCCT |  | 2 | 0.833333333 |
| ssc-miR-novel-chr15_15866 | ssc-mir-novel-chr15_15866 | 3p | TTGTTCTGGTTGATGGGTGGGA |  |  |  |
| ssc-miR-novel-chr15_16235 | ssc-mir-novel-chr15_16235 | 5p | ATGGGATAATCACACCCCTGGGG |  |  |  |
| ssc-miR-novel-chr15_16275 | ssc-mir-novel-chr15_16275 | 5p | AGCCGGCGGCGGCGGCGA |  | 2 | 0.833333333 |
| ssc-miR-novel-chr15_16277 | ssc-mir-novel-chr15_16277 | 5p | AGCCGGCGGCGGCGGCGA |  | 2 | 0.833333333 |
| ssc-miR-novel-chr15_16436 | ssc-mir-novel-chr15_16436 | 3p | TTCGCGGCCCGCGCTGGGAGC |  |  |  |
| ssc-miR-novel-chr15_16518 | ssc-mir-novel-chr15_16518 | 3p | GGGCGGACTGTTAAACCG |  |  |  |
| ssc-miR-novel-chr15_16730 | ssc-mir-novel-chr15_16730 | 3p | TCAGCAGGTCAGGTTCCATAAT |  |  |  |
| ssc-miR-novel-chr15_16769 | ssc-mir-novel-chr15_16769 | 5p | TGCAGATGATGTGAGAGA |  |  |  |
| ssc-miR-novel-chr16_16851 | ssc-mir-novel-chr16_16851 | 3p | AGAGCTGAGGGCAGAGTCCAGGA |  |  |  |
| ssc-miR-novel-chr16_16917 | ssc-mir-novel-chr16_16917 | 3p | TTGGTGTACACTGGAATAGCT | 37 | 18 | 1.678571429 |
| ssc-miR-novel-chr16_16920 | ssc-mir-novel-chr16_16920 | 5p | ATGGACCACAAACTCAAATT |  |  |  |
| ssc-miR-novel-chr16_16922 | ssc-mir-novel-chr16_16922 | 5p | TGGTCTAGCGGTTAGGA |  |  |  |
| ssc-miR-novel-chr16_17112 | ssc-mir-novel-chr16_17112 | 5p | GGTGATGATGACGATGAAGCTGAAA |  |  |  |
| ssc-miR-novel-chr16_17181 | ssc-mir-novel-chr16_17181 | 3p | TCTGCCTGTAGGAATGCTGTAGG |  |  |  |
| ssc-miR-novel-chr16_17182 | ssc-mir-novel-chr16_17182 | 5p | TCTGCCTGTAGGAATGCTGTAGG |  |  |  |
| ssc-miR-novel-chr16_17203 | ssc-mir-novel-chr16_17203 | 3p | TGTGGGGCTGATGACACT | 8 | 2 | 1.5 |
| ssc-miR-novel-chr16_17338 | ssc-mir-novel-chr16_17338 | 5p | CGTGCGTCTCTGGGTGTGATGTCG |  |  |  |
| ssc-miR-novel-chr16_17347 | ssc-mir-novel-chr16_17347 | 3p | TGGAAGGACACAGGAAGCCT | 3 |  | 1.3 |
| ssc-miR-novel-chr16_17372 | ssc-mir-novel-chr16_17372 | 3p | GTCCTCGGCTGGGAGGAGGA |  |  |  |
| ssc-miR-novel-chr16_17391 | ssc-mir-novel-chr16_17391 | 5p | TGAGGTAGTAGGCTGTGTGG | 13 | 12 | 1.045454545 |
| ssc-miR-novel-chr16_17392 | ssc-mir-novel-chr16_17392 | 3p | TGAGGTAGTAGGCTGTGTGG | 202 | 193 | 1.044334975 |
| ssc-miR-novel-chr16_17559 | ssc-mir-novel-chr16_17559 | 5p | TGGCAGTGTATTGTTAGCTGGT | 119 | 40 | 2.58 |
| ssc-miR-novel-chr16_17561 | ssc-mir-novel-chr16_17561 | 5p | AGGCAGTGTATTGTTAGCTGGCT | 6 | 2 | 1.333333333 |
| ssc-miR-novel-chr16_17571 | ssc-mir-novel-chr16_17571 | 5p | TGGGTGGAAGAAGTGACC | 16 |  | 2.6 |
| ssc-miR-novel-chr16_17722 | ssc-mir-novel-chr16_17722 | 3p | TAGCCTGACGCTGATGATTGT |  |  |  |
| ssc-miR-novel-chr16_17741 | ssc-mir-novel-chr16_17741 | 5p | TAAGAAATAGGTCATTAACAGTA |  |  |  |
| ssc-miR-novel-chr16_17764 | ssc-mir-novel-chr16_17764 | 3p | TGTGGGGCTGATGACACT | 8 | 2 | 1.5 |
| ssc-miR-novel-chr16_17872 | ssc-mir-novel-chr16_17872 | 3p | AGGGGGAGGCAGGGGGGG |  |  |  |
| ssc-miR-novel-chr16_17883 | ssc-mir-novel-chr16_17883 | 5p | GCCCCCGGTGGCGGGGGGG |  |  |  |
| ssc-miR-novel-chr17_17993 | ssc-mir-novel-chr17_17993 | 3p | CGGGGCAGCTCAGTACAGGAC |  |  |  |
| ssc-miR-novel-chr17_17994 | ssc-mir-novel-chr17_17994 | 5p | CGGGGCAGCTCAGTACAGGAC |  |  |  |
| ssc-miR-novel-chr17_18002 | ssc-mir-novel-chr17_18002 | 5p | CGGTGGGGTGCAGTGCTGGACT |  |  |  |
| ssc-miR-novel-chr17_18117 | ssc-mir-novel-chr17_18117 | 5p | CTGGGCTGCCTCTGGGT |  |  |  |
| ssc-miR-novel-chr17_18122 | ssc-mir-novel-chr17_18122 | 3p | CAAGGCTCCACCTGCGCCCAAG |  |  |  |
| ssc-miR-novel-chr17_18126 | ssc-mir-novel-chr17_18126 | 5p | AGTGGTGAAATGTATTTAGGAC |  |  |  |
| ssc-miR-novel-chr17_18148 | ssc-mir-novel-chr17_18148 | 5p | TACAGTGGCTGTGGCTC | 9 | 3 | 1.461538462 |
| ssc-miR-novel-chr17_18173 | ssc-mir-novel-chr17_18173 | 3p | AATGATGCCCCTTAGAGTTGAGC |  |  |  |
| ssc-miR-novel-chr17_18175 | ssc-mir-novel-chr17_18175 | 3p | AATGATGCCCCTTAGAGTTGAGC |  |  |  |
| ssc-miR-novel-chr17_18195 | ssc-mir-novel-chr17_18195 | 5p | GGGTTAAGGATCTGGTGT | 6 |  | 1.6 |
| ssc-miR-novel-chr17_18444 | ssc-mir-novel-chr17_18444 | 5p | TATGGTCACTGCTGTGACACGGGT |  |  |  |
| ssc-miR-novel-chr17_18693 | ssc-mir-novel-chr17_18693 | 5p | AGTGGTGAAATGTATTTAGGAC |  |  |  |
| ssc-miR-novel-chr17_18954 | ssc-mir-novel-chr17_18954 | 3p | GGTTTTGGTCCTCGGCC | 50 | 23 | 1.818181818 |
| ssc-miR-novel-chr17_18970 | ssc-mir-novel-chr17_18970 | 3p | TGTGTCCTCAGTAACCT | 82 | 13 | 4 |
| ssc-miR-novel-chr17_18987 | ssc-mir-novel-chr17_18987 | 5p | AGGGTTGGGCGGAGGCTTT | 126 | 49 | 2.305084746 |
| ssc-miR-novel-chr18_19035 | ssc-mir-novel-chr18_19035 | 3p | GCGGGGGTGGCGGCGGG |  |  |  |
| ssc-miR-novel-chr18_19202 | ssc-mir-novel-chr18_19202 | 5p | TTGCCCCGTCGAAGCTGTCGGTG |  | 2 | 0.833333333 |
| ssc-miR-novel-chr18_19204 | ssc-mir-novel-chr18_19204 | 5p | TTGCCCCGTCGAAGCTGTCGGTG |  | 2 | 0.833333333 |
| ssc-miR-novel-chr18_19238 | ssc-mir-novel-chr18_19238 | 5p | TTTGGCACTAGCACATTTTTGCT | 13 | 17 | 0.851851852 |
| ssc-miR-novel-chr18_19266 | ssc-mir-novel-chr18_19266 | 5p | TTGTGTCAATATGCGATGATGT |  |  |  |
| ssc-miR-novel-chr18_19287 | ssc-mir-novel-chr18_19287 | 3p | TGGGGTGTGGTGGTGGTGGG |  |  |  |
| ssc-miR-novel-chr18_19390 | ssc-mir-novel-chr18_19390 | 5p | TGATGTCAGGATGAAGCTTGGACT |  |  |  |
| ssc-miR-novel-chr18_19420 | ssc-mir-novel-chr18_19420 | 5p | TTCTGGAAGATGTAGTCTGGA |  |  |  |
| ssc-miR-novel-chr18_19428 | ssc-mir-novel-chr18_19428 | 5p | TGAGCGGACAAGTTTGAGCCCCT |  |  |  |
| ssc-miR-novel-chr18_19466 | ssc-mir-novel-chr18_19466 | 5p | ACGAAGAACATTTGTGTAGCT |  |  |  |
| ssc-miR-novel-chr18_19490 | ssc-mir-novel-chr18_19490 | 3p | GTTCCCTCGTGGCTCAATGGGT |  |  |  |
| ssc-miR-novel-chr18_19647 | ssc-mir-novel-chr18_19647 | 5p | TGAGGGAGGAGTATGTGCTGTG |  |  |  |
| ssc-miR-novel-chr18_19712 | ssc-mir-novel-chr18_19712 | 3p | AACATGGAAGTGGATTGAGG |  |  |  |
| ssc-miR-novel-chr18_19745 | ssc-mir-novel-chr18_19745 | 5p | TTATGGCCCTTCGGTAATTCACT | 2 | 6 | 0.75 |
| ssc-miR-novel-chr18_19771 | ssc-mir-novel-chr18_19771 | 5p | TGAACAGAGGTCATGAACAGAGC | 4 |  | 1.4 |
| ssc-miR-novel-chr18_19783 | ssc-mir-novel-chr18_19783 | 5p | TTGTGTCAATATGCGATGATGT |  |  |  |
| ssc-miR-novel-chr18_19904 | ssc-mir-novel-chr18_19904 | 3p | TTCCCTTTGGGCCCTCAGG |  |  |  |
| ssc-miR-novel-chr18_20001 | ssc-mir-novel-chr18_20001 | 5p | CAAGGAGGAGTCAGATGAGGCTG | 2 |  | 1.2 |
| ssc-miR-novel-chr18_20013 | ssc-mir-novel-chr18_20013 | 5p | TACTGCAAAGTGATTGAGGAGC |  |  |  |
| ssc-miR-novel-chr18_20025 | ssc-mir-novel-chr18_20025 | 3p | GTTCCCTCGTGGCTCAATGGGT |  |  |  |
| ssc-miR-novel-chr1_1003 | ssc-mir-novel-chr1_1003 | 3p | TGGACTAACTGTGGTATTGGGA |  |  |  |
| ssc-miR-novel-chr1_1012 | ssc-mir-novel-chr1_1012 | 5p | TTCAGGTAAAAGACACACACT |  | 2 | 0.833333333 |
| ssc-miR-novel-chr1_1152 | ssc-mir-novel-chr1_1152 | 5p | AGGCCCTTACATAGTCAGACT |  |  |  |
| ssc-miR-novel-chr1_1175 | ssc-mir-novel-chr1_1175 | 3p | ACTGTGTGTGAGGAAGTC |  |  |  |
| ssc-miR-novel-chr1_1345 | ssc-mir-novel-chr1_1345 | 3p | TAACCCGACATTCAAGGCCTGT |  |  |  |
| ssc-miR-novel-chr1_1497 | ssc-mir-novel-chr1_1497 | 3p | TACCACAGGGCAGAACCACGT |  |  |  |
| ssc-miR-novel-chr1_1514 | ssc-mir-novel-chr1_1514 | 5p | TCTGATTTGGATTCCAGA |  |  |  |
| ssc-miR-novel-chr1_1558 | ssc-mir-novel-chr1_1558 | 5p | CGGGGCGGGCGGTCGCCCGGGGC |  |  |  |
| ssc-miR-novel-chr1_1613 | ssc-mir-novel-chr1_1613 | 3p | ATAAAACTTAGTCGCTGCT |  |  |  |
| ssc-miR-novel-chr1_1798 | ssc-mir-novel-chr1_1798 | 5p | TGGAAACACTTCTGCACAAACT | 5 | 3 | 1.153846154 |
| ssc-miR-novel-chr1_1972 | ssc-mir-novel-chr1_1972 | 5p | TTCCCTTTGTCATCCTTTGCCC |  |  |  |
| ssc-miR-novel-chr1_2074 | ssc-mir-novel-chr1_2074 | 5p | TTTAGTGTGATAATGGCGTTTG |  |  |  |
| ssc-miR-novel-chr1_2132 | ssc-mir-novel-chr1_2132 | 5p | TCGTTAGTTCTCTGAGGCTCCGA | 3 |  | 1.3 |
| ssc-miR-novel-chr1_2249 | ssc-mir-novel-chr1_2249 | 3p | AGACCGGGGACCGGAGCGAGCAGA |  |  |  |
| ssc-miR-novel-chr1_2311 | ssc-mir-novel-chr1_2311 | 5p | ATTTGGTGGATTGTGGCAGTA |  |  |  |
| ssc-miR-novel-chr1_2360 | ssc-mir-novel-chr1_2360 | 5p | CGGCAGACTGGGACTTGTTGTT |  | 2 | 0.833333333 |
| ssc-miR-novel-chr1_2748 | ssc-mir-novel-chr1_2748 | 5p | AAAGGAGAGGAAAGAAGG |  |  |  |
| ssc-miR-novel-chr1_2775 | ssc-mir-novel-chr1_2775 | 3p | ACTAGAAATTGGGTAGGACAGA |  |  |  |
| ssc-miR-novel-chr1_2959 | ssc-mir-novel-chr1_2959 | 3p | TGGGGTGGGGCTCTCAACCTCCAGA |  |  |  |
| ssc-miR-novel-chr1_2989 | ssc-mir-novel-chr1_2989 | 3p | GCGCGTCTTTTGGTGCTCTGCAG | 2 |  | 1.2 |
| ssc-miR-novel-chr1_3279 | ssc-mir-novel-chr1_3279 | 5p | CTCTAGAGGTGGATTTGCTGTGCTG |  |  |  |
| ssc-miR-novel-chr1_3280 | ssc-mir-novel-chr1_3280 | 3p | CTCTAGAGGTGGATTTGCTGTGCTG |  |  |  |
| ssc-miR-novel-chr1_3515 | ssc-mir-novel-chr1_3515 | 5p | AAACATTAGAATGTCTGAGTGG |  |  |  |
| ssc-miR-novel-chr1_3810 | ssc-mir-novel-chr1_3810 | 3p | ATAAAACTTAGTCGCTGCT |  |  |  |
| ssc-miR-novel-chr1_3836 | ssc-mir-novel-chr1_3836 | 3p | CGCCGGGGGGGCGGGGG |  |  |  |
| ssc-miR-novel-chr1_3896 | ssc-mir-novel-chr1_3896 | 3p | CAATTTAGGTTAATGACTGTC |  |  |  |
| ssc-miR-novel-chr1_3924 | ssc-mir-novel-chr1_3924 | 3p | TTGCCTGGGACTCTGGAACC |  |  |  |
| ssc-miR-novel-chr1_4044 | ssc-mir-novel-chr1_4044 | 3p | ACTGGTTGGATTGGAGACTAGG | 3 |  | 1.3 |
| ssc-miR-novel-chr1_4317 | ssc-mir-novel-chr1_4317 | 5p | CTGCCGTTGGGTCTGGGGTGT |  |  |  |
| ssc-miR-novel-chr1_4339 | ssc-mir-novel-chr1_4339 | 5p | TCGTTAGTTCTCTGAGGCTCCGA | 3 |  | 1.3 |
| ssc-miR-novel-chr1_4468 | ssc-mir-novel-chr1_4468 | 3p | AACTGTTCTACCTCTGAGTAGC |  |  |  |
| ssc-miR-novel-chr1_4539 | ssc-mir-novel-chr1_4539 | 5p | AAGGTTACTTGTTAGTTCAGGA |  |  |  |
| ssc-miR-novel-chr1_4584 | ssc-mir-novel-chr1_4584 | 3p | TATGGAGGGTAGACTGTACT |  |  |  |
| ssc-miR-novel-chr1_4647 | ssc-mir-novel-chr1_4647 | 5p | CAGAGCTGTTTGTGAACTGCAGGT | 7 | 9 | 0.894736842 |
| ssc-miR-novel-chr1_4720 | ssc-mir-novel-chr1_4720 | 3p | CTGGAGGAAGAGCTCCCTG |  |  |  |
| ssc-miR-novel-chr1_4762 | ssc-mir-novel-chr1_4762 | 3p | AGAGTCGGCTGCGCGGAGCC |  |  |  |
| ssc-miR-novel-chr1_4788 | ssc-mir-novel-chr1_4788 | 3p | TAGCTAAGTGCAAATATGTCCAT |  |  |  |
| ssc-miR-novel-chr1_4860 | ssc-mir-novel-chr1_4860 | 3p | ACTAGAAATTGGGTAGGACAGA |  |  |  |
| ssc-miR-novel-chr1_4929 | ssc-mir-novel-chr1_4929 | 3p | AGGCTGGGGGTGGGGCC |  |  |  |
| ssc-miR-novel-chr1_5015 | ssc-mir-novel-chr1_5015 | 5p | ACAGTCAACGGTCGGTGGTTT | 4 |  | 1.4 |
| ssc-miR-novel-chr1_5092 | ssc-mir-novel-chr1_5092 | 3p | CAGAAGGGGAGTCGGAGCAGA |  |  |  |
| ssc-miR-novel-chr1_5095 | ssc-mir-novel-chr1_5095 | 5p | TCTGGAGGACGCTAGAGCTGGGC |  |  |  |
| ssc-miR-novel-chr1_5102 | ssc-mir-novel-chr1_5102 | 3p | AGAATTGTGGCTGGACATCTGT |  |  |  |
| ssc-miR-novel-chr1_5156 | ssc-mir-novel-chr1_5156 | 3p | TGCAGTTGCCGGGAAGCGGGC |  |  |  |
| ssc-miR-novel-chr1_5195 | ssc-mir-novel-chr1_5195 | 5p | CATTATTACTCACGGTACGAGT |  |  |  |
| ssc-miR-novel-chr1_5196 | ssc-mir-novel-chr1_5196 | 3p | CATTATTACTCACGGTACGAGT |  |  |  |
| ssc-miR-novel-chr1_5208 | ssc-mir-novel-chr1_5208 | 3p | AAGGGTGGGTGGCCACTGTGC |  |  |  |
| ssc-miR-novel-chr1_946 | ssc-mir-novel-chr1_946 | 5p | TCCCTGTGGTCTAGGGGTTAGGA | 2 | 2 | 1 |
| ssc-miR-novel-chr2_20089 | ssc-mir-novel-chr2_20089 | 5p | CTGGGCGGACGTGGGGT |  |  |  |
| ssc-miR-novel-chr2_20131 | ssc-mir-novel-chr2_20131 | 3p | CCTCTCTTCCCTCACGGTCGCT | 4 |  | 1.4 |
| ssc-miR-novel-chr2_20138 | ssc-mir-novel-chr2_20138 | 5p | GACCTGTGATGTCGTTTCTGAGA |  |  |  |
| ssc-miR-novel-chr2_20190 | ssc-mir-novel-chr2_20190 | 5p | CTCGGGCCTGCGCGCGCGAGGA |  |  |  |
| ssc-miR-novel-chr2_20212 | ssc-mir-novel-chr2_20212 | 5p | GACTGGAAGTCTCAGGGGGTGT |  | 2 | 0.833333333 |
| ssc-miR-novel-chr2_20240 | ssc-mir-novel-chr2_20240 | 5p | ATGTGTGGGTCGGCACCGA |  |  |  |
| ssc-miR-novel-chr2_20261 | ssc-mir-novel-chr2_20261 | 3p | GGGCAAGCCTGCGGAGGTGTGG |  |  |  |
| ssc-miR-novel-chr2_20299 | ssc-mir-novel-chr2_20299 | 3p | CATTCTCTGGTGGCCTAGCAGG | 2 |  | 1.2 |
| ssc-miR-novel-chr2_20300 | ssc-mir-novel-chr2_20300 | 5p | CATTCTCTGGTGGCCTAGCAGG |  |  |  |
| ssc-miR-novel-chr2_20315 | ssc-mir-novel-chr2_20315 | 5p | CCACCCTCATCGCTGCCTGTGTGT |  |  |  |
| ssc-miR-novel-chr2_20345 | ssc-mir-novel-chr2_20345 | 3p | CTTTGTGGTCCTGTTCTT |  |  |  |
| ssc-miR-novel-chr2_20448 | ssc-mir-novel-chr2_20448 | 5p | GTGGGGAAGAACTACAAGACAGCT | 114 | 289 | 0.414715719 |
| ssc-miR-novel-chr2_20504 | ssc-mir-novel-chr2_20504 | 5p | AGTCTCAGTGTGGTCTTCAGAGA |  |  |  |
| ssc-miR-novel-chr2_20597 | ssc-mir-novel-chr2_20597 | 5p | TCGTTGGACACAGATCACTACACT | 4 |  | 1.4 |
| ssc-miR-novel-chr2_20662 | ssc-mir-novel-chr2_20662 | 5p | CAGTTCACAGTTCATAGGAGGATG |  |  |  |
| ssc-miR-novel-chr2_20682 | ssc-mir-novel-chr2_20682 | 5p | ATTCGGGTGCAGGGACTGAGAGC |  |  |  |
| ssc-miR-novel-chr2_20831 | ssc-mir-novel-chr2_20831 | 3p | CCTGGGGGTCTGAGGGGC | 2 | 6 | 0.75 |
| ssc-miR-novel-chr2_20848 | ssc-mir-novel-chr2_20848 | 5p | TAACCAATGTGCAGACTACTGT |  |  |  |
| ssc-miR-novel-chr2_20962 | ssc-mir-novel-chr2_20962 | 5p | TCCTGTGATGATCTCCCTGAGC |  |  |  |
| ssc-miR-novel-chr2_20965 | ssc-mir-novel-chr2_20965 | 3p | TGAGATGAGAGCCCTTGGCCACT | 24 | 17 | 1.259259259 |
| ssc-miR-novel-chr2_21078 | ssc-mir-novel-chr2_21078 | 5p | TAGTTGCTGTGGGAAGTGAGC |  |  |  |
| ssc-miR-novel-chr2_21186 | ssc-mir-novel-chr2_21186 | 5p | TAAAATGCTATTCTGTCACTGT |  |  |  |
| ssc-miR-novel-chr2_21261 | ssc-mir-novel-chr2_21261 | 3p | TGACAATGATGTATCCACTGAGC |  |  |  |
| ssc-miR-novel-chr2_21343 | ssc-mir-novel-chr2_21343 | 3p | ACTGAAGTGATGGTCTTGGGAC |  | 2 | 0.833333333 |
| ssc-miR-novel-chr2_21352 | ssc-mir-novel-chr2_21352 | 5p | ATGTTGGACTCAGAACTCTCACTC |  |  |  |
| ssc-miR-novel-chr2_21472 | ssc-mir-novel-chr2_21472 | 5p | CGCGTGCGCGTCGGGTC | 153 | 4201 | 0.038708145 |
| ssc-miR-novel-chr2_21617 | ssc-mir-novel-chr2_21617 | 3p | TGGATTTTTGGAGCTGG | 4 | 140 | 0.093333333 |
| ssc-miR-novel-chr2_21624 | ssc-mir-novel-chr2_21624 | 5p | TGAGATGAAGCACTGTAGCT | 63 | 26 | 2.027777778 |
| ssc-miR-novel-chr2_21667 | ssc-mir-novel-chr2_21667 | 3p | TTCACTGGTGCCCATGGAGGA |  |  |  |
| ssc-miR-novel-chr2_21682 | ssc-mir-novel-chr2_21682 | 3p | AGCAGCGGCGGCGATGGCAGGCT |  |  |  |
| ssc-miR-novel-chr2_21748 | ssc-mir-novel-chr2_21748 | 3p | CCTCTCTTCCCTCACGGTCGCT | 4 |  | 1.4 |
| ssc-miR-novel-chr2_21765 | ssc-mir-novel-chr2_21765 | 5p | AGCACTTACGGATTCTGGGGG | 2 |  | 1.2 |
| ssc-miR-novel-chr2_21774 | ssc-mir-novel-chr2_21774 | 3p | TTATCCGGGCTTGTGCTGAAGC | 13 | 8 | 1.277777778 |
| ssc-miR-novel-chr2_21790 | ssc-mir-novel-chr2_21790 | 3p | AGCACTGATGGAGCCTGAGC |  |  |  |
| ssc-miR-novel-chr2_21820 | ssc-mir-novel-chr2_21820 | 3p | TTTGTTGGCTCCTCTGAAGTGA | 836 | 928 | 0.901918977 |
| ssc-miR-novel-chr2_21982 | ssc-mir-novel-chr2_21982 | 3p | TTTCCTCATATTCATTCAGGAGT | 3 | 7 | 0.764705882 |
| ssc-miR-novel-chr2_22148 | ssc-mir-novel-chr2_22148 | 3p | TAGCTCCCTGTGCCAGTCCTGA |  |  |  |
| ssc-miR-novel-chr2_22149 | ssc-mir-novel-chr2_22149 | 5p | ATGGTTGGGAGGATTCTCAGA |  |  |  |
| ssc-miR-novel-chr2_22156 | ssc-mir-novel-chr2_22156 | 3p | TGTGTCTGTGACATCTGCTGGCA | 17 | 31 | 0.658536585 |
| ssc-miR-novel-chr2_22194 | ssc-mir-novel-chr2_22194 | 3p | TAGCTCTGCCTTGTCTCCCCAGC |  |  |  |
| ssc-miR-novel-chr2_22201 | ssc-mir-novel-chr2_22201 | 5p | TACTGTGGTGTCCTGTGTCTCTG |  |  |  |
| ssc-miR-novel-chr2_22202 | ssc-mir-novel-chr2_22202 | 3p | TACTGTGGTGTCCTGTGTCTCTG |  |  |  |
| ssc-miR-novel-chr2_22309 | ssc-mir-novel-chr2_22309 | 5p | AGGATACGTCGCAGACGTGGTT |  |  |  |
| ssc-miR-novel-chr2_22316 | ssc-mir-novel-chr2_22316 | 3p | CTGCCTGGACCCCTGCTCACCGC | 2 |  | 1.2 |
| ssc-miR-novel-chr2_22420 | ssc-mir-novel-chr2_22420 | 3p | AGCCTCTCTGCTCCTTCCCAGG |  |  |  |
| ssc-miR-novel-chr2_22433 | ssc-mir-novel-chr2_22433 | 5p | TCAGGGAGGGAACACAGGGAGC |  |  |  |
| ssc-miR-novel-chr2_22438 | ssc-mir-novel-chr2_22438 | 3p | CATGGCCCTCCACCCCTGCAGG |  |  |  |
| ssc-miR-novel-chr2_22453 | ssc-mir-novel-chr2_22453 | 5p | GTCTCGGGCTCTTGCGACCCA |  |  |  |
| ssc-miR-novel-chr2_22508 | ssc-mir-novel-chr2_22508 | 3p | GAGTCAGAGCTGCAGGTTGTGG |  |  |  |
| ssc-miR-novel-chr2_22514 | ssc-mir-novel-chr2_22514 | 3p | TCTGTGCTCTGAGAGGGGC | 2 |  | 1.2 |
| ssc-miR-novel-chr2_22525 | ssc-mir-novel-chr2_22525 | 5p | CGTGGGTGTGTGATGGCT |  |  |  |
| ssc-miR-novel-chr2_22565 | ssc-mir-novel-chr2_22565 | 5p | ACATTTAAGGAGGTGCTTGCT | 4 |  | 1.4 |
| ssc-miR-novel-chr2_22609 | ssc-mir-novel-chr2_22609 | 5p | GGAAAAAAGAAATGGAGATGG | 4 |  | 1.4 |
| ssc-miR-novel-chr2_22652 | ssc-mir-novel-chr2_22652 | 3p | AGGCTCTGATTGGATTGGGTG |  |  |  |
| ssc-miR-novel-chr2_22879 | ssc-mir-novel-chr2_22879 | 5p | CGCGTGCGCGTCGGGTC | 153 | 4201 | 0.038708145 |
| ssc-miR-novel-chr2_23068 | ssc-mir-novel-chr2_23068 | 5p | TTCCGGTCATTGTCTGTACGTGG |  | 3 | 0.769230769 |
| ssc-miR-novel-chr2_23096 | ssc-mir-novel-chr2_23096 | 3p | TTCACTGGTGCCCATGGAGGA |  |  |  |
| ssc-miR-novel-chr3_23111 | ssc-mir-novel-chr3_23111 | 3p | GCAGAGCAGCGGCAGGAGG |  |  |  |
| ssc-miR-novel-chr3_23137 | ssc-mir-novel-chr3_23137 | 3p | CTCTGCCACACCACCCGCCTGC | 2 |  | 1.2 |
| ssc-miR-novel-chr3_23139 | ssc-mir-novel-chr3_23139 | 3p | CTGCGGTGCTGTGTGCTTTGGT |  |  |  |
| ssc-miR-novel-chr3_23263 | ssc-mir-novel-chr3_23263 | 3p | AGCTGGTCTGGGAGTTCCCGGGT |  |  |  |
| ssc-miR-novel-chr3_23271 | ssc-mir-novel-chr3_23271 | 3p | TAATTTTATGTATAAGCTAGT | 102 | 41 | 2.196078431 |
| ssc-miR-novel-chr3_23293 | ssc-mir-novel-chr3_23293 | 3p | AGTGGGAGCTGTGTTGACTG |  |  |  |
| ssc-miR-novel-chr3_23295 | ssc-mir-novel-chr3_23295 | 3p | AGTGGGAGCTGTGTTGACTG |  |  |  |
| ssc-miR-novel-chr3_23361 | ssc-mir-novel-chr3_23361 | 3p | AGGAAACTCTGGGGGTGG | 3 | 9 | 0.684210526 |
| ssc-miR-novel-chr3_23365 | ssc-mir-novel-chr3_23365 | 3p | CTCCCTGCTCTTCGGCTCAGAGT |  |  |  |
| ssc-miR-novel-chr3_23411 | ssc-mir-novel-chr3_23411 | 3p | ATCTGTCTGTGTCTCTGAGCAG | 3 |  | 1.3 |
| ssc-miR-novel-chr3_23413 | ssc-mir-novel-chr3_23413 | 3p | GTCAGGATGGCCGAGTGGTCTAAGG |  |  |  |
| ssc-miR-novel-chr3_23465 | ssc-mir-novel-chr3_23465 | 3p | CAGGGTCGGGCCTGGTTA | 137 | 27 | 3.972972973 |
| ssc-miR-novel-chr3_23507 | ssc-mir-novel-chr3_23507 | 3p | TCTGAGACGGACATCTGGTCC | 2 |  | 1.2 |
| ssc-miR-novel-chr3_23547 | ssc-mir-novel-chr3_23547 | 3p | CTGGGCTGTGCCGGGTGC |  |  |  |
| ssc-miR-novel-chr3_23548 | ssc-mir-novel-chr3_23548 | 5p | CTGGGCTGTGCCGGGTGC |  |  |  |
| ssc-miR-novel-chr3_23558 | ssc-mir-novel-chr3_23558 | 5p | CGGGGCTGGGCGCGCGC | 6 |  | 1.6 |
| ssc-miR-novel-chr3_23681 | ssc-mir-novel-chr3_23681 | 3p | CAGACCAGCAGGATGTGGACT | 4 | 2 | 1.166666667 |
| ssc-miR-novel-chr3_23694 | ssc-mir-novel-chr3_23694 | 5p | GGGCATCCCTGTAGGAGCT |  |  |  |
| ssc-miR-novel-chr3_23708 | ssc-mir-novel-chr3_23708 | 5p | AACACATGTGTGCTGAGTGGAACT | 2 |  | 1.2 |
| ssc-miR-novel-chr3_23955 | ssc-mir-novel-chr3_23955 | 3p | TGTTGTTCTGAAGGTGA |  |  |  |
| ssc-miR-novel-chr3_24036 | ssc-mir-novel-chr3_24036 | 5p | AGGCTGAGGCTGGAGGA |  |  |  |
| ssc-miR-novel-chr3_24093 | ssc-mir-novel-chr3_24093 | 3p | GCACCTTTATTCTTCTAACTG |  |  |  |
| ssc-miR-novel-chr3_24096 | ssc-mir-novel-chr3_24096 | 5p | CTCCCGTGCTGATCAGTAGTGGG |  | 7 | 0.588235294 |
| ssc-miR-novel-chr3_24244 | ssc-mir-novel-chr3_24244 | 5p | TTCCCTTTGTAATCCTATGCAT |  |  |  |
| ssc-miR-novel-chr3_24308 | ssc-mir-novel-chr3_24308 | 5p | CCAGTGATCAGGTTACGATGGATT |  | 7 | 0.588235294 |
| ssc-miR-novel-chr3_24388 | ssc-mir-novel-chr3_24388 | 3p | GCAGAGCAGCGGCAGGAGG |  |  |  |
| ssc-miR-novel-chr3_24518 | ssc-mir-novel-chr3_24518 | 5p | CCGGGAACTCCCAGACCAGCTTC |  |  |  |
| ssc-miR-novel-chr3_24548 | ssc-mir-novel-chr3_24548 | 3p | ACATTCAGAGAACTGTAAACGCC | 4 | 3 | 1.076923077 |
| ssc-miR-novel-chr3_24599 | ssc-mir-novel-chr3_24599 | 5p | GTAGACCCGGGACTTCGCTG |  |  |  |
| ssc-miR-novel-chr3_24618 | ssc-mir-novel-chr3_24618 | 3p | CTGCCCACCTGGGTGAGAAGGA |  |  |  |
| ssc-miR-novel-chr3_24739 | ssc-mir-novel-chr3_24739 | 5p | TGCAGGACCTCAGAGCAGCT |  |  |  |
| ssc-miR-novel-chr3_24773 | ssc-mir-novel-chr3_24773 | 5p | AGGAGTGGTTGCTGGCAGTGTGC |  |  |  |
| ssc-miR-novel-chr3_24811 | ssc-mir-novel-chr3_24811 | 5p | TGGCTCTGCGAGGTCGGCTCA | 114 | 87 | 1.278350515 |
| ssc-miR-novel-chr3_24812 | ssc-mir-novel-chr3_24812 | 3p | TGGCTCTGCGAGGTCGGCTCA | 114 | 87 | 1.278350515 |
| ssc-miR-novel-chr3_24814 | ssc-mir-novel-chr3_24814 | 3p | CGAGCGGGCCCGGACGCTGCTGGC |  |  |  |
| ssc-miR-novel-chr3_24923 | ssc-mir-novel-chr3_24923 | 5p | GGGCATCCCTGTAGGAGCT |  |  |  |
| ssc-miR-novel-chr3_24929 | ssc-mir-novel-chr3_24929 | 5p | ACACTCAGCATGCACATGGAGGCT |  |  |  |
| ssc-miR-novel-chr3_24974 | ssc-mir-novel-chr3_24974 | 3p | CTGGGAGCAGTTGCCGGATGTG |  |  |  |
| ssc-miR-novel-chr3_25028 | ssc-mir-novel-chr3_25028 | 3p | TGTGGCTGTGGCCTGCAACT | 38 | 24 | 1.411764706 |
| ssc-miR-novel-chr3_25115 | ssc-mir-novel-chr3_25115 | 5p | TGGTTGGAGGAGCTGAGAGCGC |  |  |  |
| ssc-miR-novel-chr3_25160 | ssc-mir-novel-chr3_25160 | 3p | TAGTGCAGGTTGGATCCCTAGC |  |  |  |
| ssc-miR-novel-chr3_25222 | ssc-mir-novel-chr3_25222 | 5p | GCGTCACCAGCCGAGCCGG |  |  |  |
| ssc-miR-novel-chr3_25240 | ssc-mir-novel-chr3_25240 | 3p | TTCACAGGGGCTCAGTTC |  |  |  |
| ssc-miR-novel-chr3_25266 | ssc-mir-novel-chr3_25266 | 3p | AAGAGGGGCTTGGAGCTGGGCCT |  |  |  |
| ssc-miR-novel-chr3_25267 | ssc-mir-novel-chr3_25267 | 5p | AGATTCAGTCAACCGCGGATC | 2 |  | 1.2 |
| ssc-miR-novel-chr3_25338 | ssc-mir-novel-chr3_25338 | 3p | GATATGAGAGTGTTGGTCCTGA | 4 | 4 | 1 |
| ssc-miR-novel-chr3_25350 | ssc-mir-novel-chr3_25350 | 3p | AGTGGCTGTGGCTCGACCCCT | 9 | 9 | 1 |
| ssc-miR-novel-chr3_25488 | ssc-mir-novel-chr3_25488 | 3p | CCCGGGGCTGCAGAAAGA |  |  |  |
| ssc-miR-novel-chr3_25504 | ssc-mir-novel-chr3_25504 | 3p | TTTCGGACCACAGCCTGGGCATC | 3 |  | 1.3 |
| ssc-miR-novel-chr3_25572 | ssc-mir-novel-chr3_25572 | 3p | CTGTCCTGGGCCTGTCAGAGTCTCC |  |  |  |
| ssc-miR-novel-chr4_25596 | ssc-mir-novel-chr4_25596 | 5p | GTGAAGGGTGCAGGTTCGGG |  |  |  |
| ssc-miR-novel-chr4_25600 | ssc-mir-novel-chr4_25600 | 5p | GCGCCGCGCTGGGAGCCCTGCGGA |  | 3 | 0.769230769 |
| ssc-miR-novel-chr4_25612 | ssc-mir-novel-chr4_25612 | 5p | CCCACGGTCCACCACTTTGCTGT | 2 |  | 1.2 |
| ssc-miR-novel-chr4_25615 | ssc-mir-novel-chr4_25615 | 3p | CGTTGTGATGAAGTGCTGAGG |  |  |  |
| ssc-miR-novel-chr4_25671 | ssc-mir-novel-chr4_25671 | 3p | GGGTCGGGGCGGGGCGG |  |  |  |
| ssc-miR-novel-chr4_25720 | ssc-mir-novel-chr4_25720 | 5p | AGGAGAAAGCAGACGGGCTGCA | 12 | 12 | 1 |
| ssc-miR-novel-chr4_25914 | ssc-mir-novel-chr4_25914 | 5p | TATACCTCAGTTTTATCAGGTG |  |  |  |
| ssc-miR-novel-chr4_25916 | ssc-mir-novel-chr4_25916 | 5p | TTTGATAAGCTGACATGGGACA |  |  |  |
| ssc-miR-novel-chr4_26000 | ssc-mir-novel-chr4_26000 | 5p | TTCCTGAGTCGGACTGGGCTGC |  |  |  |
| ssc-miR-novel-chr4_26125 | ssc-mir-novel-chr4_26125 | 3p | ATGGATTGTCTTCTGGGTACT |  |  |  |
| ssc-miR-novel-chr4_26129 | ssc-mir-novel-chr4_26129 | 3p | TCAAGGTCCGCTGTGAACACGG | 4 |  | 1.4 |
| ssc-miR-novel-chr4_26236 | ssc-mir-novel-chr4_26236 | 5p | CTAGAGTGGATTCTGCA |  |  |  |
| ssc-miR-novel-chr4_26332 | ssc-mir-novel-chr4_26332 | 5p | GTCACCTGGCAGGTACCTCTTT |  |  |  |
| ssc-miR-novel-chr4_26365 | ssc-mir-novel-chr4_26365 | 3p | TCATGAGAAAGTGCCTGGAAC | 5 |  | 1.5 |
| ssc-miR-novel-chr4_26369 | ssc-mir-novel-chr4_26369 | 3p | CGGACAGCTCGAAAGGGG |  |  |  |
| ssc-miR-novel-chr4_26379 | ssc-mir-novel-chr4_26379 | 3p | TCGGCGCCCCACCCTCTCTAGC | 3 |  | 1.3 |
| ssc-miR-novel-chr4_26443 | ssc-mir-novel-chr4_26443 | 3p | ACAGGACCCTACCCATCAAGA |  |  |  |
| ssc-miR-novel-chr4_26606 | ssc-mir-novel-chr4_26606 | 5p | TGGGGTTCAAAAGGACTCAAGA |  |  |  |
| ssc-miR-novel-chr4_26620 | ssc-mir-novel-chr4_26620 | 5p | TGGGGTTCAAAAGGACTCAAGA |  |  |  |
| ssc-miR-novel-chr4_26624 | ssc-mir-novel-chr4_26624 | 5p | TGGGGTTCAAAAGGACTCAAGA |  |  |  |
| ssc-miR-novel-chr4_26691 | ssc-mir-novel-chr4_26691 | 3p | GCCAGCCGGTGTTCATGCCCCATA |  |  |  |
| ssc-miR-novel-chr4_26743 | ssc-mir-novel-chr4_26743 | 3p | TGAGAGGAAGCACTGTAGGA |  |  |  |
| ssc-miR-novel-chr4_26821 | ssc-mir-novel-chr4_26821 | 3p | TCAGAAGTGGGTCCAGGAATCT | 7 |  | 1.7 |
| ssc-miR-novel-chr4_26845 | ssc-mir-novel-chr4_26845 | 3p | TCTTCCCAGGCTCTGTCTGAAC |  |  |  |
| ssc-miR-novel-chr4_26898 | ssc-mir-novel-chr4_26898 | 5p | AGTCCACTCGTCTCACTGAGA |  |  |  |
| ssc-miR-novel-chr4_26969 | ssc-mir-novel-chr4_26969 | 5p | CGGGGTGTGGGGAGGGC |  | 2 | 0.833333333 |
| ssc-miR-novel-chr4_27089 | ssc-mir-novel-chr4_27089 | 5p | CCTCTGGGCCACAGGAGAACT |  |  |  |
| ssc-miR-novel-chr4_27349 | ssc-mir-novel-chr4_27349 | 5p | TCACCTGTTGGATATTCT | 11 |  | 2.1 |
| ssc-miR-novel-chr4_27402 | ssc-mir-novel-chr4_27402 | 3p | ATGGATTGTCTTCTGGGTACT |  |  |  |
| ssc-miR-novel-chr4_27454 | ssc-mir-novel-chr4_27454 | 3p | CAGGACCAGGTGAGCGCCAGC |  | 3 | 0.769230769 |
| ssc-miR-novel-chr4_27574 | ssc-mir-novel-chr4_27574 | 3p | TCCCAAGACTCCTTTGATCCCT |  | 2 | 0.833333333 |
| ssc-miR-novel-chr4_27701 | ssc-mir-novel-chr4_27701 | 3p | TGTTGTCCTCCGAATCTGAAT |  |  |  |
| ssc-miR-novel-chr4_27704 | ssc-mir-novel-chr4_27704 | 3p | TCTCTGACTCTCTGACCTCCCAGG |  |  |  |
| ssc-miR-novel-chr4_27733 | ssc-mir-novel-chr4_27733 | 5p | CTGTGTGGTGTAGGGAGAAGCT |  |  |  |
| ssc-miR-novel-chr4_27870 | ssc-mir-novel-chr4_27870 | 5p | CACTCTGGACTCTGAATC |  | 3 | 0.769230769 |
| ssc-miR-novel-chr4_27873 | ssc-mir-novel-chr4_27873 | 5p | TGGGGTTCCGAAGGACTAAGA |  |  |  |
| ssc-miR-novel-chr4_27885 | ssc-mir-novel-chr4_27885 | 5p | TGGGGTTCCGAAGGACTAAGA |  |  |  |
| ssc-miR-novel-chr4_27887 | ssc-mir-novel-chr4_27887 | 5p | TGGGGTTCCGAAGGACTAAGA |  |  |  |
| ssc-miR-novel-chr4_27893 | ssc-mir-novel-chr4_27893 | 5p | TGAGAACTGAATTCGATGGGA |  |  |  |
| ssc-miR-novel-chr4_27928 | ssc-mir-novel-chr4_27928 | 3p | TGAGACACAGGGCGGGCTGTGCT |  |  |  |
| ssc-miR-novel-chr4_27930 | ssc-mir-novel-chr4_27930 | 3p | TATATATATATGATTCACGATG |  |  |  |
| ssc-miR-novel-chr4_27970 | ssc-mir-novel-chr4_27970 | 3p | CAGTCGGGACGCTTCCTTCTT |  |  |  |
| ssc-miR-novel-chr4_28073 | ssc-mir-novel-chr4_28073 | 5p | ACGGAGGCCTCTTACCAGCA |  |  |  |
| ssc-miR-novel-chr4_28122 | ssc-mir-novel-chr4_28122 | 3p | CGGCTCTGGGTCTGTGGGGAGC | 76 | 18 | 3.071428571 |
| ssc-miR-novel-chr5_28279 | ssc-mir-novel-chr5_28279 | 3p | ATGCGGAACCTGCGGATACGG | 126 | 61 | 1.915492958 |
| ssc-miR-novel-chr5_28309 | ssc-mir-novel-chr5_28309 | 3p | TCAGTGGGCACCCAGCCGGAGT | 2 |  | 1.2 |
| ssc-miR-novel-chr5_28311 | ssc-mir-novel-chr5_28311 | 3p | TCAGTGGGCACCCAGCCGGAGT | 2 |  | 1.2 |
| ssc-miR-novel-chr5_28315 | ssc-mir-novel-chr5_28315 | 3p | CTCAGACCTCAGGCTTGGAGCC | 3 |  | 1.3 |
| ssc-miR-novel-chr5_28386 | ssc-mir-novel-chr5_28386 | 5p | TGGGACTTATAGGTGAACCAGA |  |  |  |
| ssc-miR-novel-chr5_28455 | ssc-mir-novel-chr5_28455 | 3p | TGGGAAGTGTCCAGGTTGGGA |  |  |  |
| ssc-miR-novel-chr5_28480 | ssc-mir-novel-chr5_28480 | 5p | TTAGGAGAGGAGCTGAGAAAGGG | 2 |  | 1.2 |
| ssc-miR-novel-chr5_28483 | ssc-mir-novel-chr5_28483 | 3p | TGATGTTTATCCGAATCCTCAGA |  |  |  |
| ssc-miR-novel-chr5_28488 | ssc-mir-novel-chr5_28488 | 5p | TTAGGAGAGGAGCTGAGAAAGGG | 2 |  | 1.2 |
| ssc-miR-novel-chr5_28715 | ssc-mir-novel-chr5_28715 | 3p | TGGTGGCAGAGCCAGGTAA |  |  |  |
| ssc-miR-novel-chr5_28736 | ssc-mir-novel-chr5_28736 | 5p | AAGGATGGGAAGGAGGGG |  |  |  |
| ssc-miR-novel-chr5_28811 | ssc-mir-novel-chr5_28811 | 3p | CAACACTGTGCTGGAAGATGGA |  | 3 | 0.769230769 |
| ssc-miR-novel-chr5_28846 | ssc-mir-novel-chr5_28846 | 5p | TGGGACTGAAGGCCGCGGCCTCC |  |  |  |
| ssc-miR-novel-chr5_28892 | ssc-mir-novel-chr5_28892 | 5p | CGCAGGGCTCGGGTCGGCTGCCT | 13 | 4 | 1.642857143 |
| ssc-miR-novel-chr5_29091 | ssc-mir-novel-chr5_29091 | 3p | TGCAAGGTCGGACCGCTGTGACC |  |  |  |
| ssc-miR-novel-chr5_29097 | ssc-mir-novel-chr5_29097 | 3p | TGCAAGGTCGGACCGCTGTGACC |  |  |  |
| ssc-miR-novel-chr5_29128 | ssc-mir-novel-chr5_29128 | 3p | TGGCTCTGCGAGGTCGGCT | 82 | 103 | 0.814159292 |
| ssc-miR-novel-chr5_29297 | ssc-mir-novel-chr5_29297 | 5p | GCGGGGGTGGCGGCGGG |  |  |  |
| ssc-miR-novel-chr5_29298 | ssc-mir-novel-chr5_29298 | 3p | GCGGGGGTGGCGGCGGG |  |  |  |
| ssc-miR-novel-chr5_29391 | ssc-mir-novel-chr5_29391 | 5p | TGCGTTCATTCTGTTTGGCCT | 6 |  | 1.6 |
| ssc-miR-novel-chr5_29426 | ssc-mir-novel-chr5_29426 | 3p | TGATGTTTATCCGAATCCTCAGA |  |  |  |
| ssc-miR-novel-chr5_29480 | ssc-mir-novel-chr5_29480 | 3p | TCTGAGATGTGACCTGGGCAT | 32 | 29 | 1.076923077 |
| ssc-miR-novel-chr5_29487 | ssc-mir-novel-chr5_29487 | 5p | GACTTAATGGCTGGCTGGGAGG | 39 |  | 4.9 |
| ssc-miR-novel-chr5_29564 | ssc-mir-novel-chr5_29564 | 3p | CAGTGTGGGACCTTGGGCCTCC |  |  |  |
| ssc-miR-novel-chr5_29627 | ssc-mir-novel-chr5_29627 | 5p | TCTCCAGTGAGACAGTCTCT |  | 6 | 0.625 |
| ssc-miR-novel-chr5_29674 | ssc-mir-novel-chr5_29674 | 3p | TAACACTGTCTGGTAAAGATG | 257 | 131 | 1.893617021 |
| ssc-miR-novel-chr5_29676 | ssc-mir-novel-chr5_29676 | 3p | TAATACTGCCGGGTAATGATGGA | 2 |  | 1.2 |
| ssc-miR-novel-chr5_29710 | ssc-mir-novel-chr5_29710 | 3p | TGTCGATGATGGCAGTGCTGAGG |  |  |  |
| ssc-miR-novel-chr5_29774 | ssc-mir-novel-chr5_29774 | 3p | TCGGCTGCAGACACCACGCC |  |  |  |
| ssc-miR-novel-chr5_29781 | ssc-mir-novel-chr5_29781 | 5p | TAAAGTTATATAAGGGTTTTTG |  | 3 | 0.769230769 |
| ssc-miR-novel-chr5_29793 | ssc-mir-novel-chr5_29793 | 5p | AAATGAAAAGGATTGGTTTCT |  |  |  |
| ssc-miR-novel-chr5_29857 | ssc-mir-novel-chr5_29857 | 5p | GCAGCAGGTCTCCAAGGGG | 4 | 2 | 1.166666667 |
| ssc-miR-novel-chr5_29868 | ssc-mir-novel-chr5_29868 | 3p | TCTCAGGGATGGTGGAAATAGCCT |  |  |  |
| ssc-miR-novel-chr5_29905 | ssc-mir-novel-chr5_29905 | 5p | ACTCTAGCTGCCAAAGGCGCT |  |  |  |
| ssc-miR-novel-chr5_30018 | ssc-mir-novel-chr5_30018 | 3p | CAAGGCCACTGACTGAAGAGCAGA |  |  |  |
| ssc-miR-novel-chr5_30028 | ssc-mir-novel-chr5_30028 | 3p | TGACTCCCGGCCTGCTGGCAGG |  |  |  |
| ssc-miR-novel-chr6_30041 | ssc-mir-novel-chr6_30041 | 3p | GGCTGGGAGCCTGGGCTTCGTGG |  |  |  |
| ssc-miR-novel-chr6_30050 | ssc-mir-novel-chr6_30050 | 5p | TGAGGGCCTCGCCAGCCCCCGGC |  |  |  |
| ssc-miR-novel-chr6_30116 | ssc-mir-novel-chr6_30116 | 3p | CACGAGAAACCGCGTCCCGCCAGG |  |  |  |
| ssc-miR-novel-chr6_30192 | ssc-mir-novel-chr6_30192 | 5p | TCCTCTGGAGGCTCGAGAAGA |  |  |  |
| ssc-miR-novel-chr6_30243 | ssc-mir-novel-chr6_30243 | 5p | AGGAGGAAGAAGAAACTG |  |  |  |
| ssc-miR-novel-chr6_30244 | ssc-mir-novel-chr6_30244 | 5p | AGGAGGAAGAAGAAACTG |  |  |  |
| ssc-miR-novel-chr6_30278 | ssc-mir-novel-chr6_30278 | 5p | AGGAAAGTGTGGTGGAGG |  |  |  |
| ssc-miR-novel-chr6_30281 | ssc-mir-novel-chr6_30281 | 3p | ATCCGGGAGCTGGGAGCC |  |  |  |
| ssc-miR-novel-chr6_30340 | ssc-mir-novel-chr6_30340 | 5p | TCCCGGGGTGTGGGGAGG | 4 | 7 | 0.823529412 |
| ssc-miR-novel-chr6_30650 | ssc-mir-novel-chr6_30650 | 5p | ACTCAAACTGTGGGGGCACTT | 3 | 7 | 0.764705882 |
| ssc-miR-novel-chr6_30675 | ssc-mir-novel-chr6_30675 | 3p | GACTCCTGCCCCTCTCCCACAGG |  |  |  |
| ssc-miR-novel-chr6_30729 | ssc-mir-novel-chr6_30729 | 3p | TAATACTGCCTGGTAATGATGA | 13 | 6 | 1.4375 |
| ssc-miR-novel-chr6_30750 | ssc-mir-novel-chr6_30750 | 5p | CGGGGAGGCTGTGCAGCGCGGC |  | 2 | 0.833333333 |
| ssc-miR-novel-chr6_30792 | ssc-mir-novel-chr6_30792 | 5p | GGGGCCGGGGGTGGGGCC |  |  |  |
| ssc-miR-novel-chr6_30857 | ssc-mir-novel-chr6_30857 | 3p | CATGGATGTGGTGATGTGG | 38 | 286 | 0.162162162 |
| ssc-miR-novel-chr6_30884 | ssc-mir-novel-chr6_30884 | 5p | TAGCAGGAGGAGGACTCTG |  |  |  |
| ssc-miR-novel-chr6_30922 | ssc-mir-novel-chr6_30922 | 5p | ACCAACGTGGATACCCCGGG | 5 |  | 1.5 |
| ssc-miR-novel-chr6_30930 | ssc-mir-novel-chr6_30930 | 5p | TTGCTGTGATGACAAAT | 2 | 7 | 0.705882353 |
| ssc-miR-novel-chr6_30947 | ssc-mir-novel-chr6_30947 | 3p | CGGGCGGGAGCGGCCGGG |  | 2 | 0.833333333 |
| ssc-miR-novel-chr6_31079 | ssc-mir-novel-chr6_31079 | 3p | TGAGATGAAGCTCTGTGTCT |  |  |  |
| ssc-miR-novel-chr6_31216 | ssc-mir-novel-chr6_31216 | 5p | GTTTTGGTGGGGGCCGGGG | 4 |  | 1.4 |
| ssc-miR-novel-chr6_31226 | ssc-mir-novel-chr6_31226 | 5p | CAAGTCTTTGGGTTCCAG | 2 | 4 | 0.857142857 |
| ssc-miR-novel-chr6_31268 | ssc-mir-novel-chr6_31268 | 5p | TCATGATATAGAGGTAAATAGT | 9 | 3 | 1.461538462 |
| ssc-miR-novel-chr6_31382 | ssc-mir-novel-chr6_31382 | 5p | CTATGTGCCTGAGAACTT |  |  |  |
| ssc-miR-novel-chr6_31392 | ssc-mir-novel-chr6_31392 | 5p | TCTCAGGATGAGGTAAGATTGCT |  |  |  |
| ssc-miR-novel-chr6_31419 | ssc-mir-novel-chr6_31419 | 3p | TGCTGTGATGGGGGCTCTGAGA |  |  |  |
| ssc-miR-novel-chr6_31436 | ssc-mir-novel-chr6_31436 | 5p | CGCTTCGGCGGCGGGGG | 72 | 118 | 0.640625 |
| ssc-miR-novel-chr6_31474 | ssc-mir-novel-chr6_31474 | 5p | AGGTGGGATCCCGAGGC | 31 | 8 | 2.277777778 |
| ssc-miR-novel-chr6_31487 | ssc-mir-novel-chr6_31487 | 3p | CAAGTGCTAATGTTGGGA |  |  |  |
| ssc-miR-novel-chr6_31513 | ssc-mir-novel-chr6_31513 | 5p | CTCCCCCCCTTCCCGGG |  |  |  |
| ssc-miR-novel-chr6_31604 | ssc-mir-novel-chr6_31604 | 3p | TGTGTGTGGGCGCCGGACGCC | 18 | 11 | 1.333333333 |
| ssc-miR-novel-chr6_31692 | ssc-mir-novel-chr6_31692 | 3p | TCTGGCTGTGGTGTAGACCGTC | 167 | 65 | 2.36 |
| ssc-miR-novel-chr6_31694 | ssc-mir-novel-chr6_31694 | 3p | TTGCAGTGTGCTGAAACCTCGGC |  |  |  |
| ssc-miR-novel-chr6_31755 | ssc-mir-novel-chr6_31755 | 5p | TCCTCTGGAGGCTCGAGAAGA |  |  |  |
| ssc-miR-novel-chr6_31759 | ssc-mir-novel-chr6_31759 | 5p | TGGCTTGTACTCTAGGTG |  |  |  |
| ssc-miR-novel-chr6_31814 | ssc-mir-novel-chr6_31814 | 3p | TGGGTGGAGGAAAGCGG |  |  |  |
| ssc-miR-novel-chr6_31822 | ssc-mir-novel-chr6_31822 | 3p | GAGTTCTGGGCTGCAGTG | 6 | 7 | 0.941176471 |
| ssc-miR-novel-chr6_31936 | ssc-mir-novel-chr6_31936 | 3p | ACTGCTGTGCTTTCTGCCTGC |  |  |  |
| ssc-miR-novel-chr6_32013 | ssc-mir-novel-chr6_32013 | 5p | CGGAGTGGGTCGGTTTAAGG |  |  |  |
| ssc-miR-novel-chr6_32075 | ssc-mir-novel-chr6_32075 | 5p | ATGATGGAGGAGCGAGCGGCCG |  |  |  |
| ssc-miR-novel-chr6_32122 | ssc-mir-novel-chr6_32122 | 3p | TTCGCCGCGCAGCCTGCTGGGA |  |  |  |
| ssc-miR-novel-chr6_32142 | ssc-mir-novel-chr6_32142 | 3p | GACACACACAGCGCTCCTGGCC |  | 4 | 0.714285714 |
| ssc-miR-novel-chr6_32152 | ssc-mir-novel-chr6_32152 | 3p | GACGGGCAGTGTGCTAGGATCC |  |  |  |
| ssc-miR-novel-chr6_32276 | ssc-mir-novel-chr6_32276 | 3p | CTGGGCCCGGGTGGAGC |  |  |  |
| ssc-miR-novel-chr6_32385 | ssc-mir-novel-chr6_32385 | 5p | TTCCCTTTGTCATCCTTTGCCC |  |  |  |
| ssc-miR-novel-chr6_32446 | ssc-mir-novel-chr6_32446 | 3p | ACTAGAGCGATTGGAAGG |  |  |  |
| ssc-miR-novel-chr6_32462 | ssc-mir-novel-chr6_32462 | 3p | TTGGCATGAATGTCGCGCTGG |  |  |  |
| ssc-miR-novel-chr6_32491 | ssc-mir-novel-chr6_32491 | 5p | CAAAGCGCCAGAGAGGCGGGGA |  |  |  |
| ssc-miR-novel-chr6_32492 | ssc-mir-novel-chr6_32492 | 3p | CAAAGCGCCAGAGAGGCGGGGA |  |  |  |
| ssc-miR-novel-chr6_32552 | ssc-mir-novel-chr6_32552 | 3p | TTGGACTGGAGGTGAGGC |  |  |  |
| ssc-miR-novel-chr6_32557 | ssc-mir-novel-chr6_32557 | 5p | AGCTTATCAGACTGGTGTAG |  |  |  |
| ssc-miR-novel-chr6_32953 | ssc-mir-novel-chr6_32953 | 5p | AAAGGGACACAGGAGGCT |  |  |  |
| ssc-miR-novel-chr7_33108 | ssc-mir-novel-chr7_33108 | 5p | TCTGGTCCAGACACTGTGGAGC |  |  |  |
| ssc-miR-novel-chr7_33374 | ssc-mir-novel-chr7_33374 | 5p | CTTCCAGCAAGGAGTCTCAGA |  |  |  |
| ssc-miR-novel-chr7_33433 | ssc-mir-novel-chr7_33433 | 3p | TCGAGAATTGCGTTTGGACAAT |  |  |  |
| ssc-miR-novel-chr7_33466 | ssc-mir-novel-chr7_33466 | 5p | TGCAGGTGGAGCCCAGGGGA |  |  |  |
| ssc-miR-novel-chr7_33502 | ssc-mir-novel-chr7_33502 | 5p | TGTCATGCTGGGGAGTGTAGTGA |  |  |  |
| ssc-miR-novel-chr7_33539 | ssc-mir-novel-chr7_33539 | 3p | TTTTTTGCTGGAACATTTCTG |  |  |  |
| ssc-miR-novel-chr7_33544 | ssc-mir-novel-chr7_33544 | 5p | TAGGACACAAAATGTAGGAAGGGC |  |  |  |
| ssc-miR-novel-chr7_33656 | ssc-mir-novel-chr7_33656 | 3p | AACGGAAACAATCCAAAACTGT |  |  |  |
| ssc-miR-novel-chr7_33696 | ssc-mir-novel-chr7_33696 | 5p | TCTGGTTCTGTGACCCTGCCT | 4 | 7 | 0.823529412 |
| ssc-miR-novel-chr7_33739 | ssc-mir-novel-chr7_33739 | 3p | ATCTGAAAGTACTGGGGGCCT | 4 |  | 1.4 |
| ssc-miR-novel-chr7_33821 | ssc-mir-novel-chr7_33821 | 3p | CAAAACAGAGAATCAAGACTGA | 9 | 4 | 1.357142857 |
| ssc-miR-novel-chr7_33839 | ssc-mir-novel-chr7_33839 | 3p | CCGCCTCCTCTCGCCGCC | 2 | 2 | 1 |
| ssc-miR-novel-chr7_33933 | ssc-mir-novel-chr7_33933 | 3p | AGTAGATTCATGGATACTCT | 7 |  | 1.7 |
| ssc-miR-novel-chr7_33975 | ssc-mir-novel-chr7_33975 | 3p | TGCATCTGTGGCGTAGGCCAGT | 14 | 37 | 0.510638298 |
| ssc-miR-novel-chr7_34009 | ssc-mir-novel-chr7_34009 | 3p | TCCAGGGTGCTCGCCCCAGC |  |  |  |
| ssc-miR-novel-chr7_34024 | ssc-mir-novel-chr7_34024 | 5p | TGATGCCTGGCATTTTGTGTGT |  |  |  |
| ssc-miR-novel-chr7_34189 | ssc-mir-novel-chr7_34189 | 3p | GTTCAAGTCCAGTTCTG | 13 |  | 2.3 |
| ssc-miR-novel-chr7_34218 | ssc-mir-novel-chr7_34218 | 5p | GTGGTCAAGGCCTGTCGCCGCTGT | 2 |  | 1.2 |
| ssc-miR-novel-chr7_34297 | ssc-mir-novel-chr7_34297 | 3p | TGCATCTGTGGCGTAGGCCAGT | 14 | 37 | 0.510638298 |
| ssc-miR-novel-chr7_34314 | ssc-mir-novel-chr7_34314 | 3p | CCTTTCTGTGTTCGAGGCC |  |  |  |
| ssc-miR-novel-chr7_34318 | ssc-mir-novel-chr7_34318 | 3p | AGGCCCTGGAAAATGACTG |  |  |  |
| ssc-miR-novel-chr7_34332 | ssc-mir-novel-chr7_34332 | 3p | TCTAGCATCGAGCACCCGCCT |  |  |  |
| ssc-miR-novel-chr7_34491 | ssc-mir-novel-chr7_34491 | 5p | TACGTGCTAGTTCTGTACTGGG |  |  |  |
| ssc-miR-novel-chr7_34798 | ssc-mir-novel-chr7_34798 | 3p | AGTCCCATCTGGGTCGCCA | 16 | 4 | 1.857142857 |
| ssc-miR-novel-chr7_34809 | ssc-mir-novel-chr7_34809 | 5p | CAGGTGGTGGACTTTCAGC |  |  |  |
| ssc-miR-novel-chr7_34819 | ssc-mir-novel-chr7_34819 | 5p | TCTCTGGGCCTGTGTCT |  |  |  |
| ssc-miR-novel-chr7_34820 | ssc-mir-novel-chr7_34820 | 3p | TCTCTGGGCCTGTGTCT |  |  |  |
| ssc-miR-novel-chr7_34843 | ssc-mir-novel-chr7_34843 | 5p | GCGGGAGCTCTGTCGCT | 2 |  | 1.2 |
| ssc-miR-novel-chr7_34845 | ssc-mir-novel-chr7_34845 | 5p | AAAGGATGGATTGGACAGGCCT | 2 |  | 1.2 |
| ssc-miR-novel-chr7_34976 | ssc-mir-novel-chr7_34976 | 3p | TGAGAACTCTGCTGAAGG |  |  |  |
| ssc-miR-novel-chr7_35006 | ssc-mir-novel-chr7_35006 | 3p | TTCCAGTGGATCTGGGGATGGA |  |  |  |
| ssc-miR-novel-chr7_35053 | ssc-mir-novel-chr7_35053 | 5p | TGCTGTAATTTACTCTCACC |  |  |  |
| ssc-miR-novel-chr7_35084 | ssc-mir-novel-chr7_35084 | 3p | CAAAACAGAGAATCAAGACTGA | 9 | 4 | 1.357142857 |
| ssc-miR-novel-chr7_35242 | ssc-mir-novel-chr7_35242 | 3p | ATAGCCTTGTTTGTTTTCTAGG |  |  |  |
| ssc-miR-novel-chr7_35271 | ssc-mir-novel-chr7_35271 | 5p | TGGTGTAGATCTCAGACGCAGC |  |  |  |
| ssc-miR-novel-chr7_35273 | ssc-mir-novel-chr7_35273 | 5p | TGATGCCTGGCATTTTGTGTGT |  |  |  |
| ssc-miR-novel-chr7_35375 | ssc-mir-novel-chr7_35375 | 5p | TTGCCCGAGAGCTTGGACCG | 4 | 2 | 1.166666667 |
| ssc-miR-novel-chr7_35436 | ssc-mir-novel-chr7_35436 | 3p | GTGCACCTGGGCAAGGAT |  |  |  |
| ssc-miR-novel-chr7_35438 | ssc-mir-novel-chr7_35438 | 3p | GTGCACCTGGGCAAGGAT |  |  |  |
| ssc-miR-novel-chr7_35451 | ssc-mir-novel-chr7_35451 | 5p | TGTGCTGTGCAGTAGGACC | 2 | 2 | 1 |
| ssc-miR-novel-chr7_35509 | ssc-mir-novel-chr7_35509 | 5p | GGGCGGGGGTCCGCCGG | 2 |  | 1.2 |
| ssc-miR-novel-chr7_35579 | ssc-mir-novel-chr7_35579 | 5p | GTGCCCAGAGTCGGAAGG |  |  |  |
| ssc-miR-novel-chr8_35622 | ssc-mir-novel-chr8_35622 | 5p | CCGGCCGCGCGCGCGCG | 9 |  | 1.9 |
| ssc-miR-novel-chr8_35968 | ssc-mir-novel-chr8_35968 | 5p | TGAGCAAGTAGATTGTATAG |  |  |  |
| ssc-miR-novel-chr8_36091 | ssc-mir-novel-chr8_36091 | 3p | TATAAATTGGAATATCTT |  |  |  |
| ssc-miR-novel-chr8_36140 | ssc-mir-novel-chr8_36140 | 5p | GTGTATGTGCTTGGCTG | 3 |  | 1.3 |
| ssc-miR-novel-chr8_36342 | ssc-mir-novel-chr8_36342 | 5p | TGAGCCACAGAAACTCCAGGAC |  |  |  |
| ssc-miR-novel-chr8_36412 | ssc-mir-novel-chr8_36412 | 5p | GTGAGGGGCAGAGAGAGAGA |  |  |  |
| ssc-miR-novel-chr8_36469 | ssc-mir-novel-chr8_36469 | 3p | TGGACTGGATGACAATCTGCAGG |  |  |  |
| ssc-miR-novel-chr8_36485 | ssc-mir-novel-chr8_36485 | 3p | CAGATGGATGTAGATACAGATC |  |  |  |
| ssc-miR-novel-chr8_36503 | ssc-mir-novel-chr8_36503 | 3p | TTCTGGGAACCGGTTTTGCTGCT |  |  |  |
| ssc-miR-novel-chr8_36529 | ssc-mir-novel-chr8_36529 | 3p | CCTCCGGTCCGCCTGCGGTGG |  |  |  |
| ssc-miR-novel-chr8_36585 | ssc-mir-novel-chr8_36585 | 5p | AGATGGATTTTTGGAGATGG | 8 | 3 | 1.384615385 |
| ssc-miR-novel-chr8_36601 | ssc-mir-novel-chr8_36601 | 5p | CAGCAATTTATTTTCCAGTGA |  |  |  |
| ssc-miR-novel-chr8_36618 | ssc-mir-novel-chr8_36618 | 3p | TCTGTGGTTTGTTGAATCTGTCG |  |  |  |
| ssc-miR-novel-chr8_36650 | ssc-mir-novel-chr8_36650 | 3p | CTCAAGTGATGCCTCTGCTT |  |  |  |
| ssc-miR-novel-chr8_36861 | ssc-mir-novel-chr8_36861 | 5p | AACTGGATGTAAGATAGAATCAT |  |  |  |
| ssc-miR-novel-chr8_37121 | ssc-mir-novel-chr8_37121 | 5p | AACTGGATGTAAGATAGAATCAT |  |  |  |
| ssc-miR-novel-chr8_37190 | ssc-mir-novel-chr8_37190 | 3p | TCTGTGAACTAGAAACCTCTGG | 356 | 136 | 2.506849315 |
| ssc-miR-novel-chr8_37196 | ssc-mir-novel-chr8_37196 | 3p | CTCCTGGCTGGCTCGCCA | 2 |  | 1.2 |
| ssc-miR-novel-chr8_37351 | ssc-mir-novel-chr8_37351 | 5p | GGGCGGGGGTCCGCCGG | 2 |  | 1.2 |
| ssc-miR-novel-chr9_37440 | ssc-mir-novel-chr9_37440 | 5p | CTCCTGGCTGGCTCGCCA | 2 |  | 1.2 |
| ssc-miR-novel-chr9_37600 | ssc-mir-novel-chr9_37600 | 5p | AGGAAGCCCTGGAGGGG |  |  |  |
| ssc-miR-novel-chr9_37672 | ssc-mir-novel-chr9_37672 | 5p | TGGCAGTGGAATTAGTGATTGT |  |  |  |
| ssc-miR-novel-chr9_37686 | ssc-mir-novel-chr9_37686 | 5p | ACTGGCCTTGGAGTCAGAAGT |  |  |  |
| ssc-miR-novel-chr9_37717 | ssc-mir-novel-chr9_37717 | 3p | AGGGCCCTGGCAGGGTGGGA |  |  |  |
| ssc-miR-novel-chr9_37731 | ssc-mir-novel-chr9_37731 | 3p | GCAGCAGAGGAAAGGGTTC |  |  |  |
| ssc-miR-novel-chr9_37946 | ssc-mir-novel-chr9_37946 | 5p | CTGAGAGATGGGAGAGTG | 13 |  | 2.3 |
| ssc-miR-novel-chr9_37990 | ssc-mir-novel-chr9_37990 | 5p | TATGGCTTTTCATTCCTATGTGA | 6 | 13 | 0.695652174 |
| ssc-miR-novel-chr9_38021 | ssc-mir-novel-chr9_38021 | 3p | TCTGGCGGGAAGTTGTGGTC | 13 | 7 | 1.352941176 |
| ssc-miR-novel-chr9_38059 | ssc-mir-novel-chr9_38059 | 3p | CGATACAGAGGACTGACTGTAC |  | 2 | 0.833333333 |
| ssc-miR-novel-chr9_38293 | ssc-mir-novel-chr9_38293 | 3p | CACAGCAAGTGTAGACAGGCA | 4 |  | 1.4 |
| ssc-miR-novel-chr9_38296 | ssc-mir-novel-chr9_38296 | 5p | TAACCAATGTGCAGACTACTGT |  |  |  |
| ssc-miR-novel-chr9_38471 | ssc-mir-novel-chr9_38471 | 3p | TCTCCTTGCGAGTCTCTGCTGCC |  |  |  |
| ssc-miR-novel-chr9_38476 | ssc-mir-novel-chr9_38476 | 5p | CAGCCTGGCTTCTCCTGACATGG | 4 |  | 1.4 |
| ssc-miR-novel-chr9_38517 | ssc-mir-novel-chr9_38517 | 3p | CACGCTTGTGTCGTTGGAGTGGC | 7 |  | 1.7 |
| ssc-miR-novel-chr9_38594 | ssc-mir-novel-chr9_38594 | 3p | TCGTGCACAGATGTGGTCTCG |  |  |  |
| ssc-miR-novel-chr9_38736 | ssc-mir-novel-chr9_38736 | 3p | GACTCCAAAGTCTGCCTC |  |  |  |
| ssc-miR-novel-chr9_38827 | ssc-mir-novel-chr9_38827 | 5p | AGGCAGTGTAATTAGCTGATTGT |  | 4 | 0.714285714 |
| ssc-miR-novel-chr9_38871 | ssc-mir-novel-chr9_38871 | 5p | AGAGACCTGCTGATACAGCTG |  |  |  |
| ssc-miR-novel-chr9_38959 | ssc-mir-novel-chr9_38959 | 5p | TACCTGGTTGATCCTGC | 4 | 7 | 0.823529412 |
| ssc-miR-novel-chr9_39012 | ssc-mir-novel-chr9_39012 | 3p | CTCCCTGAGCTGTCTCTG |  |  |  |
| ssc-miR-novel-chr9_39041 | ssc-mir-novel-chr9_39041 | 5p | TATGGCTTTTCATTCCTATGTGA | 6 | 13 | 0.695652174 |
| ssc-miR-novel-chr9_39126 | ssc-mir-novel-chr9_39126 | 3p | TCACTGGAGTTTTGTTTCAACA | 47 | 4 | 4.071428571 |
| ssc-miR-novel-chr9_39325 | ssc-mir-novel-chr9_39325 | 5p | CCATCTGTGGGATTATGACTG | 9 | 4 | 1.357142857 |
| ssc-miR-novel-chr9_39438 | ssc-mir-novel-chr9_39438 | 3p | TTGAAAGGCTATTTCTTGGTC |  |  |  |
| ssc-miR-novel-chr9_39553 | ssc-mir-novel-chr9_39553 | 5p | TCTGTACGTGGGAGGGTGTGT | 4 |  | 1.4 |
| ssc-miR-novel-chr9_39595 | ssc-mir-novel-chr9_39595 | 5p | TCGGCAGCTGCTTGGCGTCCGAGG |  |  |  |
| ssc-miR-novel-chrPRV_425 | ssc-mir-novel-chrPRV_425 | 3p | TCTCACCCCTGGGTCCGTCGC | 3622 | 809 | 4.434676435 |
| ssc-miR-novel-chrPRV_428 | ssc-mir-novel-chrPRV_428 | 5p | CTCATCCCGTCAGACCTGCGCC | 736 | 842 | 0.875586854 |
| ssc-miR-novel-chrPRV_434 | ssc-mir-novel-chrPRV_434 | 5p | CGTACCGACCCGCCTACCAGGCA | 13 | 37 | 0.489361702 |
| ssc-miR-novel-chrPRV_435 | ssc-mir-novel-chrPRV_435 | 3p | ATGAGTGGATGGATGGAGGCGA | 224 | 307 | 0.738170347 |
| ssc-miR-novel-chrPRV_441 | ssc-mir-novel-chrPRV_441 | 5p | ACCCGCGGATGGCGAGGATG | 15 | 10 | 1.25 |
| ssc-miR-novel-chrX_39845 | ssc-mir-novel-chrX_39845 | 3p | CGGCGGCGACTCTGGACTC | 232 | 307 | 0.76340694 |
| ssc-miR-novel-chrX_39944 | ssc-mir-novel-chrX_39944 | 3p | CTCCCACGTGCAGGGTTTGCA |  | 2 | 0.833333333 |
| ssc-miR-novel-chrX_39950 | ssc-mir-novel-chrX_39950 | 5p | TAATCCTTGCTACCTGGGTGAGA | 4 | 8 | 0.777777778 |
| ssc-miR-novel-chrX_39952 | ssc-mir-novel-chrX_39952 | 5p | TACCCATTGCATATCGGAGTTG | 384 | 370 | 1.036842105 |
| ssc-miR-novel-chrX_39953 | ssc-mir-novel-chrX_39953 | 3p | ATGCACCTGGGCAAGGATT | 3 |  | 1.3 |
| ssc-miR-novel-chrX_39968 | ssc-mir-novel-chrX_39968 | 3p | CTCCAAGCATCGTGACCCAGGTT |  | 3 | 0.769230769 |
| ssc-miR-novel-chrX_39978 | ssc-mir-novel-chrX_39978 | 3p | CTCCAAGCATCGTGACCCAGGTT |  | 3 | 0.769230769 |
| ssc-miR-novel-chrX_40068 | ssc-mir-novel-chrX_40068 | 5p | TGATAATACAACCTGATAAGTGC |  |  |  |
| ssc-miR-novel-chrX_40069 | ssc-mir-novel-chrX_40069 | 5p | TTACAATACAACCTGATAAGTGC |  |  |  |
| ssc-miR-novel-chrX_40077 | ssc-mir-novel-chrX_40077 | 3p | ATTCCTAGAAATTGTTCACAAT |  |  |  |
| ssc-miR-novel-chrX_40247 | ssc-mir-novel-chrX_40247 | 3p | TACCCAGAGCGTGCAGTGTGA | 2 |  | 1.2 |
| ssc-miR-novel-chrX_40249 | ssc-mir-novel-chrX_40249 | 5p | AGGTCCTCAATAAGTATTTGTT |  |  |  |
| ssc-miR-novel-chrX_40252 | ssc-mir-novel-chrX_40252 | 5p | TTCATTCGGCTGTCCAGATGTA | 29 | 48 | 0.672413793 |
| ssc-miR-novel-chrX_40254 | ssc-mir-novel-chrX_40254 | 5p | TGAGTACCGCCATGTCTGTTGGGA |  |  |  |
| ssc-miR-novel-chrX_40255 | ssc-mir-novel-chrX_40255 | 3p | TTGCATATGTAGGATGTCCCA | 5 | 7 | 0.882352941 |
| ssc-miR-novel-chrX_40262 | ssc-mir-novel-chrX_40262 | 5p | CCACCAGAAAGAGACTGTAGGG |  |  |  |
| ssc-miR-novel-chrX_40264 | ssc-mir-novel-chrX_40264 | 5p | CCACCAGAAAGAGACTGTAGGG |  |  |  |
| ssc-miR-novel-chrX_40266 | ssc-mir-novel-chrX_40266 | 5p | CCACCAGAAAGAGACTGTAGGG |  |  |  |
| ssc-miR-novel-chrX_40268 | ssc-mir-novel-chrX_40268 | 5p | CCACCAGAAAGAGACTGTAGGG |  |  |  |
| ssc-miR-novel-chrX_40461 | ssc-mir-novel-chrX_40461 | 3p | CAGAGGCCAGAGGGCAGGACGCT |  |  |  |
| ssc-miR-novel-chrX_40477 | ssc-mir-novel-chrX_40477 | 3p | CAGCAAGGGTGGTCCAGGGAGT |  |  |  |
| ssc-miR-novel-chrX_40484 | ssc-mir-novel-chrX_40484 | 5p | AAGGGCGGGAGCGGCGGG | 69 | 90 | 0.79 |
| ssc-miR-novel-chrX_40486 | ssc-mir-novel-chrX_40486 | 5p | TGCACGGAGCAGCAGGGTCTGA | 3 |  | 1.3 |
| ssc-miR-novel-chrX_40490 | ssc-mir-novel-chrX_40490 | 5p | GTAGGGCCGGCCCGCCCGGGA |  |  |  |
| ssc-miR-novel-chrX_40522 | ssc-mir-novel-chrX_40522 | 3p | CATGCCTTGAGTGTAGGAC | 41 | 31 | 1.243902439 |
| ssc-miR-novel-chrX_40528 | ssc-mir-novel-chrX_40528 | 3p | AACCAGACTCTGAGAGCAGGACT |  |  |  |
| ssc-miR-novel-chrX_40533 | ssc-mir-novel-chrX_40533 | 5p | GAAAATGGATGGCACTGGAGT | 37 | 8 | 2.611111111 |
| ssc-miR-novel-chrX_40539 | ssc-mir-novel-chrX_40539 | 5p | TGGTCTAGCGGTTAGGA |  |  |  |
| ssc-miR-novel-chrX_40608 | ssc-mir-novel-chrX_40608 | 5p | GGTGGAGGCATGTAGTTCCTAA |  |  |  |
| ssc-miR-novel-chrX_40705 | ssc-mir-novel-chrX_40705 | 5p | AGCTACATTGTCTGCTGGGTTT | 2545 | 845 | 2.988304094 |
| ssc-miR-novel-chrX_40783 | ssc-mir-novel-chrX_40783 | 5p | GTATGTGAGCGGGGGGCTGGTGGGA |  |  |  |
| ssc-miR-novel-chrX_40912 | ssc-mir-novel-chrX_40912 | 3p | AAAGCTGTGGATTCTGGCAAATGG |  |  |  |
| ssc-miR-novel-chrX_41000 | ssc-mir-novel-chrX_41000 | 5p | CACCTGGGAGGATCGGAG |  |  |  |
| ssc-miR-novel-chrX_41021 | ssc-mir-novel-chrX_41021 | 5p | CCACCAGAAAGAGACTGTAGGG |  |  |  |
| ssc-miR-novel-chrX_41061 | ssc-mir-novel-chrX_41061 | 5p | CCTGGACTTGAAGTCAGAAGGC | 2 |  | 1.2 |
| ssc-miR-novel-chrX_41131 | ssc-mir-novel-chrX_41131 | 5p | TGGAAGGTGGATCTGGGC |  |  |  |
| ssc-miR-novel-chrX_41178 | ssc-mir-novel-chrX_41178 | 3p | TGAACGGTGCCTGTGTGGCTAGA |  |  |  |
| ssc-miR-novel-chrX_41179 | ssc-mir-novel-chrX_41179 | 5p | TATCCAGACAGGTGCTGTTCT |  |  |  |
| ssc-miR-novel-chrX_41184 | ssc-mir-novel-chrX_41184 | 3p | TGAACGGCGCCTGTGTGGTTAGA |  |  |  |
| ssc-miR-novel-chrX_41186 | ssc-mir-novel-chrX_41186 | 3p | TGAACAGGGCCTTTCTGGGTCGAG |  |  |  |
| ssc-miR-novel-chrX_41188 | ssc-mir-novel-chrX_41188 | 3p | CAACAGTCCCTGCCTGGGTAGA |  |  |  |
| ssc-miR-novel-chrX_41190 | ssc-mir-novel-chrX_41190 | 3p | AATGGCGCTTTTTTGTGAAGA | 2 | 2 | 1 |
| ssc-miR-novel-chrX_41192 | ssc-mir-novel-chrX_41192 | 3p | TGAATGGCGCCTTTCTGAGTAGA | 6 |  | 1.6 |
| ssc-miR-novel-chrX_41194 | ssc-mir-novel-chrX_41194 | 3p | TGATTGGCACCTCTTTGAGTGA |  |  |  |
| ssc-miR-novel-chrX_41196 | ssc-mir-novel-chrX_41196 | 3p | ACTGTCACCTTTTTGAGTAGA | 4 |  | 1.4 |
| ssc-miR-novel-chrX_41200 | ssc-mir-novel-chrX_41200 | 3p | TGATTGACACCTCTGTTAGTGGA |  | 2 | 0.833333333 |
| ssc-miR-novel-chrX_41202 | ssc-mir-novel-chrX_41202 | 3p | TGATTGGCACCTCTTGGAGTGA | 2 |  | 1.2 |
| ssc-miR-novel-GL892353-1_41323 | ssc-mir-novel-GL892353-1_41323 | 5p | CGACATGGACACGCGTCATGA |  |  |  |
| ssc-miR-novel-GL892520-2_41438 | ssc-mir-novel-GL892520-2_41438 | 5p | GGTGATGATGACGATGAAGCTGAAA |  |  |  |
| ssc-miR-novel-GL892841-1_41670 | ssc-mir-novel-GL892841-1_41670 | 5p | GAGACCATGGAGAGAAAAA | 4 |  | 1.4 |
| ssc-miR-novel-GL892848-2_41682 | ssc-mir-novel-GL892848-2_41682 | 5p | GTGGTCAAGGCCTGTCGCCGCTGT | 2 |  | 1.2 |
| ssc-miR-novel-GL892871-2_41708 | ssc-mir-novel-GL892871-2_41708 | 5p | TTCAAGTAATTCAGGATAGGTT | 2094 | 3921 | 0.535232765 |
| ssc-miR-novel-GL892918-2_41750 | ssc-mir-novel-GL892918-2_41750 | 5p | CTAGAGTGGATTCTGCA |  |  |  |
| ssc-miR-novel-GL892946-2_41815 | ssc-mir-novel-GL892946-2_41815 | 3p | TCGGAGCAGCGCGCGAGA | 6 | 6 | 1 |
| ssc-miR-novel-GL892961-2_41842 | ssc-mir-novel-GL892961-2_41842 | 3p | CAGACCCTGAGCTGCCTCTAGA | 3 |  | 1.3 |
| ssc-miR-novel-GL893103-2_41962 | ssc-mir-novel-GL893103-2_41962 | 3p | AGTCCTCTCCTGGGCACCT |  | 11 | 0.476190476 |
| ssc-miR-novel-GL893138-2_41998 | ssc-mir-novel-GL893138-2_41998 | 5p | GGCTCCCTCCACCCGCC |  | 3 | 0.769230769 |
| ssc-miR-novel-GL893173-1_42029 | ssc-mir-novel-GL893173-1_42029 | 3p | CCTTCGAGGATGCGGATGTCACC | 2 |  | 1.2 |
| ssc-miR-novel-GL893222-2_42067 | ssc-mir-novel-GL893222-2_42067 | 3p | AGAGGGCTGTGGGAGAGA | 10 |  | 2 |
| ssc-miR-novel-GL893230-2_42082 | ssc-mir-novel-GL893230-2_42082 | 3p | CTGTGCTTCTGCTCGGA |  |  |  |
| ssc-miR-novel-GL893233-1_42087 | ssc-mir-novel-GL893233-1_42087 | 5p | ATTGAGAACACTGACATAACA |  |  |  |
| ssc-miR-novel-GL893271-1_42121 | ssc-mir-novel-GL893271-1_42121 | 5p | AGAGGTGCGGCTTTGGCTGGA |  |  |  |
| ssc-miR-novel-GL893334-2_42198 | ssc-mir-novel-GL893334-2_42198 | 5p | TCTGGAGGACGCTAGAGCTGGGC |  |  |  |
| ssc-miR-novel-GL893334-2_42199 | ssc-mir-novel-GL893334-2_42199 | 3p | CAGAAGGGGAGTCGGAGCAGA |  |  |  |
| ssc-miR-novel-GL893571-2_42428 | ssc-mir-novel-GL893571-2_42428 | 5p | CACGGGTTCGATCCCTGGTGTGGGC |  |  |  |
| ssc-miR-novel-GL893616-2_42510 | ssc-mir-novel-GL893616-2_42510 | 3p | CTCAGACCTCAGGCTTGGAGCC | 3 |  | 1.3 |
| ssc-miR-novel-GL893653-2_42520 | ssc-mir-novel-GL893653-2_42520 | 5p | TTCAGGGTCCAGGATTGCTATAG | 2 |  | 1.2 |
| ssc-miR-novel-GL893741-1_42616 | ssc-mir-novel-GL893741-1_42616 | 5p | GGTGAGCACTCTGGACT |  |  |  |
| ssc-miR-novel-GL893763-1_42633 | ssc-mir-novel-GL893763-1_42633 | 3p | TCTTTCCTTGATTAAAACTGG | 4 |  | 1.4 |
| ssc-miR-novel-GL893833-1_42684 | ssc-mir-novel-GL893833-1_42684 | 3p | TTTTTGATGGGAGAAGAGAGA |  |  |  |
| ssc-miR-novel-GL894036-2_42873 | ssc-mir-novel-GL894036-2_42873 | 3p | CAAACCAGCACGTCAAGGGCCC |  |  |  |
| ssc-miR-novel-GL894094-2_42949 | ssc-mir-novel-GL894094-2_42949 | 3p | GCATTGGGGGTTCAGGGG | 12 | 14 | 0.916666667 |
| ssc-miR-novel-GL894224-1_43070 | ssc-mir-novel-GL894224-1_43070 | 5p | CTTTGGATCTCTGGTGACAGG |  |  |  |
| ssc-miR-novel-GL894231-1_43077 | ssc-mir-novel-GL894231-1_43077 | 5p | TGGTCGACCAGTTGGAAAGTAAT | 7 | 3 | 1.307692308 |
| ssc-miR-novel-GL894231-1_43090 | ssc-mir-novel-GL894231-1_43090 | 3p | CCCAATACACGGTCGATCTCT |  | 2 | 0.833333333 |
| ssc-miR-novel-GL894231-1_43091 | ssc-mir-novel-GL894231-1_43091 | 5p | AGAGGCTGGCCGTGATGAATTCG |  |  |  |
| ssc-miR-novel-GL894231-1_43093 | ssc-mir-novel-GL894231-1_43093 | 5p | TGTGACTGGTTGACCAGAGGGT |  |  |  |
| ssc-miR-novel-GL894231-1_43098 | ssc-mir-novel-GL894231-1_43098 | 3p | TGTATGTCAACTGATCCACAGT |  |  |  |
| ssc-miR-novel-GL894231-1_43100 | ssc-mir-novel-GL894231-1_43100 | 3p | ATAATACATGGTTAACCTCTTT | 2 |  | 1.2 |
| ssc-miR-novel-GL894231-1_43101 | ssc-mir-novel-GL894231-1_43101 | 5p | GGAGAAATTATCCTTGGTGTGTT |  |  |  |
| ssc-miR-novel-GL894231-1_43114 | ssc-mir-novel-GL894231-1_43114 | 3p | ATCATAGAGGAAAATCCACA |  |  |  |
| ssc-miR-novel-GL894266-1_43136 | ssc-mir-novel-GL894266-1_43136 | 5p | TTGTTTCTTGCATGTGCTCTGATT |  |  |  |
| ssc-miR-novel-GL894404-1_43244 | ssc-mir-novel-GL894404-1_43244 | 3p | TTGTGCGTTGGCAGGATGGGCCGGA |  |  |  |
| ssc-miR-novel-GL894430-2_43273 | ssc-mir-novel-GL894430-2_43273 | 3p | ATAAAACTTAGTCGCTGCT |  |  |  |
| ssc-miR-novel-GL894430-2_43278 | ssc-mir-novel-GL894430-2_43278 | 3p | ATAAAACTTAGTCGCTGCT |  |  |  |
| ssc-miR-novel-GL894520-2_43333 | ssc-mir-novel-GL894520-2_43333 | 3p | CTCAAGTGATGCCTCTGCTT |  |  |  |
| ssc-miR-novel-GL894542-2_43355 | ssc-mir-novel-GL894542-2_43355 | 5p | TTTGCTCTGCTCCTGCCACAT |  | 2 | 0.833333333 |
| ssc-miR-novel-GL894570-2_43376 | ssc-mir-novel-GL894570-2_43376 | 3p | TGTTGTCCTCCGAATCTGAAT |  |  |  |
| ssc-miR-novel-GL894677-1_43524 | ssc-mir-novel-GL894677-1_43524 | 3p | TTCACTTTGGATTTTCAGCC | 12 |  | 2.2 |
| ssc-miR-novel-GL894726-2_43559 | ssc-mir-novel-GL894726-2_43559 | 3p | CGGGGCAGCTCAGTACAGGAC |  |  |  |
| ssc-miR-novel-GL894726-2_43560 | ssc-mir-novel-GL894726-2_43560 | 5p | CGGGGCAGCTCAGTACAGGAC |  |  |  |
| ssc-miR-novel-GL894850-1_43647 | ssc-mir-novel-GL894850-1_43647 | 5p | CAGGACTGGTGACTGGGGTG |  |  |  |
| ssc-miR-novel-GL894875-2_43662 | ssc-mir-novel-GL894875-2_43662 | 5p | TGAGGTGGTAGATGGTATAG |  |  |  |
| ssc-miR-novel-GL894932-2_43717 | ssc-mir-novel-GL894932-2_43717 | 5p | TTCCAGGGAAGAAAGGAGGAAC | 2 |  | 1.2 |
| ssc-miR-novel-GL895030-2_43791 | ssc-mir-novel-GL895030-2_43791 | 5p | GCAGAGCAGCGGCAGGAGG |  |  |  |
| ssc-miR-novel-GL895143-1_43893 | ssc-mir-novel-GL895143-1_43893 | 5p | TTCCTGGGTCTCGTGGTCTCAGTCT |  |  |  |
| ssc-miR-novel-GL895351-2_44043 | ssc-mir-novel-GL895351-2_44043 | 5p | GCGCAGCACATCATGGTTTA |  |  |  |
| ssc-miR-novel-GL895485-2_44130 | ssc-mir-novel-GL895485-2_44130 | 3p | AGGCTCTGATTGGATTGGGTG |  |  |  |
| ssc-miR-novel-GL895563-1_44191 | ssc-mir-novel-GL895563-1_44191 | 3p | TAGCCTGACGCTGATGATTGT |  |  |  |
| ssc-miR-novel-GL895621-2_44210 | ssc-mir-novel-GL895621-2_44210 | 5p | TCTTCCATGTGCCACGGGTGTAGCT |  |  |  |
| ssc-miR-novel-GL896133-1_44557 | ssc-mir-novel-GL896133-1_44557 | 5p | TGCTCAGAGGTCGGAGGTGGAG |  |  |  |
| ssc-miR-novel-GL896157-1_44574 | ssc-mir-novel-GL896157-1_44574 | 3p | TGGGCTCCAAACCCCTGTCCAGG |  |  |  |
| ssc-miR-novel-GL896208-1_44612 | ssc-mir-novel-GL896208-1_44612 | 3p | TCTGGCCTTGGCCTCAGGCCTGGC |  |  |  |
| ssc-miR-novel-GL896241-2_44636 | ssc-mir-novel-GL896241-2_44636 | 5p | TTCTGGAAGATGTAGTCTGGA |  |  |  |
| ssc-miR-novel-GL896292-1_44714 | ssc-mir-novel-GL896292-1_44714 | 3p | CTTCTCTGTTACTTCCCTCAGG |  |  |  |
| ssc-miR-novel-GL896302-1_44731 | ssc-mir-novel-GL896302-1_44731 | 3p | GCAAAGCACACGGCCTGCAGAGA | 4 |  | 1.4 |
| ssc-miR-novel-GL896425-1_44856 | ssc-mir-novel-GL896425-1_44856 | 3p | TGGTGCCTGACGTCTTGGCAGT |  |  |  |
| ssc-miR-novel-GL896485-1_44921 | ssc-mir-novel-GL896485-1_44921 | 3p | AGAGGAGCCGCGACAGAGCCGG | 2 | 2 | 1 |
| ssc-miR-novel-GL896501-1_44926 | ssc-mir-novel-GL896501-1_44926 | 5p | TGTGGGGCCACGCCCTCAGGCTGT | 4 | 2 | 1.166666667 |
| ssc-miR-novel-JH118484-1_41578 | ssc-mir-novel-JH118484-1_41578 | 5p | CCGGACTCTCTAAATCTCACCT |  |  |  |
| ssc-miR-novel-JH118486-1_41595 | ssc-mir-novel-JH118486-1_41595 | 3p | TTATCCGGGCTTGTGCTGAAGC | 13 | 8 | 1.277777778 |
| ssc-miR-novel-JH118486-1_41599 | ssc-mir-novel-JH118486-1_41599 | 5p | AGCACTTACGGATTCTGGGGG | 2 |  | 1.2 |
| ssc-miR-novel-JH118494-1_41628 | ssc-mir-novel-JH118494-1_41628 | 3p | TCTGAGATGTGACCTGGGCAT | 32 | 29 | 1.076923077 |
| ssc-miR-novel-JH118511-1_41721 | ssc-mir-novel-JH118511-1_41721 | 3p | ATTGGGACTGAGACACG | 14 |  | 2.4 |
| ssc-miR-novel-JH118523-1_41818 | ssc-mir-novel-JH118523-1_41818 | 5p | AAGCCAGAGTCAGGGGACACTGT |  |  |  |
| ssc-miR-novel-JH118527-1_41847 | ssc-mir-novel-JH118527-1_41847 | 3p | CTCACGTCCATGGTCAGCACCGTG | 2 |  | 1.2 |
| ssc-miR-novel-JH118570-1_42024 | ssc-mir-novel-JH118570-1_42024 | 3p | CTCTCGGCTGCTGAGGCCT |  |  |  |
| ssc-miR-novel-JH118585-1_42104 | ssc-mir-novel-JH118585-1_42104 | 5p | TCTGGTCCAGACACTGTGGAGC |  |  |  |
| ssc-miR-novel-JH118620-1_42301 | ssc-mir-novel-JH118620-1_42301 | 3p | AATGATGCCCCTTAGAGTTGAGC |  |  |  |
| ssc-miR-novel-JH118636-1_42370 | ssc-mir-novel-JH118636-1_42370 | 3p | AGAGGGAGTAGGTTTCATA |  |  |  |
| ssc-miR-novel-JH118644-1_42413 | ssc-mir-novel-JH118644-1_42413 | 3p | GACTCCAAAGTCTGCCTC |  |  |  |
| ssc-miR-novel-JH118647-1_42435 | ssc-mir-novel-JH118647-1_42435 | 3p | TGTGTGGCTAAGTGGTAGATTT |  |  |  |
| ssc-miR-novel-JH118654-1_42487 | ssc-mir-novel-JH118654-1_42487 | 5p | TGGCAGTGTATTGTTAGCTGGT | 119 | 40 | 2.58 |
| ssc-miR-novel-JH118655-1_42490 | ssc-mir-novel-JH118655-1_42490 | 5p | AACACATGTGTGCTGAGTGGAACT | 2 |  | 1.2 |
| ssc-miR-novel-JH118656-1_42504 | ssc-mir-novel-JH118656-1_42504 | 5p | TGGCTCATTTCAGCAGGAAG | 4 |  | 1.4 |
| ssc-miR-novel-JH118676-1_42617 | ssc-mir-novel-JH118676-1_42617 | 3p | ACTGTGTGTGAGGAAGTC |  |  |  |
| ssc-miR-novel-JH118774-1_43297 | ssc-mir-novel-JH118774-1_43297 | 3p | TAACCCGACATTCAAGGCCTGT |  |  |  |
| ssc-miR-novel-JH118788-1_43363 | ssc-mir-novel-JH118788-1_43363 | 5p | AAAGGATGGATTGGACAGGCCT | 2 |  | 1.2 |
| ssc-miR-novel-JH118806-1_43515 | ssc-mir-novel-JH118806-1_43515 | 3p | ATTCATATGCAGCTGTAGGAA |  |  |  |
| ssc-miR-novel-JH118806-1_43518 | ssc-mir-novel-JH118806-1_43518 | 3p | ATTCATATGCAGCTGTAGGAA |  |  |  |
| ssc-miR-novel-JH118928-1_44242 | ssc-mir-novel-JH118928-1_44242 | 3p | TCATTCTCCTTCTTTGACCAGA |  |  |  |
| ssc-miR-novel-JH118939-1_44269 | ssc-mir-novel-JH118939-1_44269 | 5p | GACTTAATGGCTGGCTGGGAGG | 39 |  | 4.9 |
| ssc-miR-novel-JH118943-1_44283 | ssc-mir-novel-JH118943-1_44283 | 3p | TGAATTACTGTGGATTCTTGG |  |  |  |
| ssc-miR-novel-JH118951-1_44333 | ssc-mir-novel-JH118951-1_44333 | 3p | TCAAGGTCCGCTGTGAACACGG | 4 |  | 1.4 |
| ssc-miR-novel-JH118990-1_44625 | ssc-mir-novel-JH118990-1_44625 | 3p | CACGCTTGTGTCGTTGGAGTGGC | 7 |  | 1.7 |
| ssc-miR-novel-JH118993-1_44650 | ssc-mir-novel-JH118993-1_44650 | 3p | TTGGACTGGAGGTGAGGC |  |  |  |
| ssc-miR-novel-JH118993-1_44655 | ssc-mir-novel-JH118993-1_44655 | 5p | AGCTTATCAGACTGGTGTAG |  |  |  |

**Table S2. Fold-change levels of DE miRNAs (top 10)**

| Group | miRNA | Pre-miRNA | Type | Fold-change |
| --- | --- | --- | --- | --- |
| PRV Fa wild strain infected / Non-infected | ssc-miR-10b | ssc-mir-10b | Up-regulated | 149.1638225 |
| PRV Fa wild strain infected / Non-infected | ssc-miR-30a-5p | ssc-mir-30a | Up-regulated | 16.30505529 |
| PRV Fa wild strain infected / Non-infected | ssc-miR-10a-5p | ssc-mir-10a | Up-regulated | 7.377477736 |
| PRV Fa wild strain infected / Non-infected | ssc-miR-192 | ssc-mir-192 | Up-regulated | 146.0982143 |
| PRV Fa wild strain infected / Non-infected | ssc-miR-26a | ssc-mir-26a | Up-regulated | 4.544264339 |
| PRV Fa wild strain infected / Non-infected | ssc-miR-16 | ssc-mir-16-1 | Up-regulated | 7.174118885 |
| PRV Fa wild strain infected / Non-infected | ssc-miR-16 | ssc-mir-16-2 | Up-regulated | 7.174118885 |
| PRV Fa wild strain infected / Non-infected | ssc-miR-182 | ssc-mir-182 | Up-regulated | 2.652183312 |
| PRV Fa wild strain infected / Non-infected | ssc-miR-30d | ssc-mir-30d | Up-regulated | 5.951002227 |
| PRV Fa wild strain infected / Non-infected | ssc-let-7i | ssc-let-7i | Up-regulated | 2.13881108 |
| PRV Fa wild strain infected / Non-infected | ssc-miR-21 | ssc-mir-21 | Down-regulated | 0.101823 |
| PRV Fa wild strain infected / Non-infected | ssc-let-7f | ssc-let-7f-1 | Down-regulated | 0.244598 |
| PRV Fa wild strain infected / Non-infected | ssc-let-7f | ssc-let-7f-2 | Down-regulated | 0.244598 |
| PRV Fa wild strain infected / Non-infected | ssc-miR-100 | ssc-mir-100 | Down-regulated | 0.345478 |
| PRV Fa wild strain infected / Non-infected | ssc-miR-151-3p | ssc-mir-151 | Down-regulated | 0.303794 |
| PRV Fa wild strain infected / Non-infected | ssc-miR-148b-3p | ssc-mir-148b | Down-regulated | 0.237392 |
| PRV Fa wild strain infected / Non-infected | ssc-miR-19b | ssc-mir-19b-1 | Down-regulated | 0.02569 |
| PRV Fa wild strain infected / Non-infected | ssc-miR-19b | ssc-mir-19b-2 | Down-regulated | 0.02569 |
| PRV Fa wild strain infected / Non-infected | ssc-miR-18a | ssc-mir-18a | Down-regulated | 0.037692 |
| PRV Fa wild strain infected / Non-infected | ssc-let-7d-5p | ssc-let-7d | Down-regulated | 0.1576 |
| PRV FaΔgE/gI strain infected / Non-infected | ssc-miR-30a-5p | ssc-mir-30a | Up-regulated | 27.23207 |
| PRV FaΔgE/gI strain infected / Non-infected | ssc-miR-10b | ssc-mir-10b | Up-regulated | 118.8737 |
| PRV FaΔgE/gI strain infected / Non-infected | ssc-miR-182 | ssc-mir-182 | Up-regulated | 5.177691 |
| PRV FaΔgE/gI strain infected / Non-infected | ssc-miR-10a-5p | ssc-mir-10a | Up-regulated | 4.543235 |
| PRV FaΔgE/gI strain infected / Non-infected | ssc-miR-192 | ssc-mir-192 | Up-regulated | 125.5179 |
| PRV FaΔgE/gI strain infected / Non-infected | ssc-miR-30d | ssc-mir-30d | Up-regulated | 6.655345 |
| PRV FaΔgE/gI strain infected / Non-infected | ssc-let-7i | ssc-let-7i | Up-regulated | 2.648615 |
| PRV FaΔgE/gI strain infected / Non-infected | ssc-miR-27b-3p | ssc-mir-27b | Up-regulated | 38.48352 |
| PRV FaΔgE/gI strain infected / Non-infected | ssc-miR-98 | ssc-mir-98 | Up-regulated | 2.709556 |
| PRV FaΔgE/gI strain infected / Non-infected | ssc-miR-16 | ssc-mir-16-1 | Up-regulated | 3.340347 |
| PRV FaΔgE/gI strain infected / Non-infected | ssc-miR-21 | ssc-mir-21 | Down-regulated | 0.157737 |
| PRV FaΔgE/gI strain infected / Non-infected | ssc-let-7f | ssc-let-7f-1 | Down-regulated | 0.249025 |
| PRV FaΔgE/gI strain infected / Non-infected | ssc-let-7f | ssc-let-7f-2 | Down-regulated | 0.249025 |
| PRV FaΔgE/gI strain infected / Non-infected | ssc-miR-151-3p | ssc-mir-151 | Down-regulated | 0.360136 |
| PRV FaΔgE/gI strain infected / Non-infected | ssc-miR-148b-3p | ssc-mir-148b | Down-regulated | 0.478475 |
| PRV FaΔgE/gI strain infected / Non-infected | ssc-miR-novel-chr13_10861 | ssc-mir-novel-chr13_10861 | Down-regulated | 0.456129 |
| PRV FaΔgE/gI strain infected / Non-infected | ssc-miR-423-5p | ssc-mir-423 | Down-regulated | 0.24131 |
| PRV FaΔgE/gI strain infected / Non-infected | ssc-miR-146a-5p | ssc-mir-146a | Down-regulated | 0.247046 |
| PRV FaΔgE/gI strain infected / Non-infected | ssc-let-7d-5p | ssc-let-7d | Down-regulated | 0.214246 |
| PRV FaΔgE/gI strain infected / Non-infected | ssc-miR-152 | ssc-mir-152 | Down-regulated | 0.035895 |

**Table S3. Primers used for stem-loop qRT-PCR.**

| **Genes** | **Primers** |
| --- | --- |
| U6 | F:5’TCGCTTTGGCAGCACCTAT3’ R:5’AATATGGAACGCTTCGCAAA3’ |
| ssc-miR-10b | GSP:5’TACCCTGTAGAACCGAATTTGT3’ R:5’GTCGGTGTCGTGGAGTCG3’ |
| ssc-miR-24-3p | GSP:5’TGGCTCAGTTCAGCAGGAACAG3’ R:5’GTCGGTGTCGTGGAGTCG3’ |
| ssc-miR-19b | GSP:5’TGTGCAAATCCATGCAAAACTGA3’ R:5’GTCGGTGTCGTGGAGTCG3’ |
| ssc-let-7f | GSP:5’TGAGGTAGTAGATTGTATAGTT3’ R:5’GTCGGTGTCGTGGAGTCG3’ |
| ssc-miR-30a-5p | GSP:5’TGTAAACATCCTCGACTGGAAG3’ R:5’GTCGGTGTCGTGGAGTCG3’ |
| ssc-miR-21 | GSP:5’TAGCTTATCAGACTGATGTTGA3’ R:5’GTCGGTGTCGTGGAGTCG3’ |
| ssc-miR-182 | GSP:5’TTTGGCAATGGTAGAACTCACACT3’ R:5’GTCGGTGTCGTGGAGTCG3’ |
| ssc-miR-192 | GSP:5’CTGACCTATGAATTGACAGCC3’ R:5’GTCGGTGTCGTGGAGTCG3’ |
| ssc-miR-19b | GSP:5’TGTGCAAATCCATGCAAAACTGA3’ R:5’GTCGGTGTCGTGGAGTCG3’ |
| ssc-miR-24-3p | GSP:5’TGGCTCAGTTCAGCAGGAACAG3’ R:5’GTCGGTGTCGTGGAGTCG3’ |
| ssc-miR-novel-chrPRV_425 | GSP:5’TCTCACCCCTGGGTCCGTCGC3’ R:5’GTCGGTGTCGTGGAGTCG3’ |
| ssc-miR-novel-chrPRV_428 | GSP:5’CTCATCCCGTCAGACCTGCGCC3’ R:5’GTCGGTGTCGTGGAGTCG3’ |
| ssc-miR-novel-chrPRV_434 | GSP:5’CGTACCGACCCGCCTACCAGGCA3’ R:5’GTCGGTGTCGTGGAGTCG3’ |
| ssc-miR-novel-chrPRV_435 | GSP:5’ATGAGTGGATGGATGGAGGCGA3’ R:5’GTCGGTGTCGTGGAGTCG3’ |
| ssc-miR-novel-chrPRV_441 | GSP:5’ACCCGCGGATGGCGAGGATG3’ R:5’GTCGGTGTCGTGGAGTCG3’ |
|  |  |

**Table S4. Target prediction for DE miRNAs.**

Up-regulated in PRV Fa wild strain infected PK-15 cells

ssc-miR-10b

ssc-miR-30a-5p

ssc-miR-10a-5p

ssc-miR-192

ssc-miR-26a

Down-regulated in PRV Fa wild strain infected PK-15 cells

ssc-miR-21

ssc-let-7f

ssc-miR-19b

ssc-miR-24-3p

ssc-miR-152

Up-regulated in PRV FaΔgE/gI strain infected PK-15 cells

ssc-miR-30a-5p

ssc-miR-10b

ssc-miR-182

ssc-miR-10a-5p

ssc-miR-192

Down-regulated in PRV FaΔgE/gI strain infected PK-15 cells

ssc-miR-21

ssc-miR-152

ssc-miR-18a

ssc-miR-18a

ssc-miR-100

ssc-miR-19a

**Table S5. DE miRNAs in vivo and vitro under viral infection**

| **Mature miRNA** | **Type** | **JEV (Japanses Encephalitis virus) infected Porcine kidney epithelial cells** | **PPV (Porcine parvovirus) infected PK-15 cells** | **PCMV infected macrophages**  **Fold change value** | **PCMV infected lung Fold change value** | **PCMV infected**  **Liver Fold change value** | **PCMV infected**  **thymus Fold change value** | **Fa wild strain infected PK-15 cells Fold change value** | **PRV FaΔgE/gI strain infected PK-15 cells Fold change value** |
| --- | --- | --- | --- | --- | --- | --- | --- | --- | --- |
| Ssc-miR-101 | Up-regulated | 5.49 |  | 18.60 | 4.18 |  |  |  |  |
| Ssc-miR-1 | Up-regulated | 8.03 |  | 3.04 | 3.78 |  |  |  |  |
| Ssc-miR-10a | Up-regulated | 4.04 |  | 3 | 3.78 | 3 | 2.53 | 7.38 | 4.54 |
| Ssc-miR-128 | Up-regulated |  |  | 4.97 |  |  | 2.53 | 4.63 |  |
| Ssc-miR-155-5p | Up-regulated |  | 6.06 | 19.67 | 6.26 | 3.39 |  | 60.86 | 10.6 |
| Ssc-miR-192 | Up-regulated | 33.7 |  | 2.45 | 10.55 |  |  | 146.1 | 125.52 |
| Ssc-miR-450c-5p | Up-regulated | 14.81 |  | 3.13 |  |  |  | 10.88 | 8.31 |
| Ssc-miR-7d-5p | Down-regulated | 0.22 |  | 0.10 | 0.12 | 0.05 |  |  |  |
| Ssc-miR-15a | Down-regulated | 0.35 |  | 0.22 |  | 0.18 |  | 0.31 |  |
| Ssc-miR-185 | Down-regulated | 0.23 | 0.04 | 0.07 | 0.47 |  |  | 0.01 |  |
| Ssc-miR-18a | Down-regulated | 0.01 | 0.01 | 0.13 |  |  | 0.11 | 0.04 | 0.08 |
| Ssc-miR-19a | Down-regulated | 0.41 |  | 0.27 |  |  | 0.28 | 0.03 |  |
| Ssc-miR-22-3p | Down-regulated | 0.03 |  | 0.44 | 0.36 |  |  |  |  |
| Ssc-miR-27b-5p | Down-regulated | 0.37 |  | 0.34 | 0.35 |  |  | 0.19 |  |
| Ssc-miR-28-5p | Down-regulated | 0.39 |  | 0.22 | 0.13 |  |  | 0.07 |  |
| Ssc-miR-301 | Down-regulated | 0.37 |  | 0.37 | 0.36 | 0.5 |  | 0.37 |  |
| Ssc-miR-421-3p | Down-regulated | 0.27 |  | 0.29 | 0.19 | 0.25 |  |  |  |

The fold change cutoffs of the upregulated miRNAs and the downregulated miRNAs were 2 and 0.5.
